# Supplementary material for: Prospective observational study to examine health-related quality of life and develop models to predict long-term patient-reported outcomes 6 months after hospital discharge with blunt thoracic injuries
Source: BMJ Open. 2021 Jul 8;11(7):e049292. doi: 10.1136/bmjopen-2021-049292 (PMC8268921; doi:10.1136/bmjopen-2021-049292)
Supplement: Supplementary data [file bmjopen-2021-049292supp001.pdf]

Supplementary file: Table S1: Extended Characteristics of the Sample/Candidate predictor variables

Result: n= (%) unless otherwise indicated	**Total sample** (n=337)	**6/12 PCS≤35** (n=77)	**6/12 PCS>35** (n=134)	p-value	**6/12 Pain Severity Score ≥3.5** (n=77)	**6/12 Pain Severity Score <3.5** (n=134)	p-value	**6/12 PDQ score ≥12** (n=47)	**6/12 PDQ score <12** (n=163)	p-value
Age (Mean (±SD))	62.0 (16.5)	69.1 (13.9)	60.8 (16.3)	<0.001*	66.7 (16.4)	62.1 (15.6)	0.05	63.4 (17.0)	63.9 (15.7)	0.80
Male	231 (68.5)	49 (63.6)	97 (72.4)	0.20	54 (70.1)	91 (67.9)	0.80	33 (70.2)	112 (68.7)	0.90
Injury Severity Score (Mean (±SD))	11.2 (7.3)	10.9 (7.8)	11.4 (6.8)	0.60	11.3 (7.9)	11.2 (6.8)	1.0	11.6 (8.3)	11.2 (6.9)	0.80
Battle/StUMBle Score (Mean (±SD))	22.2 (12.0)	23.8 (11.0)	21.8 (11.3)	0.20	23.9 (11.4)	21.8 (11.0)	0.20	24.2 (12.0)	22.1 (10.9)	0.30
**Mechanism of Injury:**										
RTC	78 (23.1)	14 (18.2)	38 (28.4)		19 (24.7)	33 (24.6)		9 (19.1)	43 (26.4)	
Crush	11 (3.3)	3 (3.9)	4 (3.0)		1 (1.3)	6 (4.5)		1 (2.1)	6 (3.7)	
Ped vs. Vehicle	4 (1.2)	1 (1.3)	2 (1.5)		1 (1.3)	2 (1.5)		1 (2.1)	2 (1.2)	
Fall>2m	69 (20.5)	13 (16.9)	30 (22.4)	0.20	18 (23.4)	25 (18.7)	0.80	13 (27.7)	30 (18.4)	0.80
Fall<2m	153 (45.4)	44 (57.1)	54 (40.3)		37 (48.1)	61 (45.5)		22 (46.8)	76 (46.6)	
Assault	14 (4.2)	1 (1.3)	4 (3.0)		1 (1.3)	3 (2.2)		1 (2.1)	3 (1.8)	
CPR	4 (1.2)	1 (1.3)	0		0	1 (0.7)		0	1 (0.6)	
Rapid deceleration	4 (1.2)	0	2 (1.5)		0	2 (1.5)		0	2 (1.2)	
**Pre-morbid state:**										
Respiratory	70 (20.8)	21 (27.3)	21 (15.7)	0.04*	21 (27.3)	20 (14.9)	0.03*	12 (25.5)	29 (17.8)	0.20
Cardiac	147 (43.6)	49 (63.6)	53 (39.6)	0.001*	47 (61.0)	54 (40.3)	0.004*	27 (57.4)	74 (45.4)	0.20
Neuro	50 (14.8)	20 (26.0)	9 (6.7)	<0.001*	19 (24.7)	10 (7.5)	0.001*	10 (21.3)	19 (11.7)	0.09
MSK	89 (26.4)	26 (33.8)	35 (26.1)	0.20	30 (39.0)	31 (23.1)	0.02*	16 (34.0)	45 (27.6)	0.40
Cancer Current	9 (2.7)	4 (5.2)	3 (2.2)	0.08	4 (5.2)	3 (2.2)	0.009*	3 (6.4)	4 (2.5)	0.40
Cancer Historical	26 (7.7)	12 (15.6)	10 (7.5)		14 (18.2)	8 (6.0)		4 (8.5)	18 (11.0)	
DM2	33 (9.8)	9 (11.7)	11 (8.2)	0.40	5 (6.5)	15 (11.2)	0.30	5 (10.6)	15 (9.2)	0.80
No of Comorbidities (Mean (±SD))	2.3 (2.2)	2.3 (0.3)	1.8 (0.2)	<0.001*	3.1 (2.2)	1.9 (2.0)	<0.001*	2.9 (2.5)	2.2 (2.1)	0.07
Chronic Pain	27 (8.0)	11 (14.3)	8 (6.0)	0.042*	13 (16.9)	6 (4.5)	0.003*	7 (14.9)	12 (7.4)	0.10
Regular Analgesic Use	61 (18.1)	23 (29.9)	15 (11.2)	0.001*	30 (39.0)	8 (6.0)	<0.001*	19 (40.4)	19 (11.7)	<0.001*

Smoking Status:									
Current smoker	73 (21.7)	11 (14.3)	21 (15.7)	0.90	10 (13.0)	22 (16.4)	0.07	11 (23.4)	21 (12.9)	0.05
Ex-Smoker	90 (26.7)	25 (32.5)	37 (27.6)		27 (35.1)	34 (25.4)		18 (38.3)	43 (26.4)	
Never smoked	122 (36.2)	30 (39.0)	52 (67.5)		23 (29.9)	59 (44.0)		14 (29.8)	68 (41.7)	
Smoking Status unknown	50 (14.8)	11 (14.3)	23 29.9)		17 (22.1)	17 (12.7)		4 (8.5)	30 (18.4)	
Pack years (Mean (±SD))	22.1 (18.4)	27.6 (14.2)	20.3 (18.8)	0.1	19.9 (14.3)	24.1 (19.2)	0.4	21.2 (14.8)	23.3 (18.9)	0.7
**Indicators of Injury Severity:**										
No. Rib Fractures (Mean (±SD))	5.2 (4.0)	5.3 (3.8)	5.3 (4.0)	0.90	5.4 (3.9)	5.3 (3.9)	0.70	5.6 (4.1)	5.2 (3.8)	0.60
Rib fracture Categories:										
0	19 (5.6)	5 (6.5)	8 (6.0)	0.30	6 (7.8)	7 (5.2)	0.80	1 (2.1)	12 (7.4)	0.70
1-4	160 (47.5)	39 (50.6)	58 (43.3)		33 (42.9)	63 (47.0)		25 (53.2)	71 (43.6)	
5-9	114 (33.8)	22 (28.6)	52 (38.8)		46 (59.7)	28 (20.9)		14 (29.8)	60 (36.8)	
10-15	29 (8.6)	9 (11.7)	9 (6.7)		7 (9.1)	11 (8.2)		5 (10.6)	13 (8.0)	
15+	15 (4.5)	2 (2.6)	7 (5.2)		3 (2.9)	6 (4.5)		2 (4.3)	7 (4.3)	
Other Indicators of Severity:										
Operative Rib Fixation	16 (4.7)	6 (7.8)	3 (2.2)	0.05*	2 (2.6)	7 (5.2)	0.40	3 (6.4)	6 (3.7)	0.40
First Rib fracture	31 (9.2)	6 (7.8)	13 (9.7)	0.60	10 (13.0)	9 (6.7)	0.10	7 (14.9)	12 (7.4)	0.10
Bilateral rib fractures	44 (13.1)	9 (11.7)	19 (14.2)	0.60	11 (14.3)	17 (12.7)	0.80	10 (21.3)	18 (11.0)	0.07
Scapular fracture	27 (8.0)	9 (11.7)	11 (8.2)	0.40	9 (11.7)	11 (8.2)	0.40	4 (8.5)	16 (9.8)	0.90
Clavicle fracture	29 (8.6)	8 (10.4)	14 (10.4)	0.50	8 (10.4)	14 (10.4)	0.50	4 (8.5)	18 (11.0)	0.08
Sternum Fracture	26 (7.7)	4 (5.2)	13 (9.7)	0.30	5 (6.5)	12 (9.0)	0.50	2 (4.3)	15 (9.2)	0.30
Manubrium Fracture	8 (2.4)	1 (1.3)	4 (3.0)	0.40	2 (2.6)	3 (2.2)	0.90	1 (2.1)	4 (2.5)	0.90
Thoracic Process #	31 (9.2)	6 (7.8)	12 (9.0)	0.80	11 (14.3)	7 (5.2)	0.02*	5 (10.6)	13 (8.0)	0.60
No. of Thoracic Process # (Mean (±SD))	3.3 (2.8)	2.2 (1.3)	2.8 (1.4)	0.40	2.8 (1.5)	2.3 (1.2)	0.50	2.5 (1.3)	2.7 (1.5)	0.80
Lung Contusion	76 (22.6)	14 (18.2)	32 (23.9)	0.30	9 (11.7)	36 (26.9)	0.009*	8 (17.0)	37 (22.7)	0.40
Pneumothorax	107 (31.8)	20 (26.0)	50 (37.3)	0.20	20 (26.0)	50 (37.3)	0.20	15 (31.9)	55 (33.7)	0.90
Haemothorax	80 (23.7)	15 (19.5)	38 (28.4)	0.20	14 (18.2)	38 (28.4)	0.90	5 (10.6)	47 (28.8)	0.01*
Massive Haemothorax	4 (1.2)	0	1 (0.7)	0.50	1 (1.3)	0	0.20	0	1 (0.6)	0.60
Haemopneumothorax	45 (13.4)	7 (9.1)	23 (17.2)	0.10	21 (27.3)	8 (6.0)	0.30	4 (8.5)	25 (15.3)	0.20
Radiological Flail	60 (17.8)	13 (16.9)	29 (21.6)	0.40	16 (20.8)	26 (19.4)	0.80	7 (14.9)	35 (21.5)	0.30
Extra-thoracic injuries	104 (30.9)	25 (32.5)	35 (26.1)	0.30	19 (24.7)	40 (29.9)	0.40	12 (25.5)	47 (28.8)	0.70

**Initial Vital Signs:**										
Respiratory Rate (Mean (±SD))	19.6 (±4.6)	20.6 (4.8)	18.9 (4.1)	0.01*	20.1 (5.1)	19.2 (4.0)	0.20	20.2 (4.5)	19.3 (4.4)	0.30
Oxygen Saturation (%) (Mean (±SD))	95.9 (4.7)	95.4 (4.4)	96.4 (3.6	0.10	95.6 (3.1)	96.3 (4.3)	0.20	95.8 (3.1)	96.1 (4.1)	0.70
Heart Rate (Mean (±SD))	81.5 (19.3)	81.8 (4.1)	79.4 (17.3)	0.40	82.5 (22.1)	79.0 (17.3)	0.20	83.9 (17.2)	79.3 (19.6)	0.10
Systolic Blood Pressure (Mean (±SD))	142.5 (23.0)	144.8 (23.9)	141.7 (22.3)	0.40	147.9 (25.8)	140.2 (20.7)	0.03*	148.7 (25.3)	141.3 (22.0)	0.09
Temperature (Mean (±SD))	36.6 (4.2)	37.4 (7.5)	36.5 (0.7)	0.30	37.4 (7.7)	36.5 (0.7)	0.20	36.4 (0.5)	37.0 (5.3)	0.20
**Chest Drain Management:**										
Intercostal Drain	67 (19.9)	14 (18.2)	33 (24.6)	0.30	14 (18.2)	32 (23.9)	0.30	9 (19.1)	37 (22.7)	0.60
Duration of Intercostal Drain (days) (Mean (±SD))	4.9 (3.2)	4.6 (2.6)	5.0 (3.8)	0.70	6.5 (4.7)	4.4 (2.9)	0.20	4.7 (1.4)	5.0 (3.8)	0.70
**Admission Level of Care (1-3):**										
Level 1	252 (74.8)	59 (76.6)	106 (79.1)	0.10	60 (77.9)	104 (77.6)	0.50	35 (74.5)	129 (79.1)	0.40
Level 2	65 (19.3)	13 (16.9)	26 (19.4)		13 (16.9)	26 (19.4)		9 (19.1)	30 (18.4)	
Level 3	18 (5.3)	5 (6.5)	2 (1.5)		4 (5.2)	3 (2.2)		3 (6.4)	4 (2.5)	
**Initial Emergency Department analgesic management:**										
None	28 (8.3)	6 (7.8)	14 (10.4)	0.80	6 (7.8)	14 (10.4)	0.30	2 (4.3)	18 (11.0)	0.20
Opioid	207 (61.4)	47 (61.0)	79 (59.0)		42 (54.5)	83 (61.9)		27 (57.4)	98 (60.1)	
Non-opioid	102 (30.3)	24 (31.2)	41 (30.6)		29 (37.7)	36 (26.9)		18 (38.3)	47 (28.8)	
**Inpatient Analgesic management:**										
Intermittent analgesia only	179 (53.1)	37 (48.1)	77 (57.5)	0.20	37 (48.1)	76 (56.7)	0.20	22 (46.8)	91 (55.8)	0.30
Regional Analgesia	106 (31.5)	29 (37.7)	41 (30.6)	0.30	31 (40.3)	39 (29.1)	0.10	20 (42.6)	50 (30.7)	0.10
Thoracic Epidural	50 (14.8)	13 (16.9)	23 (17.2)	0.90	14 (18.2)	22 (16.4)	0.80	8 (17.0)	28 (17.2)	1.0
Time to commence TE (hrs) (Mean (±SD))	27.7 (53.0)	23.2 (26.5)	37.2 (76.9)	0.50	53.5 (92.6)	18.3 (26.0)	0.10	15.1 (14.9)	36.9 (69.8)	0.20
Paravertebral block	16 (4.7)	5 (6.5)	5 (3.7)	0.40	8 (10.4)	2 (1.5)	0.004*	4 (8.5)	6 (3.7)	0.20
Time to commence PVB (hrs) (Mean (±SD))	20.4 (17.3)	11.8 (8.8)	29.3 (27.7)	0.40	12.5 (8.6)	-	-	-	-	-
Intercostal Nerve Block	4 (1.2)	3 (3.9)	0	0.02	1 (1.3)	2 (1.5)	0.90	1 (2.1)	2 (1.2)	0.50
Time to commence Intercostal Nerve Block (hrs) (Mean (±SD))	65.3 (57.6)	39.0 (28.9)	-	-	-	-	-	-	-	-
Serratus Plane Block	22 (6.5)	4 (5.2)	14 (10.4)	0.5	4 (5.2)	10 (7.5)	0.50	2 (4.3)	12 (7.4)	0.70

Time to commence SPB (hrs) (Mean (±SD))	17.6 (19.5)	34.8 (36.9)	13.6 (8.5)	0.10	17.6 (6.4)	20.4 (25.5)	0.80	14.3 (2.5)	20.5 (23.2)	0.40
Erector Spinae Block	20 (5.9)	6 (7.8)	4 (3.0)	0.10	7 (9.1)	3 (2.2)	0.03*	5 (10.6)	5 (3.1)	0.03*
Time to commence Erector Spinae Block (hrs) (Mean (±SD))	30.4 (19.8)	25.8 (29.5)	29.5 (27.3)	1.0	27.0 (22.5)	25.8 (-)	1.0	31.0 (32.0)	24.4 (24.7)	0.60
Combined Region Analgesia with Patient Controlled Analgesic	43 (12.8)	11 (14.3)	14 (10.4)	0.40	10 (13.0)	15 (11.2)	0.70	6 (12.8)	19 (11.7)	0.80
Patient Controlled Analgesic only	93 (27.6)	23 (29.9)	29 (21.6)	0.20	20 (26.0)	32 (23.9)	0.80	12 (25.5)	40 (24.5)	0.90
Time to commence PCA (hrs) (Mean (±SD))	16.2 (23.9)	12.4 (10.9)	12.7 (12.4)	0.90	12.8 (12.2)	12.5 (11.6)	0.90	14.1 (16.3)	12.1 (10.0)	0.70
**Other Analgesics:**										
Gabapentin	37 (11.0)	11 (14.3)	11 (8.2)	0.20	11 (14.3)	11 (8.2)	0.20	9 (19.1)	13 (8.0)	0.03*
Pregabalin	15 (4.5)	5 (6.5)	4 (3.0)	0.20	7 (9.1)	2 (1.5)	0.009*	6 (12.8)	3 (1.8)	0.001*
Amitriptyline	16 (4.7)	7 (9.1)	3 (2.2)	0.02*	7 (9.1)	3 (2.2)	0.03*	5 (10.6)	5 (3.1)	0.03*
Transdermal Lignocaine	113 (33.5)	28 (36.4)	43 (32.1)	0.50	27 (35.1)	44 (32.8)	0.80	12 (25.5)	59 (36.2)	0.20
**Discharge Analgesia:**										
Opioid	220 (65.3)	51 (66.2)	95 (70.9)	0.48	55 (71.4)	90 (67.2)	0.60	33 (70.2)	112 (68.7)	0.90
Neuropathic Pain Agent	41 (12.2)	14 (18.2)	11 (8.2)	0.03*	12 (15.6)	13 (9.7)	0.20	10 (21.3)	15 (9.2)	0.02*
NSAIDS	88 (26.1)	13 (16.9)	37 (27.6)	0.08	19 (24.7)	31 (23.1)	0.80	14 (29.8)	36 (22.1)	0.30
Transdermal Lignocaine	63 (18.7)	15 (19.5)	24 (17.9)	0.80	16 (20.8)	23 (17.2)	0.50	8 (17.0)	31 (19.0)	0.80
No discharge analgesics prescribed	34 (10.1)	7 (9.1)	9 (6.7)	0.50	6 (7.8)	10 (7.5)	0.90	5 (10.6)	11 (6.7)	0.40
Number of discharge analgesic agents (Mean (±SD))	2.1 (1.3)	2.1 (1.3)	2.1 (1.2)	0.80	2.2 (1.3)	2.1 (1.2)	0.70	2.3 (1.4)	2.1 (1.2)	0.30
**Complications:**	111 (32.9)	34 (44.2)	33 (24.6)	0.003*	25 (32.5)	42 (31.3)	0.90	18 (38.3)	49 (30.1)	0.30
Type 1 Respiratory Failure	18 (5.4)	2 (2.6)	4 (3.0)	0.90	2 (2.6)	4 (3.0)	0.90	2 (4.3)	4 (2.5)	0.60
Type 2 Respiratory Failure	10 (3.0)	5 (6.5)	1 (0.7)	0.02*	1 (1.3)	5 (3.7)	0.30	4 (8.5)	2 (1.2)	0.02*
Pulmonary Embolism	4 (1.2)	0	1 (0.7)	0.50	0	1 (0.7)	0.50	0	1 (0.6)	1.0
Pneumonia	42 (12.5)	13 (16.9)	12 (9.0)	0.09	11 (14.3)	14 (10.4)	0.40	8 (17.0)	17 (10.4)	0.20
Ventilator Associated Pneumonia	5 (1.5)	3 (3.9)	1 (0.7)	0.10	2 (2.6)	2 (1.5)	0.60	2 (4.3)	2 (1.2)	0.20
ARDS	2 (0.6)	1 (1.3)	0	0.20	0	1 (0.7)	0.50	0	1 (0.6)	1.0

LRTI	58 (17.2)	16 (20.8)	19 (14.2)	0.20	11 (14.3)	24 (17.9)	0.50	7 (14.9)	28 (17.2)	0.70
Treated with Abx	110 (32.6)	29 (37.7)	38 (28.4)	0.20	22 (28.6)	45 (33.6)	0.40	15 (31.9)	52 (31.9)	1.0
**Post Admission Ventilation:**	26 (7.7)	9 (11.7)	5 (3.7)	0.03*	6 (7.8)	8 (6.0)	0.60	5 (10.6)	9 (5.5)	0.20
CPAP	4 (1.2)	2 (2.6)	0	0.06	0	2 (1.5)	0.50	0	2 (1.2)	1.0
BiPAP	1 (0.3)	1 (1.3)	0	0.20	0	1 (0.7)	1.0	0	1 (0.6)	1.0
Invasive Ventilation	17 (5.0)	7 (9.1)	2 (1.5)	0.01*	3 (3.9)	6 (4.5)	1.0	2 (4.3)	7 (4.3)	1.0
High Flow nasal Oxygen	19 (5.6)	6 (7.8)	5 (3.7)	0.20	5 (6.5)	6 (4.5)	0.50	4 (8.5)	7 (4.3)	0.30
Moved to Critical Care	29 (8.6)	10 (13.0)	7 (5.2)	0.05*	6 (7.8)	11 (8.2)	0.90	5 (10.6)	12 (7.3)	0.50
ICU Length of Stay (Mean (±SD))	7.6 (7.4)	9.42 (7.5)	6.42 (5.8)	0.20	7.6 (5.4)	7.8 (7.3)	0.90	9.4 (7.2)	7.4 (6.6)	0.50
Hospital Length of Stay (Mean (±SD))	8.9 (9.5)	11.7 (10.2)	6.9 (5.7)	<0.001*	10.4 (9.3)	7.7 (7.0)	0.03*	9.9 (7.2)	8.3 (8.2)	0.2
**Discharge outcome:**										
Home	306 (90.8)	68 (88.3)	128 (95.5)	0.01*	70 (90.9)	125 (93.3)	0.5	43 (91.5)	152 (93.3)	0.3
Rehabilitation Bed	9 (2.7)	6 (7.8)	0		3 (3.9)	3 (2.2)		3 (6.4)	3 (1.8)	
Repatriation to local hospital	15 (4.5)	3 (3.9)	5 (3.7)		3 (3.9)	5 (3.7)		1 (2.1)	7 (4.3)	
Long term care	4 (1.2)	0	1 (0.7)		1 (1.3)	0		0	1 (0.6)	

Supplementary Table S2: Comparison of characteristics of responders and non-responders

Significance denoted by *<br>Result= n=(%) unless otherwise indicated	Responded at 6 months (n=211)	Non-respondent at 6 months (n=126)	P-value
Age (Mean (SD))	63.8 (16.0)	59.0 (17.0)	0.01*
Male (%)	146 (69.5)	85 (66.9)	0.62
Injury Severity Score (Mean (SD))	11.3 (7.2)	11.1 (7.6)	0.86
Battle/StUMBle Score (Mean (SD))	22.5 (11.2)	21.5 (13.3)	0.48
**Pre-morbid state:**			
Respiratory	42 (20.0)	28 (22.0)	0.65
Cardiac	102 (48.6)	45 (35.4)	0.02*
Neuro	28 (13.3)	22 (17.3)	0.32
MSK	60 (28.6)	29 (22.8)	0.25
Cancer Current	7 (3.3)	2 (1.6)	0.03*
Cancer Historical	22 (10.5)	4 (3.1)	
DM2	20 (9.5)	13 (10.2)	0.81
No of Comorbidities (Mean (SD))	2.3 (2.2)	2.3 (2.2)	0.86
Chronic Pain	19 (9.0)	8 (6.3)	0.37
Regular Analgesic Use	38 (18.1)	23 (18.1)	1.0
**Indicators or Injury severity:**			
No. Rib Fractures (Mean (SD))	5.3 (3.9)	5.0 (4.2)	0.56
Operative Rib Fixation	9 (4.3)	7 (5.5)	0.61
First Rib fracture	19 (9.0)	12 (9.4)	0.90
Bilateral rib fractures	28 (13.3)	16 (12.6)	0.85
**Admission Level of Care (1-3):**			
Level 1	164 (78.1)	88 (69.3)	0.07
Level 2	39 (18.6)	26 (20.5)	
Level 3	7 (3.3)	11 (8.7)	
**Complications:**	66 (31.4)	45 (35.4)	0.39
Type 1 Respiratory Failure	6 (2.9)	12 (9.4)	0.008
Type 2 Respiratory Failure	6 (2.9)	4 (3.1)	0.86
Pulmonary Embolism	1 (0.5)	3 (2.4)	0.12
Pneumonia	25 (11.9)	17 (13.4)	0.65
Ventilator Associated Pneumonia	4 (1.9)	1 (0.8)	0.42
ARDS	1 (0.5)	1 (0.8)	0.71
LRTI	34 (16.2)	24 (18.9)	0.55
Treated with Abx	66 (31.4)	44 (34.6)	0.48
Post Admission Ventilation:	14 (6.7)	12 (9.4)	0.33
ICU Length of Stay (Mean (SD))	7.7 (6.7)	7.4 (8.2)	0.84
Hospital Length of Stay (Mean (SD))	8.6 (8.0)	9.5 (11.7)	0.48
**Discharge outcome:**			
Home	195 (92.9)	111 (87.4)	0.47
Rehabilitation Bed	6 (2.9)	3 (2.4)	
Repatriation to local hospital	8 (3.8)	7 (5.5)	
Long term care	1 (0.5)	3 (2.4)	
**Health Related Quality of Life:**			
Pre-injury SF-12 PCS (Mean (SD))	46.8 (10.4)	45.9 (12.0)	0.44
Pre-injury SF-12 MCS (Mean (SD))	51.2 (9.5)	47.1 (12.1)	0.001*
Discharge SF-12 PCS (Mean (SD))	26.9 (4.4)	27.7 (9.0)	0.43
Discharge SF-12 MCS (Mean (SD))	45.8 (11.6)	41.9 (11.9)	0.004*
1-month SF-12 PCS (Mean (SD))	31.2 (7.67)	33.8 (8.27)	0.09
1-month SF-12 MCS (Mean (SD))	44.8 (11.6)	39.5 (11.4)	0.02*
3-month SF-12 PCS (Mean (SD))	40.2 (11.1)	35.8 (4.03)	0.19
3-month SF-12 MCS (Mean (SD))	48.3 (11.5)	45.6 (9.51)	0.68

Supplementary File: Table S3: Extended Outcomes of interest measured at 6-months after discharge (The total sample)

Time-point	PROM	Min	Max	Mean (±SD)	Dif. from Pop. Norms	Pop Norm
Pre-Injury	SF-12 Physical Component Score	13.1	64.9	46.5 (11.0)	-3.5	50.0 (SD 1.0)
	SF-12 Mental Component Score	13.5	67.2	49.7 (10.7)	-0.3	50.0 (SD 1.0)
	EQ Index Score	-0.3	1.0	0.7 (0.3)	-0.2	0.9 (SE 0.004)
	EQ VAS Score	10.0	100.0	74.9 (19.7)	-7.9	82.8 (SE 0.4)
Discharge	SF-12 Physical Component Score	10.9	55.5	27.2 (8.6)	-22.8	50.0 (SD 1.0)
	SF-12 Mental Component Score	15.9	68.9	44.4 (11.8)	-5.6	50.0 (SD 1.0)
	EQ Index Score	-0.5	1.0	0.3 (0.3)	-0.6	0.9 (SE 0.004)
	EQ VAS Score	0	99.0	47.4 (23.8)	35.4	82.8 (SE 0.4)
	BPI Pain Severity Score	0	10.0	5.0 (2.1)	-	-
	BPI Pain Interference Score	0	10.0	5.7 (2.3)	-	-
	BPI Analgesic Effectiveness	0	100.0	65.3 (24.8)	-	-
	PainDetect Score	-1	29	10.3 (6.0)	-	-
	Prop. reporting Thoracic Pain (%)	-	-	283 (88.4)	-	-
	Mean No. affected body regions (21)	0	16.0	2.5 (2.1)	-	-
1-month	SF-12 Physical Component Score	14.8	55.7	31.6 (7.8)	-18.4	50.0 (SD 1.0)
	SF-12 Mental Component Score	18.4	66.3	43.9 (11.8)	-6.1	50.0 (SD 1.0)
	EQ Index Score	-0.5	1.0	0.5 (0.3)	-0.4	0.9 (SE 0.004)
	EQ VAS Score	5.0	100.0	60.0 (22.7)	-22.8	82.8 (SE 0.4)
	BPI Pain Severity Score	0	10.0	3.8 (2.2)	-	-
	BPI Pain Interference Score	0	10.0	4.4 (2.7)	-	-
	BPI Analgesic Effectiveness	0	100.0	48.4 (30.0)	-	-
	PainDetect Score	0	35.0	8.2 (7.5)	-	-
	Prop. reporting Thoracic Pain (%)	-	-	162 (77.1)	-	-
	Mean No. affected body regions (21)	0	12	3.0 (2.3)	-	-
3-months	SF-12 Physical Component Score	13.3	63.5	40.1 (11.0)	-9.9	50.0 (SD 1.0)
	SF-12 Mental Component Score	19.1	66.8	48.3 (11.4)	-1.7	50.0 (SD 1.0)
	EQ Index Score	-0.5	1.0	0.7 (0.3)	-0.2	0.9 (SE 0.004)
	EQ VAS Score	0	100.0	68.9 (24.4)	-13.9	82.8 (SE 0.4)
	BPI Pain Severity Score	0	9.8	2.9 (2.6)	-	-
	BPI Pain Interference Score	0	9.9	2.6 (2.7)	-	-
	BPI Analgesic Effectiveness	0	100.0	46.5 (35.8)	-	-
	PainDetect Score	0	32.0	8.2 (7.5)	-	-
	Prop. reporting Thoracic Pain (%)	-	-	79 (54.9)	-	-
	Mean No. affected body regions (21)	0	20.0	2.6 (2.7)	-	-
6-months	SF-12 Physical Component Score	12.3	62.1	41.7 (11.5)	-8.3	50.0 (SD 1.0)
	SF-12 Mental Component Score	19.1	65.8	47.8 (11.8)	-2.2	50.0 (SD 1.0)
	EQ Index Score	-0.3	1.0	0.7 (0.3)	-0.2	0.9 (SE 0.004)
	EQ VAS Score	0	100.0	69.2 (26.5)	-13.6	82.8 (SE 0.4)
	BPI Pain Severity Score	0	10.0	2.7 (2.7)	-	-
	BPI Pain Interference Score	0	10.0	2.8 (2.9)	-	-
	BPI Analgesic Effectiveness	0	100.0	49.2 (33.4)	-	-
	PainDetect Score	-1	33	7.5 (7.4)	-	-
	Prop. reporting Thoracic Pain (%)	-	-	99 (46.9)	-	--
	Mean No. affected body regions (21)	0	21	2.6 (3.2)	-	

Supplemental File: Table S4: Extended Outcomes of Interest Data/Candidate predictor variables at six-months after discharge

Mean (±SD)	Outcome Data:	PCS <35 (n=76)	PCS>35 (n=135)	p-value	PSS ≥3.5 (n=77)	PSS <3.5 (n=134)	p-value	PDQ >12 (n=47)	PDQ <12 (n=164)	p-value
Pre-Injury	PCS	40.0 (11.0)	50.8 (7.7)	<0.001*	41.9 (11.4)	49.6 (8.6)	<0.001*	41.7 (11.5)	48.3 (9.6)	<0.001*
	MCS	48.2 (11.7)	53.1 (7.6)	<0.001*	49.1 (10.9)	52.7 (8.4)	0.009*	48.2 (10.6)	52.3 (9.1)	0.009*
	Prop. PCS<20 (n=3) (%)	3 (100)	0	0.022*	3 (100)	0	0.021*	1 (2.2)	2 (1.2)	0.63
	Prop. PCS <30 (n= 22) (%)	17 (77.3)	5 (22.7)	<0.001*	17 (22.4)	5 (3.8)	<0.001*	11 (23.9)	11 (6.7)	0.001*
	Prop. PCS <35 (n=37) (%)	30 (81.1)	7 (18.9)	<0.001*	23 (30.3)	14 (10.5)	<0.001*	16 (34.8)	21 (12.9)	0.001*
Discharge	PCS	24.3 (7.4)	28.4 (8.6)	0.001*	25.1 (8.3)	27.9 (8.3)	0.02*	25.1 (8.4)	27.5 (8.3)	0.09
	MCS	43.5 (11.8)	47.0 (11.5)	0.04*	46.1 (11.5)	45.5 (11.9)	0.76	42.6 (11.7)	46.7 (11.6)	0.04*
	Prop. PCS<20 (n=54) (%)	28 (37.3))	26 (20.0)	0.007*	27 (36.5)	27 (20.8)	0.014*	19 (40.4)	35 (22.3)	0.013*
	Prop. PCS <30 (n=150) (%)	64 (85.3))	86 (66.2)	0.003*	59 (79.7)	90 (69.2)	0.10	39 (83.0)	110 (70.1)	0.08
	Prop. PCS <35 (n=154) (%)	65 (56.7)	89 (68.5)	0.004*	61 (82.4)	93 (71.5)	0.082	36 (76.6)	118 (75.2)	0.84
	BPI Worst Pain	7.4 (2.0)	7.1 (2.2)	0.30	7.3 (2.0)	7.1 (2.3)	0.40	7.5 (1.8)	7.1 (2.2)	0.20
	BPI Least Pain	3.5 (2.7)	3.2 (2.6)	0.40	3.7 (2.7)	3.0 (2.6)	0.06	3.9 (2.6)	3.0 (2.6)	0.04*
	BPI Average Pain	5.4 (2.1)	5.1 (2.0)	0.30	5.4 (2.0)	5.1 (2.1)	0.40	6.7 (2.0)	5.1 (2.1)	0.08
	BPI Current Pain	4.7 (2.8)	3.8 (2.6)	0.01*	4.7 (2.8)	3.8 (2.5)	0.02*	5.3 (2.7)	3.8 (2.6)	0.001*
	BPI Pain Severity Score	5.3 (2.1)	4.8 (2.0)	0.10	5.3 (2.0)	4.7 (2.0)	0.06	5.6 (2.0)	4.7 (2.0)	0.009*
	Prop. PSS >3.5 (n=157) (%)	60 (81.1)	97 (74.6)	0.30	59 (80.8)	97 (74.6)	0.30	40 (87.0)	116 (73.9)	0.07
	Analgesic Effectiveness	57.8 (27.4)	68.4 (22.8)	0.003*	63.3 (26.2)	65.8 (23.8)	0.50	64.3 (23.3)	65.1 (25.1)	0.90
	General Activities	7.8 (2.6)	6.6 (3.0)	0.006*	7.3 (2.7)	6.9 (3.0)	0.30	7.4 (2.8)	6.9 (3.0)	0.30
	Mood	5.5 (3.2)	4.0 (3.2)	0.002*	4.6 (3.0)	4.4 (3.5)	0.70	5.3 (3.2)	4.2 (3.3)	0.05
	Walking	6.9 (3.0)	4.7 (3.1)	<0.001*	6.4 (3.2)	5.0 (3.2)	0.004*	6.8 (3.0)	5.1 (3.2)	0.001*
	Normal Work	8.6 (2.3)	7.4 (3.3)	0.005*	8.4 (2.5)	7.5 (3.3)	0.04*	8.3 (2.4)	7.7 (3.2)	0.20
	Relations	3.6 (3.7)	2.5 (2.9)	0.02*	3.1 (3.3)	2.7 (3.2)	0.50	3.9 (3.5)	2.5 (3.1)	0.02*
	Sleep	6.0 (3.3)	2.3 (3.3)	0.20	5.6 (3.5)	5.5 (3.2)	0.70	6.4 (3.0)	5.3 (3.4)	0.03*
	Enjoyment	7.0 (3.1)	3.0 (3.5)	0.04*	6.2 (3.4)	6.4 (3.4)	0.70	3.9 (3.0)	6.2 (3.5)	0.20
	BPI Pain Interference Score	6.5 (2.0)	5.2 (2.4)	<0.001*	5.9 (2.0)	5.5 (2.4)	0.18	6.4 (2.0)	5.4 (2.3)	0.004*
	PDQ: Low Risk (n=143) (%)	48 (64.9)	95 (73.1)	0.42	47 (63.5)	96 (74.4)	0.08	24 (52.2)	119 (75.8)	0.006*
	PDQ: Moderate Risk (n=44) (%)	18 (24.3)	26 (20.0)		22 (29.7)	21 (16.3)		17 (37.0)	26 (16.6)	
	PDQ: High Risk (n=17) (%)	8 (10.8)	9 (6.9)		5 (6.8)	12 (9.3)		5 (10.9)	12 (7.6)	
1-month	PCS	27.0 (6.0)	33.5 (7.5)	<0.001*	27.7 (6.0)	32.9 (7.8)	<0.001*	27.8 (5.8)	32.1 (7.9)	<0.001*
	MCS	40.8 (12.2)	46.8 (11.2)	0.001*	4.07 (12.7)	46.7 (10.9)	0.001*	35.6 (12.3)	47.0 (10.6)	<0.001*

	Prop. PCS<20 (n=10) (%)	7 (10.9)	3 (2.7)	0.022*	6 (10.0)	4 (3.4)	0.073	3 (8.1)	7 (5.0)	0.47
	Prop. PCS <30 (n=95) (%)	50 (78.1)	45 (39.8)	<0.001*	42 (70.0)	53 (45.3)	0.002*	26 (70.3)	69 (49.3)	0.023*
	Prop. PCS <35 (n=130) (%)	60 (93.8)	70 (61.9)	<0.001*	52 (86.7)	78 (66.7)	0.004*	33 (89.2)	97 (69.3)	0.015*
	BPI Worst Pain	5.7 (2.5)	4.5 (2.3)	0.002*	6.1 (2.2)	4.4 (2.3)	<0.001*	6.6 (2.3)	4.5 (2.3)	<0.001*
	BPI Least Pain	3.6 (2.4)	2.5 (2.2)	0.003*	4.4 (2.3)	2.2 (2.0)	<0.001*	4.5 (2.3)	2.5 (2.1)	<0.001*
	BPI Average Pain	4.8 (2.3)	3.5 (2.0)	<0.001*	5.3 (2.0)	3.2 (2.0)	<0.001*	5.7 (2.2)	3.5 (2.0)	<0.001*
	BPI Current Pain	4.5 (2.6)	2.5 (2.2)	<0.001*	5.0 (2.4)	2.4 (2.1)	<0.001*	5.4 (2.6)	2.7 (2.2)	<0.001*
	BPI Pain Severity Score	4.7 (2.3)	3.2 (1.9)	<0.001*	5.2 (2.0)	3.0 (1.9)	<0.001*	5.5 (2.2)	3.3 (1.9)	<0.001*
	Prop. PSS >3.5 (n=101) (%)	45 (71.4)	56 (49.6)	0.005*	49 (83.1)	52 (44.4)	<0.001*	31 (86.1)	70 (50.0)	<0.001*
	Analgesic Effectiveness	50.0 (24.8)	48.2 (32.7)	0.70	49.7 (23.6)	48.4 (33.1)	0.8	50.0 (27.2)	48.6 (30.8)	0.80
	General Activities	6.1 (2.9)	3.9 (2.6)	<0.001*	6.2 (2.8)	3.9 (2.7)	<0.001*	6.7 (2.8)	4.2 (2.7)	<0.001*
	Mood	4.5 (3.2)	3.1 (2.9)	0.006*	5.0 (3.0)	2.9 (2.9)	<0.001*	5.8 (3.2)	3.0 (2.8)	<0.001*
	Walking	5.8 (3.1)	3.1 (3.0)	<0.001*	5.9 (3.0)	3.1 (3.1)	<0.001*	6.1 (3.2)	3.5 (3.1)	<0.001*
	Normal Work	6.8 (3.1)	4.4 (3.1)	<0.001*	6.8 (2.9)	4.5 (3.2)	<0.001*	7.4 (2.7)	4.7 (3.2)	<0.001*
	Relations	4.0 (3.5)	2.4 (3.0)	0.001*	4.2 (3.3)	2.3 (3.1)	<0.001*	5.3 (3.1)	2.4 (3.0)	<0.001*
	Sleep	5.9 (3.2)	4.8 (3.1)	0.04*	6.7 (2.8)	4.5 (3.1)	<0.001*	7.6 (2.5)	4.6 (3.0)	<0.001*
	Enjoyment	6.2 (3.3)	4.1 (3.1)	<0.001*	6.2 (3.1)	4.2 (3.3	<0.001*	7.0 (3.0)	4.3 (3.2)	<0.001*
	BPI Pain Interference Score	5.6 (2.6)	3.7 (2.5)	<0.001*	5.8 (2.4)	3.6 (2.5)	<0.001*	6.6 (2.4)	3.8 (2.4)	<0.001*
	PDQ: Low Risk (n=122) (%)	39 (61.9)	83 (73.5)	0.10	34 (57.6)	88 (75.2)	0.005*	14 (38.9)	108 (77.1)	<0.001*
	PDQ: Moderate Risk (n=36) (%)	14 (22.2)	22 (19.5)		13 (22.0)	23 (19.7)		9 (25.0)	27 (19.3)	
	PDQ: High Risk (n=18) (%)	10 (15.9)	8 (7.1)		12 (20.3)	6 (5.1)		13 (36.1)	5 (3.6)	
3-months	PCS	31.4 (8.8)	44.4 (9.5)	<0.001*	32.4 (9.1)	43.9 (10.0)	<0.001*	32.9 (8.8)	42.1 (10.8)	<0.001*
	MCS	43.1 (12.9)	50.9 (9.7)	<0.001*	43.1 (11.8)	51.0 (10.4)	<0.001*	40.0 (12.0)	50.7 (10.2)	<0.001*
	Prop. PCS<20 (n=4) (%)	3 (6.4)	1 (1.1)	0.07	3 (6.4)	1 (1.1)	0.071	1 (3.2)	3 (2.7)	0.88
	Prop. PCS <30 (n=32) (%)	24 (51.1)	8 (8.4)	<0.001*	23 (48.9)	9 (9.5)	<0.001*	14 (45.2)	18 (16.2)	0.001*
	Prop. PCS <35 (n=56) (%)	35 (74.5)	21 (22.1)	<0.001*	34 (74.5)	22 (23.2)	<0.001*	21 (67.7)	35 (31.5)	<0.001*
	BPI Worst Pain	5.6 (2.8)	2.9 (2.5)	<0.001*	6.2 (2.2)	2.6 (2.4)	<0.001*	5.9 (2.2)	3.2 (2.8)	<0.001*
	BPI Least Pain	3.4 (2.9)	1.7 (2.3)	0.001*	4.4 (2.7)	1.2 (1.9)	<0.001*	4.0 (2.7)	1.8 (2.4)	<0.001*
	BPI Average Pain	4.6 (2.8)	2.3 (2.3)	<0.001*	5.3 (2.4)	2.0 (2.0)	<0.001*	5.0 (2.4)	2.5 (2.5)	<0.001*
	BPI Current Pain	4.1 (3.2)	1.8 (2.3)	<0.001*	4.7 (2.7)	1.5 (2.2)	<0.001*	4.4 (2.8)	2.0 (2.7)	<0.001*
	BPI Pain Severity Score	4.4 (2.7)	2.2 (2.2)	<0.001*	5.1 (2.2)	1.8 (2.0)	<0.001*	4.8 (2.2)	2.4 (2.4)	<0.001*
	Prop. PSS >3.5 (n=53) (%)	27 (58.7)	26 (27.4)	<0.001*	35 (74.5)	18 (19.1)	<0.001*	23 (74.2)	30 (27.3)	<0.001*
	Analgesic Effectiveness	40.0 (32.1)	50.1 (37.6)	0.10	48.5 (29.6)	44.5 (40.0)	0.60	48.7 (31.3)	45.3 (37.5)	0.70
	General Activities	4.8 (3.5)	2.1 (2.6)	<0.001*	5.7 (3.0)	1.6 (2.2)	<0.001*	5.6 (3.0)	2.2 (2.8)	<0.001*

	Mood	4.0 (3.5)	1.5 (2.3)	<0.001*	4.6 (3.1)	1.2 (2.2)	<0.001*	5.1 (3.1)	1.6 (2.5)	<0.001*
	Walking	4.9 (3.8)	1.4 (2.4)	<0.001*	5.0 (3.4)	1.4 (2.6)	<0.001*	4.4 (3.3)	2.1 (3.2)	0.001*
	Normal Work	5.4 (3.7)	2.0 (2.7)	<0.001*	5.7 (3.2)	1.8 (2.7)	<0.001*	5.6 (3.2)	2.5 (3.2)	<0.001*
	Relations	2.5 (3.0)	1.1 (2.1)	0.001*	3.2 (3.0)	0.8 (1.7)	<0.001*	3.8 (3.2)	1.0 (1.8)	<0.001*
	Sleep	4.2 (3.4)	2.2 (2.7)	<0.001*	4.9 (3.1)	1.9 (2.5)	<0.001*	5.1 (3.2)	2.3 (2.7)	<0.001*
	Enjoyment	5.0 (3.7)	2.0 (2.6)	<0.001*	5.7 (3.4)	1.7 (2.4)	<0.001*	5.3 (3.6)	2.4 (3.0)	<0.001*
	BPI Pain Interference Score	4.4 (3.1)	1.8 (2.1)	<0.001*	5.0 (2.6)	1.5 (2.0)	<0.001*	5.0 (2.6)	2.0 (2.4)	<0.001*
	PDQ: Low Risk (n=107) (%)	28 (60.9)	79 (83.2)	0.012*	23 (48.9)	84 (89.4)	<0.001*	9 (29.0)	98 (89.1)	<0.001*
	PDQ: Moderate Risk (n=15) (%)	7 (15.2)	8 (8.4)		9 (19.1)	6 (6.4)		9 (29.0)	6 (5.5)	
	PDQ: High Risk (n=19) (%)	11 (23.9)	8 (8.4)		15 (31.9)	4 (4.3)		13 (41.9)	6 (5.5)	
6-months	PCS	29.2 (5.1)	48.9 (7.2)	<0.001*	32.9 (8.0)	46.8 (10.1)	<0.001*	32.4 (8.5)	44.4 (10.9)	<0.001*
	MCS	42.3 (13.2)	51.0 (9.7)	<0.001*	39.4 (11.6)	52.7 (8.9)	<0.001*	36.4 (11.8)	51.1 (9.6)	<0.001*
	Prop. PCS<20 (n=4) (%)	-	-	-	3 (4.0)	1 (0.7)	0.1	3 (6.4)	1 (0.6)	0.01*
	Prop. PCS <30 (n=42) (%)	-	-	-	30 (39.0)	12 (9.0)	<0.001*	20 (42.6)	22 (13.4)	<0.001*
	Prop. PCS <35 (n=76) (%)	-	-	-	51 (66.2)	25 (18.8)	<0.001*	32 (68.1)	44 (26.8)	<0.001*
	BPI Worst Pain	5.8 (4.5)	2.1 (2.4)	<0.001*	6.5 (1.8)	1.6 (2.0)	<0.001*	6.3 (2.2)	2.6 (2.7)	<0.001*
	BPI Least Pain	4.1 (3.0)	1.2 (2.0)	<0.001*	5.2 (2.4)	0.6 (0.9)	<0.001*	4.9 (2.8)	1.5 (2.2)	<0.001*
	BPI Average Pain	5.0 (2.6)	1.6 (2.0)	<0.001*	5.9 (1.8)	1.1 (1.3)	<0.001*	5.4 (2.4)	2.1 (2.4)	<0.001*
	BPI Current Pain	4.5 (3.0)	1.2 (2.0)	<0.001*	5.6 (2.2)	0.5 (0.9)	<0.001*	5.2 (2.7)	1.6 (2.4)	<0.001*
	BPI Pain Severity Score	4.8 (2.6)	1.5 (2.0)	<0.001*	5.8 (1.8)	0.9 (1.1)	<0.001*	5.4 (2.3)	1.9 (2.3)	<0.001*
	Prop. PSS >3.5 (n=77) (%)	51 (67.1)	26 (19.3)	<0.001*	-	-	-	37 (78.7)	40 (24.5)	<0.001*
	Analgesic Effectiveness	45.6 (28.6)	53.0 (37.6)	0.22	46.6 (26.5)	52.7 (41.0)	0.32	49.1 (24.7)	49.2 (37.8)	1.0
	General Activities	5.7 (3.0)	1.5 (2.2)	<0.001*	6.2 (2.2)	1.2 (2.1)	<0.001*	6.3 (2.7)	2.1 (2.7)	<0.001*
	Mood	4.5 (3.3)	1.3 (2.1)	<0.001*	5.0 (3.0)	1.0 (1.9)	<0.001*	6.1 (2.9)	1.4 (2.1)	<0.001*
	Walking	6.0 (3.5)	1.3 (2.2)	<0.001*	6.0 (3.1)	1.3 (2.4)	<0.001*	6.2 (3.4)	2.1 (2.9)	<0.001*
	Normal Work	6.0 (3.2)	1.6 (2.5)	<0.001*	6.4 (2.8)	1.3 (2.3)	<0.001*	6.5 (3.0)	2.3 (3.0)	<0.001*
	Relations	3.8 (3.4)	0.8 (1.8)	<0.001*	4.26 (3.2)	0.5 (1.4)	<0.001*	5.0 (3.1)	1.0 (2.0)	<0.001*
	Sleep	5.0 (3.3)	1.9 (2.6)	<0.001*	5.6 (2.8)	1.6 (2.4)	<0.001*	6.5 (2.6)	2.05 (2.6)	<0.001*
	Enjoyment	5.5 (3.2)	1.5 (2.4)	<0.001*	6.0 (2.7)	1.2 (2.2)	<0.001*	6.4 (3.2)	2.0 (2.6)	<0.001*
	BPI Pain Interference Score	5.2 (2.7)	1.4 (2.0)	<0.001*	5.6 (2.3)	1.2 (1.7)	<0.001*	6.1 (2.6)	1.8 (2.2)	<0.001*
	PDQ: Low Risk (n=) (%)	44 (57.9)	119 (88.8)	<0.001*	40 (51.9)	123 (92.5)	<0.001*	0	163 (100)	<0.001*
	PDQ: Moderate Risk (n=) (%)	17 (22.4)	11 (8.2)		20 (26.0)	8 (6.0)		28 (59.6)	0	
	PDQ: High Risk (n=) (%)	15 (19.7)	4 (3.0)		17 (22.1)	2 (1.5)		19 (40.4)	0	
