# Supplementary material for: Prospective observational study to examine health-related quality of life and develop models to predict long-term patient-reported outcomes 6 months after hospital discharge with blunt thoracic injuries
Source: BMJ Open. 2021 Jul 8;11(7):e049292. doi: 10.1136/bmjopen-2021-049292 (PMC8268921; doi:10.1136/bmjopen-2021-049292)
Supplement: Author's manuscript [file bmjopen-2021-049292.draft_revisions.pdf]

BMJ Open is committed to open peer review. As part of this commitment we make the peer review history of every article we publish publicly available.

When an article is published we post the peer reviewers' comments and the authors' responses online. We also post the versions of the paper that were used during peer review. These are the versions that the peer review comments apply to.

The versions of the paper that follow are the versions that were submitted during the peer review process. They are not the versions of record or the final published versions. They should not be cited or distributed as the published version of this manuscript.

BMJ Open is an open access journal and the full, final, typeset and author-corrected version of record of the manuscript is available on our site with no access controls, subscription charges or pay-per-view fees (http://bmjopen.bmj.com).

If you have any questions on BMJ Open's open peer review process please email info.bmjopen@bmj.com

BMJ Open

A prospective observational study to examine health related quality of life and develop models to predict long-term patient reported outcomes six-months after hospital discharge with blunt thoracic injuries.

Journal:	*BMJ Open*
Manuscript ID	bmjopen-2021-049292
Article Type:	Original research
Date Submitted by the Author:	25-Jan-2021
Complete List of Authors:	Baker, Edward; King's College London, Florence Nightingale Faculty of Nursing, Midwifery and Palliative Care; King's College Hospital NHS Foundation Trust, Emergency Department<br>Battle, Ceri; Swansea Bay University Health Board, Welsh Institute of Biomedical and Emergency Medicine Research<br>Banjeri, Abhishek; Buckingham Healthcare NHS Trust<br>Carlton, Edward; North Bristol NHS Trust, Emergency Department<br>Dixon, Christine; Surrey and Sussex Healthcare NHS Trust, Emergency Department<br>Ferry, Jennifer; Aneurin Bevan Health Board, Department of Anesthetics<br>Hopkins, Philip; King's College Hospital NHS Foundation Trust, Critical Care<br>Jones, Robert; Barnsley Hospital NHS Foundation Trust<br>Murrells, Trevor; King's College London, Florence Nightingale Faculty of Nursing, Midwifery and Palliative Care<br>Norton, Christine; King's College London, Florence Nightingale Faculty of Nursing & Midwifery<br>Patient, Lee; St George's Healthcare NHS Trust, Emergency Department<br>Rasheed, Ashraf; Aneurin Bevan Health Board, General Surgery<br>Skene, Imogen; UCL, Primary care and population health; Barts Health NHS Trust, Emergency Department<br>Tabner, Andrew; University Hospitals of Derby and Burton NHS Foundation Trust<br>Tunnicliff, Malcolm; King's College Hospital NHS Foundation Trust, Emergency Department<br>Xyrichis, Andreas; King's College London, Florence Nightingale Faculty of Nursing, Midwifery and Palliative Care<br>Young, Louise; Imperial College Healthcare NHS Trust, Emergency Department<br>Lee, Gerry; King's College London, Florence Nightingale Faculty of Nursing, Midwifery and Palliative Care
Keywords:	ACCIDENT & EMERGENCY MEDICINE, Risk management < HEALTH SERVICES ADMINISTRATION & MANAGEMENT, Trauma management < ORTHOPAEDIC & TRAUMA SURGERY, PAIN MANAGEMENT, Cardiothoracic surgery < SURGERY

I, the Submitting Author has the right to grant and does grant on behalf of all authors of the Work (as defined in the below author licence), an exclusive licence and/or a non-exclusive licence for contributions from authors who are: i) UK Crown employees; ii) where BMJ has agreed a CC-BY licence shall apply, and/or iii) in accordance with the terms applicable for US Federal Government officers or employees acting as part of their official duties; on a worldwide, perpetual, irrevocable, royalty-free basis to BMJ Publishing Group Ltd ("BMJ") its licensees and where the relevant Journal is co-owned by BMJ to the co-owners of the Journal, to publish the Work in this journal and any other BMJ products and to exploit all rights, as set out in our licence.

The Submitting Author accepts and understands that any supply made under these terms is made by BMJ to the Submitting Author unless you are acting as an employee on behalf of your employer or a postgraduate student of an affiliated institution which is paying any applicable article publishing charge ("APC") for Open Access articles. Where the Submitting Author wishes to make the Work available on an Open Access basis (and intends to pay the relevant APC), the terms of reuse of such Open Access shall be governed by a Creative Commons licence – details of these licences and which Creative Commons licence will apply to this Work are set out in our licence referred to above.

Other than as permitted in any relevant BMJ Author's Self Archiving Policies, I confirm this Work has not been accepted for publication elsewhere, is not being considered for publication elsewhere and does not duplicate material already published. I confirm all authors consent to publication of this Work and authorise the granting of this licence.

For peer review only

A prospective observational study to examine health related quality of life and develop models to predict long-term patient reported outcomes six-months after hospital discharge with blunt thoracic injuries.

Mr. Edward BAKER (Corresponding Author)
NIHR Clinical Doctoral Research Fellow - Kings College London
edward.e.baker@kcl.ac.uk
Florence Nightingale Faculty of Nursing, Midwifery and Palliative Care
King's College London
James Clerk Maxwell Building
57 Waterloo Road
London
SE1 8WA

Dr Ceri Battle
Consultant Physiotherapist - Swansea Bay University Health Board ceri.battle@wales.nhs.uk

Dr Abhishek Banjeri
Emergency Medicine Consultant - Buckingham Healthcare NHS Trust
abhishekbanerji@nhs.net

Dr Edward Carlton
Emergency Medicine Consultant – North Bristol NHS Trust
eddcarlton@gmail.com

Ms. Christin Dixon
Trauma Nursing Lead – Surrey and Sussex Healthcare NHS Trust
christine.dixon14@nhs.net

Dr Jennifer Ferry
Consultant Anaesthetist – Aneurin Bevan University Health Board
jennifer.ferry@wales.nhs.uk

Dr Philip Hopkins
Intensive Care Consultant and ICM Research Lead – Kings College Hospital NHS Foundation Trust
p.hopkins@nhs.net

Dr Robert Jones
Emergency Medicine Consultant – Barnsley Hospital NHS Foundation Trust
robertjones5@nhs.net

Mr. Trevor Murrells
Faculty Statistician – King's College London
trevor.murrells@kcl.ac.uk

Professor Christine Norton
Professor of Nursing Research – King's College London
christine.norton@kcl.ac.uk

For peer review only

Mr. Lee Patient
Nurse Consultant Emergency Medicine – St Georges University Hospital NHS Foundation Trust
lee.patient@stgeorges.nhs.uk

Professor Ashraf Rasheed
Professor of General Surgery – Aneurin Bevan University Health Board
ashraf.rasheed@wales.nhs.uk

Ms. Imogen Skene
Lead Research Nurse Emergency Medicine – Barts Health NHS Trust
i.skene@nhs.net

Dr Andrew Tabner
Emergency Medicine Consultant – University Hospitals of Derby and Burton NHS Foundation Trust
andrew.tabner@nhs.net

Dr Malcolm Tunnicliff
Emergency Medicine Consultant – King's College Hospital NHS Foundation Trusts
malcolm.tunnicliff@nhs.net

Ms. Louise Young
Lead Research Nurse Emergency Medicine – Imperial College Healthcare NHS Trust
louise.young10@nhs.net

Dr Andreas Xyrichis
Senior Lecturer in Nursing – King's College London
andreas.xyrichis@kcl.ac.uk

Dr Gerry Lee
Reader in Advanced Clinical Practice – King's College London
gerry.lee@kcl.ac.uk
Florence Nightingale Faculty of Nursing, Midwifery and Palliative Care
King's College London
James Clerk Maxwell Building
57 Waterloo Road
London
SE1 8WA

For peer review only

For peer review only - http://bmjopen.bmj.com/site/about/guidelines.xhtml

Abstract:

Objective: This study aimed to examine the long-term outcomes and health-related quality of life in patients with blunt thoracic injuries over six-months from hospital discharge and develop models to predict long-term patient reported outcomes.

Design: A prospective observational study using longitudinal survey design.

Setting: The study recruitment was undertaken at 12 UK hospitals which represented diverse geographical locations and covered urban, sub-urban and rural areas across England and Wales.

Participants: 377 patients admitted to hospital with blunt thoracic injuries were recruited between June 2018-October 2020.

Methods: Participants completed a bank of 2 quality of life surveys (Short Form-12 and EuroQol-5d-5l) and 2 pain questionnaires (Brief Pain Inventory and PainDetect questionnaire) at 4 time-points over the first 6-months after discharge from hospital. A total of 211 (63%) participants completed the outcomes data at 6-months after hospital discharge.

Outcomes Measures: Three outcomes were measured using pre-existing and validated patient reported outcome measures. Outcomes included: Poor Physical function (SF-12 Physical Component Score); Chronic pain (Brief Pain Inventory Pain Severity Score); and Neuropathic pain (PainDetect questionnaire).

Results: Despite a trend towards improving physical functional and pain at 6-months, outcomes did not return to participants perceived baseline level of function. At six-months after hospital discharge, 37% (n=77) of participants reported poor physical function; 36.5% (n=77) reported a chronic pain state; and 22% (n=47) reported pain with a neuropathic component. Predictive models were developed for each outcome highlighting important data collection requirements for predicting long-term outcomes in this population. Model diagnostics including calibration and discrimination statistics suggested good model fit in this development cohort.

Conclusions: This study identified the recovery trajectories for patients with blunt thoracic injuries over the first six-months after hospital discharge and present prognostic models for three important outcomes which after external validation could be used as clinical risk stratification scores.

Keywords: Trauma; Chest Injury; Rib Fractures; Predictive modelling; Long-term outcomes; Pain; Neuropathic Pain; Physical Functioning; Quality of Life; Patient Reported Outcome Measures.

For peer review only - http://bmjopen.bmj.com/site/about/guidelines.xhtml

For peer review only

Strengths and Limitations of the Study:

- This study highlights the physical and pain sequelae associated with recovery after blunt thoracic injuries and provides insights into the recovery trajectory for this population.
- Data are derived from geographically diverse trauma hospital as across England and Wales.
- The longitudinal study design using patient reported outcome measures demonstrates the importance of eliciting health data from this population over throughout the post discharge recovery period.
- Patient reported outcome measures are valuable in outcome prediction, particularly where usual care does not include follow-up after hospital discharge.
- Despite the benefits of patient reported outcome measures, it is important to acknowledge the potential inherent bias within these measures.

For peer review only - http://bmjopen.bmj.com/site/about/guidelines.xhtml

A prospective observational study to examine health related quality of life and develop models to predict long-term patient reported outcomes six-months after hospital discharge with blunt thoracic injuries.

Background:

Blunt thoracic injury (BTI) is a term that covers a spectrum of different injury patterns and severities across all adult age groups.[1] It accounts for about 15% of injured patients presenting to trauma hospitals, with mortality rates reported between 4-20%.[2] Outcomes within trauma care have historically been measured primarily using mortality as a key outcome.[3] During the last two decades, there has been a decrease in the mortality rates of polytrauma patients in high income countries with organised trauma systems.[4] As the mortality rates have decreased, there has been a growing focus on measuring outcomes relating to injury morbidity.[3 5] There is a growing body of international research investigating the long-term outcomes of patients with BTI, but within the United Kingdom (UK), there is a paucity of BTI outcomes research with little consistency in the methods employed.[6 7]

For patients recovering from BTI, it has been identified that reduced physical function, persistent altered respiratory function, and chronic pain frequently impact on all aspects of daily living.[6] Health Related Quality of Life (HRQoL) provides a strong foundation for measuring the impact of these symptoms with a holistic and comprehensive approach.[8] It is important not to isolate individual components of daily living but consider them as a single entity, and HRQoL measurement provides a vehicle to do this.[9] Furthermore, it allows the development of theory driven interventions that impact on all elements of daily living.[10] For the purposes of this study HRQOL is defined as including all aspects of self-perceived well-being that are related to, or affected by, the presence of disease or treatment.[11]

Follow-up after hospital discharge with major trauma is often limited within the UK's National Health Service (NHS), resulting in limited clinical knowledge about the recovery journey of BTI patients.[12] This also leaves clinicians without a trajectory for recovery making it very difficult to adequately educate and prepare patients for self-management in the post hospital discharge recovery period.[13] Evidence from this study will be used to facilitate the identification of patients at risk of poor outcomes after BTI, help develop health promotion materials, and to support the development of follow-up interventions within BTI management.[13]

Having the ability to predict outcomes and develop methods for risk stratification is extremely important in trauma care.[14] There is a growing body of prognostic research in this area with simple

For peer review only - http://bmjopen.bmj.com/site/about/guidelines.xhtml

scoring systems developed and validated with effective predictive abilities across different health systems.[2] Where resources for patient follow-up are limited, it is key that effective methods for identifying patients in high-risk groups for complications or poor outcomes are in place, allowing resources to be focused on these patients at the greatest risk. The aim of this study was to examine the long-term outcomes and HRQoL in patients with BTI over six-months from hospital discharge and develop models to predict long-term patient reported outcomes. Study objectives were as follows:

- Investigate changes in HRQoL over 6 months after hospital discharge with BTI.
- Examine the evolution of pain and neuropathic pain over six months after discharge from hospital with BTI.
- Develop predictive models for poor physical functioning, chronic pain, and neuropathic pain at six-months after hospital discharge with BTI.

Methods:

This prospective multicentred observational study employed longitudinal survey methods to follow-up patients recovering from BTI over a six-month period from hospital discharge. This research forms the quantitative component of a mixed methods study investigating recovery after BTI. The qualitative components will be reported separately.

Study Setting:

The study population sample was recruited from Major Trauma Centres (MTC) (Level 1) (n=5) and Trauma Units (Level 2-3) (n=7) from diverse geographical locations within urban, sub-urban and rural areas across England and Wales. Sites were set up in a stepwise fashion and in total 12 acute hospital sites recruited participants between 6 to 10 months in two separate recruitment cohorts between June 2018 and March 2020.

Eligibility Criteria:

Inclusion criteria:

- Participant is willing and able to give informed consent for participation in the study
- Male or female, aged 16 years or above
- Admitted to a Major Trauma Centre/Trauma Unit with primary BTI
- Admitted to hospital for a period of 24 hours or greater

For peer review only - http://bmjopen.bmj.com/site/about/guidelines.xhtml

Exclusion criteria:

- Unstable spinal fracture or spinal cord injury (SCI)
- Traumatic brain injury (TBI) with cognitive impairment
- Altered mental status that would prevent accurate self-assessment through questionnaires or prevent informed consent into study
- Associated unstable pelvic fracture or open abdomen requiring extended periods of treatment in hospital
- Limb injuries from same traumatic event that require surgical intervention
- Participant is a prisoner / in police custody at time of recruitment

Data Collection:

Data collection was undertaken at four timepoints: (i) Hospital discharge (including a pre-injury estimation of HRQoL); (ii) 1 month after discharge; (iii) 3 months after discharge; and (iv) 6 months after discharge. Potential participants were identified by the local clinical team or the clinical research nurses at each site. Demographic and injury related data were collated throughout the acute admission using a previously tested case report form,[1] and site staff conducted data collection at hospital discharge. Post discharge follow-up was conducted by the central research team at the host university using either postal surveys or electronic surveys hosted by SurveyMonkey.

Ethics Considerations:

Formal informed written consent was taken from all participants after information sheets had been provided and sufficient time was allowed for discussion with family and clinical research staff. Ethical approval was granted by the 'Hampshire A' South Central Research Ethics Committee in June 2018 (ref: 18/SC/0230). Participants were informed that everyone who completed data collection at all timepoints would be placed in a prize draw to win a £50 shopping voucher to recognise the time associated with participation in this longitudinal follow-up study.

Sample Size:

This observational study had an exploratory design and pragmatic approach to sample size. Although there is no consensus on the sample requirements for logistic regression analysis, based on previous studies we aimed to have 10 events for each predictor variables included in the model.[2 3] As it was likely that 10 predictor variables would be used in the modelling process for each model, the aim was to achieve 100 events (i.e., outcomes of interest) in the sample.

For peer review only - http://bmjopen.bmj.com/site/about/guidelines.xhtml

Outcome Measures:

HRQoL was explored firstly at a global level through the Physical Component Score (PCS) and Mental Component Score (MCS) of the Short Form-12 (SF-12),[15] and secondly, using the individual levels of the EuroQol 5-dimensions, 5-Levels (EQ5D-5L).[16 17] Pain was previously recognised as an ongoing problem throughout post hospital discharge recovery.[6] In this study, pain severity and impact were measured using the Brief Pain Inventory[18] and neuropathic pain was assessed using the PainDetect Score[19] Using existing literature on HRQoL after BTI,[6 20] 3 key outcomes were selected:

1. **Physical Function** is an individual's ability to undertake actions that involve physical activities, ranging from activities of daily living (ADLs) to more complex activities that involve a combination of skills, often within a social context.[3] For the purposes of this study, physical functioning was measured using the PCS of the SF-12 at six-months after discharge from hospital. A PCS score of ≤35 (Range: 0-100 with higher scores equalling better Physical function) was taken as defining poor physical functioning from previous literature.[15]
2. **Chronic Pain** is defined as pain that persists beyond the normal expected healing time and therefore lacks the acute warning function of physiological nociception. Pain is normally considered as chronic when it lasts or recurs for more than three to 6-months.[21] For this study, chronic pain was defined as having a Brief Pain Inventory (BPI) Pain Severity Score (PSS) of ≥3.5 (Range: 0-10 and higher scores equals increased pain severity) at 6-months after hospital discharge.[22]
3. **Neuropathic pain** is defined as pain caused by a lesion or disease of the somatosensory nervous system.[23] For the purposes of this study, participants with a potential neuropathic pain component were identified using the PainDetect Questionnaire.[19] A PainDetect score ≥12 (Range: -1 – 38) at 6-months after hospital discharge defined our cohort of patients with a potential neuropathic pain component.[24]

Selection of candidate predictor variables:

A collection of predictor variables was required which covered multiple potentially influential factors relating to the outcomes of interest. Initially, during the study development, a long list of candidate variables were selected from previous literature.[6 10 25 26] From this long-list, shortlists of variables for each outcome were compiled. Decision-making for entering variables into the modelling process was based on their clinical significance and achieving statistical significance at the 5% level in the univariate analysis phase. There was an 'a priori' plan for candidate variables to be selected in 3 phases, initially using only variables that are readily available in clinical practice, secondly, variables

For peer review only - http://bmjopen.bmj.com/site/about/guidelines.xhtml

248 that are currently not readily available in clinical practice, and finally using variables collected at post
249 discharge timepoints.

250 Statistical analysis:

251 Data analysis was undertaken using SPSS v.26. Statistical data were presented using descriptive and
252 inferential statistical methods and reported according to the Strengthening the Reporting of
253 Observational Studies in Epidemiology (STROBE) statement.[27] Binary and categorical data were
254 presented as percentages and number of cases. Normally distributed continuous data were
255 presented as means with standard deviations whilst non-parametric continuous data were
256 presented using median values with interquartile ranges. The statistical tests applied for
257 binary/categorical data were Chi-square test of association, except where cell counts were ≤5 when
258 the Fisher's exact test was used. Comparisons of two independent groups, where data were
259 normally distributed, were undertaken using the Student t-test. When data were not normally
260 distributed a Mann-Whitney U test was used. A Shapiro-Wilks test was used to determine whether
261 the data were normally distributed or not.

262 Missing data was addressed using the multiple imputation procedure in SPSS. The imputation model
263 assumes that data were Missing at Random (MAR). Most missing data were found within the follow-
264 up phase of data collection where units of data (i.e., surveys) were not returned. The imputation
265 model included all predictor variables with potential for inclusion in the modelling process including
266 candidate predictor variables from within the hospital admission and from the post discharge follow-
267 up. An *'a priori'* decision was made not to impute the outcome variables within the study. A total of
268 10 imputed datasets were created.

269 The predictive model framework was developed in accordance with the TRIPOD (Transparent
270 Reporting of multivariable predication model for Individual Prognosis Or Diagnosis) methodology[28]
271 and the study methods based on previously published prognostic modelling studies.[2 3 29] A
272 multivariable logistic regression analysis was used to evaluate the association of the predictors with
273 the outcome (dependent variable) in each imputed data set using the forward step wise method.[30]
274 In this process, SPSS automatically produced a final model that aggregated the results across all the
275 imputed datasets.

276 Calibration was evaluated using a Hosmer-Lemeshow test where a p-value >0.05 was considered to
277 represent good calibration. In addition, graphical calibration plots were developed. A receiver
278 operating characteristic (ROC) curve was plotted for each model. A c-statistic (area under the
279 receiver operator curve) was calculated, that tested whether the amount of discrimination was

For peer review only - http://bmjopen.bmj.com/site/about/guidelines.xhtml

significantly different from random (c-statistic of 0.5 indicates that discrimination is no better than the toss of a coin). Performance of the final model was assessed using sensitivity, specificity, positive predictive value (PPV), and negative predictive value (NPV).[31] Throughout statistical significance was recognised at the 5% level (p<0.05).

Patient and Public involvement:

A patient and public reference group was formed and involved in the development phases of this study including during protocol development and application for ethical approval but have not been involved in data collection, analysis, or interpretation.

Results:

A total of 337 patients were recruited to the study. Overall, the combined response rate at all timepoints was 72%. At hospital discharge 95% (n=320) completed the discharge data collection timepoint, subsequently, 62% (n=210) and 43% (n=144) completed data collection at 1-month and 6-month after discharge respectively. At 6-months, 63% (211) of the total sample completed follow-up where the study outcomes data were collected. Of the 211 participants who completed the 6-month follow-up, n=200 (94%) completed discharge data collection, 174 (82%) completed the 1-month data collection timepoint, and 142 (67%) completed data collection at 3-months after discharge.

Overall missingness within the dataset was predominately from 'unit missing data' (i.e., completed survey not returned by participant) as individual items of missing data within a returned survey were followed up with participants at the time of data collection. Predominate item missingness were found in candidate predictor variables collected at the 1-month follow-up timepoint where 17% (n=37) of variables were missing, and at three months where, 33% (n=69) of variables were missing within the dataset.

The median age of participants was 63 years (IQR: 51.0-75.0) and 69% (n=231) were male. The predominant mechanism of injury was falls <2m (45%, n=153) and the median number of rib fractures was 6.0 (3.0-7.0). At six-months after discharge, 37% (n=77) of the sample self-reported poor physical functioning, 37% (n=77) reported chronic pain, and 22% (n=47) reported pain with a likely neuropathic pain component. **Table 1** summarises key demographic data for the total sample. Further greater detailed demographic data is presented in **Table S1** as a supplementary file to this study.

For peer review only - http://bmjopen.bmj.com/site/about/guidelines.xhtml

Table 1: Demographics of entire cohort and a comparison of characteristics for each outcome of interest at six-months after discharge

Result: n= (%) unless otherwise indicated	Total sample (n=337)	Poor Physical Function (n=77)	Good Physical Function (n=134)	p-value	Chronic Pain (n=77)	No Chronic Pain (n=134)	p-value	Neuropathic component likely (n=47)	Neuropathic component unlikely (n=163)	p-value
Age ^	**63 (51.0-75.0)**	74.0 (70.0-77.0)	61.5 (52.0-77.0)	<0.001*	74.0 (66.0-79.5)	66.5 (52.0-77.3)	0.030*	61.0 (48.0-71.8)	74.0 (56.0-78.0)	0.86
Male (%)	**231 (68.5)**	49 (63.6)	97 (72.4)	0.19	54 (70.1)	91 (67.9)	0.80	33 (70.2)	112 (68.7)	0.85
Injury Severity Score ^	**9.0 (4.0-16.0)**	14.0 (9.0-16.0)	16.0 (9.0-20.0)	0.34	10.0 (9.0-18.5)	16.0 (9.0-20.0)	1.00	13.0 (9.0-16.8)	15.0 (9.0-20.0)	0.97
Mechanism of Injury:										
RTC	**78 (23.1)**	14 (18.2)	38 (28.4)		19 (24.7)	33 (24.6)		9 (19.1)	43 (26.4)	
Crush	**11 (3.3)**	3 (3.9)	4 (3.0)		1 (1.3)	6 (4.5)		1 (2.1)	6 (3.7)	
Ped vs. Vehicle	**4 (1.2)**	1 (1.3)	2 (1.5)		1 (1.3)	2 (1.5)		1 (2.1)	2 (1.2)	
Fall>2m	**69 (20.5)**	13 (16.9)	30 (22.4)	0.23	18 (23.4)	25 (18.7)	0.78	13 (27.7)	30 (18.4)	0.82
Fall<2m	**153 (45.4)**	44 (57.1)	54 (40.3)		37 (48.1)	61 (45.5)		22 (46.8)	76 (46.6)	
Assault	**14 (4.2)**	1 (1.3)	4 (3.0)		1 (1.3)	3 (2.2)		1 (2.1)	3 (1.8)	
CPR	**4 (1.2)**	1 (1.3)	0		0	1 (0.7)		0	1 (0.6)	
Rapid deceleration	**4 (1.2)**	0	2 (1.5)		0	2 (1.5)		0	2 (1.2)	
Respiratory co-morbidity	**70 (20.8)**	21 (27.3)	21 (15.7)	0.042*	21 (27.3)	20 (14.9)	0.031*	12 (25.5)	29 (17.8)	0.24
Cardiac co-morbidity	**147 (43.6)**	49 (63.6)	53 (39.6)	0.001*	47 (61.0)	54 (40.3)	0.004*	27 (57.4)	74 (45.4)	0.15
Neuro co-morbidity	**50 (14.8)**	20 (26.0)	9 (6.7)	<0.001*	19 (24.7)	10 (7.5)	0.001*	10 (21.3)	19 (11.7)	0.092
MSK co-morbidity	**89 (26.4)**	26 (33.8)	35 (26.1)	0.24	30 (39.0)	31 (23.1)	0.016*	16 (34.0)	45 (27.6)	0.39
Pre-Injury reg. analgesic use	**61 (18.1)**	23 (29.9)	15 (11.2)	0.001*	30 (39.0)	8 (6.0)	<0.001*	19 (40.4)	19 (11.7)	<0.001*
No. Rib Fractures ^	**6.0 (3.0-7.0)**	5.0 (4.0-10.0)	7.0 (4.3-8.0)	0.98	5.0 (4.5-8.0)	7.0 (3.8-9.0)	0.55	6.0 (4.3-11.5)	7.0 (4.0-9.0)	0.63
Radiological Flail	**60 (17.8)**	13 (16.9)	29 (21.6)	0.41	16 (20.8)	26 (19.4)	0.83	7 (14.9)	35 (21.5)	0.32
Extra-thoracic injuries	**104 (30.9)**	25 (32.5)	35 (26.1)	0.33	19 (24.7)	40 (29.9)	0.40	12 (25.5)	47 (28.8)	0.66
Complications:	**111 (32.9)**	34 (44.2)	33 (24.6)	0.003*	25 (32.5)	42 (31.3)	0.89	18 (38.3)	49 (30.1)	0.29
Post Admission Ventilation:	**26 (7.7)**	9 (11.7)	5 (3.7)	0.025*	6 (7.8)	8 (6.0)	0.62	5 (10.6)	9 (5.5)	0.22
ICU Length of Stay ^	**5.0 (3.0-9.0)**	7.0 (4.0-12.0)	5.0 (4.0-12.0)	0.097	6.0 (3.0-11.0)	5.5 (2.8-9.5)	0.72	7.5 (4.5-10.8)	5.0 (2.0-9.0)	0.21
Hospital Length of Stay ^	**6.0 (11.0)**	18.0 (11.0-20.0)	8.0 (5.0-19.3)	<0.001*	17.0 (6.5-20.0)	12.0 (5.8-20.3)	0.002*	15.5 (10.3-26.0)	14.0 (6.0-20.0)	0.026*

Abbrev: CPR = Cardio-pulmonary Resuscitation; ICU = Intensive Care Unit; IQR = Interquartile Range; Ped = Pedestrian; RTC = Road Traffic Collision; ^ = Median (IQR). * = Statistically significant at 5% level

For peer review only - http://bmjopen.bmj.com/site/about/guidelines.xhtml

For peer review only

Changes in HRQoL over six-months after discharge from hospital

Physical function was clearly affected by the injuries experienced by this cohort. Measures of HRQoL are presented in **Table 2**. As expected, the median PCS score (SF-12) saw a 25% (50.4-25.9) drop from the pre-injury estimate through to the initial hospital discharge timepoint representing a substantial negative change in physical functioning. Over the 6-month follow-up there were incremental improvements in the median PCS score (30.4, 39.7, 42.2 at 1, 3 and 6-month respectively) but the sample failed to return to their baseline estimate of physical function (-8.2%) or population norms (-7.8%) within the 6-month follow-up period. During the same timeframe, the impact of injury on mental health was lesser than on physical function. The median MCS score (SF-12) dropped from 52.9 to 44.7 (-8.2) from the pre-injury estimate through to hospital discharge, dropping a further 1% to 45.6 at 1-month after hospital discharge. Between the 1 and 6 months the median MCS increased to 53.7 which exceeded the pre-injury estimate and 3.7% greater than the population norms for England.

For peer review only - http://bmjopen.bmj.com/site/about/guidelines.xhtml

Table 2: Cohort outcomes with comparisons between outcomes of interest at six-months after discharge

	Outcome	**Total sample**<br><br>**Median (IQR)**	Poor physical function Median (IQR)	Good physical function Median (IQR)	p-value	Chronic Pain<br><br>Median (IQR)	No chronic pain<br><br>Median (IQR)	p-value	Neuropathic pain likely Median (IQR)	Neuropathic pain unlikely Median (IQR)	p-value
Pre-Injury	SF-12 PCS	**50.4 (39.0-55.4)**	39.0 (31.7-50.0)	52.3 (48.9-55.9)	<0.001*	42.4 (33.1-51.0)	52.1 (45.7-55.6)	<0.001*	43.2 (31.7-51.2)	51.5 (41.8-55.5)	<0.001*
	SF-12 MCS	**53.2 (42.6-58.1)**	51.3 (37.9-57.7)	55.2 (49.1-58.7)	0.006*	51.8 (40.8-57.5)	54.8 (48.5-58.8)	0.022*	48.0 (39.6-57.8)	54.7 (48.1-58.7)	0.019*
Discharge	SF-12 PCS	**25.9 (20.9-32.7)**	23.2 (19.3-27.2)	27.4 (22.2-35.1)	0.001*	24.3 (19.1-30.3)	26.7 (21.6-33.9)	0.020*	24.0 (19.2-29.9)	26.3 (21.3-32.7)	0.041*
	SF-12 MCS	**44.8 (36.3-54.5)**	40.8 (36.5-52.7)	48.5 (39.8-55.8)	0.024*	44.2 (37.6-57.0)	47.2 (37.6-55.3)	0.89	40.9 (36.0-50.3)	47.9 (38.6-55.8)	0.028*
	BPI Pain Severity Score	**5.0 (3.8-6.5)**	5.5 (3.9-6.8)	4.8 (3.3-6.0)	0.085	5.3 (3.9-6.5)	4.8 (3.3-6.0)	0.053	5.8 (4.4-6.9)	4.8 (3.3-6.0)	0.009*
	BPI Pain Interference Score	**6.1 (4.1-7.2)**	6.6 (4.8-8.2)	5.0 (3.5-6.7)	<0.001*	6.4 (4.4-7.5)	5.7 (3.7-7.2)	0.22	6.4 (4.7-8.0)	5.6 (3.7-7.1)	0.016*
	PDQ: Low Risk ++	**222 (69.4)**	48 (64.9)	95 (73.1)		47 (63.5)	96 (74.4)		24 (52.2)	119 (75.8)	
	PDQ: Moderate Risk ++	**67 (20.9)**	18 (24.3)	26 (20.0)	0.42	22 (29.7)	21 (16.3)	0.075	17 (37.0)	26 (16.6)	0.006*
	PDQ: High Risk ++	**31 (9.7)**	8 (10.8)	9 (6.9)		5 (6.8)	12 (9.3)		5 (10.9)	12 (7.6)	
1-month	SF-12 PCS +	**30.4 (10.3)**	27.0 (6.0)	33.5 (7.5)	<0.001*	27.7 (6.0)	32.9 (7.8)	<0.001*	27.8 (5.8)	32.1 (7.9)	<0.001*
	SF-12 MCS +	**45.6 (19.0)**	40.8 (12.2)	46.8 (11.2)	0.001*	40.7 (12.7)	46.7 (10.9)	0.001*	35.6 (12.3)	47.0 (10.6)	<0.001*
	BPI Pain Severity Score +	**4.0 (3.3)**	4.7 (2.3)	3.2 (1.9)	<0.001*	5.2 (2.0)	3.0 (1.9)	<0.001*	5.5 (2.2)	3.5 (2.0)	<0.001*
	BPI Pain Interference Score +	**4.4 (4.3)**	5.6 (2.6)	3.7 (2.5)	<0.001*	5.8 (2.4)	3.6 (2.5)	<0.001*	6.6 (2.4)	3.8 (2.4)	<0.001*
	PDQ: Low Risk ++	**142 (67.6)**	39 (61.9)	83 (73.5)		34 (57.6)	88 (75.2)		14 (38.9)	108 (77.1)	
	PDQ: Moderate Risk ++	**45 (21.4)**	14 (22.2)	22 (19.5)	0.14	13 (22.0)	23 (19.7)	0.005*	9 (25.0)	27 (19.3)	<0.001*
	PDQ: High Risk ++	**23 (11.0)**	10 (15.9)	8 (7.1)		12 (20.3)	6 (5.1)		13 (36.1)	5 (3.6)	
3-months	SF-12 PCS	**39.9 (31.6-50.9)**	31.0 (25.7-35.8)	44.4 (36.7-53.1)	<0.001*	31.5 (26.3-36.0)	43.8 (36.7-53.1)	<0.001*	32.0 (25.7-39.4)	42.1 (33.0-52.4)	<0.001*
	SF-12 MCS	**51.3 (39.8-57.8)**	42.2 (33.7-55.4)	54.2(46.3-57.9)	0.001*	42.2 (34.6-53.6)	54.5 (46.7-58.6)	<0.001*	40.7 (28.6-48.7)	53.6 (45.8-58.0)	<0.001*
	BPI Pain Severity Score	**2.5 (0.8-5.0)**	4.5 (2.5-6.6)	1.3 (0.5-3.5)	<0.001*	5.0 (3.3-6.5)	1.0 (0.3-2.8)	<0.001*	5.0 (3.3-6.3)	1.5 (0.5-3.5)	<0.001*
	BPI Pain Interference Score	**1.3 (0.3-4.7)**	4.7 (1.3-7.2)	0.9 (0.1-3.1)	<0.001*	5.0 (3.0-7.0)	0.6 (0.1-2.0)	<0.001*	4.7 (3.6-7.1)	0.9 (0.1-3.2)	<0.001*
	PDQ: Low Risk ++	**110 (76.4)**	28 (60.9)	79 (83.2)		23 (48.9)	84 (89.4)	<0.001*	9 (29.0)	98 (89.1)	
	PDQ: Moderate Risk ++	**15 (10.4)**	7 (15.2)	8 (8.4)	0.012*	9 (19.1)	6 (6.4)		9 (29.0)	6 (5.5)	<0.001*
	PDQ: High Risk ++	**19 (13.2)**	11 (23.9)	8 (8.4)		15 (31.9)	4 (4.3)		13 (41.9)	6 (5.5)	
6-months	SF-12 PCS +	**42.2 (21.8)**	30.5 (26.2-32.8)	51.0 (42.1-55.5)	<0.001*	32.9 (27.3-37.5)	50.3 (38.7-55.5)	<0.001*	32.4 (26.2-37.5)	46.2 (35.5-54.8)	<0.001*
	SF-12 MCS +	**53.7 (16.0)**	40.6 (31.9-54.7)	53.9 (44.7-58.7)	<0.001*	39.5 (31.0-47.0)	55.9 (47.2-59.8)	<0.001*	34.5 (26.1-44.8)	54.0 (43.9-58.8)	<0.001*
	BPI Pain Severity Score	**2.0 (0-5.0)**	5.0 (2.8-6.7)	0.8 (0-2.6)	<0.001*	5.0 (4.5-7.0)	0.5 (0-1.9)	<0.001*	5.0 (3.8-7.0)	1.0 (0-3.3)	<0.001*
	BPI Pain Interference Score	**1.7 (0-4.9)**	5.5 (2.9-7.4)	0.6 (0-2.3)	<0.001*	5.9 (3.8-7.4)	0.4 (0-1.5)	<0.001*	6.6 (4.6-8.3)	0.9 (0-3.1)	<0.001*
	PDQ: Low Risk ++	**164 (77.7)**	44 (57.9)	119 (88.8)		40 (51.9)	123 (92.5)		0	163 (100)	
	PDQ: Moderate Risk ++	**28 (13.3)**	17 (22.4)	11 (8.2)	<0.001*	20 (26.0)	8 (6.0)	<0.001*	28 (59.6)	0	<0.001*
	PDQ: High Risk ++	**19 (9.0)**	15 (19.7)	4 (3.0)		17 (22.1)	2 (1.5)		19 (40.4)	0	

Key: + = Mean (±SD); ++ = n (%); * = Statistically significant at 5% level.

For peer review only - http://bmjopen.bmj.com/site/about/guidelines.xhtml

The impact of BTI on individual components of HRQoL were measured using the EuroQol-5d-5L questionnaire. **Table 3** presents the individual dimension/level data for each timepoint. At hospital discharge, the lowest scored (best) dimension was Anxiety and Depression with 43% of the sample reporting no problems in this dimension. At 6-months after discharge: 'Mobility', 'Self-Care', 'Usual Activities' and 'Anxiety/Depression' were the best rated dimensions, with between 42% and 73% of the sample reporting no problems in these areas. The highest scoring (worst) dimensions at hospital discharge were 'Usual Activities' where 42% (n=135) reported being wholly unable to complete usual activities. Between 42-43% of the sample reported moderate (3), severe (4) or extreme (5) pain at both 3-months and 6-months after discharge from hospital (42.3% (n=61) at 3-months and 43.1% (n=91) at six months). At 6-months after injury, there was a proportion of participants who reported severe or extreme problems in all dimensions, and these exceeded the pre-injury estimated levels. This demonstrates that although the 6-month follow-up period was sufficient to observe recovery in a vast proportion of this sample, there were participants who reported extreme disability beyond the follow-up.

For peer review only - http://bmjopen.bmj.com/site/about/guidelines.xhtml

Table 3: Individual dimension and level data from EuroQoL-5D-(5L)

(n= (%))	No Problems	Slight Problems	Moderate Problems	Severe Problems	Unable/extreme Problems
**Mobility:**					
Pre-injury	**204 (60.5)**	50 (14.8)	47(13.9)	33 (9.8)	3 (0.9)
Discharge	50 (15.6)	78 (24.4)	**102 (31.9)**	72 (22.5)	18 (5.6)
One-Month	65 (31.0)	**76 (36.2)**	38 (18.1)	26 (12.4)	5 (2.4)
Three-Months	**71 (49.3)**	36 (25.0)	17 (11.8)	16 (11.1)	4 (2.8)
Six-Months	**110 (52.1)**	41 (19.4)	32 (15.2)	23 (10.9)	5 (2.4)
**Self-Care:**					
Pre-injury	**269 (79.8)**	33 (9.8)	23 (6.8)	10 (3.0)	2 (0.6)
Discharge	68 (21.3)	**92 (28.8)**	81 (25.3)	59 (18.4)	20 (6.3)
One-Month	**108 (51.4)**	59 (28.1)	30 (14.3)	10 (4.8)	3 (1.4)
Three-Months	**106 (73.6)**	21 (14.6)	9 (6.3)	6 (4.2)	2 (1.4)
Six-Months	**153 (72.5)**	31 (14.7)	20 (9.5)	5 (2.4)	2 (0.9)
**Usual Activities:**					
Pre-injury	**224 (66.5)**	53 (15.7)	33 (9.8)	19 (5.6)	8 (2.4)
Discharge	21 (6.6)	36 (11.3)	72 (22.5)	56 (17.5)	**135 (42.2)**
One-Month	20 (9.5)	**95 (45.2)**	41 (19.5)	29 (13.8)	25 (11.9)
Three-Months	49 (34.0)	**51 (35.4)**	22 (15.3)	9 (6.3)	13 (9.0)
Six-Months	**89 (42.2)**	62 (29.4)	34 (16.1)	14 (6.6)	12 (5.7)
**Pain/Discomfort:**					
Pre-injury	**157 (46.6)**	94 (27.9)	61 (18.1)	18 (5.3)	7 (2.1)
Discharge	14 (4.4)	41 (12.8)	**151 (47.2)**	74 (23.1)	40 (12.5)
One-Month	15 (7.1)	49 (23.3)	**112 (53.3)**	22 (10.5)	12 (5.7)
Three-Months	37 (25.7)	46 (31.9)	**47 (32.6)**	10 (6.9)	4 (2.8)
Six-Months	**72 (34.1)**	48 (22.7)	**72 (34.1)**	11 (5.2)	8 (3.8)
**Anxiety/Depression:**					
Pre-injury	**188 (55.8)**	79 (23.4)	54 (16.0)	11 (3.3)	5 (1.5)
Discharge	**138 (43.1)**	85 (26.6)	64 (20.0)	20 (6.3)	13 (4.1)
One-Month	**100 (47.6)**	38 (18.1)	57 (27.1)	7 (3.3)	8 (3.8)
Three-Months	**82 (56.9)**	27 (18.8)	26 (18.1)	4 (2.8)	5 (3.5)
Six-months	**119 (56.4)**	42 (19.9)	33 (19.9)	8 (3.8)	9 (4.3)

For peer review only

For peer review only - http://bmjopen.bmj.com/site/about/guidelines.xhtml

Evolution of chronic pain and pain with a neuropathic component

Subjective pain evaluated through the BPI Pain Severity Score (PSS) reflects the persistent levels of pain experienced by study participants throughout the 6-month study follow-up period. Pain related outcome measures are presented in **Table 2**. Although there were consistent incremental reductions in the median pain score over time, at 6-months after injury the median pain score was 2.0 (IQR: 0-5.0); 36.5% (n=77) of participants were reporting chronic pain. The median BPI Pain Interference Score (PIS) also demonstrated incremental decreases until the 3-month timepoint where there appears to be a plateau, followed by a marginal deterioration of the median PIS (1.3 (IQR: 0.3-4.7) to 1.7 (IQR: 0-4.9)). This highlights that participants reporting pain that impacted on daily activities at three months were likely to continue to experience these interferences at 6-months after hospital discharge. Similarly, median self-reported analgesic effectiveness decreased from 60.0% (IQR: 50.0-80.0) at hospital discharge to 50% (IQR: 12.5-70.0) at 3-months which then plateaued and was 50% (IQR: 10.0-80.0) at 6-months after hospital discharge. Individual components of the BPI PSS and BPI PIS can be seen in the supplementary file **Table S2 and S3.**

At hospital discharge and 1-month after, 30.6% (n= 98) and 32.4% (68%) (respectively) of patients reported moderate or high risk of having a neuropathic pain component. At 3-months and 6-months, there was a reduction in the number of participants reporting neuropathic pain (23.6% (n=34) and 22.3% (n=47) respectively) with 13.2% (n=19) and 9.0% (n=19) respectively reporting PainDetect scores in the high-risk category (representing 90% likelihood of having a neuropathic pain component).

Development of Predictive models for the three outcomes of interest

Table 4 highlights three models developed in this study, one for each outcome. Initially candidate predictors were selected from variables readily available within the inpatient clinical journey (including hospital discharge), as this would be most useful in clinical practice. Unfortunately, we were unable to isolate a model with acceptable levels of discrimination and calibration using these variables alone. In a second approach models were developed using variables from the post discharge follow-up period (Table 4). Three models with acceptable levels of calibration and discrimination were identified. All models require measures collected at hospital discharge, 1-month, and 3-months after discharge to predict the outcomes of interest.

Model 1: Poor physical function at 6-months after hospital discharge was predicted using the following: the combination of the measures of physical function both prior to injury and at 3-months

For peer review only - http://bmjopen.bmj.com/site/about/guidelines.xhtml

after injury, a measurement of pain levels at 1-month after discharge, and a measurement of the impact of pain on the individuals walking ability at hospital discharge. **Model 2:** It was possible to predict chronic pain at 6-months after hospital discharge using the follow variables: Pre-injury regular use of analgesic agents, measures of physical function before the injury and at 3-months after discharge, and a measurement of pain severity at 1- and 3-months after discharge. **Model 3:** neuropathic pain was predicted using the following variables: starting Pregabalin during the acute hospital admission, the Mental Component Score at 1-month and the impact of pain on mood at 3-months after discharge, and the outcome of taking the PainDetect Questionnaire at 3-months after discharge.

For peer review only

For peer review only - http://bmjopen.bmj.com/site/about/guidelines.xhtml

Table 4: Predictive models for three outcomes of interest

	Co-efficient	Std error	Odds Ratio	P-value	95% CI
**Model 1: Poor physical function at 6-months after hospital discharge**					
Pre-Injury PCS (Range: 0-100) (1 step increment increase)	-0.093	0.019	0.911	<0.001	0.878-0.945
Current Pain at 1-month after injury (Range 0-10) (1 step increment increase)	0.224	0.078	1.252	0.004	1.075-1.457
PCS at 3 months (Range: 0-100) (1 step increment increase)	-0.040	0.014	0.960	0.005	0.934-0.988
Impact of pain on walking at discharge (Range: 0-10) (1 step increment increase)	0.139	0.057	1.149	0.015	1.027-1.285
Constant	*3.745*	*1.132*	-	*0.001*	-
**Model 2: Chronic pain at 6-months after hospital discharge**					
Regular Analgesia use before injury	1.813	0.533	6.130	0.001	2.158-17.414
Pre-Injury PCS (Range: 0-100) (1 step increment increase)	-0.039	0.019	0.962	0.044	0.926-0.999
Pain score at 1 month after discharge (Range: 0-10) (1 step increment increase)	0.270	0.089	1.310	0.003	1.099-1.560
PCS at 3 months (Range: 0-100) (1 step increment increase)	-0.051	0.015	0.951	0.001	0.922-0.980
Current Pain score 3 months after discharge (Range: 0-10) (1 step increment increase)	0.219	0.084	1.245	0.009	1.057-1.467
Constant	*1.361*	*1.098*	-	*0.215*	-
**Model 3: Likely neuropathic component to pain at 6-months after hospital discharge**					
Commenced on Pregabalin during Hospital admission	2.340	1.156	10.377	0.043	1.076-100.113
MCS at 1 month after discharge (Range: 0-100) (1 step increment increase)	-0.058	0.026	0.944	0.026	0.897-0.993
Pain Detect score at 3 months (Range: -1-38) (1 step increment increase)	0.174	0.043	1.190	<0.001	1.094-1.295
Impact of Pain on Mood at 3 months after discharge (Range 0-10) (1 step increment increase)	0.192	0.095	1.212	0.043	1.212-1.006
Constant	*-1.421*	*1.188*	-	*0.232*	-

For peer review only

Insert Figure 1 and Figure 2 here please.

For peer review only - http://bmjopen.bmj.com/site/about/guidelines.xhtml

The ROC diagrams for each model are presented in **Figure 1**. **Figure 2** presents the graphitised calibration plots whereby plot points present risk of outcome in tenths of patients with similar predicted probabilities. The line highlights the relationship between the observed frequency and the predicted probability of developing a poor outcome. **Model 1** for predicting poor physical functioning at 6 months after discharge had a c-statistic (area under the receiver operator curve) of 0.846 (Std error: 0.028; p<0.001; 95%CI 0.790-0.901) with a Hosmer and Lemeshow Chi-square value of 5.570 (p=0.695) with 8 degrees of freedom (df), demonstrating acceptable goodness-of-fit. This resulted in a model with a sensitivity of 79.0% and a specificity of 81.2%, a PPV of 63.6% and a NPV of 90.3%. **Model 2** for predicting chronic pain at 6-months after discharge had a c-statistic of 0.878 (Std error: 0.023; p<0.001; 95%CI 0.833-0.923) with a Hosmer and Lemeshow Chi-Square value of 6.778 (p=0.561) with 8 degrees of freedom (df), also demonstrating acceptable goodness-of-fit. This resulted in a model with a sensitivity of 63.6% and a specificity of 86.5%, a PPV or 73.1% and a NPV of 80.4%. Finally, **Model 3** for predicting pain with a neuropathic component had a c-statistic of 0.910 (Std error: 0.027; <0.001; 95%CI 0.857-0.964) with a Hosmer and Lemeshow Chi-square value of 2.221 (p=0.912) with 8 degrees of freedom (df), demonstrating acceptable goodness-of-fit. This resulted in a model with a sensitivity of 61.3% and a specificity of 92.7%, a PPV of 70.4% and a NPV of 89.5%. The overall accuracy of models 1-3 were 80.6%, 78.1% and 85.8% respectively. The calibration plots for each model **(Fig. 2)** demonstrated acceptable levels of calibration as many of the plots were close to the 45-degree line.

Discussion:

This study investigated changes in self-reported HRQoL and pain outcomes over a 6-month period from hospital discharge safter BTI. The study has provided new insights into the recovery of this patient group within the UK. This study has identified the high levels of pain and poor physical function experienced after BTI in the UK and measured the presence of neuropathic pain components within this population. The process of predictive prognostic modelling has identified factors that predict outcomes, suggesting that it is possible to develop a risk stratification score. This score could have clinical utility in identifying patients at risk of poor functional outcomes or chronic/neuropathic pain, subject to external validation in a future study. In respect to the models presented in this study, it is important to remember that good model performance in a development sample does not necessarily mean good predictive abilities in a new external validation dataset.

For peer review only - http://bmjopen.bmj.com/site/about/guidelines.xhtml

479 The limitations of using generic Patient Reported Outcome Measures (PROMs) to measure HRQoL have been reported previously.[7 32] Although validated within the major trauma population, it remains unclear how specific and comprehensive generic outcome measures (e.g., SF-12 and EQ5D-5L) are in groups like the BTI population. Although a trauma PROM has previously been developed,[33] there is a need for injury specific measures, particularly where unique outcomes have been identified.[7 34] Physical functioning was measured at a global level in this study using the SF-12 PCS and using the individual levels of the EuroQoL-5d-5L questionnaire. Both measures highlighted the extent of the altered physical function experienced by this population, which had not returned to their perceived baseline level of function at 6-months. In previous non-UK research, sequela related to BTI has been measured up to 2-years after injury highlighting the long-term impact of BTI.[35] Due to funding, it was not possible to extend the follow-up in the current study, but the 6-month timepoint was clinically ideal for the prognostic modelling process, particularly as Marasco et al. highlighted that patients reporting poor physical function at 6-months after injury reported poor physical function at 1 year and greater odds of reporting poor physical function at 2-years after injury.[35]

494 One key factor that influences HRQoL after BTI is pain, and the efficacy of pain management.[6] Measures of pain severity within this study highlighted 43.1% (n=91) of participants reported pain that was moderate, severe, or extreme at 6-months after hospital discharge with a median PSS of 2.0. For these participants, the median analgesic effectiveness was 50.0% (IQR: 80.0-10.0) demonstrating how a chronic pain state existed where analgesic agents were not effective in managing pain. The variables in model 2 demonstrate the complexity in chronic pain management particularly in identifying those at risk through the predictive modelling process. Within this model it was not possible to predict the likelihood of whether a participant would experience chronic pain at 6-months after discharge using clinically available variables only. The final model included variables from pre-injury estimates through to 3-months after discharge which highlighted the need to alter our current model for patient follow-up to enable measurements at 1 and 3 months after discharge. Unsurprisingly, participants using regular analgesic agents prior to their injury had an odds ratio greater than six highlighting the need to identify patients with pre-existing chronic pain states during hospital admission as these patients are most likely to have ongoing injury related pain requirements at six months after hospital discharge.

509 There was greater odds of reporting neuropathic pain at 6 months if the participant had been initiated on a neuropathic pain agent (e.g. pregabalin) during the acute hospital admission. This suggests that clinical teams and specialist pain teams are accurately identifying patients at risk of neuropathic pain and commencing treatment. This unfortunately also highlights that these patients

For peer review only - http://bmjopen.bmj.com/site/about/guidelines.xhtml

are still progressing to develop symptoms of neuropathic pain at six-months after discharge which suggests that greater input from specialist pain services after hospital discharge may be required to manage this pain trajectory. The model for neuropathic pain highlights an association with the MCS of the SF-12 at one month and the effect of pain on mood at 3-months after hospital discharge. The relationship between neuropathic pain and mental health has previously been identified in the non-trauma literature.[36] Although there was a less substantial drop in SF-12 MCS compared to the PCS and this returned to the pre-injury baseline within the 6-month follow-up period, this shows the importance of measuring mental wellbeing as both a part of HRQoL and as an important predictor of neuropathic pain.

This study has developed a model for each of the three outcomes of interest: physical function, chronic pain, and neuropathic pain at 6-months after hospital discharge with BTI. The next phase of the modelling process will be to conduct a further study to validate the predictive ability of the models within an external sample of participants with BTI. This study will further explore the follow-up needs of patients with BTI and look at effective ways of collecting these data needed to predict 6-month outcomes. It will also be of interest to investigate these outcomes beyond the 6-months follow-up period used in this study. In the meantime, these predictive variables have clinical utility for clinicians reviewing patients during the follow-up period in primary and secondary care settings and these variables may be useful in highlighting patients at risk who need referral to specialist services (e.g., physiotherapy or specialist pain services).

Strengths and limitations of this study:

Although we recruited a moderate sized sample using the exploratory approach to this study, we did not achieve the aim of recruiting 100 events relating to our three outcomes of interest. Care was taken to ensure that that the number of variables imputed into each model were justified by the number participants reporting the outcomes of interest. There are reports within the literature that the arbitrary rule of 10 cases per variable does not necessarily negatively impact on the model fit.[37] During subsequent validation of the models, care will be taken to assess for overfitting of the models but from the model diagnostics presented here, there is no suggestion of overfitting within the current sample.[31] It is important to remember that the final models are to some degree variable selection method dependent. In an ideal world, it would have been beneficial to use more than one method of selection but in the case of this study, it was not possible to undertake backward selection because of concerns of overfitting the model.

Overall, the combined response rate of 72% at all timepoints is comparable to equivalent studies undertaken previously in this area of trauma research.[6] Despite this, the response rate was a

For peer review only - http://bmjopen.bmj.com/site/about/guidelines.xhtml

546 limitation as the key outcomes were measured at the 6-month timepoint resulting in only 63% (n=211) of the overall recruited sample being included in the modelling process. For further studies investigating outcomes within a longitudinal follow-up process it will be important to use these data to consider potential sample sizes required to adequately power these studies.

550 As subjective outcome measures have been used throughout this study, it is important to acknowledge the risk of bias which is inherent within these measures, particularly in relation to participants selecting the extremes of a rating scale.[38] Care has been taken within the study to control this bias through the instructions given to patients prior to completing each questionnaire and investigation of outliers within the sample during data analysis.[39] In future research, it would be interesting to compare the predictive ability of both subjective outcomes (i.e. SF-12 PCS for physical function) with an objective measure (grip strength testing as an estimate of physical functional ability for example) and compare the difference in the predictive functions of these outcomes.[40]

558 Finally, although most of the study was undertaken before the Covid-19 pandemic began in the UK, there will be seen and unseen impacts of the Covid-19 pandemic on this study.[41] The final three sites closed to recruitment 2 to 4 weeks early causing them to not meet recruitment targets. Furthermore, follow-up continued throughout the first wave of the pandemic in the UK. Although we were not able to collect data relating Covid-19 infection amongst our sample, we were not informed that any participants had been infected with the virus. Despite this, there is potential bias relating to lockdown, social isolation that remains unmeasured within our sample which may have impacted on the self-reported outcome measures reported in this study.[42]

566 Conclusions:

567 It remains vital that the interdisciplinary team do not underestimate the impact of the long-term outcomes associated with BTI, particularly relating to physical functioning, chronic pain, and neuropathic pain. This study has demonstrated how predictive models can be developed for these outcomes and highlight how PROMS can be used to develop prognostic models particularly in longitudinal processes where objective clinical data is not routinely collected. These models add to our understanding of which factors influence the development of sequela after hospital discharge with BTI and after external validation, will have clinical utility in the risk stratification of high-risk patient groups.

For peer review only - http://bmjopen.bmj.com/site/about/guidelines.xhtml

Reference:

1. Baker EJ, Lee GA. A Retrospective Observational Study Examining the Effect of Thoracic Epidural and Patient Controlled Analgesia on Short-term Outcomes in Blunt Thoracic Trauma Injuries. *Medicine (Baltimore)* 2016;95(2):e2374. doi: 10.1097/MD.0000000000002374
2. Battle C, Hutchings H, Lovett S, et al. Predicting outcomes after blunt chest wall trauma: development and external validation of a new prognostic model. *Crit Care* 2014;18(3):R98. doi: 10.1186/cc13873 [published Online First: 2014/05/14]
3. de Graaf MW, Reininga IHF, Heineman E, et al. The development and internal validation of a model to predict functional recovery after trauma. *PLoS One* 2019;14(3):e0213510. doi: 10.1371/journal.pone.0213510 [published Online First: 2019/03/14]
4. Cole E, Lecky F, West A, et al. The Impact of a Pan-regional Inclusive Trauma System on Quality of Care. *Ann Surg* 2016;264(1):188-94. doi: 10.1097/SLA.0000000000001393
5. Martino C, Russo E, Santonastaso DP, et al. Long-term outcomes in major trauma patients and correlations with the acute phase. *World J Emerg Surg* 2020;15:6. doi: 10.1186/s13017-020-0289-3 [published Online First: 2020/01/13]
6. Baker E, Xyrichis A, Norton C, et al. The long-term outcomes and health-related quality of life of patients following blunt thoracic injury: a narrative literature review. *Scand J Trauma Resusc Emerg Med* 2018;26(1):67. doi: 10.1186/s13049-018-0535-9 [published Online First: 2018/08/17]
7. Craxford S, Deacon C, Myint Y, et al. Assessing outcome measures used after rib fracture: A COSMIN systematic review. *Injury* 2019;50(11):1816-25. doi: 10.1016/j.injury.2019.07.002 [published Online First: 2019/07/04]
8. Wilson IB, Cleary PD. Linking clinical variables with health-related quality of life. A conceptual model of patient outcomes. *JAMA* 1995;273(1):59-65.
9. Ojelabi AO, Graham Y, Haighton C, et al. A systematic review of the application of Wilson and Cleary health-related quality of life model in chronic diseases. *Health Qual Life Outcomes* 2017;15(1):241. doi: 10.1186/s12955-017-0818-2 [published Online First: 2017/12/11]
10. Baker E, Woolley A, Xyrichis A, et al. How does the implementation of a patient pathway-based intervention in the acute care of blunt thoracic injury impact on patient outcomes? A systematic review of the literature. *Injury* 2020 doi: 10.1016/j.injury.2020.06.002 [published Online First: 2020/06/04]
11. Karimi M, Brazier J. Health, Health-Related Quality of Life, and Quality of Life: What is the Difference? *Pharmacoeconomics* 2016;34(7):645-9. doi: 10.1007/s40273-016-0389-9
12. Vernon D, Brown JE, Griffiths E, et al. Reducing readmission rates through a discharge follow-up service. *Future Healthc J* 2019;6(2):114-17. doi: 10.7861/futurehosp.6-2-114
13. Braaf S, Ameratunga S, Nunn A, et al. Patient-identified information and communication needs in the context of major trauma. *BMC Health Serv Res* 2018;18(1):163. doi: 10.1186/s12913-018-2971-7 [published Online First: 2018/03/07]
14. Keene DJ, Vadher K, Willett K, et al. Predicting patient-reported and objectively measured functional outcome 6 months after ankle fracture in people aged 60 years or over in the UK: prognostic model development and internal validation. *BMJ Open* 2019;9(7):e029813. doi: 10.1136/bmjopen-2019-029813 [published Online First: 2019/07/23]
15. Ware J, Kosinski M, Keller SD. A 12-Item Short-Form Health Survey: construction of scales and preliminary tests of reliability and validity. *Med Care* 1996;34(3):220-33.
16. Devlin NJ, Parkin D, Browne J. Patient-reported outcome measures in the NHS: new methods for analysing and reporting EQ-5D data. *Health Econ* 2010;19(8):886-905. doi: 10.1002/hec.1608
17. Parkin D, Rice N, Devlin N. Statistical analysis of EQ-5D profiles: does the use of value sets bias inference? *Med Decis Making* 2010;30(5):556-65. doi: 10.1177/0272989X09357473 [published Online First: 2010/03/12]

For peer review only

For peer review only - http://bmjopen.bmj.com/site/about/guidelines.xhtml

18. Kumar SP. Utilization of brief pain inventory as an assessment tool for pain in patients with cancer: a focused review. *Indian J Palliat Care* 2011;17(2):108-15. doi: 10.4103/0973-1075.84531
19. Freynhagen R, Baron R, Gockel U, et al. painDETECT: a new screening questionnaire to identify neuropathic components in patients with back pain. *Curr Med Res Opin* 2006;22(10):1911-20. doi: 10.1185/030079906X132488
20. Battle CE, Hutchings H, James K, et al. The risk factors for the development of complications during the recovery phase following blunt chest wall trauma: a retrospective study. *Injury* 2013;44(9):1171-6. doi: 10.1016/j.injury.2012.05.019 [published Online First: 2012/06/12]
21. Treede RD, Rief W, Barke A, et al. A classification of chronic pain for ICD-11. *Pain* 2015;156(6):1003-7. doi: 10.1097/j.pain.0000000000000160
22. Boonstra AM, Schiphorst Preuper HR, Balk GA, et al. Cut-off points for mild, moderate, and severe pain on the visual analogue scale for pain in patients with chronic musculoskeletal pain. *Pain* 2014;155(12):2545-50. doi: 10.1016/j.pain.2014.09.014 [published Online First: 2014/09/17]
23. Murnion BP. Neuropathic pain: current definition and review of drug treatment. *Aust Prescr* 2018;41(3):60-63. doi: 10.18773/austprescr.2018.022 [published Online First: 2018/06/01]
24. Mathieson S, Lin C. painDETECT questionnaire. *J Physiother* 2013;59(3):211. doi: 10.1016/S1836-9553(13)70189-9
25. Unsworth A, Curtis K, Asha SE. Treatments for blunt chest trauma and their impact on patient outcomes and health service delivery. *Scand J Trauma Resusc Emerg Med* 2015;23:17. doi: 10.1186/s13049-015-0091-5 [published Online First: 2015/02/08]
26. Kourouche S, Buckley T, Munroe B, et al. Development of a blunt chest injury care bundle: An integrative review. *Injury* 2018;49(6):1008-23. doi: 10.1016/j.injury.2018.03.037 [published Online First: 2018/04/07]
27. von Elm E, Altman DG, Egger M, et al. The Strengthening the Reporting of Observational Studies in Epidemiology (STROBE) Statement: guidelines for reporting observational studies. *Int J Surg* 2014;12(12):1495-9. doi: 10.1016/j.ijsu.2014.07.013 [published Online First: 2014/07/18]
28. Collins GS, Reitsma JB, Altman DG, et al. Transparent reporting of a multivariable prediction model for individual prognosis or diagnosis (TRIPOD): the TRIPOD statement. *BMJ* 2015;350:g7594. doi: 10.1136/bmj.g7594 [published Online First: 2015/01/07]
29. Pareek N, Kordis P, Beckley-Hoelscher N, et al. A practical risk score for early prediction of neurological outcome after out-of-hospital cardiac arrest: MIRACLE2. *Eur Heart J* 2020 doi: 10.1093/eurheartj/ehaa570 [published Online First: 2020/07/30]
30. Shipe ME, Deppen SA, Farjah F, et al. Developing prediction models for clinical use using logistic regression: an overview. *J Thorac Dis* 2019;11(Suppl 4):S574-S84. doi: 10.21037/jtd.2019.01.25
31. Alba AC, Agoritsas T, Walsh M, et al. Discrimination and Calibration of Clinical Prediction Models: Users' Guides to the Medical Literature. *JAMA* 2017;318(14):1377-84. doi: 10.1001/jama.2017.12126
32. Hoffman K, Cole E, Playford ED, et al. Health outcome after major trauma: what are we measuring? *PLoS One* 2014;9(7):e103082. doi: 10.1371/journal.pone.0103082 [published Online First: 2014/07/22]
33. Attenberger C, Amsler F, Gross T. Clinical evaluation of the Trauma Outcome Profile (TOP) in the longer-term follow-up of polytrauma patients. *Injury* 2012;43(9):1566-74. doi: 10.1016/j.injury.2011.01.002 [published Online First: 2011/01/20]
34. Rosenberg GM, Stave C, Spain DA, et al. Patient-reported outcomes in trauma: a scoping study of published research. *Trauma Surg Acute Care Open* 2018;3(1):e000202. doi: 10.1136/tsaco-2018-000202 [published Online First: 2018/09/04]

For peer review only

For peer review only - http://bmjopen.bmj.com/site/about/guidelines.xhtml

35. Marasco S, Lee G, Summerhayes R, et al. Quality of life after major trauma with multiple rib fractures. *Injury* 2015;46(1):61-5. doi: 10.1016/j.injury.2014.06.014 [published Online First: 2014/06/21]
36. Torta R, Ieraci V, Zizzi F. A Review of the Emotional Aspects of Neuropathic Pain: From Comorbidity to Co-Pathogenesis. *Pain Ther* 2017;6(Suppl 1):11-17. doi: 10.1007/s40122-017-0088-z [published Online First: 2017/11/24]
37. Ogundimu EO, Altman DG, Collins GS. Adequate sample size for developing prediction models is not simply related to events per variable. *J Clin Epidemiol* 2016;76:175-82. doi: 10.1016/j.jclinepi.2016.02.031 [published Online First: 2016/03/08]
38. Dowling NM, Bolt DM, Deng S, et al. Measurement and control of bias in patient reported outcomes using multidimensional item response theory. *BMC Med Res Methodol* 2016;16:63. doi: 10.1186/s12874-016-0161-z [published Online First: 2016/05/26]
39. Tempelaar D, Rienties B, Nguyen Q. Subjective data, objective data and the role of bias in predictive modelling: Lessons from a dispositional learning analytics application. *PLoS One* 2020;15(6):e0233977. doi: 10.1371/journal.pone.0233977 [published Online First: 2020/06/12]
40. Koskinen S, Hokkinen EM, Wilson L, et al. Comparison of subjective and objective assessments of outcome after traumatic brain injury using the International Classification of Functioning, Disability and Health (ICF). *Disabil Rehabil* 2011;33(25-26):2464-78. doi: 10.3109/09638288.2011.574776 [published Online First: 2011/05/03]
41. Baker E, Lee G. Reflecting on the challenges of sustaining emergency care research during the Covid-19 pandemic. *Int Emerg Nurs* 2020;51:100894. doi: 10.1016/j.ienj.2020.100894 [published Online First: 2020/07/13]
42. Baker E, Clark LL. Biopsychopharmacosocial approach to assess impact of social distancing and isolation on mental health in older adults. *Br J Community Nurs* 2020;25(5):231-38. doi: 10.12968/bjcn.2020.25.5.231

Figure Legends/Caption:

Figure 1: Receiver Operator Curve (ROC) for predictive models for three outcomes of interest

Figure 2: Calibration plot for model using expected and observed probabilities of developing poor outcomes

For peer review only

Declarations:

Author Contribution:

EB, GL, AX, CN, PH conceived and designed the study and developed the study protocol. AB, CB, EC, CD, JF, RJ, LP, AR, IS, AT, MT, & LY were local primary investigators and coordinated and contributed significantly to the recruitment and data collection processes. Data analysis was planned with ED, GL, TM & CB. Data analysis was undertaken by EB and reviewed by TM. EB developed the first draft of the manuscript and EC, CB, AX, CN, PH, & GL made subsequent revisions. All authors commented on and approved the manuscript prior to submission for publication. EB takes overall responsibility for the manuscript.

Acknowledgements:

The research team would like to acknowledge the study participants who gave up their time to participate in this interview study and the clinical research staff at each site who contributed to the participants recruitment and initial data collection for the Rib Injury Outcomes Study.

Conflict of Interest:

Nothing to declare.

Funding Statement:

Edward Baker is funded by a National Institute for Health Research (NIHR), Clinical Doctoral Research Fellowship for this research project (ICA-CDRF-2016-02-006). The publication presents independent research funded by the National Institute for Health Research (NIHR).

For peer review only - http://bmjopen.bmj.com/site/about/guidelines.xhtml

The views expressed are those of the authors and not necessarily those of the NHS, the NIHR or the Department of Health and Social Care.

Data Statement:

The authors confirm that the data supporting the findings of this study are available within the article and its supplementary materials.

For peer review only

For peer review only - http://bmjopen.bmj.com/site/about/guidelines.xhtml

Figure 1: Receiver Operator Curve (ROC) for predictive models for three outcomes of interest

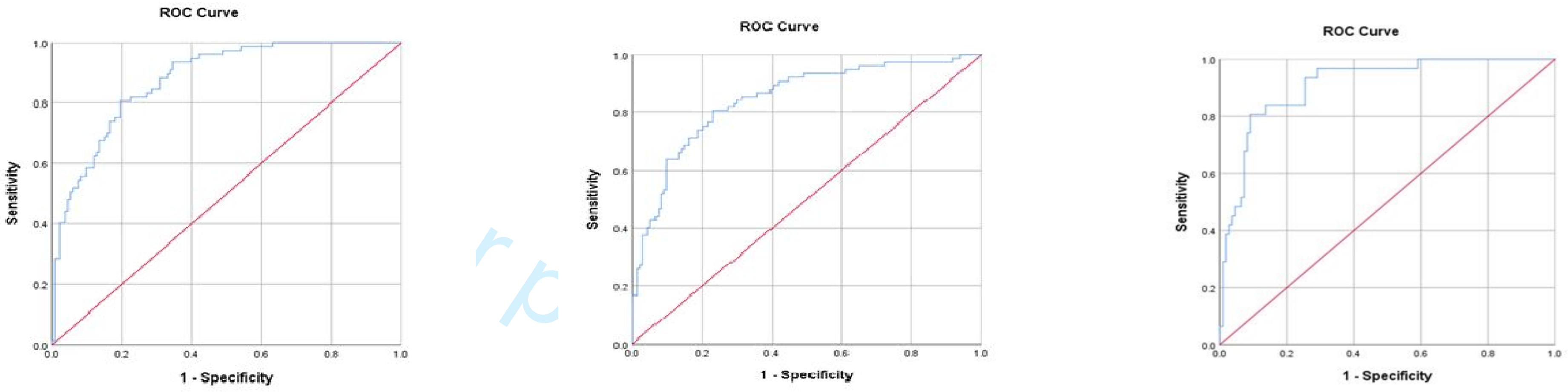

A: Model 1: Physical **B: Model 2: Chronic** **C: Model 3: Neuropathic**

Figure 2: Calibration plot for model using expected and observed probabilities of developing poor outcomes

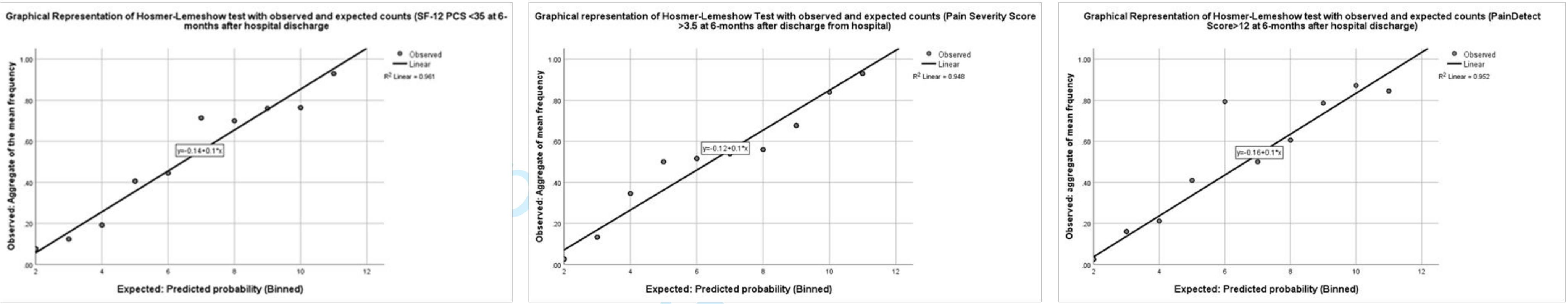

Model 1: Physical Functioning

Model 2: Chronic Pain

Model 3: Neuropathic Pain

Supplementary file: Table S1: Extended Characteristics of the Sample/Candidate predictor variables

Result: n= (%) unless otherwise indicated	**Total sample (n=337)**	**6/12 PCS≤35** (n=77)	**6/12 PCS>35** (n=134)	p-value	**6/12 Pain Severity Score ≥3.5** (n=77)	**6/12 Pain Severity Score <3.5** (n=134)	p-value	**6/12 PDQ score ≥12** (n=47)	**6/12 PDQ score <12** (n=163)	p-value
Age (Median (IQR))	**63 (51.0-75.0)**	74.0 (70.0-77.0)	61.5 (52.0-77.0)	<0.001*	74.0 (66.0-79.5)	66.5 (52.0-77.3)	0.030*	61.0 (48.0-71.8)	74.0 (56.0-78.0)	0.86
Male	**231 (68.5)**	49 (63.6)	97 (72.4)	0.19	54 (70.1)	91 (67.9)	0.80	33 (70.2)	112 (68.7)	0.85
Injury Severity Score (Median (IQR))	**9.0 (4.0-16.0)**	14.0 (9.0-16.0)	16.0 (9.0-20.0)	0.34	10.0 (9.0-18.5)	16.0 (9.0-20.0)	1.00	13.0 (9.0-16.8)	15.0 (9.0-20.0)	0.97
Battle/StUMBle Score (Median (IQR))	**20.0 (14.0-27.0)**	21.0 (16.5-28.5)	20.0 (13.0-28.0)	0.13	22.0 (15.5-30.0)	20.0 (14.0-26.5)	0.18	21.0 (16.0-29.0)	20.0 (14.0-28.0)	0.33
**Mechanism of Injury:**										
RTC	**78 (23.1)**	14 (18.2)	38 (28.4)		19 (24.7)	33 (24.6)		9 (19.1)	43 (26.4)	
Crush	**11 (3.3)**	3 (3.9)	4 (3.0)		1 (1.3)	6 (4.5)		1 (2.1)	6 (3.7)	
Ped vs. Vehicle	**4 (1.2)**	1 (1.3)	2 (1.5)		1 (1.3)	2 (1.5)		1 (2.1)	2 (1.2)	
Fall>2m	**69 (20.5)**	13 (16.9)	30 (22.4)	0.23	18 (23.4)	25 (18.7)	0.78	13 (27.7)	30 (18.4)	0.82
Fall<2m	**153 (45.4)**	44 (57.1)	54 (40.3)		37 (48.1)	61 (45.5)		22 (46.8)	76 (46.6)	
Assault	**14 (4.2)**	1 (1.3)	4 (3.0)		1 (1.3)	3 (2.2)		1 (2.1)	3 (1.8)	
CPR	**4 (1.2)**	1 (1.3)	0		0	1 (0.7)		0	1 (0.6)	
Rapid deceleration	**4 (1.2)**	0	2 (1.5)		0	2 (1.5)		0	2 (1.2)	
**Pre-morbid state:**										
Respiratory	**70 (20.8)**	21 (27.3)	21 (15.7)	0.042*	21 (27.3)	20 (14.9)	0.031*	12 (25.5)	29 (17.8)	0.24
Cardiac	**147 (43.6)**	49 (63.6)	53 (39.6)	0.001*	47 (61.0)	54 (40.3)	0.004*	27 (57.4)	74 (45.4)	0.15
Neuro	**50 (14.8)**	20 (26.0)	9 (6.7)	<0.001*	19 (24.7)	10 (7.5)	0.001*	10 (21.3)	19 (11.7)	0.092
MSK	**89 (26.4)**	26 (33.8)	35 (26.1)	0.24	30 (39.0)	31 (23.1)	0.016*	16 (34.0)	45 (27.6)	0.39
Cancer Current	**9 (2.7)**	4 (5.2)	3 (2.2)	0.079	4 (5.2)	3 (2.2)	0.009*	3 (6.4)	4 (2.5)	0.38
Cancer Historical	**26 (7.7)**	12 (15.6)	10 (7.5)		14 (18.2)	8 (6.0)		4 (8.5)	18 (11.0)	
DM2	**33 (9.8)**	9 (11.7)	11 (8.2)	0.41	5 (6.5)	15 (11.2)	0.26	5 (10.6)	15 (9.2)	0.77
No of Comorbidities (Median (IQR))	**2.0 (0-4.0)**	3.0 (2.0-5.0)	1.0 (0-3.0)	<0.001*	3.0 (1.5-4.0)	1.0 (0-3.0)	<0.001*	2.0 (1.0-5.0)	2.0 (0-3.0)	0.083
Chronic Pain	**27 (8.0)**	11 (14.3)	8 (6.0)	0.042*	13 (16.9)	6 (4.5)	0.003*	7 (14.9)	12 (7.4)	0.11
Regular Analgesic Use	**61 (18.1)**	23 (29.9)	15 (11.2)	0.001*	30 (39.0)	8 (6.0)	<0.001*	19 (40.4)	19 (11.7)	<0.001*
Smoking Status:										
Current smoker	**73 (21.7)**	11 (14.3)	21 (15.7)		10 (13.0)	22 (16.4)		11 (23.4)	21 (12.9)	

For peer review only

Ex-Smoker	**90 (26.7)**	25 (32.5)	37 (27.6)	0.87	27 (35.1)	34 (25.4)	0.068	18 (38.3)	43 (26.4)	0.047*
Never smoked	**122 (36.2)**	30 (39.0)	52 (67.5)		23 (29.9)	59 (44.0)		14 (29.8)	68 (41.7)	
Smoking Status unknown	**50 (14.8)**	11 (14.3)	23 29.9)		17 (22.1)	17 (12.7)		4 (8.5)	30 (18.4)	
Pack years (Median (IQR))	**8.6 (5.0-20.0)**	29.0 (16.5-39.5)	16.9 (4.6-36.5)	0.047*	23.1 (6.8-30.0)	20.0 (5.0-39.0)	0.74	24.1 (7.1-31.9)	20.0 (5.0-38.5)	0.96
**Indicators of Injury Severity:**										
No. Rib Fractures (Median (IQR))	**6.0 (1.8-9.5)**	4.0 (3.0-7.0)	5.0 (3.0-7.0)	0.98	4.0 (3.0-7.5)	4.0 (3.0-7.0)	0.55	4.0 (3.0-8.0)	4.0 (3.0-7.0)	0.63
Rib fracture Categories:										
0	**19 (5.6)**	5 (6.5)	8 (6.0)	0.30	6 (7.8)	7 (5.2)	0.82	1 (2.1)	12 (7.4)	0.67
1-4	**160 (47.5)**	39 (50.6)	58 (43.3)		33 (42.9)	63 (47.0)		25 (53.2)	71 (43.6)	
5-9	**114 (33.8)**	22 (28.6)	52 (38.8)		46 (59.7)	28 (20.9)		14 (29.8)	60 (36.8)	
10-15	**29 (8.6)**	9 (11.7)	9 (6.7)		7 (9.1)	11 (8.2)		5 (10.6)	13 (8.0)	
15+	**15 (4.5)**	2 (2.6)	7 (5.2)		3 (2.9)	6 (4.5)		2 (4.3)	7 (4.3)	
Other Indicators of Severity:										
Operative Rib Fixation	**16 (4.7)**	6 (7.8)	3 (2.2)	0.05*	2 (2.6)	7 (5.2)	0.36	3 (6.4)	6 (3.7)	0.42
First Rib fracture	**31 (9.2)**	6 (7.8)	13 (9.7)	0.64	10 (13.0)	9 (6.7)	0.13	7 (14.9)	12 (7.4)	0.11
Bilateral rib fractures	**44 (13.1)**	9 (11.7)	19 (14.2)	0.61	11 (14.3)	17 (12.7)	0.76	10 (21.3)	18 (11.0)	0.07
Scapular fracture	**27 (8.0)**	9 (11.7)	11 (8.2)	0.36	9 (11.7)	11 (8.2)	0.36	4 (8.5)	16 (9.8)	0.85
Clavicle fracture	**29 (8.6)**	8 (10.4)	14 (10.4)	0.50	8 (10.4)	14 (10.4)	0.50	4 (8.5)	18 (11.0)	0.08
Sternum Fracture	**26 (7.7)**	4 (5.2)	13 (9.7)	0.25	5 (6.5)	12 (9.0)	0.52	2 (4.3)	15 (9.2)	0.27
Manubrium Fracture	**8 (2.4)**	1 (1.3)	4 (3.0)	0.44	2 (2.6)	3 (2.2)	0.88	1 (2.1)	4 (2.5)	0.90
Thoracic Process #	**31 (9.2)**	6 (7.8)	12 (9.0)	0.77	11 (14.3)	7 (5.2)	0.024*	5 (10.6)	13 (8.0)	0.57
No. of Thoracic Process # (Median (IQR))	**3.0 (2.0-3.3)**	2.0 (1.0-3.5)	3.0 (2.0-4.0)	0.51	2.5 (1.8-4.0)	2.5 (1.0-3.3)	0.64	2.5 (1.3-3.8)	2.5 (1.3-3.8)	0.95
Lung Contusion	**76 (22.6)**	14 (18.2)	32 (23.9)	0.33	9 (11.7)	36 (26.9)	0.009*	8 (17.0)	37 (22.7)	0.40
Pneumothorax	**107 (31.8)**	20 (26.0)	50 (37.3)	0.17	20 (26.0)	50 (37.3)	0.19	15 (31.9)	55 (33.7)	0.85
Haemothorax	**80 (23.7)**	15 (19.5)	38 (28.4)	0.15	14 (18.2)	38 (28.4)	0.93	5 (10.6)	47 (28.8)	0.011*
Massive Haemothorax	**4 (1.2)**	0	1 (0.7)	0.45	1 (1.3)	0	0.19	0	1 (0.6)	0.59
Haemopneumothorax	**45 (13.4)**	7 (9.1)	23 (17.2)	0.11	21 (27.3)	8 (6.0)	0.27	4 (8.5)	25 (15.3)	0.23
Radiological Flail	**60 (17.8)**	13 (16.9)	29 (21.6)	0.41	16 (20.8)	26 (19.4)	0.83	7 (14.9)	35 (21.5)	0.32
Extra-thoracic injuries	**104 (30.9)**	25 (32.5)	35 (26.1)	0.33	19 (24.7)	40 (29.9)	0.40	12 (25.5)	47 (28.8)	0.66
**Initial Vital Signs:**										

For peer review only

Respiratory Rate (Mean (±SD))	**19.6 (±4.6)**	20.6 (4.8)	18.9 (4.1)	0.010*	20.1 (5.1)	19.2 (4.0)	0.15	20.2 (4.5)	19.3 (4.4)	0.25
Oxygen Saturation (Median (IQR))	**97.0 (95.8-99.0)**	96.0 (94.0-98.0)	97.0 (95.0-99.0)	0.054	96.0 (94.0-98.0)	97.0 (95.0-99.0)	0.024*	96.0 (94.0-98.0)	97.0 (95.0-99.0)	0.30
Heart Rate (Mean (±SD))	**81.5 (19.3)**	81.8 (4.1)	79.4 (17.3)	0.43	82.5 (22.1)	79.0 (17.3)	0.24	83.9 (17.2)	79.3 (19.6)	0.13
Systolic Blood Pressure (Mean (±SD))	**142.5 (23.0)**	144.8 (23.9)	141.7 (22.3)	0.38	147.9 (25.8)	140.2 (20.7)	0.032*	148.7 (25.3)	141.3 (22.0)	0.088
Temperature (Median (IQR))	**36.6 (36.1-36.8)**	36.5 (36.1-36.9)	36.5 (36.1-37.0)	0.41	36.5 (36.1-37.0)	36.5 (36.1-37.0)	0.90	36.4 (36.1-36.7)	36.5 (36.1-37.0)	0.17
**Chest Drain Management:**										
Intercostal Drain	**67 (19.9)**	14 (18.2)	33 (24.6)	0.28	14 (18.2)	32 (23.9)	0.32	9 (19.1)	37 (22.7)	0.60
Duration of Intercostal Drain (days) (Median (IQR))	**4.0 (3.0-6.0)**	4.0 (3.0-6.0)	4.0 (3.0-6.0)	0.99	5.5 (3.3-7.8)	4.0 (3.0-5.0)	0.09	4.0 (4.0-6.0)	4.0 (3.0-6.0)	0.49
**Admission Level of Care (1-3):**										
Level 1	**252 (74.8)**	59 (76.6)	106 (79.1)		60 (77.9)	104 (77.6)		35 (74.5)	129 (79.1)	
Level 2	**65 (19.3)**	13 (16.9)	26 (19.4)	0.14	13 (16.9)	26 (19.4)	0.48	9 (19.1)	30 (18.4)	0.41
Level 3	**18 (5.3)**	5 (6.5)	2 (1.5)		4 (5.2)	3 (2.2)		3 (6.4)	4 (2.5)	
**Initial Emergency Department analgesic management:**										
None	**28 (8.3)**	6 (7.8)	14 (10.4)		6 (7.8)	14 (10.4)		2 (4.3)	18 (11.0)	
Opioid	**207 (61.4)**	47 (61.0)	79 (59.0)	0.82	42 (54.5)	83 (61.9)	0.27	27 (57.4)	98 (60.1)	0.24
Non-opioid	**102 (30.3)**	24 (31.2)	41 (30.6)		29 (37.7)	36 (26.9)		18 (38.3)	47 (28.8)	
**Inpatient Analgesic management:**										
Intermittent analgesia only	**179 (53.1)**	37 (48.1)	77 (57.5)	0.19	37 (48.1)	76 (56.7)	0.20	22 (46.8)	91 (55.8)	0.28
Regional Analgesia	**106 (31.5)**	29 (37.7)	41 (30.6)	0.29	31 (40.3)	39 (29.1)	0.11	20 (42.6)	50 (30.7)	0.13
Thoracic Epidural	**50 (14.8)**	13 (16.9)	23 (17.2)	0.94	14 (18.2)	22 (16.4)	0.78	8 (17.0)	28 (17.2)	0.97
Time to commence TE (hrs) (Median (IQR))	**11.0 (8.0-24.8)**	9.0 (5.0-37.0)	11.5 (8.0-24.0)	0.60	22.0 (8.2-64.8)	9.2 (7.0-21.3)	0.13	9.3 (3.1-28.0)	10.5 (8.0-24.8)	0.31
Paravertebral block	**16 (4.7)**	5 (6.5)	5 (3.7)	0.36	8 (10.4)	2 (1.5)	0.004*	4 (8.5)	6 (3.7)	0.17
Time to commence PVB (hrs) (Median (IQR))	**15.0 (7.3-24.0)**	-	-	-	-	-	-	-	-	-
Intercostal Nerve Block	**4 (1.2)**	3 (3.9)	0	0.02	1 (1.3)	2 (1.5)	0.90	1 (2.1)	2 (1.2)	0.53
Time to commence Intercostal Nerve Block (hrs) (Median (IQR))	**49.5 (20.3-126.0)**	-	-	-	-	-	-	-	-	-
Serratus Plane Block	**22 (6.5)**	4 (5.2)	14 (10.4)	0.52	4 (5.2)	10 (7.5)	0.52	2 (4.3)	12 (7.4)	0.74

For peer review only

Time to commence SPB (hrs) (Median (IQR))	**12.5 (6.5-23.3)**	17.0 (15.3-72.0)	10.9 (6.6-22.9)	0.24	15.5 (13.1-24.3)	13.1 (6.6-22.9)	0.64	14.3 (12.5-16.0)	16.0 (7.6-23.6)	0.92
Erector Spinae Block	**20 (5.9)**	6 (7.8)	4 (3.0)	0.11	7 (9.1)	3 (2.2)	0.025	5 (10.6)	5 (3.1)	0.032*
Time to commence Erector Spinae Block (hrs) (Mean (±SD))	**30.4 (19.8)**	25.8 (29.5)	29.5 (27.3)	1.0	27.0 (22.5)	25.8 (-)	1.0	31.0 (32.0)	24.4 (24.7)	0.56
Combined Region Analgesia with Patient Controlled Analgesic	**43 (12.8)**	11 (14.3)	14 (10.4)	0.41	10 (13.0)	15 (11.2)	0.71	6 (12.8)	19 (11.7)	0.84
Patient Controlled Analgesic only	**93 (27.6)**	23 (29.9)	29 (21.6)	0.18	20 (26.0)	32 (23.9)	0.76	12 (25.5)	40 (24.5)	0.89
Time to commence PCA (hrs) (Median (IQR))	**10.0 (4.0-20.0)**	8.8 (4.0-20.0)	9.3 (3.0-16.0)	0.93	8.8 (3.0-20.0)	9.3 (3.5-16.5)	0.83	8.0 (2.0-19.0)	9.3 (4.0-17.0)	0.72
**Other Analgesics:**										
Gabapentin	**37 (11.0)**	11 (14.3)	11 (8.2)	0.16	11 (14.3)	11 (8.2)	0.17	9 (19.1)	13 (8.0)	0.028*
Pregabalin	**15 (4.5)**	5 (6.5)	4 (3.0)	0.23	7 (9.1)	2 (1.5)	0.009*	6 (12.8)	3 (1.8)	0.001*
Amitriptyline	**16 (4.7)**	7 (9.1)	3 (2.2)	0.024*	7 (9.1)	3 (2.2)	0.025*	5 (10.6)	5 (3.1)	0.032*
Transdermal Lignocaine	**113 (33.5)**	28 (36.4)	43 (32.1)	0.53	27 (35.1)	44 (32.8)	0.77	12 (25.5)	59 (36.2)	0.17
**Discharge Analgesia:**										
Opioid	**220 (65.3)**	51 (66.2)	95 (70.9)	0.48	55 (71.4)	90 (67.2)	0.57	33 (70.2)	112 (68.7)	0.85
Neuropathic Pain Agent	**41 (12.2)**	14 (18.2)	11 (8.2)	0.031*	12 (15.6)	13 (9.7)	0.21	10 (21.3)	15 (9.2)	0.024*
NSAIDS	**88 (26.1)**	13 (16.9)	37 (27.6)	0.078	19 (24.7)	31 (23.1)	0.82	14 (29.8)	36 (22.1)	0.28
Transdermal Lignocaine	**63 (18.7)**	15 (19.5)	24 (17.9)	0.78	16 (20.8)	23 (17.2)	0.53	8 (17.0)	31 (19.0)	0.76
No discharge analgesics prescribed	**34 (10.1)**	7 (9.1)	9 (6.7)	0.53	6 (7.8)	10 (7.5)	0.94	5 (10.6)	11 (6.7)	0.38
Number of discharge analgesic agents (Median (IQR))	**2.0 (1.0-3.0)**	2.0 (1.0-3.0)	2.0 (1.0-3.0)	0.68	2.0 (1.0-3.0)	2.0 (1.0-3.0)	0.85	2.0 (1.0-3.0)	2.0 (1.0-3.0)	0.39
**Complications:**	**111 (32.9)**	34 (44.2)	33 (24.6)	0.003*	25 (32.5)	42 (31.3)	0.89	18 (38.3)	49 (30.1)	0.29
Type 1 Respiratory Failure	**18 (5.4)**	2 (2.6)	4 (3.0)	0.87	2 (2.6)	4 (3.0)	0.86	2 (4.3)	4 (2.5)	0.62
Type 2 Respiratory Failure	**10 (3.0)**	5 (6.5)	1 (0.7)	0.016*	1 (1.3)	5 (3.7)	0.30	4 (8.5)	2 (1.2)	0.024*
Pulmonary Embolism	**4 (1.2)**	0	1 (0.7)	0.45	0	1 (0.7)	0.45	0	1 (0.6)	1.0
Pneumonia	**42 (12.5)**	13 (16.9)	12 (9.0)	0.086	11 (14.3)	14 (10.4)	0.42	8 (17.0)	17 (10.4)	0.22
Ventilator Associated Pneumonia	**5 (1.5)**	3 (3.9)	1 (0.7)	0.11	2 (2.6)	2 (1.5)	0.58	2 (4.3)	2 (1.2)	0.18
ARDS	**2 (0.6)**	1 (1.3)	0	0.19	0	1 (0.7)	0.45	0	1 (0.6)	1.0

For peer review only

LRTI	**58 (17.2)**	16 (20.8)	19 (14.2)	0.22	11 (14.3)	24 (17.9)	0.48	7 (14.9)	28 (17.2)	0.71
Treated with Abx	**110 (32.6)**	29 (37.7)	38 (28.4)	0.16	22 (28.6)	45 (33.6)	0.43	15 (31.9)	52 (31.9)	1.0
**Post Admission Ventilation:**	**26 (7.7)**	9 (11.7)	5 (3.7)	0.025*	6 (7.8)	8 (6.0)	0.62	5 (10.6)	9 (5.5)	0.22
CPAP	**4 (1.2)**	2 (2.6)	0	0.061	0	2 (1.5)	0.53	0	2 (1.2)	1.0
BiPAP	**1 (0.3)**	1 (1.3)	0	0.19	0	1 (0.7)	1.0	0	1 (0.6)	1.0
Invasive Ventilation	**17 (5.0)**	7 (9.1)	2 (1.5)	0.013*	3 (3.9)	6 (4.5)	1.0	2 (4.3)	7 (4.3)	1.0
High Flow nasal Oxygen	**19 (5.6)**	6 (7.8)	5 (3.7)	0.20	5 (6.5)	6 (4.5)	0.53	4 (8.5)	7 (4.3)	0.27
Moved to Critical Care	**29 (8.6)**	10 (13.0)	7 (5.2)	0.046*	6 (7.8)	11 (8.2)	0.90	5 (10.6)	12 (7.3)	0.54
ICU Length of Stay (Median (IQR))	**5.0 (3.0-9.0)**	7.0 (4.0-12.0)	5.0 (2.0-8.0)	0.097	6.0 (3.0-11.0)	5.5 (2.8-9.5)	0.72	7.5 (4.5-10.8)	5.0 (2.0-9.0)	0.21
Hospital Length of Stay (Median (IQR))	**11.0 (6.0-20.0)**	9.0 (6.0-16.5)	5.0 (3.0-8.0)	<0.001*	8.0 (5.0-12.5)	6.0 (3.0-9.5)	0.002*	8.0 (5.0-12.0)	6.0 (3.0-10.0)	0.026*
**Discharge outcome:**										
Home	**306 (90.8)**	68 (88.3)	128 (95.5)	0.010*	70 (90.9)	125 (93.3)	0.52	43 (91.5)	152 (93.3)	0.34
Rehabilitation Bed	**9 (2.7)**	6 (7.8)	0		3 (3.9)	3 (2.2)		3 (6.4)	3 (1.8)	
Repatriation to local hospital	**15 (4.5)**	3 (3.9)	5 (3.7)		3 (3.9)	5 (3.7)		1 (2.1)	7 (4.3)	
Long term care	**4 (1.2)**	0	1 (0.7)		1 (1.3)	0		0	1 (0.6)	

For peer review only

Supplementary File: Table S2: Extended Outcomes of interest measured at 6-months after discharge (The total sample)

Time-point	PROM	Min	Max	Median Value (IQR)	Dif. from Pop. Norms	Pop Norm
Pre-Injury	SF-12 Physical Component Score	13.1	64.9	50.4 (39.0-55.4)	0.4	50.0 (SD 1.0)
	SF-12 Mental Component Score	13.5	67.2	53.2 (42.6-58.1)	3.2	50.0 (SD 1.0)
	EQ Index Score	-0.3	1.0	0.81 (0.7-1.0)	-0.05	0.86 (SE 0.004)
	EQ VAS Score	10.0	100.0	80.0 (63.8-90.0)	-2.8	82.8 (SE 0.4)
Discharge	SF-12 Physical Component Score	10.9	55.5	25.9 (20.9-32.7)	-24.1	50.0 (SD 1.0)
	SF-12 Mental Component Score	15.9	68.9	44.8 (36.3-54.5)	-5.2	50.0 (SD 1.0)
	EQ Index Score	-0.5	1.0	0.32 (0.09-0.57)	-0.54	0.86 (SE 0.004)
	EQ VAS Score	0	99.0	50.0 (25.0-65.0)	-32.8	82.8 (SE 0.4)
	BPI Pain Severity Score	0	10.0	5.0 (3.8-6.5)	-	-
	BPI Pain Interference Score	0	10.0	6.1 (4.1-7.2)	-	-
	BPI Analgesic Effectiveness	0	100.0	70.0 (50.0-80.0)	-	-
	PainDetect Score	-1	29	10.0 (6.0-14.0)	-	-
	Prop. reporting Thoracic Pain (%)	-	-	283 (88.4)	-	-
	No. affected body regions (21)	0	16.0	2.0 (1.0-3.0)	-	-
1-month	SF-12 Physical Component Score (Mean (SD))	14.8	55.7	30.4 (10.3)	-19.6	50.0 (SD 1.0)
	SF-12 Mental Component Score (Mean (SD))	18.4	66.3	45.6 (19.0)	-4.4	50.0 (SD 1.0)
	EQ Index Score	-0.5	1.0	0.64 (0.41-0.74)	-0.22	0.86 (SE 0.004)
	EQ VAS Score	5.0	100.0	65.0 (45.0-80.0)	-17.8	82.8 (SE 0.4)
	BPI Pain Severity Score (Mean (SD))	0	10.0	4.0 (3.3)	-	-
	BPI Pain Interference Score (Mean (SD))	0	10.0	4.4 (4.3)	-	-
	BPI Analgesic Effectiveness	0	100.0	50.0 (30.0-70.0)	-	-
	PainDetect Score	0	35.0	9.0 (5.0-14.0)	-	-
	Prop. reporting Thoracic Pain (%)	-	-	162 (77.1)	-	-
	No. affected body regions (21)	0	12	2.0 (1.0-4.0)	-	-
3-months	SF-12 Physical Component Score	13.3	63.5	39.9 (31.6-50.9)	-10.1	50.0 (SD 1.0)
	SF-12 Mental Component Score	19.1	66.8	51.3 (39.8-57.8)	1.3	50.0 (SD 1.0)
	EQ Index Score	-0.5	1.0	0.69 (0.5-0.8)	-0.17	0.86 (SE 0.004)
	EQ VAS Score	0	100.0	70.0 (50.0-85.0)	-12.8	82.8 (SE 0.4)
	BPI Pain Severity Score	0	9.8	2.5 (0.8-5.0)	-	-
	BPI Pain Interference Score	0	9.9	1.3 (0.3-4.7)	-	-
	BPI Analgesic Effectiveness	0	100.0	50.0 (10.0-80.0)	-	-
	PainDetect Score	0	32.0	6.0 (2.0-12.0)	-	-
	Prop. reporting Thoracic Pain (%)	-	-	79 (54.9)	-	-
	No. affected body regions (21)	0	20.0	2.0 (1.0-3.0)	-	-
6-months	SF-12 Physical Component Score (Mean (SD))	12.3	62.1	42.2 (21.8)	-7.8	50.0 (SD 1.0)
	SF-12 Mental Component Score (Mean (SD))	19.1	65.8	53.7 (16.0)	3.7	50.0 (SD 1.0)
	EQ Index Score	-0.3	1.0	0.65 (0.34-0.77)	-0.21	0.86 (SE 0.004)
	EQ VAS Score	0	100.0	70.0 (34.8-85.0)	-12.8	82.8 (SE 0.4)
	BPI Pain Severity Score	0	10.0	2.0 (0-5.0)	-	-
	BPI Pain Interference Score	0	10.0	1.7 (0-4.9)	-	-
	BPI Analgesic Effectiveness	0	100.0	50.0 (17.5-80.0)	-	-
	PainDetect Score	-1	33	6.0 (1.0-11.0)	-	-
	Prop. reporting Thoracic Pain (%)	-	-	99 (46.9)	-	--
	No. affected body regions (21)	0	21	2.0 (1.0-3.0)	-	

For peer review only

Supplemental File: Table S3: Extended Outcomes of Interest Data/Candidate predictor variables (Pre-injury to three months after discharge)

	Outcome Data:	PCS <35 (Median (IQR))	PCS>35 (Median (IQR))	p-value	PSS ≥3.5 (Median (IQR))	PSS <3.5 (Median (IQR))	p-value	PDQ >12 (Median (IQR))	PDQ <12 (Median (IQR))	p-value
Pre-Injury	PCS	39.0 (31.7-50.0)	52.3 (48.9-55.9)	<0.001*	42.4 (33.1-51.0)	52.1 (45.7-55.6)	<0.001*	43.2 (31.7-51.2)	51.5 (41.8-55.5)	<0.001*
	MCS	51.3 (37.9-57.7)	55.2 (49.1-58.7)	0.006*	51.8 (40.8-57.5)	54.8 (48.5-58.8)	0.022*	48.0 (39.6-57.8)	54.7 (48.1-58.7)	0.019*
	Prop. PCS<20 (n=3) (%)	3 (100)	0	0.022*	3 (100)	0	0.021*	1 (2.2)	2 (1.2)	0.63
	Prop. PCS <30 (n= 22) (%)	17 (77.3)	5 (22.7)	<0.001*	17 (22.4)	5 (3.8)	<0.001*	11 (23.9)	11 (6.7)	0.001*
	Prop. PCS <35 (n=37) (%)	30 (81.1)	7 (18.9)	<0.001*	23 (30.3)	14 (10.5)	<0.001*	16 (34.8)	21 (12.9)	0.001*
Discharge	PCS	23.2 (19.3-27.2)	27.4 (22.2-35.1)	0.001*	24.3 (19.1-30.3)	26.7 (21.6-33.9)	0.02*	24.0 (19.2-29.9)	26.3 (21.3-32.7)	0.041*
	MCS	40.8 (36.5-52.7)	48.5 (39.8-55.8)	0.024*	44.2 (37.6-57.0)	47.2 (37.6-55.3)	0.89	40.9 (36.0-50.3)	47.9 (38.6-55.8)	0.028*
	Prop. PCS<20 (n=54) (%)	28 (37.3))	26 (20.0)	0.007*	27 (36.5)	27 (20.8)	0.014*	19 (40.4)	35 (22.3)	0.013*
	Prop. PCS <30 (n=150) (%)	64 (85.3))	86 (66.2)	0.003*	59 (79.7)	90 (69.2)	0.10	39 (83.0)	110 (70.1)	0.08
	Prop. PCS <35 (n=154) (%)	65 (56.7)	89 (68.5)	0.004*	61 (82.4)	93 (71.5)	0.082	36 (76.6)	118 (75.2)	0.84
	BPI Worst Pain	8.0 (6.0-9.0)	7.5 (5.0-9.0)	0.40	8.0 (6.0-9.0)	7.5 (5.0-9.0)	0.60	8.0 (6.0-9.0)	7.0 (5.0-9.0)	0.35
	BPI Least Pain	3.0 (1.0-5.0)	3.0 (1.0-5.0)	0.42	4.0 (1.0-5.0)	2.0 (1.0-5.0)	0.048*	4.0 (2.0-5.3)	2.0 (1.0-5.0)	0.029*
	BPI Average Pain	5.0 (4.0-7.0)	5.0 (4.0-6.3)	0.22	5.0 (4.0-7.0)	5.0 (4.0-7.0)	0.53	5.5 (4.0-7.0)	5.0 (4.0-6.5)	0.11
	BPI Current Pain	5.0 (3.0-7.0)	4.0 (2.0-6.0)	0.01*	5.0 (3.0-7.0)	4.0 (2.0-6.0)	0.021*	5.0 (4.0-7.0)	4.0 (2.0-6.0)	0.001*
	BPI Pain Severity Score	5.5 (3.9-6.8)	4.8 (3.3-6.0)	0.085	5.3 (3.9-6.5)	4.8 (3.3-6.0)	0.053	5.8 (4.4-6.9)	4.8 (3.3-6.0)	0.009*
	Prop. PSS >3.5 (n=157) (%)	60 (81.1)	97 (74.6)	0.29	59 (80.8)	97 (74.6)	0.31	40 (87.0)	116 (73.9)	0.07
	Analgesic Effectiveness	65.0 (40.0-80.0)	70.0 (50.0-90.0)	0.009*	65.0 (50.0-80.0)	70.0 (50.0-80.0)	0.44	60.0 (50.0-80.0)	70.0 (50.0-80.0)	0.60
	General Activities	8.0 (6.0-10.0)	7.0 (4.5-10.0)	0.005*	8.0 (5.0-10.0)	8.0 (5.0-10.0)	0.38	8.0 (5.0-10.0)	8.0 (5.0-10.0)	0.30
	Mood	6.0 (3.0-8.0)	3.0 (1.0-6.5)	0.002*	5.0 (2.0-7.0)	5.0 (1.0-8.0)	0.65	5.0 (2.8-8.0)	4.0 (1.0-7.0)	0.05
	Walking	8.0 (5.0-10.0)	5.0 (2.0-7.0)	<0.001*	7.0 (4.0-9.0)	5.0 (2.0-8.0)	0.004*	8.0 (4.0-9.3)	5.0 (2.0-8.0)	0.001*
	Normal Work	10.0 (8.0-10.0)	10.0 (5.0-10.0)	0.023*	10.0 (7.0-10.0)	10.0 (5.0-10.0)	0.15	10.0 (7.0-10.0)	10.0 (5.5-10.0)	0.54
	Relations	2.5 (0-7.0)	2.0 (0-4.0)	0.15	2.0 (0-5.0)	2.0 (0-5.0)	0.53	3.0 (0-7.0)	1.0 (0-5.0)	0.021*
	Sleep	7.0 (3.8-8.3)	5.0 (2.0-8.0)	0.15	6.0 (2.0-8.0)	6.0 (3.0-8.0)	0.64	7.0 (5.0-9.0)	5.0 (2.0-8.0)	0.055
	Enjoyment	8.0 (5.0-10.0)	6.5 (3.0-9.0)	0.057	7.0 (3.8-10.0)	7.0 (4.0-10.0)	0.69	8.0 (4.0-10.0)	7.0 (3.5-9.0)	0.24
	BPI Pain Interference Score	6.6 (4.8-8.2)	5.0 (3.5-6.7)	<0.001*	6.4 (4.4-7.5)	5.7 (3.7-7.2)	0.22	6.4 (4.7-8.0)	5.6 (3.7-7.1)	0.016*
	PDQ: Low Risk (n=143) (%)	48 (64.9)	95 (73.1)	0.42	47 (63.5)	96 (74.4)	0.075	24 (52.2)	119 (75.8)	0.006*

For peer review only

	PDQ: Moderate Risk (n=44) (%)	18 (24.3)	26 (20.0)		22 (29.7)	21 (16.3)		17 (37.0)	26 (16.6)	
	PDQ: High Risk (n=17) (%)	8 (10.8)	9 (6.9)		5 (6.8)	12 (9.3)		5 (10.9)	12 (7.6)	
1-month	PCS (Mean (±SD))	27.0 (6.0)	33.5 (7.5)	<0.001*	27.7 (6.0)	32.9 (7.8)	<0.001*	27.8 (5.8)	32.1 (7.9)	<0.001*
	MCS (Mean (±SD))	40.8 (12.2)	46.8 (11.2)	0.001*	40.7 (12.7)	46.7 (10.9)	0.001*	35.6 (12.3)	47.0 (10.6)	<0.001*
	Prop. PCS<20 (n=10) (%)	7 (10.9)	3 (2.7))	0.022*	6 (10.0)	4 (3.4)	0.073	3 (8.1)	7 (5.0)	0.47
	Prop. PCS <30 (n=95) (%)	50 (78.1)	45 (39.8)	<0.001*	42 (70.0)	53 (45.3)	0.002*	26 (70.3)	69 (49.3)	0.023*
	Prop. PCS <35 (n=130) (%)	60 (93.8)	70 (61.9)	<0.001*	52 (86.7)	78 (66.7)	0.004*	33 (89.2)	97 (69.3)	0.015*
	BPI Worst Pain	6.0 (4.0-8.0)	5.0 (3.0-6.0)	0.001*	6.0 (5.0-8.0)	4.0 (3.0-6.0)	<0.001*	7.0 (5.0-8.0)	5.0 (3.0-6.0)	<0.001*
	BPI Least Pain	3.0 (2.0-6.0)	2.0 (1.0-4.0)	0.003*	4.0 (3.0-6.0)	2.0 (0-4.0)	<0.001*	5.0 (3.0-6.0)	2.0 (1.0-4.0)	<0.001*
	BPI Average Pain (Mean (±SD))	4.8 (2.3)	3.5 (2.0)	<0.001*	5.3 (2.0)	3.2 (2.0)	<0.001*	5.7 (2.2)	3.5 (2.0)	<0.001*
	BPI Current Pain	5.0 (3.0-7.0)	2.0 (0.5-4.0)	<0.001*	5.0 (3.0-7.0)	2.0 (0-4.0)	<0.001*	5.5 (3.3-7.8)	2.5 (1.0-4.0)	<0.001*
	BPI Pain Severity Score (Mean (±SD))	4.7 (2.3)	3.2 (1.9)	<0.001*	5.2 (2.0)	3.0 (1.9)	<0.001*	5.5 (2.2)	3.5 (2.0)	<0.001*
	Prop. PSS >3.5 (n=101) (%)	45 (71.4)	56 (49.6)	0.005*	49 (83.1)	52 (44.4)	<0.001*	31 (86.1)	70 (50.0)	<0.001*
	Analgesic Effectiveness	50.0 (30.0-70.0)	50.0 (20.0-70.0)	0.98	50.0 (37.5-60.0)	50.0 (20.0-80.0)	0.86	50.0 (30.0-70.0)	50.0 (25.0-70.0)	0.88
	General Activities	6.0 (5.0-9.0)	4.0 (2.0-6.0)	<0.001*	6.0 (4.0-9.0)	5.0 (2.0-6.0)	<0.001*	7.5 (5.0-9.0)	5.0 (2.0-6.0)	<0.001*
	Mood	5.0 (2.0-7.0)	2.5 (1.0-6.0)	0.006*	6.0 (3.0-7.0)	2.0 (1.0-5.0)	<0.001*	6.0 (3.3-8.0)	3.0 (1.0-5.0)	<0.001*
	Walking	6.0 (3.0-9.0)	2.0 (0.8-5.0)	<0.001*	6.0 (3.0-9.0)	2.0 (0-5.0)	<0.001*	7.0 (3.0-9.0)	3.0 (1.0-6.0)	<0.001*
	Normal Work	7.0 (5.0-10.0)	4.0 (2.0-7.0)	<0.001*	7.0 (5.0-10.0)	4.0 (2.0-7.0)	<0.001*	8.0 (5.0-10.0)	5.0 (2.0-7.0)	<0.001*
	Relations	4.0 (0-7.3)	1.0 (0-4.0)	0.002*	4.0 (1.0-7.0)	1.0 (0-4.0)	<0.001*	6.0 (3.0-8.0)	1.0 (0-4.0)	<0.001*
	Sleep	7.0 (3.0-9.0)	5.0 (2.0-8.0)	0.036*	7.0 (5.0-9.0)	4.0 (2.0-7.5)	<0.001*	8.0 (7.0-9.0)	4.0 (2.0-7.0)	<0.001*
	Enjoyment	7.0 (3.8-9.0)	4.0 (2.0-7.0)	<0.001*	7.0 (4.0-9.0)	4.0 (1.0-7.0)	<0.001*	8.0 (5.3-9.8)	4.0 (2.0-7.0)	<0.001*
	BPI Pain Interference Score (Mean (±SD))	5.6 (2.6)	3.7 (2.5)	<0.001*	5.8 (2.4)	3.6 (2.5)	<0.001*	6.6 (2.4)	3.8 (2.4)	<0.001*
	PDQ: Low Risk (n=122) (%)	39 (61.9)	83 (73.5)	0.14	34 (57.6)	88 (75.2)	0.005*	14 (38.9)	108 (77.1)	<0.001*
	PDQ: Moderate Risk (n=36) (%)	14 (22.2)	22 (19.5)		13 (22.0)	23 (19.7)		9 (25.0)	27 (19.3)	
	PDQ: High Risk (n=18) (%)	10 (15.9)	8 (7.1)		12 (20.3)	6 (5.1)		13 (36.1)	5 (3.6)	
3-months	PCS	31.0 (25.7-35.8)	44.4 (36.7-53.1)	<0.001*	31.5 (26.3-36.0)	43.8 (36.7-53.1)	<0.001*	32.0 (25.7-39.4)	42.1 (33.0-52.4)	<0.001*
	MCS	42.2 (33.7-55.4)	54.2(46.3-57.9)	0.001*	42.2 (34.6-53.6)	54.5 (46.7-58.6)	<0.001*	40.7 (28.6-48.7)	53.6 (45.8-58.0)	<0.001*
	Prop. PCS<20 (n=4) (%)	3 (6.4)	1 (1.1)	0.071	3 (6.4)	1 (1.1)	0.071	1 (3.2)	3 (2.7)	0.88
	Prop. PCS <30 (n=32) (%)	24 (51.1)	8 (8.4)	<0.001*	23 (48.9)	9 (9.5)	<0.001*	14 (45.2)	18 (16.2)	0.001*
	Prop. PCS <35 (n=56) (%)	35 (74.5)	21 (22.1)	<0.001*	34 (74.5)	22 (23.2)	<0.001*	21 (67.7)	35 (31.5)	<0.001*
	BPI Worst Pain (Mean (±SD))	5.6 (2.8)	2.9 (2.5)	<0.001*	6.2 (2.2)	2.6 (2.4)	<0.001*	5.9 (2.2)	3.2 (2.8)	<0.001*

For peer review only

	BPI Least Pain	3.0 (0-6.0)	1.0 (0-2.0)	0.001*	5.0 (2.0-6.0)	0.0 (0-2.0)	<0.001*	4.0 (2.0-6.0)	1.0 (0-2.0)	<0.001*
	BPI Average Pain	5.0 (2.0-7.0)	2.0 (0-4.0)	<0.001*	5.0 (4.0-7.0)	1.0 (0-3.0)	<0.001*	5.0 (3.0-7.0)	2.0 (1.0-4.0)	<0.001*
	BPI Current Pain	4.0 (1.0-7.3)	1.0 (0-3.0)	<0.001*	5.0 (3.0-7.0)	0.0 (0-2.0)	<0.001*	5.0 (2.0-6.0)	1.0 (0-3.0)	<0.001*
	BPI Pain Severity Score	4.5 (2.5-6.6)	1.3 (0.5-3.5)	<0.001*	5.0 (3.3-6.5)	1.0 (0.3-2.8)	<0.001*	5.0 (3.3-6.3)	1.5 (0.5-3.5)	<0.001*
	Prop. PSS >3.5 (n=53) (%)	27 (58.7)	26 (27.4)	<0.001*	35 (74.5)	18 (19.1)	<0.001*	23 (74.2)	30 (27.3)	<0.001*
	Analgesic Effectiveness	35.0 (10.0-70.0)	50.0 (0-90.0)	0.19	50.0 (20.0-70.0)	35.0 (0-90.0)	0.55	50.0 (27.5-70.0)	50.0 (0-80.0)	0.66
	General Activities	5.0 (2.0-8.3)	2.0 (0-5.0)	<0.001*	6.0 (4.0-8.0)	1.5 (0-4.0)	<0.001*	5.0 (4.0-8.3)	2.0 (0-5.0)	<0.001*
	Mood	4.0 (1.0-7.3)	1.0 (0-4.0)	<0.001*	4.0 (2.0-7.0)	0.5 (0-3.3)	<0.001*	5.0 (3.0-8.0)	1.0 (0-4.0)	<0.001*
	Walking	5.0 (2.0-9.3)	1.0 (0-3.0)	<0.001*	4.0 (2.0-8.0)	0 (0-3.0)	<0.001*	4.0 (1.8-8.0)	2.0 (0-5.0)	<0.001*
	Normal Work	5.5 (2.0-9.0)	1.0 (0-4.0)	<0.001*	5.0 (3.0-9.0)	0 (0-2.0)	<0.001*	5.0 (4.0-9.0)	1.0 (0-4.0)	<0.001*
	Relations	1.0 (0-4.3)	0 (0-1.0)	0.002*	3.0 (0-5.0)	0 (0-1.0)	<0.001*	4.0 (1.0-6.0)	0 (0-1.0)	<0.001*
	Sleep	4.0 (0.8-8.0)	1.0 (0-3.0)	0.001*	5.0 (2.0-8.0)	1.0 (0-3.0)	<0.001*	5.0 (2.0-8.0)	1.0 (0-3.3)	<0.001*
	Enjoyment	5.0 (1.0-8.3)	1.0 (0-3.0)	<0.001*	6.0 (4.0-9.0)	1.0 (0-2.0)	<0.001*	5.0 (2.0-9.0)	1.0 (0-4.3)	<0.001*
	BPI Pain Interference Score	4.7 (1.3-7.2)	0.9 (0.1-3.1)	<0.001*	5.0 (3.0-7.0)	0.6 (0.1-2.0)	<0.001*	4.7 (3.6-7.1)	0.9 (0.1-3.2)	<0.001*
	PDQ: Low Risk (n=107) (%)	28 (60.9)	79 (83.2)	0.012*	23 (48.9)	84 (89.4)	<0.001*	9 (29.0)	98 (89.1)	<0.001*
	PDQ: Moderate Risk (n=15) (%)	7 (15.2)	8 (8.4)		9 (19.1)	6 (6.4)		9 (29.0)	6 (5.5)	
	PDQ: High Risk (n=19) (%)	11 (23.9)	8 (8.4)		15 (31.9)	4 (4.3)		13 (41.9)	6 (5.5)	

For peer review only

STROBE Statement—checklist of items that should be included in reports of observational studies

	Item No.	Recommendation	Page No.
**Title and abstract**	1	(*a*) Indicate the study's design with a commonly used term in the title or the abstract	1
		(*b*) Provide in the abstract an informative and balanced summary of what was done and what was found	1
**Introduction**			
Background/rationale	2	Explain the scientific background and rationale for the investigation being reported	2
Objectives	3	State specific objectives, including any prespecified hypotheses	3
**Methods**			
Study design	4	Present key elements of study design early in the paper	3
Setting	5	Describe the setting, locations, and relevant dates, including periods of recruitment, exposure, follow-up, and data collection	3
Participants	6	(*a*) *Cohort study*—Give the eligibility criteria, and the sources and methods of selection of participants. Describe methods of follow-up<br>*Case-control study*—Give the eligibility criteria, and the sources and methods of case ascertainment and control selection. Give the rationale for the choice of cases and controls<br>*Cross-sectional study*—Give the eligibility criteria, and the sources and methods of selection of participants	3 &4
		(*b*) *Cohort study*—For matched studies, give matching criteria and number of exposed and unexposed<br>*Case-control study*—For matched studies, give matching criteria and the number of controls per case	N/A
Variables	7	Clearly define all outcomes, exposures, predictors, potential confounders, and effect modifiers. Give diagnostic criteria, if applicable	5
Data sources/ measurement	8*	For each variable of interest, give sources of data and details of methods of assessment (measurement). Describe comparability of assessment methods if there is more than one group	5
Bias	9	Describe any efforts to address potential sources of bias	4-6
Study size	10	Explain how the study size was arrived at	4

Continued on next page

For peer review only - http://bmjopen.bmj.com/site/about/guidelines.xhtml

For peer review only

Quantitative variables	11	Explain how quantitative variables were handled in the analyses. If applicable, describe which groupings were chosen and why	6
Statistical methods	12	(*a*) Describe all statistical methods, including those used to control for confounding	6
		(*b*) Describe any methods used to examine subgroups and interactions	6
		(*c*) Explain how missing data were addressed	6
		(*d*) *Cohort study*—If applicable, explain how loss to follow-up was addressed<br>*Case-control study*—If applicable, explain how matching of cases and controls was addressed<br>*Cross-sectional study*—If applicable, describe analytical methods taking account of sampling strategy	6
		(*e*) Describe any sensitivity analyses	-
**Results**			
Participants	13*	(a) Report numbers of individuals at each stage of study—eg numbers potentially eligible, examined for eligibility, confirmed eligible, included in the study, completing follow-up, and analysed	7
		(b) Give reasons for non-participation at each stage	7
		(c) Consider use of a flow diagram	-
Descriptive data	14*	(a) Give characteristics of study participants (eg demographic, clinical, social) and information on exposures and potential confounders	7
		(b) Indicate number of participants with missing data for each variable of interest	7
		(c) *Cohort study*—Summarise follow-up time (eg, average and total amount)	7
Outcome data	15*	*Cohort study*—Report numbers of outcome events or summary measures over time	9
		Case-control study—Report numbers in each exposure category, or summary measures of exposure	-
		Cross-sectional study—Report numbers of outcome events or summary measures	-
Main results	16	(*a*) Give unadjusted estimates and, if applicable, confounder-adjusted estimates and their precision (eg, 95% confidence interval). Make clear which confounders were adjusted for and why they were included	8-10
		(*b*) Report category boundaries when continuous variables were categorized	8-10
		(*c*) If relevant, consider translating estimates of relative risk into absolute risk for a meaningful time period	N/A

Continued on next page

For peer review only - http://bmjopen.bmj.com/site/about/guidelines.xhtml
For peer review only

Other analyses	17	Report other analyses done—eg analyses of subgroups and interactions, and sensitivity analyses	8-10
**Discussion**			
Key results	18	Summarise key results with reference to study objectives	-
Limitations	19	Discuss limitations of the study, taking into account sources of potential bias or imprecision. Discuss both direction and magnitude of any potential bias	10-12
Interpretation	20	Give a cautious overall interpretation of results considering objectives, limitations, multiplicity of analyses, results from similar studies, and other relevant evidence	10-12
Generalisability	21	Discuss the generalisability (external validity) of the study results	10-12
**Other information**			
Funding	22	Give the source of funding and the role of the funders for the present study and, if applicable, for the original study on which the present article is based	Declarations section

*Give information separately for cases and controls in case-control studies and, if applicable, for exposed and unexposed groups in cohort and cross-sectional studies.

Note: An Explanation and Elaboration article discusses each checklist item and gives methodological background and published examples of transparent reporting. The STROBE checklist is best used in conjunction with this article (freely available on the Web sites of PLoS Medicine at http://www.plosmedicine.org/, Annals of Internal Medicine at http://www.annals.org/, and Epidemiology at http://www.epidem.com/). Information on the STROBE Initiative is available at www.strobe-statement.org.

For peer review only

BMJ Open

A prospective observational study to examine health related quality of life and develop models to predict long-term patient reported outcomes six-months after hospital discharge with blunt thoracic injuries.

Journal:	*BMJ Open*
Manuscript ID	bmjopen-2021-049292.R1
Article Type:	Original research
Date Submitted by the Author:	02-May-2021
Complete List of Authors:	Baker, Edward; King's College London, Florence Nightingale Faculty of Nursing, Midwifery and Palliative Care; King's College Hospital NHS Foundation Trust, Emergency Department<br>Battle, Ceri; Swansea Bay University Health Board, Welsh Institute of Biomedical and Emergency Medicine Research<br>Banjeri, Abhishek; Buckingham Healthcare NHS Trust<br>Carlton, Edward; North Bristol NHS Trust, Emergency Department<br>Dixon, Christine; Surrey and Sussex Healthcare NHS Trust, Emergency Department<br>Ferry, Jennifer; Aneurin Bevan Health Board, Department of Anesthetics<br>Hopkins, Philip; King's College Hospital NHS Foundation Trust, Critical Care<br>Jones, Robert; Barnsley Hospital NHS Foundation Trust<br>Murrells, Trevor; King's College London, Florence Nightingale Faculty of Nursing, Midwifery and Palliative Care<br>Norton, Christine; King's College London, Florence Nightingale Faculty of Nursing & Midwifery<br>Patient, Lee; St George's Healthcare NHS Trust, Emergency Department<br>Rasheed, Ashraf; Aneurin Bevan Health Board, General Surgery<br>Skene, Imogen; UCL, Primary care and population health; Barts Health NHS Trust, Emergency Department<br>Tabner, Andrew; University Hospitals of Derby and Burton NHS Foundation Trust<br>Tunnicliff, Malcolm; King's College Hospital NHS Foundation Trust, Emergency Department<br>Young, Louise; Imperial College Healthcare NHS Trust, Emergency Department<br>Xyrichis, Andreas; King's College London, Florence Nightingale Faculty of Nursing, Midwifery and Palliative Care<br>Lee, Gerry; King's College London, Florence Nightingale Faculty of Nursing, Midwifery and Palliative Care
**Primary Subject Heading**:	Emergency medicine
Secondary Subject Heading:	Emergency medicine
Keywords:	ACCIDENT & EMERGENCY MEDICINE, Risk management < HEALTH

	SERVICES ADMINISTRATION & MANAGEMENT, Trauma management < ORTHOPAEDIC & TRAUMA SURGERY, PAIN MANAGEMENT, Cardiothoracic surgery < SURGERY

SCHOLARONE™ Manuscripts

I, the Submitting Author has the right to grant and does grant on behalf of all authors of the Work (as defined in the below author licence), an exclusive licence and/or a non-exclusive licence for contributions from authors who are: i) UK Crown employees; ii) where BMJ has agreed a CC-BY licence shall apply, and/or iii) in accordance with the terms applicable for US Federal Government officers or employees acting as part of their official duties; on a worldwide, perpetual, irrevocable, royalty-free basis to BMJ Publishing Group Ltd ("BMJ") its licensees and where the relevant Journal is co-owned by BMJ to the co-owners of the Journal, to publish the Work in this journal and any other BMJ products and to exploit all rights, as set out in our licence.

The Submitting Author accepts and understands that any supply made under these terms is made by BMJ to the Submitting Author unless you are acting as an employee on behalf of your employer or a postgraduate student of an affiliated institution which is paying any applicable article publishing charge ("APC") for Open Access articles. Where the Submitting Author wishes to make the Work available on an Open Access basis (and intends to pay the relevant APC), the terms of reuse of such Open Access shall be governed by a Creative Commons licence – details of these licences and which Creative Commons licence will apply to this Work are set out in our licence referred to above.

Other than as permitted in any relevant BMJ Author's Self Archiving Policies, I confirm this Work has not been accepted for publication elsewhere, is not being considered for publication elsewhere and does not duplicate material already published. I confirm all authors consent to publication of this Work and authorise the granting of this licence.

For peer review only

A prospective observational study to examine health related quality of life and develop models to predict long-term patient reported outcomes six-months after hospital discharge with blunt thoracic injuries.

Mr. Edward BAKER (Corresponding Author)
NIHR Clinical Doctoral Research Fellow - Kings College London
edward.e.baker@kcl.ac.uk
Florence Nightingale Faculty of Nursing, Midwifery and Palliative Care
King's College London
James Clerk Maxwell Building
57 Waterloo Road
London
SE1 8WA

Dr Ceri Battle
Consultant Physiotherapist - Swansea Bay University Health Board
ceri.battle@wales.nhs.uk

Dr Abhishek Banjeri
Emergency Medicine Consultant - Buckingham Healthcare NHS Trust
abhishekbanerji@nhs.net

Dr Edward Carlton
Emergency Medicine Consultant – North Bristol NHS Trust
eddcarlton@gmail.com

Ms. Christin Dixon
Trauma Nursing Lead – Surrey and Sussex Healthcare NHS Trust
christine.dixon14@nhs.net

Dr Jennifer Ferry
Consultant Anaesthetist – Aneurin Bevan University Health Board
jennifer.ferry@wales.nhs.uk

Dr Philip Hopkins
Intensive Care Consultant and ICM Research Lead – Kings College Hospital NHS Foundation Trust
p.hopkins@nhs.net

Dr Robert Jones
Emergency Medicine Consultant – Barnsley Hospital NHS Foundation Trust
robertjones5@nhs.net

Mr. Trevor Murrells
Faculty Statistician – King's College London
trevor.murrells@kcl.ac.uk

Professor Christine Norton
Professor of Nursing Research – King's College London

For peer review only

christine.norton@kcl.ac.uk

Mr. Lee Patient
Nurse Consultant Emergency Medicine – St Georges University Hospital NHS Foundation Trust
lee.patient@stgeorges.nhs.uk

Professor Ashraf Rasheed
Professor of General Surgery – Aneurin Bevan University Health Board
ashraf.rasheed@wales.nhs.uk

Ms. Imogen Skene
Lead Research Nurse Emergency Medicine – Barts Health NHS Trust
i.skene@nhs.net

Dr Andrew Tabner
Emergency Medicine Consultant – University Hospitals of Derby and Burton NHS Foundation Trust
andrew.tabner@nhs.net

Dr Malcolm Tunnicliff
Emergency Medicine Consultant – King's College Hospital NHS Foundation Trusts
malcolm.tunnicliff@nhs.net

Ms. Louise Young
Lead Research Nurse Emergency Medicine – Imperial College Healthcare NHS Trust
louise.young10@nhs.net

Dr Andreas Xyrichis
Senior Lecturer in Nursing – King's College London
andreas.xyrichis@kcl.ac.uk

Dr Gerry Lee
Reader in Advanced Clinical Practice – King's College London
gerry.lee@kcl.ac.uk
Florence Nightingale Faculty of Nursing, Midwifery and Palliative Care
King's College London
James Clerk Maxwell Building
57 Waterloo Road
London
SE1 8WA

For peer review only

For peer review only - http://bmjopen.bmj.com/site/about/guidelines.xhtml

Abstract:

Objective: This study aimed to examine the long-term outcomes and health-related quality of life in patients with blunt thoracic injuries over six-months from hospital discharge and develop models to predict long-term patient reported outcomes.

Design: A prospective observational study using longitudinal survey design.

Setting: The study recruitment was undertaken at 12 UK hospitals which represented diverse geographical locations and covered urban, sub-urban and rural areas across England and Wales.

Participants: 377 patients admitted to hospital with blunt thoracic injuries were recruited between June 2018-October 2020.

Methods: Participants completed a bank of 2 quality of life surveys (Short Form-12 and EuroQol-5d-5l) and 2 pain questionnaires (Brief Pain Inventory and PainDetect questionnaire) at 4 time-points over the first 6-months after discharge from hospital. A total of 211 (63%) participants completed the outcomes data at 6-months after hospital discharge.

Outcomes Measures: Three outcomes were measured using pre-existing and validated patient reported outcome measures. Outcomes included: Poor Physical function (SF-12 Physical Component Score); Chronic pain (Brief Pain Inventory Pain Severity Score); and Neuropathic pain (PainDetect questionnaire).

Results: Despite a trend towards improving physical functional and pain at 6-months, outcomes did not return to participants perceived baseline level of function. At six-months after hospital discharge, 37% (n=77) of participants reported poor physical function; 36.5% (n=77) reported a chronic pain state; and 22% (n=47) reported pain with a neuropathic component. Predictive models were developed for each outcome highlighting important data collection requirements for predicting long-term outcomes in this population. Model diagnostics including calibration and discrimination statistics suggested good model fit in this development cohort.

Conclusions: This study identified the recovery trajectories for patients with blunt thoracic injuries over the first six-months after hospital discharge and present prognostic models for three important outcomes which after external validation could be used as clinical risk stratification scores.

Keywords: Trauma; Chest Injury; Rib Fractures; Predictive modelling; Long-term outcomes; Pain; Neuropathic Pain; Physical Functioning; Quality of Life; Patient Reported Outcome Measures.

For peer review only - http://bmjopen.bmj.com/site/about/guidelines.xhtml

For peer review only

For peer review only - http://bmjopen.bmj.com/site/about/guidelines.xhtml

Strengths and Limitations of the Study:

- This study highlights the physical and pain sequelae associated with recovery after blunt thoracic injuries and provides insights into the recovery trajectory for this population.
- Data are derived from geographically diverse trauma hospital as across England and Wales.
- The longitudinal study design using patient reported outcome measures demonstrates the importance of eliciting health data from this population over throughout the post discharge recovery period.
- Patient reported outcome measures are valuable in outcome prediction, particularly where usual care does not include follow-up after hospital discharge.
- Despite the benefits of patient reported outcome measures, it is important to acknowledge the potential inherent bias within these measures.

For peer review only

A prospective observational study to examine health related quality of life and develop models to predict long-term patient reported outcomes six-months after hospital discharge with blunt thoracic injuries.

Background:

Blunt thoracic injury (BTI) is a term that covers a spectrum of different injury patterns and severities across all adult age groups.[1] It accounts for about 15% of injured patients presenting to trauma hospitals, with mortality rates reported between 4-20%.[2] Outcomes within trauma care have historically been measured primarily using mortality as a key outcome.[3] During the last two decades, there has been a decrease in the mortality rates of polytrauma patients in high income countries with organised trauma systems.[4] As the mortality rates have decreased, there has been a growing focus on measuring outcomes relating to injury morbidity.[3 5] There is a growing body of international research investigating the long-term outcomes of patients with BTI, but within the United Kingdom (UK), there is a paucity of BTI outcomes research with little consistency in the methods employed.[6 7]

For patients recovering from BTI, it has been identified that reduced physical function, persistent altered respiratory function, and chronic pain frequently impact on all aspects of daily living.[6] Health Related Quality of Life (HRQoL) provides a strong foundation for measuring the impact of these symptoms with a holistic and comprehensive approach.[8] It is important not to isolate individual components of daily living but consider them as a single entity, and HRQoL measurement provides a vehicle to do this.[9] Furthermore, it allows the development of theory driven interventions that impact on all elements of daily living.[10] For the purposes of this study HRQOL is defined as including all aspects of self-perceived well-being that are related to, or affected by, the presence of disease or treatment.[11]

Follow-up after hospital discharge with major trauma is often limited within the UK's National Health Service (NHS), resulting in limited clinical knowledge about the recovery journey of BTI patients.[12] This also leaves clinicians without a trajectory for recovery making it very difficult to adequately educate and prepare patients for self-management in the post hospital discharge recovery period.[13] Evidence from this study will be used to facilitate the identification of patients at risk of poor outcomes after BTI, help develop health promotion materials, and to support the development of follow-up interventions within BTI management.[13]

Having the ability to predict outcomes and develop methods for risk stratification is extremely important in trauma care.[14] There is a growing body of prognostic research in this area with simple

For peer review only

scoring systems developed and validated with effective predictive abilities across different health systems.[2] Where resources for patient follow-up are limited, it is key that effective methods for identifying patients in high-risk groups for complications or poor outcomes are in place, allowing resources to be focused on these patients at the greatest risk. The aim of this study was to examine the long-term outcomes and HRQoL in patients with BTI over six-months from hospital discharge and develop models to predict long-term patient reported outcomes. Study objectives were as follows:

- Investigate changes in HRQoL over 6 months after hospital discharge with BTI.
- Examine the evolution of pain and neuropathic pain over six months after discharge from hospital with BTI.
- Develop predictive models for poor physical functioning, chronic pain, and neuropathic pain at six-months after hospital discharge with BTI.

Methods:

This prospective multicentred observational study employed longitudinal survey methods to follow-up patients recovering from BTI over a six-month period from hospital discharge. This research forms the quantitative component of a mixed methods study investigating recovery after BTI. The qualitative components will be reported separately.

Study Setting:

The study population sample was recruited from Major Trauma Centres (MTC) (Level 1) (n=5) and Trauma Units (Level 2-3) (n=7) from diverse geographical locations within urban, sub-urban and rural areas across England and Wales. Sites were set up in a stepwise fashion and in total 12 acute hospital sites recruited participants between 6 to 10 months in two separate recruitment cohorts between June 2018 and March 2020.

Eligibility Criteria:

Inclusion criteria:

- Participant is willing and able to give informed consent for participation in the study
- Male or female, aged 16 years or above
- Admitted to a Major Trauma Centre/Trauma Unit with primary BTI
- Admitted to hospital for a period of 24 hours or greater

For peer review only - http://bmjopen.bmj.com/site/about/guidelines.xhtml

Exclusion criteria:

- Unstable spinal fracture or spinal cord injury (SCI)
- Traumatic brain injury (TBI) with cognitive impairment
- Altered mental status that would prevent accurate self-assessment through questionnaires or prevent informed consent into study
- Associated unstable pelvic fracture or open abdomen requiring extended periods of treatment in hospital
- Limb injuries from same traumatic event that require surgical intervention
- Participant is a prisoner / in police custody at time of recruitment

Data Collection:

Data collection was undertaken at four timepoints: (i) Hospital discharge (including a pre-injury estimation of HRQoL); (ii) 1 month after discharge; (iii) 3 months after discharge; and (iv) 6 months after discharge. Potential participants were identified by the local clinical team or the clinical research nurses at each site. Demographic and injury related data were collated throughout the acute admission using a previously tested case report form,[1] and site staff conducted data collection at hospital discharge. Post discharge follow-up was conducted by the central research team at the host university using either postal surveys or electronic surveys hosted by SurveyMonkey. During post discharge follow-up non-responding particiapnts were contacted up to three time using a mixed multi-media approach including telephone, post, or email, to maximise participation and minimise the potential for missing data.

Ethics Considerations:

Formal informed written consent was taken from all participants after information sheets had been provided and sufficient time was allowed for discussion with family and clinical research staff. Ethical approval was granted by the 'Hampshire A' South Central Research Ethics Committee in June 2018 (ref: 18/SC/0230). Participants were informed that everyone who completed data collection at all timepoints would be placed in a prize draw to win a £50 shopping voucher to recognise the time associated with participation in this longitudinal follow-up study.

Sample Size:

This observational study had an exploratory design and pragmatic approach to sample size. Although there is no consensus on the sample requirements for logistic regression analysis, based on previous

For peer review only - http://bmjopen.bmj.com/site/about/guidelines.xhtml

studies we aimed to have 10 events for each predictor variables included in the model.[2 3] As it was likely that 10 predictor variables would be used in the modelling process for each model, the aim was to achieve 100 events (i.e., outcomes of interest) in the sample.

Outcome Measures:

HRQoL was explored firstly at a global level through the Physical Component Score (PCS) and Mental Component Score (MCS) of the Short Form-12 (SF-12),[15] and secondly, using the individual levels of the EuroQol 5-dimensions, 5-Levels (EQ5D-5L).[16 17] Pain was previously recognised as an ongoing problem throughout post hospital discharge recovery.[6] In this study, pain severity and impact were measured using the Brief Pain Inventory[18] and neuropathic pain was assessed using the PainDetect Score[19] Using existing literature on HRQoL after BTI,[6 20] 3 key outcomes were selected:

1. **Physical Function** is an individual's ability to undertake actions that involve physical activities, ranging from activities of daily living (ADLs) to more complex activities that involve a combination of skills, often within a social context.[3] For the purposes of this study, physical functioning was measured using the PCS of the SF-12 at six-months after discharge from hospital. A PCS score of ≤35 (Range: 0-100 with higher scores equalling better Physical function) was taken as defining poor physical functioning from previous literature.[15]
2. **Chronic Pain** is defined as pain that persists beyond the normal expected healing time and therefore lacks the acute warning function of physiological nociception. Pain is normally considered as chronic when it lasts or recurs for more than three to 6-months.[21] For this study, chronic pain was defined as having a Brief Pain Inventory (BPI) Pain Severity Score (PSS) of ≥3.5 (Range: 0-10 and higher scores equals increased pain severity) at 6-months after hospital discharge.[22]
3. **Neuropathic pain** is defined as pain caused by a lesion or disease of the somatosensory nervous system.[23] For the purposes of this study, participants with a potential neuropathic pain component were identified using the PainDetect Questionnaire (PDQ).[19] A PainDetect score ≥12 (Range: -1 – 38) at 6-months after hospital discharge defined our cohort of patients with a potential neuropathic pain component.[24]

Although the limitations of using binary outcome measures were acknowledged in the planning phase, this was an important component in the process of developing prognostic models which could be used as risk stratification scores in the clinical practice setting.

For peer review only - http://bmjopen.bmj.com/site/about/guidelines.xhtml

Selection of candidate predictor variables:

A collection of predictor variables was required which covered multiple potentially influential factors relating to the outcomes of interest. Initially, during the study development, a long list of candidate variables were selected from previous literature.[6 10 25 26] From this long-list, shortlists of variables for each outcome were compiled. Decision-making for entering variables into the modelling process was based on their clinical significance and achieving statistical significance at the 5% level in the univariate analysis phase. There was an 'a priori' plan for candidate variables to be selected in 3 phases, initially using only variables that are readily available in clinical practice, secondly, variables that are currently not readily available in clinical practice, and finally using variables collected at post discharge timepoints.

Statistical analysis:

Data analysis was undertaken using SPSS v.26. Statistical data were presented using descriptive and inferential statistical methods and reported according to the Strengthening the Reporting of Observational Studies in Epidemiology (STROBE) statement.[27] Binary and categorical data were presented as percentages and number of cases. Continuous data were presented as means with standard deviations. The statistical tests applied for binary/categorical data were Chi-square test of association, except where the expected cell counts were ≤5 when the Fisher's exact test was used. Comparisons continuous data from two independent groups was undertaken using the Student t-test. Longitudinal changes in continuous variables over the course of the post discharge follow-up period are reported at a group level. This uses the longitudinal change in the group mean from one timepoint to the next to measure change.

Missing data was addressed using the multiple imputation procedure in SPSS. Most missing data were found within the follow-up phase of data collection where units of data (i.e., surveys) were not returned. The imputation model included all predictor variables with potential for inclusion in the modelling process including candidate predictor variables from within the hospital admission and from the post discharge follow-up. A total of 10 imputed datasets were created. Longitudinal imputation of outcome variables was considered but not conducted in this study due to the high level of missingness at both 1-month and 3 months and the low number of particiapnts who completed the follow-up data collection at all timepoints. Ultimately despite the potential limitations, a case wise approach was used.

The predictive model framework was developed in accordance with the TRIPOD (Transparent Reporting of multivariable predication model for Individual Prognosis Or Diagnosis) methodology[28]

For peer review only - http://bmjopen.bmj.com/site/about/guidelines.xhtml

and the study methods based on previously published prognostic modelling studies.[2 3 29] A multivariable logistic regression analysis was used to evaluate the association of the predictors with the outcome (dependent variable) in each imputed data set using the forward step wise method.[30] In this process, SPSS automatically produced a final model that aggregated the results across all the imputed datasets.

Calibration was evaluated using a Hosmer-Lemeshow test where a p-value >0.05 was considered to represent good calibration. In addition, graphical calibration plots were developed. A receiver operating characteristic (ROC) curve was plotted for each model. A c-statistic (area under the receiver operator curve) was calculated, that tested whether the amount of discrimination was significantly different from random (c-statistic of 0.5 indicates that discrimination is no better than the toss of a coin). Performance of the final model was assessed using sensitivity and specificity.[31] Throughout statistical significance was recognised at the 5% level (p<0.05).

Patient and Public involvement:

A patient and public reference group was formed and involved in the development phases of this study including during protocol development and application for ethical approval but have not been involved in data collection, analysis, or interpretation.

Results:

A total of 337 patients were recruited to the study. The median age of participants was 63 years (IQR: 51.0-75.0) and 69% (n=231) were male. The predominant mechanism of injury was falls <2m (45%, n=153) and the median number of rib fractures was 6.0 (3.0-7.0). At six-months after discharge, 37% (n=77) of the sample self-reported poor physical functioning, 37% (n=77) reported chronic pain, and 22% (n=47) reported pain with a likely neuropathic pain component. **Table 1** summarises key demographic data for the total sample. Further greater detailed demographic data is presented in **Table S1** as a supplementary file to this study.

Missing Data Analysis:

A total of 337 patients were recruited to the study. Overall, the combined response rate at all timepoints was 72%. At hospital discharge 95% (n=320) completed the discharge data collection timepoint, subsequently, 62% (n=210) and 43% (n=144) completed data collection at 1-month and 3-month after discharge respectively. At 6-months, 63% (211) of the total sample completed follow-up where the study outcomes data were collected. Of the 211 participants who completed the 6-month

For peer review only - http://bmjopen.bmj.com/site/about/guidelines.xhtml

follow-up, n=200 (94%) completed discharge data collection, 174 (82%) completed the 1-month data collection timepoint, and 142 (67%) completed data collection at 3-months after discharge.

Overall missingness within the dataset was predominately from 'unit missing data' (i.e., completed survey not returned by participant) as individual items of missing data within a returned survey were followed up with participants at the time of data collection. Amongst candidate predictor variables 17% (n=37) were missing at 1-month follow-up, and 33% (n=69) at 3 months. Although this represents high levels of missingness within the dataset, this is not uncommon in longitudinal follow-up studies after traumatic injuries (Baker, Xyrichis, Norton, Hopkins, & Lee, 2018).

A missing data analysis was undertaken as part of the analysis process to identify any differences in the characteristics of participants who did or did not respond to follow-up at six months after hospital discharge when outcome data were collected. **Supplementary table S2** presents the results from this analysis. Although there was a statistically significant difference in participants age, incidence of cardiac disease and cancer, and MCS at the pre-injury, discharge and one-month post discharge timepoint, these were not deemed to be clinically significant differences. Furthermore, a logistic regression model was built using a stepwise forward approach using variables identified in the univariable analysis of missingness to identify predictors of responder status at six months after discharge. It was not possible to fit a model using the selected variables and no statistically significant predictors were identified. Since none were identified, it was assumed that the two groups were characteristically similar and therefore a sensitivity analysis, using weights calculated from the logistic regression model, was not undertaken. Based on Little and Rubin's taxonomy missingness was assumed to be Missing at Random (MAR) at the unit level and multiple imputation methods in SPSS were used to address item level missingness (Ayilara et al., 2019; Schlomer, Bauman, & Card, 2010).

For peer review only - http://bmjopen.bmj.com/site/about/guidelines.xhtml

Table 1: Demographics of entire cohort and a comparison of characteristics for each outcome of interest at six-months after discharge

Result: n= (%) unless otherwise indicated	Total sample (n=337)	**Poor Physical Function** (n=77)	**Good Physical Function** (n=134)	p-value	**Chronic Pain** (n=77)	**No Chronic Pain** (n=134)	p-value	**Neuropathic component likely** (n=47)	**Neuropathic component unlikely** (n=164)	p-value
Age (Mean (SD))	62.0 (16.5)	69.1 (13.9)	60.8 (16.3)	<0.001	66.7 (16.4)	62.1 (15.6)	0.05	63.4 (17.0)	63.9 (15.7)	0.84
Male (%)	231 (68.5)	49 (63.6)	97 (72.4)	0.19	54 (70.1)	91 (67.9)	0.80	33 (70.2)	112 (68.7)	0.85
Injury Severity Score (Mean (SD))	11.2 (7.3)	10.9 (7.8)	11.4 (6.8)	0.61	11.3 (7.9)	11.2 (6.8)	0.89	11.6 (8.3)	11.2 (6.9)	0.76
**Mechanism of Injury:**										
RTC	78 (23.1)	14 (18.2)	38 (28.4)		19 (24.7)	33 (24.6)		9 (19.1)	43 (26.4)	
Crush	11 (3.3)	3 (3.9)	4 (3.0)		1 (1.3)	6 (4.5)		1 (2.1)	6 (3.7)	
Ped vs. Vehicle	4 (1.2)	1 (1.3)	2 (1.5)		1 (1.3)	2 (1.5)		1 (2.1)	2 (1.2)	
Fall>2m	69 (20.5)	13 (16.9)	30 (22.4)	0.23	18 (23.4)	25 (18.7)	0.78	13 (27.7)	30 (18.4)	0.82
Fall<2m	153 (45.4)	44 (57.1)	54 (40.3)		37 (48.1)	61 (45.5)		22 (46.8)	76 (46.6)	
Assault	14 (4.2)	1 (1.3)	4 (3.0)		1 (1.3)	3 (2.2)		1 (2.1)	3 (1.8)	
CPR	4 (1.2)	1 (1.3)	0		0	1 (0.7)		0	1 (0.6)	
Rapid deceleration	4 (1.2)	0	2 (1.5)		0	2 (1.5)		0	2 (1.2)	
**Co-morbidities:**										
Respiratory co-morbidity	70 (20.8)	21 (27.3)	21 (15.7)	0.042	21 (27.3)	20 (14.9)	0.031	12 (25.5)	29 (17.8)	0.24
Cardiac co-morbidity	147 (43.6)	49 (63.6)	53 (39.6)	0.001	47 (61.0)	54 (40.3)	0.004	27 (57.4)	74 (45.4)	0.15
Neuro co-morbidity	50 (14.8)	20 (26.0)	9 (6.7)	<0.001	19 (24.7)	10 (7.5)	0.001	10 (21.3)	19 (11.7)	0.092
MSK co-morbidity	89 (26.4)	26 (33.8)	35 (26.1)	0.24	30 (39.0)	31 (23.1)	0.016	16 (34.0)	45 (27.6)	0.39
Pre-Injury regular analgesic use	61 (18.1)	23 (29.9)	15 (11.2)	0.001	30 (39.0)	8 (6.0)	<0.001	19 (40.4)	19 (11.7)	<0.001
**Injury Descriptors:**										
No. Rib Fractures (Mean (SD))	5.2 (4.0)	5.3 (3.8)	5.3 (4.0)	0.90	5.4 (3.9)	5.3 (3.9)	0.74	5.6 (4.1)	5.2 (3.8)	0.55
Operative Rib Fixation	16 (4.7)	6 (7.8)	3 (2.2)	0.05	2 (2.6)	7 (5.2)	0.36	3 (6.4)	6 (3.7)	0.42
First Rib fracture	31 (9.2)	6 (7.8)	13 (9.7)	0.64	10 (13.0)	9 (6.7)	0.13	7 (14.9)	12 (7.4)	0.11
Bilateral rib fractures	44 (13.1)	9 (11.7)	19 (14.2)	0.61	11 (14.3)	17 (12.7)	0.76	10 (21.3)	18 (11.0)	0.07
Radiological Flail	60 (17.8)	13 (16.9)	29 (21.6)	0.41	16 (20.8)	26 (19.4)	0.83	7 (14.9)	35 (21.5)	0.32
Extra-thoracic injuries	104 (30.9)	25 (32.5)	35 (26.1)	0.33	19 (24.7)	40 (29.9)	0.40	12 (25.5)	47 (28.8)	0.66
**Analgesic mode:**										

For peer review only

Intermittent analgesia only	179 (53.1)	37 (48.1)	77 (57.5)	0.19	37 (48.1)	76 (56.7)	0.20	22 (46.8)	91 (55.8)	0.28
Regional Analgesia	106 (31.5)	29 (37.7)	41 (30.6)	0.29	31 (40.3)	39 (29.1)	0.11	20 (42.6)	50 (30.7)	0.13
Patient Controlled Analgesic only	93 (27.6)	23 (29.9)	29 (21.6)	0.18	20 (26.0)	32 (23.9)	0.76	12 (25.5)	40 (24.5)	0.89
**Short term inpatient outcomes:**										
Complications:	111 (32.9)	34 (44.2)	33 (24.6)	0.003	25 (32.5)	42 (31.3)	0.89	18 (38.3)	49 (30.1)	0.29
Post Admission Ventilation:	26 (7.7)	9 (11.7)	5 (3.7)	0.025	6 (7.8)	8 (6.0)	0.62	5 (10.6)	9 (5.5)	0.22
ICU Length of Stay (Mean (SD))	7.6 (7.4)	9.42 (7.5)	6.42 (5.8)	0.16	7.6 (5.4)	7.8 (7.3)	0.93	9.4 (7.2)	7.4 (6.6)	0.49
Hospital Length of Stay (Mean (SD))	8.9 (9.5)	11.7 (10.2)	6.9 (5.7)	<0.001	10.4 (9.3)	7.7 (7.0)	0.03	9.9 (7.2)	8.3 (8.2)	0.21
Abbrev: CPR = Cardio-pulmonary Resuscitation; ICU = Intensive Care Unit; IQR = Interquartile Range; Ped = Pedestrian; RTC = Road Traffic Collision										

For peer review only - http://bmjopen.bmj.com/site/about/guidelines.xhtml

Changes in HRQoL over six-months after discharge from hospital

Physical function was clearly affected by the injuries experienced by this cohort. Measures of HRQoL are presented in **Table 2**. As expected, the mean PCS score (SF-12) saw a 19.3% (46.5-27.2) drop from the pre-injury estimate through to the initial hospital discharge timepoint representing a substantial negative change in physical functioning. Over the 6-month follow-up there were incremental improvements in the mean PCS score (31.6, 40.1, and 41.7 at 1, 3 and 6-month respectively) but the sample failed to return to their baseline estimate of physical function (-4.8%) or population norms (-8.3%) within the 6-month follow-up period. During the same timeframe, the impact of injury on mental health was lesser than on physical function. The mean MCS score (SF-12) dropped from 49.7 to 44.4 (-5.3) from the pre-injury estimate through to hospital discharge, dropping a further 0.5% to 43.9 at 1-month after hospital discharge. Between the 1 and 6 months the mean MCS increased to 47.8 which remained 1.9% below the pre-injury estimate and 2.2% below the population norms for England.

For peer review only

Table 2: Cohort outcomes with comparisons between outcomes of interest at six-months after discharge

	Outcome	Total sample (n=337)	Poor physical function (n=77)	Good physical function (n=134)	p-value	Chronic Pain (n=77)	No chronic pain (n=134)	p-value	Neuropathic pain likely (n=47)	Neuropathic pain unlikely (n=164)	p-value
Pre-Injury	SF-12 PCS +	46.5 (11.0)	40.0 (11.0)	50.8 (7.7)	<0.001	41.9 (11.4)	49.6 (8.6)	<0.001	41.7 (11.5)	48.3 (9.6)	<0.001
	SF-12 MCS +	49.7 (10.7)	48.2 (11.7)	53.1 (7.6)	<0.001	49.1 (10.9)	52.7 (8.4)	0.009	48.2 (10.6)	52.3 (9.1)	0.009
Discharge (n=320)	SF-12 PCS +	27.2 (8.6)	24.3 (7.4)	28.4 (8.6)	0.001	25.1 (8.3)	27.9 (8.3)	0.02	25.1 (8.4)	27.5 (8.3)	0.09
	SF-12 MCS +	44.4 (11.8)	43.5 (11.8)	47.0 (11.5)	0.04	46.1 (11.5)	45.5 (11.9)	0.76	42.6 (11.7)	46.7 (11.6)	0.04
	BPI Pain Severity Score +	5.0 (2.1)	5.3 (2.1)	4.8 (2.0)	0.10	5.3 (2.0)	4.7 (2.0)	0.06	5.6 (2.0)	4.7 (2.0)	0.009
	BPI Pain Interference Score +	5.7 (2.3)	6.5 (2.0)	5.2 (2.4)	<0.001	5.9 (2.0)	5.5 (2.4)	0.18	6.4 (2.0)	5.4 (2.3)	0.004
	PDQ: Low Risk ++	222 (69.4)	48 (64.9)	95 (73.1)		47 (63.5)	96 (74.4)		24 (52.2)	119 (75.8)	
	PDQ: Moderate Risk ++	67 (20.9)	18 (24.3)	26 (20.0)	0.42	22 (29.7)	21 (16.3)	0.075	17 (37.0)	26 (16.6)	0.006
	PDQ: High Risk ++	31 (9.7)	8 (10.8)	9 (6.9)		5 (6.8)	12 (9.3)		5 (10.9)	12 (7.6)	
1-month (n=210)	SF-12 PCS +	31.6 (7.8)	27.0 (6.0)	33.5 (7.5)	<0.001	27.7 (6.0)	32.9 (7.8)	<0.001	27.8 (5.8)	32.1 (7.9)	<0.001
	SF-12 MCS +	43.9 (11.8)	40.8 (12.2)	46.8 (11.2)	0.001	4.07 (12.7)	46.7 (10.9)	0.001	35.6 (12.3)	47.0 (10.6)	<0.001
	BPI Pain Severity Score +	3.8 (2.2)	4.7 (2.3)	3.2 (1.9)	<0.001	5.2 (2.0)	3.0 (1.9)	<0.001	5.5 (2.2)	3.3 (1.9)	<0.001
	BPI Pain Interference Score +	4.4 (2.7)	5.6 (2.6)	3.7 (2.5)	<0.001	5.8 (2.4)	3.6 (2.5)	<0.001	5.6 (2.4)	3.8 (2.4)	<0.001
	PDQ: Low Risk ++	142 (67.6)	39 (61.9)	83 (73.5)		34 (57.6)	88 (75.2)		14 (38.9)	108 (77.1)	
	PDQ: Moderate Risk ++	45 (21.4)	14 (22.2)	22 (19.5)	0.14	13 (22.0)	23 (19.7)	0.005	9 (25.0)	27 (19.3)	<0.001
	PDQ: High Risk ++	23 (11.0)	10 (15.9)	8 (7.1)		12 (20.3)	6 (5.1)		13 (36.1)	5 (3.6)	
3-months (n=144)	SF-12 PCS +	40.1 (11.0)	31.4 (8.8)	44.4 (9.5)	<0.001	32.4 (9.1)	43.9 (10.0)	<0.001	32.9 (8.8)	42.1 (10.8)	<0.001
	SF-12 MCS +	48.3 (11.4)	43.1 (12.9)	50.9 (9.7)	<0.001	43.1 (11.8)	51.0 (10.4)	<0.001	40.0 (12.0)	50.7 (10.2)	<0.001
	BPI Pain Severity Score +	2.9 (2.6)	4.4 (2.7)	2.2 (2.2)	<0.001	5.1 (2.2)	1.8 (2.0)	<0.001	4.8 (2.2)	2.4 (2.4)	<0.001
	BPI Pain Interference Score +	2.6 (2.7)	4.4 (3.1)	1.8 (2.1)	<0.001	5.0 (2.6)	1.5 (2.0)	<0.001	5.0 (2.6)	2.0 (2.4)	<0.001
	PDQ: Low Risk ++	110 (76.4)	28 (60.9)	79 (83.2)		23 (48.9)	84 (89.4)	<0.001	9 (29.0)	98 (89.1)	
	PDQ: Moderate Risk ++	15 (10.4)	7 (15.2)	8 (8.4)	0.012	9 (19.1)	6 (6.4)		9 (29.0)	6 (5.5)	<0.001
	PDQ: High Risk ++	19 (13.2)	11 (23.9)	8 (8.4)		15 (31.9)	4 (4.3)		13 (41.9)	6 (5.5)	
6-months (n=211)	SF-12 PCS +	41.7 (11.5)	29.2 (5.1)	48.9 (7.2)	<0.001	32.9 (8.0)	46.8 (10.1)	<0.001	32.4 (8.5)	44.4 (10.9)	<0.001
	SF-12 MCS +	47.8 (11.8)	42.3 (13.2)	51.0 (9.7)	<0.001	39.4 (11.6)	52.7 (8.9)	<0.001	36.4 (11.8)	51.1 (9.6)	<0.001
	BPI Pain Severity Score +	2.7 (2.7)	4.8 (2.6)	1.5 (2.0)	<0.001	5.8 (1.8)	0.9 (1.1)	<0.001	5.4 (2.3)	1.9 (2.3)	<0.001
	BPI Pain Interference Score +	2.8 (2.9)	5.2 (2.7)	1.4 (2.0)	<0.001	5.6 (2.3)	1.2 (1.7)	<0.001	6.1 (2.6)	1.8 (2.2)	<0.001
	PDQ: Low Risk ++	164 (77.7)	44 (57.9)	119 (88.8)		40 (51.9)	123 (92.5)		0	163 (100)	
	PDQ: Moderate Risk ++	28 (13.3)	17 (22.4)	11 (8.2)	<0.001	20 (26.0)	8 (6.0)	<0.001	28 (59.6)	0	<0.001
	PDQ: High Risk ++	19 (9.0)	15 (19.7)	4 (3.0)		17 (22.1)	2 (1.5)		19 (40.4)	0	

Abbrev: BPI = Brief Pain Inventory; IQR = Interquartile Range; MCS = Mental Component Score; PCS = Physical Component Score; PDQ = PainDetect Questionnaire; SD = Standard Deviation; SF-12 = Short Form -12 Health Survey.

For peer review only - http://bmjopen.bmj.com/site/about/guidelines.xhtml

Key: + = Mean (±SD); ++ = n (%)

364

For peer review only

For peer review only - http://bmjopen.bmj.com/site/about/guidelines.xhtml

The impact of BTI on individual components of HRQoL were measured using the EuroQol-5d-5L questionnaire. **Table 3** presents the individual dimension/level data for each timepoint. At hospital discharge, the lowest scored (best) dimension was Anxiety and Depression with 43% of the sample reporting no problems in this dimension. At 6-months after discharge: ‘Mobility’, ‘Self-Care’, ‘Usual Activities’ and ‘Anxiety/Depression’ were the best rated dimensions, with between 42% and 73% of the sample reporting no problems in these areas. The highest scoring (worst) dimensions at hospital discharge were ‘Usual Activities’ where 42% (n=135) reported being wholly unable to complete usual activities. Between 42-43% of the sample reported moderate (3), severe (4) or extreme (5) pain at both 3-months and 6-months after discharge from hospital (42.3% (n=61) at 3-months and 43.1% (n=91) at six months). At 6-months after injury, there was a proportion of participants who reported severe or extreme problems in all dimensions, and these exceeded the pre-injury estimated levels. This demonstrates that although the 6-month follow-up period was sufficient to observe recovery in a vast proportion of this sample, there were participants who reported extreme disability beyond the follow-up.

For peer review only - http://bmjopen.bmj.com/site/about/guidelines.xhtml

Table 3: Individual dimension and level data from EuroQoL-5D-(5L)

(n= (%))	No Problems	Slight Problems	Moderate Problems	Severe Problems	Unable/extreme Problems
**Mobility:**					
Pre-injury	204 (60.5)	50 (14.8)	47(13.9)	33 (9.8)	3 (0.9)
Discharge	50 (15.6)	78 (24.4)	102 (31.9)	72 (22.5)	18 (5.6)
One-Month	65 (31.0)	76 (36.2)	38 (18.1)	26 (12.4)	5 (2.4)
Three-Months	71 (49.3)	36 (25.0)	17 (11.8)	16 (11.1)	4 (2.8)
Six-Months	110 (52.1)	41 (19.4)	32 (15.2)	23 (10.9)	5 (2.4)
**Self-Care:**					
Pre-injury	269 (79.8)	33 (9.8)	23 (6.8)	10 (3.0)	2 (0.6)
Discharge	68 (21.3)	92 (28.8)	81 (25.3)	59 (18.4)	20 (6.3)
One-Month	108 (51.4)	59 (28.1)	30 (14.3)	10 (4.8)	3 (1.4)
Three-Months	106 (73.6)	21 (14.6)	9 (6.3)	6 (4.2)	2 (1.4)
Six-Months	153 (72.5)	31 (14.7)	20 (9.5)	5 (2.4)	2 (0.9)
**Usual Activities:**					
Pre-injury	224 (66.5)	53 (15.7)	33 (9.8)	19 (5.6)	8 (2.4)
Discharge	21 (6.6)	36 (11.3)	72 (22.5)	56 (17.5)	135 (42.2)
One-Month	20 (9.5)	95 (45.2)	41 (19.5)	29 (13.8)	25 (11.9)
Three-Months	49 (34.0)	51 (35.4)	22 (15.3)	9 (6.3)	13 (9.0)
Six-Months	89 (42.2)	62 (29.4)	34 (16.1)	14 (6.6)	12 (5.7)
**Pain/Discomfort:**					
Pre-injury	157 (46.6)	94 (27.9)	61 (18.1)	18 (5.3)	7 (2.1)
Discharge	14 (4.4)	41 (12.8)	151 (47.2)	74 (23.1)	40 (12.5)
One-Month	15 (7.1)	49 (23.3)	112 (53.3)	22 (10.5)	12 (5.7)
Three-Months	37 (25.7)	46 (31.9)	47 (32.6)	10 (6.9)	4 (2.8)
Six-Months	72 (34.1)	48 (22.7)	72 (34.1)	11 (5.2)	8 (3.8)
**Anxiety/Depression:**					
Pre-injury	188 (55.8)	79 (23.4)	54 (16.0)	11 (3.3)	5 (1.5)
Discharge	138 (43.1)	85 (26.6)	64 (20.0)	20 (6.3)	13 (4.1)
One-Month	100 (47.6)	38 (18.1)	57 (27.1)	7 (3.3)	8 (3.8)
Three-Months	82 (56.9)	27 (18.8)	26 (18.1)	4 (2.8)	5 (3.5)
Six-months	119 (56.4)	42 (19.9)	33 (19.9)	8 (3.8)	9 (4.3)

For peer review only

Evolution of chronic pain and pain with a neuropathic component

Subjective pain evaluated through the BPI Pain Severity Score (PSS) reflects the persistent levels of pain experienced by study participants throughout the 6-month study follow-up period. Pain related outcome measures are presented in **Table 2**. Although there were consistent incremental reductions in the mean pain score over time, at 6-months after injury the mean pain severity score was 2.7 (SD 2.7); 36.5% (n=77) of participants were reporting chronic pain. The mean BPI Pain Interference Score (PIS) also demonstrated incremental decreases until the 3-month timepoint where there appears to be a plateau, followed by a marginal deterioration of the mean PIS (2.6 (SD 2.7) to 2.7 (SD 2.9)). This highlights that participants reporting pain that impacted on daily activities at three months were likely to continue to experience these interferences at 6-months after hospital discharge. Similarly, mean self-reported analgesic effectiveness decreased from 65.3 (SD 24.8) at hospital discharge to 46.5 (SD 35.8) at 3-months which then plateaued and was 49.2 (SD 33.4) at 6-months after hospital discharge. Individual components of the BPI PSS and BPI PIS can be seen in the supplementary file **Table S3 and S4.**

At hospital discharge and 1-month after, 30.6% (n= 98) and 32.4% (68%) (respectively) of patients reported moderate or high risk of having a neuropathic pain component. At 3-months and 6-months, there was a reduction in the number of participants reporting neuropathic pain (23.6% (n=34) and 22.3% (n=47) respectively) with 13.2% (n=19) and 9.0% (n=19) respectively reporting PainDetect scores in the high-risk category (representing 90% likelihood of having a neuropathic pain component).

Development of Predictive models for the three outcomes of interest

Table 4 highlights three models developed in this study, one for each outcome. Initially candidate predictors were selected from variables readily available within the inpatient clinical journey (including hospital discharge), as this would be most useful in clinical practice. Unfortunately, we were unable to isolate a model with acceptable levels of discrimination and calibration using these variables alone. In a second approach models were developed using variables from the post discharge follow-up period (Table 4). Three models with acceptable levels of calibration and discrimination were identified. All models require measures collected at hospital discharge, 1-month, and 3-months after discharge to predict the outcomes of interest.

Model 1: Poor physical function at 6-months after hospital discharge was predicted using the following: the combination of the measures of physical function both prior to injury and at 3-months

For peer review only - http://bmjopen.bmj.com/site/about/guidelines.xhtml

after injury, a measurement of pain levels at 1-month after discharge, and a measurement of the impact of pain on the individuals walking ability at hospital discharge. **Model 2:** It was possible to predict chronic pain at 6-months after hospital discharge using the follow variables: Pre-injury regular use of analgesic agents, measures of physical function before the injury and at 3-months after discharge, and a measurement of pain severity at 1- and 3-months after discharge. **Model 3:** neuropathic pain was predicted using the following variables: starting Pregabalin during the acute hospital admission, the Mental Component Score at 1-month and the impact of pain on mood at 3-months after discharge, and the outcome of taking the PainDetect Questionnaire at 3-months after discharge.

For peer review only - http://bmjopen.bmj.com/site/about/guidelines.xhtml

Table 4: Predictive models for three outcomes of interest

	Co-efficient	Std error	Odds Ratio	P-value	95% CI
**Model 1: Poor physical function at 6-months after hospital discharge**					
Pre-Injury PCS (Range: 0-100) (1 step increment increase)	-0.093	0.019	0.911	<0.001	0.878-0.945
Current Pain at 1-month after injury (Range 0-10) (1 step increment increase)	0.224	0.078	1.252	0.004	1.075-1.457
PCS at 3 months (Range: 0-100) (1 step increment increase)	-0.040	0.014	0.960	0.005	0.934-0.988
Impact of pain on walking at discharge (Range: 0-10) (1 step increment increase)	0.139	0.057	1.149	0.015	1.027-1.285
Constant	*3.745*	*1.132*	-	*0.001*	-
**Model 2: Chronic pain at 6-months after hospital discharge**					
Regular Analgesia use before injury	1.813	0.533	6.130	0.001	2.158-17.414
Pre-Injury PCS (Range: 0-100) (1 step increment increase)	-0.039	0.019	0.962	0.044	0.926-0.999
Pain score at 1 month after discharge (Range: 0-10) (1 step increment increase)	0.270	0.089	1.310	0.003	1.099-1.560
PCS at 3 months (Range: 0-100) (1 step increment increase)	-0.051	0.015	0.951	0.001	0.922-0.980
Current Pain score 3 months after discharge (Range: 0-10) (1 step increment increase)	0.219	0.084	1.245	0.009	1.057-1.467
Constant	*1.361*	*1.098*	-	*0.215*	-
**Model 3: Likely neuropathic component to pain at 6-months after hospital discharge**					
Commenced on Pregabalin during Hospital admission	2.340	1.156	10.377	0.043	1.076-100.113
MCS at 1 month after discharge (Range: 0-100) (1 step increment increase)	-0.058	0.026	0.944	0.026	0.897-0.993
Pain Detect score at 3 months (Range: -1-38) (1 step increment increase)	0.174	0.043	1.190	<0.001	1.094-1.295
Impact of Pain on Mood at 3 months after discharge (Range 0-10) (1 step increment increase)	0.192	0.095	1.212	0.043	1.212-1.006
Constant	*-1.421*	*1.188*	-	*0.232*	-

<u>Insert Figure 1 and Figure 2 here please.</u>

For peer review only

The ROC diagrams for each model are presented in **Figure 1**. **Figure 2** presents the graphitised calibration plots whereby plot points present risk of outcome in tenths of patients with similar predicted probabilities. The line highlights the relationship between the observed frequency and the predicted probability of developing a poor outcome. **Model 1** for predicting poor physical functioning at 6 months after discharge had a c-statistic (area under the receiver operator curve) of 0.846 (Std error: 0.028; p<0.001; 95%CI 0.790-0.901) with a Hosmer and Lemeshow Chi-square value of 5.570 (p=0.695) with 8 degrees of freedom (df), demonstrating acceptable goodness-of-fit. This resulted in a model with a sensitivity of 79.0% and a specificity of 81.2%. **Model 2** for predicting chronic pain at 6-months after discharge had a c-statistic of 0.878 (Std error: 0.023; p<0.001; 95%CI 0.833-0.923) with a Hosmer and Lemeshow Chi-Square value of 6.778 (p=0.561) with 8 degrees of freedom (df), also demonstrating acceptable goodness-of-fit. This resulted in a model with a sensitivity of 63.6% and a specificity of 86.5%. Finally, **Model 3** for predicting pain with a neuropathic component had a c-statistic of 0.910 (Std error: 0.027; <0.001; 95%CI 0.857-0.964) with a Hosmer and Lemeshow Chi-square value of 2.221 (p=0.912) with 8 degrees of freedom (df), demonstrating acceptable goodness-of-fit. This resulted in a model with a sensitivity of 61.3% and a specificity of 92.7%. The overall accuracy of models 1-3 were 80.6%, 78.1% and 85.8% respectively. The calibration plots for each model **(Fig. 2)** demonstrated acceptable levels of calibration as many of the plots were close to the 45-degree line.

Discussion:

This study investigated changes in self-reported HRQoL and pain outcomes over a 6-month period from hospital discharge safter BTI. The study has provided new insights into the recovery of this patient group within the UK. This study has identified the high levels of pain and poor physical function experienced after BTI in the UK and measured the presence of neuropathic pain components within this population. Although it was not possible to construct a predictive model using only clinically available baseline and discharge level variables, the process of predictive prognostic modelling has identified factors that predict outcomes. This suggests that it is possible to develop a risk stratification score, but this involves using variables which are not routinely available in clinical practice currently. It is acknowledged there is limited clinical utility in these current models as the data required to achieve accurate predictions of outcomes is not routinely available in clinical practice. Further research is needed to understand how clinically useful prediction models can be developed for this population. There is potential, with refinement, to make the current prediction models more clinically useful, and there are ways that this score could have clinical utility in

For peer review only - http://bmjopen.bmj.com/site/about/guidelines.xhtml

identifying patients at risk of poor functional outcomes or chronic/neuropathic pain, subject to external validation in a future study. In respect to the models presented in this study, it is important to remember that good model performance in a development sample does not necessarily mean good predictive abilities in a new external validation dataset.

The limitations of using generic Patient Reported Outcome Measures (PROMs) to measure HRQoL have been reported previously.[7 32] Although validated within the major trauma population, it remains unclear how specific and comprehensive generic outcome measures (e.g., SF-12 and EQ5D-5L) are in groups like the BTI population. Although a trauma PROM has previously been developed,[33] there is a need for injury specific measures, particularly where unique outcomes have been identified.[7 34] Physical functioning was measured at a global level in this study using the SF-12 PCS and using the individual levels of the EuroQoL-5d-5L questionnaire. Both measures highlighted the extent of the altered physical function experienced by this population, which had not returned to their perceived baseline level of function at 6-months. In previous non-UK research, sequela related to BTI has been measured up to 2-years after injury highlighting the long-term impact of BTI.[35] Due to funding, it was not possible to extend the follow-up in the current study, but the 6-month timepoint was clinically ideal for the prognostic modelling process, particularly as Marasco et al. highlighted that patients reporting poor physical function at 6-months after injury reported poor physical function at 1 year and greater odds of reporting poor physical function at 2-years after injury.[35]

One key factor that influences HRQoL after BTI is pain, and the efficacy of pain management.[6] Measures of pain severity within this study highlighted 43.1% (n=91) of participants reported pain that was moderate, severe, or extreme at 6-months after hospital discharge with a mean Pain Severity Score of 2.7 (SD 2.7). For these participants, the mean analgesic effectiveness was 49.2 (SD 33.4) demonstrating how a chronic pain state existed where analgesic agents were not effective in managing pain. The variables in model 2 demonstrate the complexity in chronic pain management particularly in identifying those at risk through the predictive modelling process. Within this model it was not possible to predict the likelihood of whether a participant would experience chronic pain at 6-months after discharge using clinically available variables only. The final model included variables from pre-injury estimates through to 3-months after discharge which highlighted the need to alter our current model for patient follow-up to enable measurements at 1 and 3 months after discharge. Unsurprisingly, participants using regular analgesic agents prior to their injury had an odds ratio greater than six highlighting the need to identify patients with pre-existing chronic pain states during hospital admission as these patients are most likely to have ongoing injury related pain requirements at six months after hospital discharge.

For peer review only - http://bmjopen.bmj.com/site/about/guidelines.xhtml

There were greater odds of reporting neuropathic pain at 6 months if the participant had been initiated on a neuropathic pain agent (e.g. pregabalin) during the acute hospital admission. This suggests that clinical teams and specialist pain teams are accurately identifying patients at risk of neuropathic pain and commencing treatment. This unfortunately also highlights that these patients are still progressing to develop symptoms of neuropathic pain at six-months after discharge which suggests that greater input from specialist pain services after hospital discharge may be required to manage this pain trajectory. The model for neuropathic pain highlights an association with the MCS of the SF-12 at one month and the effect of pain on mood at 3-months after hospital discharge. The relationship between neuropathic pain and mental health has previously been identified in the non-trauma literature.[36] Although there was a less substantial drop in SF-12 MCS compared to the PCS and this returned to the pre-injury baseline within the 6-month follow-up period, this shows the importance of measuring mental wellbeing as both a part of HRQoL and as an important predictor of neuropathic pain.

This study has developed a model for each of the three outcomes of interest: physical function, chronic pain, and neuropathic pain at 6-months after hospital discharge with BTI. The next phase of the modelling process will be to conduct a further study to validate the predictive ability of the models within an external sample of participants with BTI. This study will further explore the follow-up needs of patients with BTI and look at effective ways of collecting these data needed to predict 6-month outcomes. It will also be of interest to investigate these outcomes beyond the 6-months follow-up period used in this study. In the meantime, these predictive variables have clinical utility for clinicians reviewing patients during the follow-up period in primary and secondary care settings and these variables may be useful in highlighting patients at risk who need referral to specialist services (e.g., physiotherapy or specialist pain services).

Strengths and limitations of this study:

Although we recruited a moderate sized sample using the exploratory approach to this study, we did not achieve the aim of recruiting 100 events relating to our three outcomes of interest. Care was taken to ensure that that the number of variables inputted into each model were justified by the number participants reporting the outcomes of interest. There are reports within the literature that the arbitrary rule of 10 cases per variable does not necessarily negatively impact on the model fit.[37] During subsequent validation of the models, care will be taken to assess for overfitting of the models but from the model diagnostics presented here, there is no suggestion of overfitting within the current sample.[31] It is important to remember that the final models are to some degree variable selection method dependent. In an ideal world, it would have been beneficial to use more than one

For peer review only - http://bmjopen.bmj.com/site/about/guidelines.xhtml

method of selection but in the case of this study, it was not possible to undertake backward selection because of concerns of overfitting the model.

Overall, the combined response rate of 72% at all timepoints is comparable to equivalent studies undertaken previously in this area of trauma research.[6] Despite this, the response rate was a limitation as the key outcomes were measured at the 6-month timepoint resulting in only 63% (n=211) of the overall recruited sample being included in the modelling process. For further studies investigating outcomes within a longitudinal follow-up process, it will be important to use these data to consider potential sample sizes required to adequately power these studies.

Although multiple imputation was used in the management of missingness for independent variables, it is acknowledged that the case-wise deletion approach used in the management of missingness in the outcome data is a limitation in this study. In the presence of MAR data, this approach results in large proportions of data being discarded, which results in the introduction of potential bias, and reduced precision and power. For this reason, and despite there being no clinically significant differences between the characteristics of responders and non-responders, the findings of this study should be interpreted cautiously. Despite all efforts to maximise response rates at all timepoints, future longitudinal studies in the BTI population will need to consider other ways of accessing patients to facilitate complete follow-up data collection in a geographically diverse population.

As subjective outcome measures have been used throughout this study, it is important to acknowledge the risk of bias which is inherent within these measures, particularly in relation to participants selecting the extremes of a rating scale.[38] Care has been taken within the study to control this bias through the instructions given to patients prior to completing each questionnaire and investigation of outliers within the sample during data analysis.[39] In future research, it would be interesting to compare the predictive ability of both subjective outcomes (i.e. SF-12 PCS for physical function) with an objective measure (grip strength testing as an estimate of physical functional ability for example) and compare the difference in the predictive functions of these outcomes.[40]

Finally, although most of the study was undertaken before the Covid-19 pandemic began in the UK, there will be seen and unseen impacts of the Covid-19 pandemic on this study.[41] The final three sites closed to recruitment 2 to 4 weeks early causing them to not meet recruitment targets. Furthermore, follow-up continued throughout the first wave of the pandemic in the UK. Although we were not able to collect data relating Covid-19 infection amongst our sample, we were not informed that any participants had been infected with the virus. Despite this, there is potential bias

For peer review only - http://bmjopen.bmj.com/site/about/guidelines.xhtml

602 relating to lockdown, social isolation that remains unmeasured within our sample which may have
603 impacted on the self-reported outcome measures reported in this study.[42]

604 Conclusions:

605 It remains vital that the interdisciplinary team do not underestimate the impact of the long-term
606 outcomes associated with BTI, particularly relating to physical functioning, chronic pain, and
607 neuropathic pain. This study has demonstrated how predictive models can be developed for these
608 outcomes and highlight how PROMS can be used to develop prognostic models particularly in
609 longitudinal processes where objective clinical data is not routinely collected. These models add to
610 our understanding of which factors influence the development of sequela after hospital discharge
611 with BTI and after external validation, will have clinical utility in the risk stratification of high-risk
612 patient groups.

613

For peer review only

For peer review only - http://bmjopen.bmj.com/site/about/guidelines.xhtml

Reference:

1. Baker EJ, Lee GA. A Retrospective Observational Study Examining the Effect of Thoracic Epidural and Patient Controlled Analgesia on Short-term Outcomes in Blunt Thoracic Trauma Injuries. *Medicine (Baltimore)* 2016;95(2):e2374. doi: 10.1097/MD.0000000000002374
2. Battle C, Hutchings H, Lovett S, et al. Predicting outcomes after blunt chest wall trauma: development and external validation of a new prognostic model. *Crit Care* 2014;18(3):R98. doi: 10.1186/cc13873 [published Online First: 2014/05/14]
3. de Graaf MW, Reininga IHF, Heineman E, et al. The development and internal validation of a model to predict functional recovery after trauma. *PLoS One* 2019;14(3):e0213510. doi: 10.1371/journal.pone.0213510 [published Online First: 2019/03/14]
4. Cole E, Lecky F, West A, et al. The Impact of a Pan-regional Inclusive Trauma System on Quality of Care. *Ann Surg* 2016;264(1):188-94. doi: 10.1097/SLA.0000000000001393
5. Martino C, Russo E, Santonastaso DP, et al. Long-term outcomes in major trauma patients and correlations with the acute phase. *World J Emerg Surg* 2020;15:6. doi: 10.1186/s13017-020-0289-3 [published Online First: 2020/01/13]
6. Baker E, Xyrichis A, Norton C, et al. The long-term outcomes and health-related quality of life of patients following blunt thoracic injury: a narrative literature review. *Scand J Trauma Resusc Emerg Med* 2018;26(1):67. doi: 10.1186/s13049-018-0535-9 [published Online First: 2018/08/17]
7. Craxford S, Deacon C, Myint Y, et al. Assessing outcome measures used after rib fracture: A COSMIN systematic review. *Injury* 2019;50(11):1816-25. doi: 10.1016/j.injury.2019.07.002 [published Online First: 2019/07/04]
8. Wilson IB, Cleary PD. Linking clinical variables with health-related quality of life. A conceptual model of patient outcomes. *JAMA* 1995;273(1):59-65.
9. Ojelabi AO, Graham Y, Haighton C, et al. A systematic review of the application of Wilson and Cleary health-related quality of life model in chronic diseases. *Health Qual Life Outcomes* 2017;15(1):241. doi: 10.1186/s12955-017-0818-2 [published Online First: 2017/12/11]
10. Baker E, Woolley A, Xyrichis A, et al. How does the implementation of a patient pathway-based intervention in the acute care of blunt thoracic injury impact on patient outcomes? A systematic review of the literature. *Injury* 2020 doi: 10.1016/j.injury.2020.06.002 [published Online First: 2020/06/04]
11. Karimi M, Brazier J. Health, Health-Related Quality of Life, and Quality of Life: What is the Difference? *Pharmacoeconomics* 2016;34(7):645-9. doi: 10.1007/s40273-016-0389-9
12. Vernon D, Brown JE, Griffiths E, et al. Reducing readmission rates through a discharge follow-up service. *Future Healthc J* 2019;6(2):114-17. doi: 10.7861/futurehosp.6-2-114
13. Braaf S, Ameratunga S, Nunn A, et al. Patient-identified information and communication needs in the context of major trauma. *BMC Health Serv Res* 2018;18(1):163. doi: 10.1186/s12913-018-2971-7 [published Online First: 2018/03/07]
14. Keene DJ, Vadher K, Willett K, et al. Predicting patient-reported and objectively measured functional outcome 6 months after ankle fracture in people aged 60 years or over in the UK: prognostic model development and internal validation. *BMJ Open* 2019;9(7):e029813. doi: 10.1136/bmjopen-2019-029813 [published Online First: 2019/07/23]
15. Ware J, Kosinski M, Keller SD. A 12-Item Short-Form Health Survey: construction of scales and preliminary tests of reliability and validity. *Med Care* 1996;34(3):220-33.
16. Devlin NJ, Parkin D, Browne J. Patient-reported outcome measures in the NHS: new methods for analysing and reporting EQ-5D data. *Health Econ* 2010;19(8):886-905. doi: 10.1002/hec.1608
17. Parkin D, Rice N, Devlin N. Statistical analysis of EQ-5D profiles: does the use of value sets bias inference? *Med Decis Making* 2010;30(5):556-65. doi: 10.1177/0272989X09357473 [published Online First: 2010/03/12]

For peer review only - http://bmjopen.bmj.com/site/about/guidelines.xhtml

18. Kumar SP. Utilization of brief pain inventory as an assessment tool for pain in patients with cancer: a focused review. *Indian J Palliat Care* 2011;17(2):108-15. doi: 10.4103/0973-1075.84531
19. Freynhagen R, Baron R, Gockel U, et al. painDETECT: a new screening questionnaire to identify neuropathic components in patients with back pain. *Curr Med Res Opin* 2006;22(10):1911-20. doi: 10.1185/030079906X132488
20. Battle CE, Hutchings H, James K, et al. The risk factors for the development of complications during the recovery phase following blunt chest wall trauma: a retrospective study. *Injury* 2013;44(9):1171-6. doi: 10.1016/j.injury.2012.05.019 [published Online First: 2012/06/12]
21. Treede RD, Rief W, Barke A, et al. A classification of chronic pain for ICD-11. *Pain* 2015;156(6):1003-7. doi: 10.1097/j.pain.0000000000000160
22. Boonstra AM, Schiphorst Preuper HR, Balk GA, et al. Cut-off points for mild, moderate, and severe pain on the visual analogue scale for pain in patients with chronic musculoskeletal pain. *Pain* 2014;155(12):2545-50. doi: 10.1016/j.pain.2014.09.014 [published Online First: 2014/09/17]
23. Murnion BP. Neuropathic pain: current definition and review of drug treatment. *Aust Prescr* 2018;41(3):60-63. doi: 10.18773/austprescr.2018.022 [published Online First: 2018/06/01]
24. Mathieson S, Lin C. painDETECT questionnaire. *J Physiother* 2013;59(3):211. doi: 10.1016/S1836-9553(13)70189-9
25. Unsworth A, Curtis K, Asha SE. Treatments for blunt chest trauma and their impact on patient outcomes and health service delivery. *Scand J Trauma Resusc Emerg Med* 2015;23:17. doi: 10.1186/s13049-015-0091-5 [published Online First: 2015/02/08]
26. Kourouche S, Buckley T, Munroe B, et al. Development of a blunt chest injury care bundle: An integrative review. *Injury* 2018;49(6):1008-23. doi: 10.1016/j.injury.2018.03.037 [published Online First: 2018/04/07]
27. von Elm E, Altman DG, Egger M, et al. The Strengthening the Reporting of Observational Studies in Epidemiology (STROBE) Statement: guidelines for reporting observational studies. *Int J Surg* 2014;12(12):1495-9. doi: 10.1016/j.ijsu.2014.07.013 [published Online First: 2014/07/18]
28. Collins GS, Reitsma JB, Altman DG, et al. Transparent reporting of a multivariable prediction model for individual prognosis or diagnosis (TRIPOD): the TRIPOD statement. *BMJ* 2015;350:g7594. doi: 10.1136/bmj.g7594 [published Online First: 2015/01/07]
29. Pareek N, Kordis P, Beckley-Hoelscher N, et al. A practical risk score for early prediction of neurological outcome after out-of-hospital cardiac arrest: MIRACLE2. *Eur Heart J* 2020 doi: 10.1093/eurheartj/ehaa570 [published Online First: 2020/07/30]
30. Shipe ME, Deppen SA, Farjah F, et al. Developing prediction models for clinical use using logistic regression: an overview. *J Thorac Dis* 2019;11(Suppl 4):S574-S84. doi: 10.21037/jtd.2019.01.25
31. Alba AC, Agoritsas T, Walsh M, et al. Discrimination and Calibration of Clinical Prediction Models: Users' Guides to the Medical Literature. *JAMA* 2017;318(14):1377-84. doi: 10.1001/jama.2017.12126
32. Hoffman K, Cole E, Playford ED, et al. Health outcome after major trauma: what are we measuring? *PLoS One* 2014;9(7):e103082. doi: 10.1371/journal.pone.0103082 [published Online First: 2014/07/22]
33. Attenberger C, Amsler F, Gross T. Clinical evaluation of the Trauma Outcome Profile (TOP) in the longer-term follow-up of polytrauma patients. *Injury* 2012;43(9):1566-74. doi: 10.1016/j.injury.2011.01.002 [published Online First: 2011/01/20]
34. Rosenberg GM, Stave C, Spain DA, et al. Patient-reported outcomes in trauma: a scoping study of published research. *Trauma Surg Acute Care Open* 2018;3(1):e000202. doi: 10.1136/tsaco-2018-000202 [published Online First: 2018/09/04]

For peer review only

35. Marasco S, Lee G, Summerhayes R, et al. Quality of life after major trauma with multiple rib fractures. *Injury* 2015;46(1):61-5. doi: 10.1016/j.injury.2014.06.014 [published Online First: 2014/06/21]
36. Torta R, Ieraci V, Zizzi F. A Review of the Emotional Aspects of Neuropathic Pain: From Comorbidity to Co-Pathogenesis. *Pain Ther* 2017;6(Suppl 1):11-17. doi: 10.1007/s40122-017-0088-z [published Online First: 2017/11/24]
37. Ogundimu EO, Altman DG, Collins GS. Adequate sample size for developing prediction models is not simply related to events per variable. *J Clin Epidemiol* 2016;76:175-82. doi: 10.1016/j.jclinepi.2016.02.031 [published Online First: 2016/03/08]
38. Dowling NM, Bolt DM, Deng S, et al. Measurement and control of bias in patient reported outcomes using multidimensional item response theory. *BMC Med Res Methodol* 2016;16:63. doi: 10.1186/s12874-016-0161-z [published Online First: 2016/05/26]
39. Tempelaar D, Rienties B, Nguyen Q. Subjective data, objective data and the role of bias in predictive modelling: Lessons from a dispositional learning analytics application. *PLoS One* 2020;15(6):e0233977. doi: 10.1371/journal.pone.0233977 [published Online First: 2020/06/12]
40. Koskinen S, Hokkinen EM, Wilson L, et al. Comparison of subjective and objective assessments of outcome after traumatic brain injury using the International Classification of Functioning, Disability and Health (ICF). *Disabil Rehabil* 2011;33(25-26):2464-78. doi: 10.3109/09638288.2011.574776 [published Online First: 2011/05/03]
41. Baker E, Lee G. Reflecting on the challenges of sustaining emergency care research during the Covid-19 pandemic. *Int Emerg Nurs* 2020;51:100894. doi: 10.1016/j.ienj.2020.100894 [published Online First: 2020/07/13]
42. Baker E, Clark LL. Biopsychopharmacosocial approach to assess impact of social distancing and isolation on mental health in older adults. *Br J Community Nurs* 2020;25(5):231-38. doi: 10.12968/bjcn.2020.25.5.231

Figure Legends/Caption:

Figure 1: Receiver Operator Curve (ROC) for predictive models for three outcomes of interest

Figure 2: Calibration plot for model using expected and observed probabilities of developing poor outcomes

For peer review only - http://bmjopen.bmj.com/site/about/guidelines.xhtml

Declarations:

Author Contribution:

EB, GL, AX, CN, PH conceived and designed the study and developed the study protocol. AB, CB, EC, CD, JF, RJ, LP, AR, IS, AT, MT, & LY were local primary investigators and coordinated and contributed significantly to the recruitment and data collection processes. Data analysis was planned with ED, GL, TM & CB. Data analysis was undertaken by EB and reviewed by TM. EB developed the first draft of the manuscript and EC, CB, AX, CN, PH, & GL made subsequent revisions. All authors commented on and approved the manuscript prior to submission for publication. EB takes overall responsibility for the manuscript.

Acknowledgements:

The research team would like to acknowledge the study participants who gave up their time to participate in this interview study and the clinical research staff at each site who contributed to the participants recruitment and initial data collection for the Rib Injury Outcomes Study.

Conflict of Interest:

Nothing to declare.

Funding Statement:

Edward Baker is funded by a National Institute for Health Research (NIHR), Clinical Doctoral Research Fellowship for this research project (ICA-CDRF-2016-02-006). The publication presents independent research funded by the National Institute for Health Research (NIHR).

For peer review only

For peer review only - http://bmjopen.bmj.com/site/about/guidelines.xhtml

The views expressed are those of the authors and not necessarily those of the NHS, the NIHR or the Department of Health and Social Care.

Data Statement:

The authors confirm that the data supporting the findings of this study are available within the article and its supplementary materials.

Ethical Approvals:

Ethical approval was granted by the 'Hampshire A' South Central Research Ethics Committee in June 2018 (ref: 18/SC/0230).

For peer review only

For peer review only - http://bmjopen.bmj.com/site/about/guidelines.xhtml

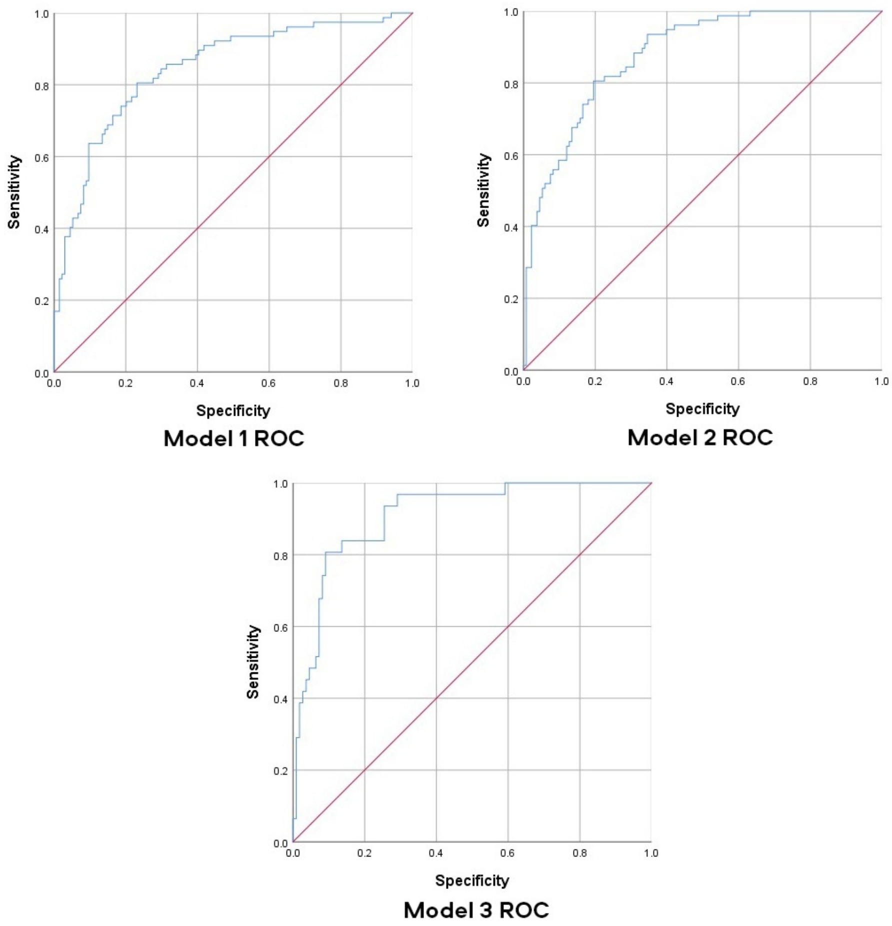

Figure 1: Receiver Operator Curve (ROC) for predictive models for three outcomes of interest

187x187mm (144 x 144 DPI)

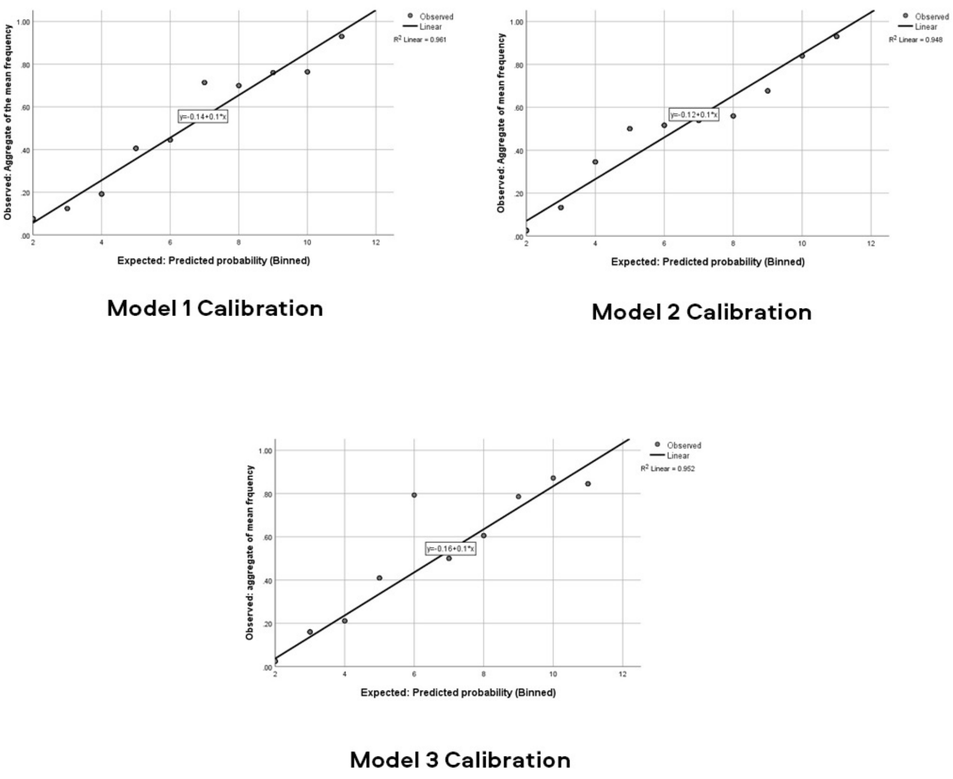

Figure 2: Calibration plots for models using expected and observed probabilities of developing poor outcomes of interest

187x187mm (144 x 144 DPI)

Supplementary file: Table S1: Extended Characteristics of the Sample/Candidate predictor variables

Result: n= (%) unless otherwise indicated	**Total sample** (n=337)	**6/12 PCS≤35** (n=77)	**6/12 PCS>35** (n=134)	p-value	**6/12 Pain Severity Score ≥3.5** (n=77)	**6/12 Pain Severity Score <3.5** (n=134)	p-value	**6/12 PDQ score ≥12** (n=47)	**6/12 PDQ score <12** (n=163)	p-value
Age (Mean (±SD))	62.0 (16.5)	69.1 (13.9)	60.8 (16.3)	<0.001*	66.7 (16.4)	62.1 (15.6)	0.05	63.4 (17.0)	63.9 (15.7)	0.80
Male	231 (68.5)	49 (63.6)	97 (72.4)	0.20	54 (70.1)	91 (67.9)	0.80	33 (70.2)	112 (68.7)	0.90
Injury Severity Score (Mean (±SD))	11.2 (7.3)	10.9 (7.8)	11.4 (6.8)	0.60	11.3 (7.9)	11.2 (6.8)	1.0	11.6 (8.3)	11.2 (6.9)	0.80
Battle/StUMBle Score (Mean (±SD))	22.2 (12.0)	23.8 (11.0)	21.8 (11.3)	0.20	23.9 (11.4)	21.8 (11.0)	0.20	24.2 (12.0)	22.1 (10.9)	0.30
**Mechanism of Injury:**										
RTC	78 (23.1)	14 (18.2)	38 (28.4)		19 (24.7)	33 (24.6)		9 (19.1)	43 (26.4)	
Crush	11 (3.3)	3 (3.9)	4 (3.0)		1 (1.3)	6 (4.5)		1 (2.1)	6 (3.7)	
Ped vs. Vehicle	4 (1.2)	1 (1.3)	2 (1.5)		1 (1.3)	2 (1.5)		1 (2.1)	2 (1.2)	
Fall>2m	69 (20.5)	13 (16.9)	30 (22.4)	0.20	18 (23.4)	25 (18.7)	0.80	13 (27.7)	30 (18.4)	0.80
Fall<2m	153 (45.4)	44 (57.1)	54 (40.3)		37 (48.1)	61 (45.5)		22 (46.8)	76 (46.6)	
Assault	14 (4.2)	1 (1.3)	4 (3.0)		1 (1.3)	3 (2.2)		1 (2.1)	3 (1.8)	
CPR	4 (1.2)	1 (1.3)	0		0	1 (0.7)		0	1 (0.6)	
Rapid deceleration	4 (1.2)	0	2 (1.5)		0	2 (1.5)		0	2 (1.2)	
**Pre-morbid state:**										
Respiratory	70 (20.8)	21 (27.3)	21 (15.7)	0.04*	21 (27.3)	20 (14.9)	0.03*	12 (25.5)	29 (17.8)	0.20
Cardiac	147 (43.6)	49 (63.6)	53 (39.6)	0.001*	47 (61.0)	54 (40.3)	0.004*	27 (57.4)	74 (45.4)	0.20
Neuro	50 (14.8)	20 (26.0)	9 (6.7)	<0.001*	19 (24.7)	10 (7.5)	0.001*	10 (21.3)	19 (11.7)	0.09
MSK	89 (26.4)	26 (33.8)	35 (26.1)	0.20	30 (39.0)	31 (23.1)	0.02*	16 (34.0)	45 (27.6)	0.40
Cancer Current	9 (2.7)	4 (5.2)	3 (2.2)	0.08	4 (5.2)	3 (2.2)	0.009*	3 (6.4)	4 (2.5)	0.40
Cancer Historical	26 (7.7)	12 (15.6)	10 (7.5)		14 (18.2)	8 (6.0)		4 (8.5)	18 (11.0)	
DM2	33 (9.8)	9 (11.7)	11 (8.2)	0.40	5 (6.5)	15 (11.2)	0.30	5 (10.6)	15 (9.2)	0.80
No of Comorbidities (Mean (±SD))	2.3 (2.2)	2.3 (0.3)	1.8 (0.2)	<0.001*	3.1 (2.2)	1.9 (2.0)	<0.001*	2.9 (2.5)	2.2 (2.1)	0.07
Chronic Pain	27 (8.0)	11 (14.3)	8 (6.0)	0.042*	13 (16.9)	6 (4.5)	0.003*	7 (14.9)	12 (7.4)	0.10
Regular Analgesic Use	61 (18.1)	23 (29.9)	15 (11.2)	0.001*	30 (39.0)	8 (6.0)	<0.001*	19 (40.4)	19 (11.7)	<0.001*

For peer review only

Smoking Status:										
Current smoker	73 (21.7)	11 (14.3)	21 (15.7)	0.90	10 (13.0)	22 (16.4)	0.07	11 (23.4)	21 (12.9)	0.05
Ex-Smoker	90 (26.7)	25 (32.5)	37 (27.6)		27 (35.1)	34 (25.4)		18 (38.3)	43 (26.4)	
Never smoked	122 (36.2)	30 (39.0)	52 (67.5)		23 (29.9)	59 (44.0)		14 (29.8)	68 (41.7)	
Smoking Status unknown	50 (14.8)	11 (14.3)	23 29.9)		17 (22.1)	17 (12.7)		4 (8.5)	30 (18.4)	
Pack years (Mean (±SD))	22.1 (18.4)	27.6 (14.2)	20.3 (18.8)	0.1	19.9 (14.3)	24.1 (19.2)	0.4	21.2 (14.8)	23.3 (18.9)	0.7
**Indicators of Injury Severity:**										
No. Rib Fractures (Mean (±SD))	5.2 (4.0)	5.3 (3.8)	5.3 (4.0)	0.90	5.4 (3.9)	5.3 (3.9)	0.70	5.6 (4.1)	5.2 (3.8)	0.60
Rib fracture Categories:										
0	19 (5.6)	5 (6.5)	8 (6.0)	0.30	6 (7.8)	7 (5.2)	0.80	1 (2.1)	12 (7.4)	0.70
1-4	160 (47.5)	39 (50.6)	58 (43.3)		33 (42.9)	63 (47.0)		25 (53.2)	71 (43.6)	
5-9	114 (33.8)	22 (28.6)	52 (38.8)		46 (59.7)	28 (20.9)		14 (29.8)	60 (36.8)	
10-15	29 (8.6)	9 (11.7)	9 (6.7)		7 (9.1)	11 (8.2)		5 (10.6)	13 (8.0)	
15+	15 (4.5)	2 (2.6)	7 (5.2)		3 (2.9)	6 (4.5)		2 (4.3)	7 (4.3)	
Other Indicators of Severity:										
Operative Rib Fixation	16 (4.7)	6 (7.8)	3 (2.2)	0.05*	2 (2.6)	7 (5.2)	0.40	3 (6.4)	6 (3.7)	0.40
First Rib fracture	31 (9.2)	6 (7.8)	13 (9.7)	0.60	10 (13.0)	9 (6.7)	0.10	7 (14.9)	12 (7.4)	0.10
Bilateral rib fractures	44 (13.1)	9 (11.7)	19 (14.2)	0.60	11 (14.3)	17 (12.7)	0.80	10 (21.3)	18 (11.0)	0.07
Scapular fracture	27 (8.0)	9 (11.7)	11 (8.2)	0.40	9 (11.7)	11 (8.2)	0.40	4 (8.5)	16 (9.8)	0.90
Clavicle fracture	29 (8.6)	8 (10.4)	14 (10.4)	0.50	8 (10.4)	14 (10.4)	0.50	4 (8.5)	18 (11.0)	0.08
Sternum Fracture	26 (7.7)	4 (5.2)	13 (9.7)	0.30	5 (6.5)	12 (9.0)	0.50	2 (4.3)	15 (9.2)	0.30
Manubrium Fracture	8 (2.4)	1 (1.3)	4 (3.0)	0.40	2 (2.6)	3 (2.2)	0.90	1 (2.1)	4 (2.5)	0.90
Thoracic Process #	31 (9.2)	6 (7.8)	12 (9.0)	0.80	11 (14.3)	7 (5.2)	0.02*	5 (10.6)	13 (8.0)	0.60
No. of Thoracic Process # (Mean (±SD))	3.3 (2.8)	2.2 (1.3)	2.8 (1.4)	0.40	2.8 (1.5)	2.3 (1.2)	0.50	2.5 (1.3)	2.7 (1.5)	0.80
Lung Contusion	76 (22.6)	14 (18.2)	32 (23.9)	0.30	9 (11.7)	36 (26.9)	0.009*	8 (17.0)	37 (22.7)	0.40
Pneumothorax	107 (31.8)	20 (26.0)	50 (37.3)	0.20	20 (26.0)	50 (37.3)	0.20	15 (31.9)	55 (33.7)	0.90
Haemothorax	80 (23.7)	15 (19.5)	38 (28.4)	0.20	14 (18.2)	38 (28.4)	0.90	5 (10.6)	47 (28.8)	0.01*
Massive Haemothorax	4 (1.2)	0	1 (0.7)	0.50	1 (1.3)	0	0.20	0	1 (0.6)	0.60
Haemopneumothorax	45 (13.4)	7 (9.1)	23 (17.2)	0.10	21 (27.3)	8 (6.0)	0.30	4 (8.5)	25 (15.3)	0.20
Radiological Flail	60 (17.8)	13 (16.9)	29 (21.6)	0.40	16 (20.8)	26 (19.4)	0.80	7 (14.9)	35 (21.5)	0.30
Extra-thoracic injuries	104 (30.9)	25 (32.5)	35 (26.1)	0.30	19 (24.7)	40 (29.9)	0.40	12 (25.5)	47 (28.8)	0.70

For peer review only

**Initial Vital Signs:**										
Respiratory Rate (Mean (±SD))	19.6 (±4.6)	20.6 (4.8)	18.9 (4.1)	0.01*	20.1 (5.1)	19.2 (4.0)	0.20	20.2 (4.5)	19.3 (4.4)	0.30
Oxygen Saturation (%) (Mean (±SD))	95.9 (4.7)	95.4 (4.4)	96.4 (3.6	0.10	95.6 (3.1)	96.3 (4.3)	0.20	95.8 (3.1)	96.1 (4.1)	0.70
Heart Rate (Mean (±SD))	81.5 (19.3)	81.8 (4.1)	79.4 (17.3)	0.40	82.5 (22.1)	79.0 (17.3)	0.20	83.9 (17.2)	79.3 (19.6)	0.10
Systolic Blood Pressure (Mean (±SD))	142.5 (23.0)	144.8 (23.9)	141.7 (22.3)	0.40	147.9 (25.8)	140.2 (20.7)	0.03*	148.7 (25.3)	141.3 (22.0)	0.09
Temperature (Mean (±SD))	36.6 (4.2)	37.4 (7.5)	36.5 (0.7)	0.30	37.4 (7.7)	36.5 (0.7)	0.20	36.4 (0.5)	37.0 (5.3)	0.20
**Chest Drain Management:**										
Intercostal Drain	67 (19.9)	14 (18.2)	33 (24.6)	0.30	14 (18.2)	32 (23.9)	0.30	9 (19.1)	37 (22.7)	0.60
Duration of Intercostal Drain (days) (Mean (±SD))	4.9 (3.2)	4.6 (2.6)	5.0 (3.8)	0.70	6.5 (4.7)	4.4 (2.9)	0.20	4.7 (1.4)	5.0 (3.8)	0.70
**Admission Level of Care (1-3):**										
Level 1	252 (74.8)	59 (76.6)	106 (79.1)		60 (77.9)	104 (77.6)		35 (74.5)	129 (79.1)	
Level 2	65 (19.3)	13 (16.9)	26 (19.4)	0.10	13 (16.9)	26 (19.4)	0.50	9 (19.1)	30 (18.4)	0.40
Level 3	18 (5.3)	5 (6.5)	2 (1.5)		4 (5.2)	3 (2.2)		3 (6.4)	4 (2.5)	
**Initial Emergency Department analgesic management:**										
None	28 (8.3)	6 (7.8)	14 (10.4)		6 (7.8)	14 (10.4)		2 (4.3)	18 (11.0)	
Opioid	207 (61.4)	47 (61.0)	79 (59.0)	0.80	42 (54.5)	83 (61.9)	0.30	27 (57.4)	98 (60.1)	0.20
Non-opioid	102 (30.3)	24 (31.2)	41 (30.6)		29 (37.7)	36 (26.9)		18 (38.3)	47 (28.8)	
**Inpatient Analgesic management:**										
Intermittent analgesia only	179 (53.1)	37 (48.1)	77 (57.5)	0.20	37 (48.1)	76 (56.7)	0.20	22 (46.8)	91 (55.8)	0.30
Regional Analgesia	106 (31.5)	29 (37.7)	41 (30.6)	0.30	31 (40.3)	39 (29.1)	0.10	20 (42.6)	50 (30.7)	0.10
Thoracic Epidural	50 (14.8)	13 (16.9)	23 (17.2)	0.90	14 (18.2)	22 (16.4)	0.80	8 (17.0)	28 (17.2)	1.0
Time to commence TE (hrs) (Mean (±SD))	27.7 (53.0)	23.2 (26.5)	37.2 (76.9)	0.50	53.5 (92.6)	18.3 (26.0)	0.10	15.1 (14.9)	36.9 (69.8)	0.20
Paravertebral block	16 (4.7)	5 (6.5)	5 (3.7)	0.40	8 (10.4)	2 (1.5)	0.004*	4 (8.5)	6 (3.7)	0.20
Time to commence PVB (hrs) (Mean (±SD))	20.4 (17.3)	11.8 (8.8)	29.3 (27.7)	0.40	12.5 (8.6)	-	-	-	-	-
Intercostal Nerve Block	4 (1.2)	3 (3.9)	0	0.02	1 (1.3)	2 (1.5)	0.90	1 (2.1)	2 (1.2)	0.50
Time to commence Intercostal Nerve Block (hrs) (Mean (±SD))	65.3 (57.6)	39.0 (28.9)	-	-	-	-	-	-	-	-
Serratus Plane Block	22 (6.5)	4 (5.2)	14 (10.4)	0.5	4 (5.2)	10 (7.5)	0.50	2 (4.3)	12 (7.4)	0.70

For peer review only

Time to commence SPB (hrs) (Mean (±SD))	17.6 (19.5)	34.8 (36.9)	13.6 (8.5)	0.10	17.6 (6.4)	20.4 (25.5)	0.80	14.3 (2.5)	20.5 (23.2)	0.40
Erector Spinae Block	20 (5.9)	6 (7.8)	4 (3.0)	0.10	7 (9.1)	3 (2.2)	0.03*	5 (10.6)	5 (3.1)	0.03*
Time to commence Erector Spinae Block (hrs) (Mean (±SD))	30.4 (19.8)	25.8 (29.5)	29.5 (27.3)	1.0	27.0 (22.5)	25.8 (-)	1.0	31.0 (32.0)	24.4 (24.7)	0.60
Combined Region Analgesia with Patient Controlled Analgesic	43 (12.8)	11 (14.3)	14 (10.4)	0.40	10 (13.0)	15 (11.2)	0.70	6 (12.8)	19 (11.7)	0.80
Patient Controlled Analgesic only	93 (27.6)	23 (29.9)	29 (21.6)	0.20	20 (26.0)	32 (23.9)	0.80	12 (25.5)	40 (24.5)	0.90
Time to commence PCA (hrs) (Mean (±SD))	16.2 (23.9)	12.4 (10.9)	12.7 (12.4)	0.90	12.8 (12.2)	12.5 (11.6)	0.90	14.1 (16.3)	12.1 (10.0)	0.70
**Other Analgesics:**										
Gabapentin	37 (11.0)	11 (14.3)	11 (8.2)	0.20	11 (14.3)	11 (8.2)	0.20	9 (19.1)	13 (8.0)	0.03*
Pregabalin	15 (4.5)	5 (6.5)	4 (3.0)	0.20	7 (9.1)	2 (1.5)	0.009*	6 (12.8)	3 (1.8)	0.001*
Amitriptyline	16 (4.7)	7 (9.1)	3 (2.2)	0.02*	7 (9.1)	3 (2.2)	0.03*	5 (10.6)	5 (3.1)	0.03*
Transdermal Lignocaine	113 (33.5)	28 (36.4)	43 (32.1)	0.50	27 (35.1)	44 (32.8)	0.80	12 (25.5)	59 (36.2)	0.20
**Discharge Analgesia:**										
Opioid	220 (65.3)	51 (66.2)	95 (70.9)	0.48	55 (71.4)	90 (67.2)	0.60	33 (70.2)	112 (68.7)	0.90
Neuropathic Pain Agent	41 (12.2)	14 (18.2)	11 (8.2)	0.03*	12 (15.6)	13 (9.7)	0.20	10 (21.3)	15 (9.2)	0.02*
NSAIDS	88 (26.1)	13 (16.9)	37 (27.6)	0.08	19 (24.7)	31 (23.1)	0.80	14 (29.8)	36 (22.1)	0.30
Transdermal Lignocaine	63 (18.7)	15 (19.5)	24 (17.9)	0.80	16 (20.8)	23 (17.2)	0.50	8 (17.0)	31 (19.0)	0.80
No discharge analgesics prescribed	34 (10.1)	7 (9.1)	9 (6.7)	0.50	6 (7.8)	10 (7.5)	0.90	5 (10.6)	11 (6.7)	0.40
Number of discharge analgesic agents (Mean (±SD))	2.1 (1.3)	2.1 (1.3)	2.1 (1.2)	0.80	2.2 (1.3)	2.1 (1.2)	0.70	2.3 (1.4)	2.1 (1.2)	0.30
**Complications:**	111 (32.9)	34 (44.2)	33 (24.6)	0.003*	25 (32.5)	42 (31.3)	0.90	18 (38.3)	49 (30.1)	0.30
Type 1 Respiratory Failure	18 (5.4)	2 (2.6)	4 (3.0)	0.90	2 (2.6)	4 (3.0)	0.90	2 (4.3)	4 (2.5)	0.60
Type 2 Respiratory Failure	10 (3.0)	5 (6.5)	1 (0.7)	0.02*	1 (1.3)	5 (3.7)	0.30	4 (8.5)	2 (1.2)	0.02*
Pulmonary Embolism	4 (1.2)	0	1 (0.7)	0.50	0	1 (0.7)	0.50	0	1 (0.6)	1.0
Pneumonia	42 (12.5)	13 (16.9)	12 (9.0)	0.09	11 (14.3)	14 (10.4)	0.40	8 (17.0)	17 (10.4)	0.20
Ventilator Associated Pneumonia	5 (1.5)	3 (3.9)	1 (0.7)	0.10	2 (2.6)	2 (1.5)	0.60	2 (4.3)	2 (1.2)	0.20
ARDS	2 (0.6)	1 (1.3)	0	0.20	0	1 (0.7)	0.50	0	1 (0.6)	1.0

For peer review only

LRTI	58 (17.2)	16 (20.8)	19 (14.2)	0.20	11 (14.3)	24 (17.9)	0.50	7 (14.9)	28 (17.2)	0.70
Treated with Abx	110 (32.6)	29 (37.7)	38 (28.4)	0.20	22 (28.6)	45 (33.6)	0.40	15 (31.9)	52 (31.9)	1.0
**Post Admission Ventilation:**	26 (7.7)	9 (11.7)	5 (3.7)	0.03*	6 (7.8)	8 (6.0)	0.60	5 (10.6)	9 (5.5)	0.20
CPAP	4 (1.2)	2 (2.6)	0	0.06	0	2 (1.5)	0.50	0	2 (1.2)	1.0
BiPAP	1 (0.3)	1 (1.3)	0	0.20	0	1 (0.7)	1.0	0	1 (0.6)	1.0
Invasive Ventilation	17 (5.0)	7 (9.1)	2 (1.5)	0.01*	3 (3.9)	6 (4.5)	1.0	2 (4.3)	7 (4.3)	1.0
High Flow nasal Oxygen	19 (5.6)	6 (7.8)	5 (3.7)	0.20	5 (6.5)	6 (4.5)	0.50	4 (8.5)	7 (4.3)	0.30
Moved to Critical Care	29 (8.6)	10 (13.0)	7 (5.2)	0.05*	6 (7.8)	11 (8.2)	0.90	5 (10.6)	12 (7.3)	0.50
ICU Length of Stay (Mean (±SD))	7.6 (7.4)	9.42 (7.5)	6.42 (5.8)	0.20	7.6 (5.4)	7.8 (7.3)	0.90	9.4 (7.2)	7.4 (6.6)	0.50
Hospital Length of Stay (Mean (±SD))	8.9 (9.5)	11.7 (10.2)	6.9 (5.7)	<0.001*	10.4 (9.3)	7.7 (7.0)	0.03*	9.9 (7.2)	8.3 (8.2)	0.2
**Discharge outcome:**										
Home	306 (90.8)	68 (88.3)	128 (95.5)	0.01*	70 (90.9)	125 (93.3)	0.5	43 (91.5)	152 (93.3)	0.3
Rehabilitation Bed	9 (2.7)	6 (7.8)	0		3 (3.9)	3 (2.2)		3 (6.4)	3 (1.8)	
Repatriation to local hospital	15 (4.5)	3 (3.9)	5 (3.7)		3 (3.9)	5 (3.7)		1 (2.1)	7 (4.3)	
Long term care	4 (1.2)	0	1 (0.7)		1 (1.3)	0		0	1 (0.6)	

For peer review only

Supplementary Table S2: Comparison of characteristics of responders and non-responders

Significance denoted by *<br>Result= n=(%) unless otherwise indicated	Responded at 6 months (n=211)	Non-respondent at 6 months (n=126)	P-value
Age (Mean (SD))	63.8 (16.0)	59.0 (17.0)	0.01*
Male (%)	146 (69.5)	85 (66.9)	0.62
Injury Severity Score (Mean (SD))	11.3 (7.2)	11.1 (7.6)	0.86
Battle/StUMBle Score (Mean (SD))	22.5 (11.2)	21.5 (13.3)	0.48
**Pre-morbid state:**			
Respiratory	42 (20.0)	28 (22.0)	0.65
Cardiac	102 (48.6)	45 (35.4)	0.02*
Neuro	28 (13.3)	22 (17.3)	0.32
MSK	60 (28.6)	29 (22.8)	0.25
Cancer Current	7 (3.3)	2 (1.6)	0.03*
Cancer Historical	22 (10.5)	4 (3.1)	
DM2	20 (9.5)	13 (10.2)	0.81
No of Comorbidities (Mean (SD))	2.3 (2.2)	2.3 (2.2)	0.86
Chronic Pain	19 (9.0)	8 (6.3)	0.37
Regular Analgesic Use	38 (18.1)	23 (18.1)	1.0
**Indicators or Injury severity:**			
No. Rib Fractures (Mean (SD))	5.3 (3.9)	5.0 (4.2)	0.56
Operative Rib Fixation	9 (4.3)	7 (5.5)	0.61
First Rib fracture	19 (9.0)	12 (9.4)	0.90
Bilateral rib fractures	28 (13.3)	16 (12.6)	0.85
**Admission Level of Care (1-3):**			
Level 1	164 (78.1)	88 (69.3)	
Level 2	39 (18.6)	26 (20.5)	0.07
Level 3	7 (3.3)	11 (8.7)	
**Complications:**	66 (31.4)	45 (35.4)	0.39
Type 1 Respiratory Failure	6 (2.9)	12 (9.4)	0.008
Type 2 Respiratory Failure	6 (2.9)	4 (3.1)	0.86
Pulmonary Embolism	1 (0.5)	3 (2.4)	0.12
Pneumonia	25 (11.9)	17 (13.4)	0.65
Ventilator Associated Pneumonia	4 (1.9)	1 (0.8)	0.42
ARDS	1 (0.5)	1 (0.8)	0.71
LRTI	34 (16.2)	24 (18.9)	0.55
Treated with Abx	66 (31.4)	44 (34.6)	0.48
Post Admission Ventilation:	14 (6.7)	12 (9.4)	0.33
ICU Length of Stay (Mean (SD))	7.7 (6.7)	7.4 (8.2)	0.84
Hospital Length of Stay (Mean (SD))	8.6 (8.0)	9.5 (11.7)	0.48
**Discharge outcome:**			
Home	195 (92.9)	111 (87.4)	
Rehabilitation Bed	6 (2.9)	3 (2.4)	0.47
Repatriation to local hospital	8 (3.8)	7 (5.5)	
Long term care	1 (0.5)	3 (2.4)	
**Health Related Quality of Life:**			
Pre-injury SF-12 PCS (Mean (SD))	46.8 (10.4)	45.9 (12.0)	0.44
Pre-injury SF-12 MCS (Mean (SD))	51.2 (9.5)	47.1 (12.1)	0.001*
Discharge SF-12 PCS (Mean (SD))	26.9 (4.4)	27.7 (9.0)	0.43
Discharge SF-12 MCS (Mean (SD))	45.8 (11.6)	41.9 (11.9)	0.004*
1-month SF-12 PCS (Mean (SD))	31.2 (7.67)	33.8 (8.27)	0.09
1-month SF-12 MCS (Mean (SD))	44.8 (11.6)	39.5 (11.4)	0.02*
3-month SF-12 PCS (Mean (SD))	40.2 (11.1)	35.8 (4.03)	0.19
3-month SF-12 MCS (Mean (SD))	48.3 (11.5)	45.6 (9.51)	0.68

For peer review only

Supplementary File: Table S3: Extended Outcomes of interest measured at 6-months after discharge (The total sample)

Time-point	PROM	Min	Max	Mean (±SD)	Dif. from Pop. Norms	Pop Norm
Pre-Injury	SF-12 Physical Component Score	13.1	64.9	46.5 (11.0)	-3.5	50.0 (SD 1.0)
	SF-12 Mental Component Score	13.5	67.2	49.7 (10.7)	-0.3	50.0 (SD 1.0)
	EQ Index Score	-0.3	1.0	0.7 (0.3)	-0.2	0.9 (SE 0.004)
	EQ VAS Score	10.0	100.0	74.9 (19.7)	-7.9	82.8 (SE 0.4)
Discharge	SF-12 Physical Component Score	10.9	55.5	27.2 (8.6)	-22.8	50.0 (SD 1.0)
	SF-12 Mental Component Score	15.9	68.9	44.4 (11.8)	-5.6	50.0 (SD 1.0)
	EQ Index Score	-0.5	1.0	0.3 (0.3)	-0.6	0.9 (SE 0.004)
	EQ VAS Score	0	99.0	47.4 (23.8)	35.4	82.8 (SE 0.4)
	BPI Pain Severity Score	0	10.0	5.0 (2.1)	-	-
	BPI Pain Interference Score	0	10.0	5.7 (2.3)	-	-
	BPI Analgesic Effectiveness	0	100.0	65.3 (24.8)	-	-
	PainDetect Score	-1	29	10.3 (6.0)	-	-
	Prop. reporting Thoracic Pain (%)	-	-	283 (88.4)	-	-
	Mean No. affected body regions (21)	0	16.0	2.5 (2.1)	-	-
1-month	SF-12 Physical Component Score	14.8	55.7	31.6 (7.8)	-18.4	50.0 (SD 1.0)
	SF-12 Mental Component Score	18.4	66.3	43.9 (11.8)	-6.1	50.0 (SD 1.0)
	EQ Index Score	-0.5	1.0	0.5 (0.3)	-0.4	0.9 (SE 0.004)
	EQ VAS Score	5.0	100.0	60.0 (22.7)	-22.8	82.8 (SE 0.4)
	BPI Pain Severity Score	0	10.0	3.8 (2.2)	-	-
	BPI Pain Interference Score	0	10.0	4.4 (2.7)	-	-
	BPI Analgesic Effectiveness	0	100.0	48.4 (30.0)	-	-
	PainDetect Score	0	35.0	8.2 (7.5)	-	-
	Prop. reporting Thoracic Pain (%)	-	-	162 (77.1)	-	-
	Mean No. affected body regions (21)	0	12	3.0 (2.3)	-	-
3-months	SF-12 Physical Component Score	13.3	63.5	40.1 (11.0)	-9.9	50.0 (SD 1.0)
	SF-12 Mental Component Score	19.1	66.8	48.3 (11.4)	-1.7	50.0 (SD 1.0)
	EQ Index Score	-0.5	1.0	0.7 (0.3)	-0.2	0.9 (SE 0.004)
	EQ VAS Score	0	100.0	68.9 (24.4)	-13.9	82.8 (SE 0.4)
	BPI Pain Severity Score	0	9.8	2.9 (2.6)	-	-
	BPI Pain Interference Score	0	9.9	2.6 (2.7)	-	-
	BPI Analgesic Effectiveness	0	100.0	46.5 (35.8)	-	-
	PainDetect Score	0	32.0	8.2 (7.5)	-	-
	Prop. reporting Thoracic Pain (%)	-	-	79 (54.9)	-	-
	Mean No. affected body regions (21)	0	20.0	2.6 (2.7)	-	-
6-months	SF-12 Physical Component Score	12.3	62.1	41.7 (11.5)	-8.3	50.0 (SD 1.0)
	SF-12 Mental Component Score	19.1	65.8	47.8 (11.8)	-2.2	50.0 (SD 1.0)
	EQ Index Score	-0.3	1.0	0.7 (0.3)	-0.2	0.9 (SE 0.004)
	EQ VAS Score	0	100.0	69.2 (26.5)	-13.6	82.8 (SE 0.4)
	BPI Pain Severity Score	0	10.0	2.7 (2.7)	-	-
	BPI Pain Interference Score	0	10.0	2.8 (2.9)	-	-
	BPI Analgesic Effectiveness	0	100.0	49.2 (33.4)	-	-
	PainDetect Score	-1	33	7.5 (7.4)	-	-
	Prop. reporting Thoracic Pain (%)	-	-	99 (46.9)	-	--
	Mean No. affected body regions (21)	0	21	2.6 (3.2)	-	

For peer review only

Supplemental File: Table S4: Extended Outcomes of Interest Data/Candidate predictor variables at six-months after discharge

Mean (±SD)	Outcome Data:	PCS <35 (n=76)	PCS>35 (n=135)	p-value	PSS ≥3.5 (n=77)	PSS <3.5 (n=134)	p-value	PDQ >12 (n=47)	PDQ <12 (n=164)	p-value
Pre-Injury	PCS	40.0 (11.0)	50.8 (7.7)	<0.001*	41.9 (11.4)	49.6 (8.6)	<0.001*	41.7 (11.5)	48.3 (9.6)	<0.001*
	MCS	48.2 (11.7)	53.1 (7.6)	<0.001*	49.1 (10.9)	52.7 (8.4)	0.009*	48.2 (10.6)	52.3 (9.1)	0.009*
	Prop. PCS<20 (n=3) (%)	3 (100)	0	0.022*	3 (100)	0	0.021*	1 (2.2)	2 (1.2)	0.63
	Prop. PCS <30 (n= 22) (%)	17 (77.3)	5 (22.7)	<0.001*	17 (22.4)	5 (3.8)	<0.001*	11 (23.9)	11 (6.7)	0.001*
	Prop. PCS <35 (n=37) (%)	30 (81.1)	7 (18.9)	<0.001*	23 (30.3)	14 (10.5)	<0.001*	16 (34.8)	21 (12.9)	0.001*
Discharge	PCS	24.3 (7.4)	28.4 (8.6)	0.001*	25.1 (8.3)	27.9 (8.3)	0.02*	25.1 (8.4)	27.5 (8.3)	0.09
	MCS	43.5 (11.8)	47.0 (11.5)	0.04*	46.1 (11.5)	45.5 (11.9)	0.76	42.6 (11.7)	46.7 (11.6)	0.04*
	Prop. PCS<20 (n=54) (%)	28 (37.3))	26 (20.0)	0.007*	27 (36.5)	27 (20.8)	0.014*	19 (40.4)	35 (22.3)	0.013*
	Prop. PCS <30 (n=150) (%)	64 (85.3))	86 (66.2)	0.003*	59 (79.7)	90 (69.2)	0.10	39 (83.0)	110 (70.1)	0.08
	Prop. PCS <35 (n=154) (%)	65 (56.7)	89 (68.5)	0.004*	61 (82.4)	93 (71.5)	0.082	36 (76.6)	118 (75.2)	0.84
	BPI Worst Pain	7.4 (2.0)	7.1 (2.2)	0.30	7.3 (2.0)	7.1 (2.3)	0.40	7.5 (1.8)	7.1 (2.2)	0.20
	BPI Least Pain	3.5 (2.7)	3.2 (2.6)	0.40	3.7 (2.7)	3.0 (2.6)	0.06	3.9 (2.6)	3.0 (2.6)	0.04*
	BPI Average Pain	5.4 (2.1)	5.1 (2.0)	0.30	5.4 (2.0)	5.1 (2.1)	0.40	6.7 (2.0)	5.1 (2.1)	0.08
	BPI Current Pain	4.7 (2.8)	3.8 (2.6)	0.01*	4.7 (2.8)	3.8 (2.5)	0.02*	5.3 (2.7)	3.8 (2.6)	0.001*
	BPI Pain Severity Score	5.3 (2.1)	4.8 (2.0)	0.10	5.3 (2.0)	4.7 (2.0)	0.06	5.6 (2.0)	4.7 (2.0)	0.009*
	Prop. PSS >3.5 (n=157) (%)	60 (81.1)	97 (74.6)	0.30	59 (80.8)	97 (74.6)	0.30	40 (87.0)	116 (73.9)	0.07
	Analgesic Effectiveness	57.8 (27.4)	68.4 (22.8)	0.003*	63.3 (26.2)	65.8 (23.8)	0.50	64.3 (23.3)	65.1 (25.1)	0.90
	General Activities	7.8 (2.6)	6.6 (3.0)	0.006*	7.3 (2.7)	6.9 (3.0)	0.30	7.4 (2.8)	6.9 (3.0)	0.30
	Mood	5.5 (3.2)	4.0 (3.2)	0.002*	4.6 (3.0)	4.4 (3.5)	0.70	5.3 (3.2)	4.2 (3.3)	0.05
	Walking	6.9 (3.0)	4.7 (3.1)	<0.001*	6.4 (3.2)	5.0 (3.2)	0.004*	6.8 (3.0)	5.1 (3.2)	0.001*
	Normal Work	8.6 (2.3)	7.4 (3.3)	0.005*	8.4 (2.5)	7.5 (3.3)	0.04*	8.3 (2.4)	7.7 (3.2)	0.20
	Relations	3.6 (3.7)	2.5 (2.9)	0.02*	3.1 (3.3)	2.7 (3.2)	0.50	3.9 (3.5)	2.5 (3.1)	0.02*
	Sleep	6.0 (3.3)	2.3 (3.3)	0.20	5.6 (3.5)	5.5 (3.2)	0.70	6.4 (3.0)	5.3 (3.4)	0.03*
	Enjoyment	7.0 (3.1)	3.0 (3.5)	0.04*	6.2 (3.4)	6.4 (3.4)	0.70	3.9 (3.0)	6.2 (3.5)	0.20
	BPI Pain Interference Score	6.5 (2.0)	5.2 (2.4)	<0.001*	5.9 (2.0)	5.5 (2.4)	0.18	6.4 (2.0)	5.4 (2.3)	0.004*
	PDQ: Low Risk (n=143) (%)	48 (64.9)	95 (73.1)	0.42	47 (63.5)	96 (74.4)	0.08	24 (52.2)	119 (75.8)	0.006*
	PDQ: Moderate Risk (n=44) (%)	18 (24.3)	26 (20.0)		22 (29.7)	21 (16.3)		17 (37.0)	26 (16.6)	
	PDQ: High Risk (n=17) (%)	8 (10.8)	9 (6.9)		5 (6.8)	12 (9.3)		5 (10.9)	12 (7.6)	
1-month	PCS	27.0 (6.0)	33.5 (7.5)	<0.001*	27.7 (6.0)	32.9 (7.8)	<0.001*	27.8 (5.8)	32.1 (7.9)	<0.001*
	MCS	40.8 (12.2)	46.8 (11.2)	0.001*	4.07 (12.7)	46.7 (10.9)	0.001*	35.6 (12.3)	47.0 (10.6)	<0.001*

	Prop. PCS<20 (n=10) (%)	7 (10.9)	3 (2.7)	0.022*	6 (10.0)	4 (3.4)	0.073	3 (8.1)	7 (5.0)	0.47
	Prop. PCS <30 (n=95) (%)	50 (78.1)	45 (39.8)	<0.001*	42 (70.0)	53 (45.3)	0.002*	26 (70.3)	69 (49.3)	0.023*
	Prop. PCS <35 (n=130) (%)	60 (93.8)	70 (61.9)	<0.001*	52 (86.7)	78 (66.7)	0.004*	33 (89.2)	97 (69.3)	0.015*
	BPI Worst Pain	5.7 (2.5)	4.5 (2.3)	0.002*	6.1 (2.2)	4.4 (2.3)	<0.001*	6.6 (2.3)	4.5 (2.3)	<0.001*
	BPI Least Pain	3.6 (2.4)	2.5 (2.2)	0.003*	4.4 (2.3)	2.2 (2.0)	<0.001*	4.5 (2.3)	2.5 (2.1)	<0.001*
	BPI Average Pain	4.8 (2.3)	3.5 (2.0)	<0.001*	5.3 (2.0)	3.2 (2.0)	<0.001*	5.7 (2.2)	3.5 (2.0)	<0.001*
	BPI Current Pain	4.5 (2.6)	2.5 (2.2)	<0.001*	5.0 (2.4)	2.4 (2.1)	<0.001*	5.4 (2.6)	2.7 (2.2)	<0.001*
	BPI Pain Severity Score	4.7 (2.3)	3.2 (1.9)	<0.001*	5.2 (2.0)	3.0 (1.9)	<0.001*	5.5 (2.2)	3.3 (1.9)	<0.001*
	Prop. PSS >3.5 (n=101) (%)	45 (71.4)	56 (49.6)	0.005*	49 (83.1)	52 (44.4)	<0.001*	31 (86.1)	70 (50.0)	<0.001*
	Analgesic Effectiveness	50.0 (24.8)	48.2 (32.7)	0.70	49.7 (23.6)	48.4 (33.1)	0.8	50.0 (27.2)	48.6 (30.8)	0.80
	General Activities	6.1 (2.9)	3.9 (2.6)	<0.001*	6.2 (2.8)	3.9 (2.7)	<0.001*	6.7 (2.8)	4.2 (2.7)	<0.001*
	Mood	4.5 (3.2)	3.1 (2.9)	0.006*	5.0 (3.0)	2.9 (2.9)	<0.001*	5.8 (3.2)	3.0 (2.8)	<0.001*
	Walking	5.8 (3.1)	3.1 (3.0)	<0.001*	5.9 (3.0)	3.1 (3.1)	<0.001*	6.1 (3.2)	3.5 (3.1)	<0.001*
	Normal Work	6.8 (3.1)	4.4 (3.1)	<0.001*	6.8 (2.9)	4.5 (3.2)	<0.001*	7.4 (2.7)	4.7 (3.2)	<0.001*
	Relations	4.0 (3.5)	2.4 (3.0)	0.001*	4.2 (3.3)	2.3 (3.1)	<0.001*	5.3 (3.1)	2.4 (3.0)	<0.001*
	Sleep	5.9 (3.2)	4.8 (3.1)	0.04*	6.7 (2.8)	4.5 (3.1)	<0.001*	7.6 (2.5)	4.6 (3.0)	<0.001*
	Enjoyment	6.2 (3.3)	4.1 (3.1)	<0.001*	6.2 (3.1)	4.2 (3.3	<0.001*	7.0 (3.0)	4.3 (3.2)	<0.001*
	BPI Pain Interference Score	5.6 (2.6)	3.7 (2.5)	<0.001*	5.8 (2.4)	3.6 (2.5)	<0.001*	6.6 (2.4)	3.8 (2.4)	<0.001*
	PDQ: Low Risk (n=122) (%)	39 (61.9)	83 (73.5)	0.10	34 (57.6)	88 (75.2)	0.005*	14 (38.9)	108 (77.1)	<0.001*
	PDQ: Moderate Risk (n=36) (%)	14 (22.2)	22 (19.5)		13 (22.0)	23 (19.7)		9 (25.0)	27 (19.3)	
	PDQ: High Risk (n=18) (%)	10 (15.9)	8 (7.1)		12 (20.3)	6 (5.1)		13 (36.1)	5 (3.6)	
3-months	PCS	31.4 (8.8)	44.4 (9.5)	<0.001*	32.4 (9.1)	43.9 (10.0)	<0.001*	32.9 (8.8)	42.1 (10.8)	<0.001*
	MCS	43.1 (12.9)	50.9 (9.7)	<0.001*	43.1 (11.8)	51.0 (10.4)	<0.001*	40.0 (12.0)	50.7 (10.2)	<0.001*
	Prop. PCS<20 (n=4) (%)	3 (6.4)	1 (1.1)	0.07	3 (6.4)	1 (1.1)	0.071	1 (3.2)	3 (2.7)	0.88
	Prop. PCS <30 (n=32) (%)	24 (51.1)	8 (8.4)	<0.001*	23 (48.9)	9 (9.5)	<0.001*	14 (45.2)	18 (16.2)	0.001*
	Prop. PCS <35 (n=56) (%)	35 (74.5)	21 (22.1)	<0.001*	34 (74.5)	22 (23.2)	<0.001*	21 (67.7)	35 (31.5)	<0.001*
	BPI Worst Pain	5.6 (2.8)	2.9 (2.5)	<0.001*	6.2 (2.2)	2.6 (2.4)	<0.001*	5.9 (2.2)	3.2 (2.8)	<0.001*
	BPI Least Pain	3.4 (2.9)	1.7 (2.3)	0.001*	4.4 (2.7)	1.2 (1.9)	<0.001*	4.0 (2.7)	1.8 (2.4)	<0.001*
	BPI Average Pain	4.6 (2.8)	2.3 (2.3)	<0.001*	5.3 (2.4)	2.0 (2.0)	<0.001*	5.0 (2.4)	2.5 (2.5)	<0.001*
	BPI Current Pain	4.1 (3.2)	1.8 (2.3)	<0.001*	4.7 (2.7)	1.5 (2.2)	<0.001*	4.4 (2.8)	2.0 (2.7)	<0.001*
	BPI Pain Severity Score	4.4 (2.7)	2.2 (2.2)	<0.001*	5.1 (2.2)	1.8 (2.0)	<0.001*	4.8 (2.2)	2.4 (2.4)	<0.001*
	Prop. PSS >3.5 (n=53) (%)	27 (58.7)	26 (27.4)	<0.001*	35 (74.5)	18 (19.1)	<0.001*	23 (74.2)	30 (27.3)	<0.001*
	Analgesic Effectiveness	40.0 (32.1)	50.1 (37.6)	0.10	48.5 (29.6)	44.5 (40.0)	0.60	48.7 (31.3)	45.3 (37.5)	0.70
	General Activities	4.8 (3.5)	2.1 (2.6)	<0.001*	5.7 (3.0)	1.6 (2.2)	<0.001*	5.6 (3.0)	2.2 (2.8)	<0.001*

	Mood	4.0 (3.5)	1.5 (2.3)	<0.001*	4.6 (3.1)	1.2 (2.2)	<0.001*	5.1 (3.1)	1.6 (2.5)	<0.001*
	Walking	4.9 (3.8)	1.4 (2.4)	<0.001*	5.0 (3.4)	1.4 (2.6)	<0.001*	4.4 (3.3)	2.1 (3.2)	0.001*
	Normal Work	5.4 (3.7)	2.0 (2.7)	<0.001*	5.7 (3.2)	1.8 (2.7)	<0.001*	5.6 (3.2)	2.5 (3.2)	<0.001*
	Relations	2.5 (3.0)	1.1 (2.1)	0.001*	3.2 (3.0)	0.8 (1.7)	<0.001*	3.8 (3.2)	1.0 (1.8)	<0.001*
	Sleep	4.2 (3.4)	2.2 (2.7)	<0.001*	4.9 (3.1)	1.9 (2.5)	<0.001*	5.1 (3.2)	2.3 (2.7)	<0.001*
	Enjoyment	5.0 (3.7)	2.0 (2.6)	<0.001*	5.7 (3.4)	1.7 (2.4)	<0.001*	5.3 (3.6)	2.4 (3.0)	<0.001*
	BPI Pain Interference Score	4.4 (3.1)	1.8 (2.1)	<0.001*	5.0 (2.6)	1.5 (2.0)	<0.001*	5.0 (2.6)	2.0 (2.4)	<0.001*
	PDQ: Low Risk (n=107) (%)	28 (60.9)	79 (83.2)		23 (48.9)	84 (89.4)		9 (29.0)	98 (89.1)	
	PDQ: Moderate Risk (n=15) (%)	7 (15.2)	8 (8.4)	0.012*	9 (19.1)	6 (6.4)	<0.001*	9 (29.0)	6 (5.5)	<0.001*
	PDQ: High Risk (n=19) (%)	11 (23.9)	8 (8.4)		15 (31.9)	4 (4.3)		13 (41.9)	6 (5.5)	
6-months	PCS	29.2 (5.1)	48.9 (7.2)	<0.001*	32.9 (8.0)	46.8 (10.1)	<0.001*	32.4 (8.5)	44.4 (10.9)	<0.001*
	MCS	42.3 (13.2)	51.0 (9.7)	<0.001*	39.4 (11.6)	52.7 (8.9)	<0.001*	36.4 (11.8)	51.1 (9.6)	<0.001*
	Prop. PCS<20 (n=4) (%)	-	-	-	3 (4.0)	1 (0.7)	0.1	3 (6.4)	1 (0.6)	0.01*
	Prop. PCS <30 (n=42) (%)	-	-	-	30 (39.0)	12 (9.0)	<0.001*	20 (42.6)	22 (13.4)	<0.001*
	Prop. PCS <35 (n=76) (%)	-	-	-	51 (66.2)	25 (18.8)	<0.001*	32 (68.1)	44 (26.8)	<0.001*
	BPI Worst Pain	5.8 (4.5)	2.1 (2.4)	<0.001*	6.5 (1.8)	1.6 (2.0)	<0.001*	6.3 (2.2)	2.6 (2.7)	<0.001*
	BPI Least Pain	4.1 (3.0)	1.2 (2.0)	<0.001*	5.2 (2.4)	0.6 (0.9)	<0.001*	4.9 (2.8)	1.5 (2.2)	<0.001*
	BPI Average Pain	5.0 (2.6)	1.6 (2.0)	<0.001*	5.9 (1.8)	1.1 (1.3)	<0.001*	5.4 (2.4)	2.1 (2.4)	<0.001*
	BPI Current Pain	4.5 (3.0)	1.2 (2.0)	<0.001*	5.6 (2.2)	0.5 (0.9)	<0.001*	5.2 (2.7)	1.6 (2.4)	<0.001*
	BPI Pain Severity Score	4.8 (2.6)	1.5 (2.0)	<0.001*	5.8 (1.8)	0.9 (1.1)	<0.001*	5.4 (2.3)	1.9 (2.3)	<0.001*
	Prop. PSS >3.5 (n=77) (%)	51 (67.1)	26 (19.3)	<0.001*	-	-	-	37 (78.7)	40 (24.5)	<0.001*
	Analgesic Effectiveness	45.6 (28.6)	53.0 (37.6)	0.22	46.6 (26.5)	52.7 (41.0)	0.32	49.1 (24.7)	49.2 (37.8)	1.0
	General Activities	5.7 (3.0)	1.5 (2.2)	<0.001*	6.2 (2.2)	1.2 (2.1)	<0.001*	6.3 (2.7)	2.1 (2.7)	<0.001*
	Mood	4.5 (3.3)	1.3 (2.1)	<0.001*	5.0 (3.0)	1.0 (1.9)	<0.001*	6.1 (2.9)	1.4 (2.1)	<0.001*
	Walking	6.0 (3.5)	1.3 (2.2)	<0.001*	6.0 (3.1)	1.3 (2.4)	<0.001*	6.2 (3.4)	2.1 (2.9)	<0.001*
	Normal Work	6.0 (3.2)	1.6 (2.5)	<0.001*	6.4 (2.8)	1.3 (2.3)	<0.001*	6.5 (3.0)	2.3 (3.0)	<0.001*
	Relations	3.8 (3.4)	0.8 (1.8)	<0.001*	4.26 (3.2)	0.5 (1.4)	<0.001*	5.0 (3.1)	1.0 (2.0)	<0.001*
	Sleep	5.0 (3.3)	1.9 (2.6)	<0.001*	5.6 (2.8)	1.6 (2.4)	<0.001*	6.5 (2.6)	2.05 (2.6)	<0.001*
	Enjoyment	5.5 (3.2)	1.5 (2.4)	<0.001*	6.0 (2.7)	1.2 (2.2)	<0.001*	6.4 (3.2)	2.0 (2.6)	<0.001*
	BPI Pain Interference Score	5.2 (2.7)	1.4 (2.0)	<0.001*	5.6 (2.3)	1.2 (1.7)	<0.001*	6.1 (2.6)	1.8 (2.2)	<0.001*
	PDQ: Low Risk (n=) (%)	44 (57.9)	119 (88.8)		40 (51.9)	123 (92.5)		0	163 (100)	
	PDQ: Moderate Risk (n=) (%)	17 (22.4)	11 (8.2)	<0.001*	20 (26.0)	8 (6.0)	<0.001*	28 (59.6)	0	<0.001*
	PDQ: High Risk (n=) (%)	15 (19.7)	4 (3.0)		17 (22.1)	2 (1.5)		19 (40.4)	0	

For peer review only

For peer review only

STROBE Statement—checklist of items that should be included in reports of observational studies

	Item No.	Recommendation	Page No.
**Title and abstract**	1	(*a*) Indicate the study's design with a commonly used term in the title or the abstract	1
		(*b*) Provide in the abstract an informative and balanced summary of what was done and what was found	1
**Introduction**			
Background/rationale	2	Explain the scientific background and rationale for the investigation being reported	2
Objectives	3	State specific objectives, including any prespecified hypotheses	3
**Methods**			
Study design	4	Present key elements of study design early in the paper	3
Setting	5	Describe the setting, locations, and relevant dates, including periods of recruitment, exposure, follow-up, and data collection	3
Participants	6	(*a*) *Cohort study*—Give the eligibility criteria, and the sources and methods of selection of participants. Describe methods of follow-up<br>*Case-control study*—Give the eligibility criteria, and the sources and methods of case ascertainment and control selection. Give the rationale for the choice of cases and controls<br>*Cross-sectional study*—Give the eligibility criteria, and the sources and methods of selection of participants	3 &4
		(*b*) *Cohort study*—For matched studies, give matching criteria and number of exposed and unexposed<br>*Case-control study*—For matched studies, give matching criteria and the number of controls per case	N/A
Variables	7	Clearly define all outcomes, exposures, predictors, potential confounders, and effect modifiers. Give diagnostic criteria, if applicable	5
Data sources/ measurement	8*	For each variable of interest, give sources of data and details of methods of assessment (measurement). Describe comparability of assessment methods if there is more than one group	5
Bias	9	Describe any efforts to address potential sources of bias	4-6
Study size	10	Explain how the study size was arrived at	4

Continued on next page

For peer review only

Quantitative variables	11	Explain how quantitative variables were handled in the analyses. If applicable, describe which groupings were chosen and why	6
Statistical methods	12	(*a*) Describe all statistical methods, including those used to control for confounding	6
		(*b*) Describe any methods used to examine subgroups and interactions	6
		(*c*) Explain how missing data were addressed	6
		(*d*) *Cohort study*—If applicable, explain how loss to follow-up was addressed<br>*Case-control study*—If applicable, explain how matching of cases and controls was addressed<br>*Cross-sectional study*—If applicable, describe analytical methods taking account of sampling strategy	6
		(*e*) Describe any sensitivity analyses	-
**Results**			
Participants	13*	(a) Report numbers of individuals at each stage of study—eg numbers potentially eligible, examined for eligibility, confirmed eligible, included in the study, completing follow-up, and analysed	7
		(b) Give reasons for non-participation at each stage	7
		(c) Consider use of a flow diagram	-
Descriptive data	14*	(a) Give characteristics of study participants (eg demographic, clinical, social) and information on exposures and potential confounders	7
		(b) Indicate number of participants with missing data for each variable of interest	7
		(c) *Cohort study*—Summarise follow-up time (eg, average and total amount)	7
Outcome data	15*	*Cohort study*—Report numbers of outcome events or summary measures over time	9
		Case-control study—Report numbers in each exposure category, or summary measures of exposure	-
		Cross-sectional study—Report numbers of outcome events or summary measures	-
Main results	16	(*a*) Give unadjusted estimates and, if applicable, confounder-adjusted estimates and their precision (eg, 95% confidence interval). Make clear which confounders were adjusted for and why they were included	8-10
		(*b*) Report category boundaries when continuous variables were categorized	8-10
		(*c*) If relevant, consider translating estimates of relative risk into absolute risk for a meaningful time period	N/A

Continued on next page

For peer review only - http://bmjopen.bmj.com/site/about/guidelines.xhtml

Other analyses	17	Report other analyses done—eg analyses of subgroups and interactions, and sensitivity analyses	8-10
**Discussion**			
Key results	18	Summarise key results with reference to study objectives	-
Limitations	19	Discuss limitations of the study, taking into account sources of potential bias or imprecision. Discuss both direction and magnitude of any potential bias	10-12
Interpretation	20	Give a cautious overall interpretation of results considering objectives, limitations, multiplicity of analyses, results from similar studies, and other relevant evidence	10-12
Generalisability	21	Discuss the generalisability (external validity) of the study results	10-12
**Other information**			
Funding	22	Give the source of funding and the role of the funders for the present study and, if applicable, for the original study on which the present article is based	Declarations section

*Give information separately for cases and controls in case-control studies and, if applicable, for exposed and unexposed groups in cohort and cross-sectional studies.

Note: An Explanation and Elaboration article discusses each checklist item and gives methodological background and published examples of transparent reporting. The STROBE checklist is best used in conjunction with this article (freely available on the Web sites of PLoS Medicine at http://www.plosmedicine.org/, Annals of Internal Medicine at http://www.annals.org/, and Epidemiology at http://www.epidem.com/). Information on the STROBE Initiative is available at www.strobe-statement.org.

For peer review only

BMJ Open

A prospective observational study to examine health related quality of life and develop models to predict long-term patient reported outcomes six-months after hospital discharge with blunt thoracic injuries.

Journal:	*BMJ Open*
Manuscript ID	bmjopen-2021-049292.R2
Article Type:	Original research
Date Submitted by the Author:	07-Jun-2021

Complete List of Authors:	Baker, Edward; King's College London, Florence Nightingale Faculty of Nursing, Midwifery and Palliative Care; King's College Hospital, Emergency Department<br>Battle, Ceri; Swansea Bay University Health Board, Welsh Institute of Biomedical and Emergency Medicine Research<br>Banjeri, Abhishek; Buckingham Healthcare NHS Trust<br>Carlton, Edward; North Bristol NHS Trust, Emergency Department<br>Dixon, Christine; Surrey and Sussex Healthcare NHS Trust, Emergency Department<br>Ferry, Jennifer; Aneurin Bevan Health Board, Department of Anesthetics<br>Hopkins, Philip; King's College Hospital NHS Foundation Trust, Critical Care<br>Jones, Robert; Barnsley Hospital NHS Foundation Trust<br>Murrells, Trevor; King's College London, Florence Nightingale Faculty of Nursing, Midwifery and Palliative Care<br>Norton, Christine; King's College London, Florence Nightingale Faculty of Nursing & Midwifery<br>Patient, Lee; St George's Healthcare NHS Trust, Emergency Department<br>Rasheed, Ashraf; Aneurin Bevan Health Board, General Surgery<br>Skene, Imogen; UCL, Primary care and population health; Barts Health NHS Trust, Emergency Department<br>Tabner, Andrew; University Hospitals of Derby and Burton NHS Foundation Trust<br>Tunnicliff, Malcolm; King's College Hospital NHS Foundation Trust, Emergency Department<br>Young, Louise; Imperial College Healthcare NHS Trust, Emergency Department<br>Xyrichis, Andreas; King's College London, Florence Nightingale Faculty of Nursing, Midwifery and Palliative Care<br>Lee, Gerry; King's College London, Florence Nightingale Faculty of Nursing, Midwifery & Palliative Care

**Primary Subject Heading**:	Emergency medicine
Secondary Subject Heading:	Emergency medicine
Keywords:	ACCIDENT & EMERGENCY MEDICINE, Risk management < HEALTH

	SERVICES ADMINISTRATION & MANAGEMENT, Trauma management < ORTHOPAEDIC & TRAUMA SURGERY, PAIN MANAGEMENT, Cardiothoracic surgery < SURGERY

SCHOLARONE™
Manuscripts

I, the Submitting Author has the right to grant and does grant on behalf of all authors of the Work (as defined in the below author licence), an exclusive licence and/or a non-exclusive licence for contributions from authors who are: i) UK Crown employees; ii) where BMJ has agreed a CC-BY licence shall apply, and/or iii) in accordance with the terms applicable for US Federal Government officers or employees acting as part of their official duties; on a worldwide, perpetual, irrevocable, royalty-free basis to BMJ Publishing Group Ltd ("BMJ") its licensees and where the relevant Journal is co-owned by BMJ to the co-owners of the Journal, to publish the Work in this journal and any other BMJ products and to exploit all rights, as set out in our licence.

The Submitting Author accepts and understands that any supply made under these terms is made by BMJ to the Submitting Author unless you are acting as an employee on behalf of your employer or a postgraduate student of an affiliated institution which is paying any applicable article publishing charge ("APC") for Open Access articles. Where the Submitting Author wishes to make the Work available on an Open Access basis (and intends to pay the relevant APC), the terms of reuse of such Open Access shall be governed by a Creative Commons licence – details of these licences and which Creative Commons licence will apply to this Work are set out in our licence referred to above.

Other than as permitted in any relevant BMJ Author's Self Archiving Policies, I confirm this Work has not been accepted for publication elsewhere, is not being considered for publication elsewhere and does not duplicate material already published. I confirm all authors consent to publication of this Work and authorise the granting of this licence.

For peer review only

A prospective observational study to examine health related quality of life and develop models to predict long-term patient reported outcomes six-months after hospital discharge with blunt thoracic injuries.

Mr. Edward BAKER (Corresponding Author)
NIHR Clinical Doctoral Research Fellow - Kings College London
edward.e.baker@kcl.ac.uk
Florence Nightingale Faculty of Nursing, Midwifery and Palliative Care
King's College London
James Clerk Maxwell Building
57 Waterloo Road
London
SE1 8WA

Dr Ceri Battle
Consultant Physiotherapist - Swansea Bay University Health Board
ceri.battle@wales.nhs.uk

Dr Abhishek Banjeri
Emergency Medicine Consultant - Buckingham Healthcare NHS Trust
abhishekbanerji@nhs.net

Dr Edward Carlton
Emergency Medicine Consultant – North Bristol NHS Trust
eddcarlton@gmail.com

Ms. Christine Dixon
Trauma Nursing Lead – Surrey and Sussex Healthcare NHS Trust
christine.dixon14@nhs.net

Dr Jennifer Ferry
Consultant Anaesthetist – Aneurin Bevan University Health Board
jennifer.ferry@wales.nhs.uk

Dr Philip Hopkins
Intensive Care Consultant and ICM Research Lead – Kings College Hospital NHS Foundation Trust
p.hopkins@nhs.net

Dr Robert Jones
Emergency Medicine Consultant – Barnsley Hospital NHS Foundation Trust
robertjones5@nhs.net

Mr. Trevor Murrells
Faculty Statistician – King's College London
trevor.murrells@kcl.ac.uk

Professor Christine Norton
Professor of Nursing Research – King's College London

For peer review only

christine.norton@kcl.ac.uk

Mr. Lee Patient
Nurse Consultant Emergency Medicine – St Georges University Hospital NHS Foundation Trust
lee.patient@stgeorges.nhs.uk

Professor Ashraf Rasheed
Professor of General Surgery – Aneurin Bevan University Health Board
ashraf.rasheed@wales.nhs.uk

Ms. Imogen Skene
Lead Research Nurse Emergency Medicine – Barts Health NHS Trust
i.skene@nhs.net

Dr Andrew Tabner
Emergency Medicine Consultant – University Hospitals of Derby and Burton NHS Foundation Trust
andrew.tabner@nhs.net

Dr Malcolm Tunnicliff
Emergency Medicine Consultant – King's College Hospital NHS Foundation Trusts
malcolm.tunnicliff@nhs.net

Ms. Louise Young
Lead Research Nurse Emergency Medicine – Imperial College Healthcare NHS Trust
louise.young10@nhs.net

Dr Andreas Xyrichis
Senior Lecturer in Nursing – King's College London
andreas.xyrichis@kcl.ac.uk

Dr Gerry Lee
Reader in Advanced Clinical Practice – King's College London
gerry.lee@kcl.ac.uk
Florence Nightingale Faculty of Nursing, Midwifery and Palliative Care
King's College London
James Clerk Maxwell Building
57 Waterloo Road
London
SE1 8WA

For peer review only

For peer review only - http://bmjopen.bmj.com/site/about/guidelines.xhtml

For peer review only - http://bmjopen.bmj.com/site/about/guidelines.xhtml

Abstract:

Objective: This study aimed to examine the long-term outcomes and health-related quality of life in patients with blunt thoracic injuries over six-months from hospital discharge and develop models to predict long-term patient reported outcomes.

Design: A prospective observational study using longitudinal survey design.

Setting: The study recruitment was undertaken at 12 UK hospitals which represented diverse geographical locations and covered urban, sub-urban and rural areas across England and Wales.

Participants: 377 patients admitted to hospital with blunt thoracic injuries were recruited between June 2018-October 2020.

Methods: Participants completed a bank of 2 quality of life surveys (Short Form-12 and EuroQol-5d-5l) and 2 pain questionnaires (Brief Pain Inventory and PainDetect questionnaire) at 4 time-points over the first 6-months after discharge from hospital. A total of 211 (63%) participants completed the outcomes data at 6-months after hospital discharge.

Outcomes Measures: Three outcomes were measured using pre-existing and validated patient reported outcome measures. Outcomes included: Poor Physical function (SF-12 Physical Component Score); Chronic pain (Brief Pain Inventory Pain Severity Score); and Neuropathic pain (PainDetect questionnaire).

Results: Despite a trend towards improving physical functional and pain at 6-months, outcomes did not return to participants perceived baseline level of function. At six-months after hospital discharge, 37% (n=77) of participants reported poor physical function; 36.5% (n=77) reported a chronic pain state; and 22% (n=47) reported pain with a neuropathic component. Predictive models were developed for each outcome highlighting important data collection requirements for predicting long-term outcomes in this population. Model diagnostics including calibration and discrimination statistics suggested good model fit in this development cohort.

Conclusions: This study identified the recovery trajectories for patients with blunt thoracic injuries over the first six-months after hospital discharge and present prognostic models for three important outcomes which after external validation could be used as clinical risk stratification scores.

Keywords: Trauma; Chest Injury; Rib Fractures; Predictive modelling; Long-term outcomes; Pain; Neuropathic Pain; Physical Functioning; Quality of Life; Patient Reported Outcome Measures.

For peer review only - http://bmjopen.bmj.com/site/about/guidelines.xhtml

For peer review only

For peer review only - http://bmjopen.bmj.com/site/about/guidelines.xhtml

Strengths and Limitations of the Study:

- This study highlights the physical and pain sequelae associated with recovery after blunt thoracic injuries and provides insights into the recovery trajectory for this population.
- The study highlights the complexity of developing prognostic models for long term outcomes after blunt thoracic injuries and acknowledges the limited current clinical utility in predictive models presented in this study.
- The longitudinal study design using patient reported outcome measures demonstrates the importance of eliciting health data from this population over throughout the post discharge recovery period.
- Missingness within follow-up dataset is a limitation to the prognostic modelling process.
- Despite the benefits of patient reported outcome measures, it is important to acknowledge the potential inherent bias within these measures.

For peer review only

For peer review only - http://bmjopen.bmj.com/site/about/guidelines.xhtml

A prospective observational study to examine health related quality of life and develop models to predict long-term patient reported outcomes six-months after hospital discharge with blunt thoracic injuries.

Background:

Blunt thoracic injury (BTI) is a term that covers a spectrum of different injury patterns and severities across all adult age groups.[1] It accounts for about 15% of injured patients presenting to trauma hospitals, with mortality rates reported between 4-20%.[2] Outcomes within trauma care have historically been measured primarily using mortality as a key outcome.[3] During the last two decades, there has been a decrease in the mortality rates of polytrauma patients in high income countries with organised trauma systems.[4] As the mortality rates have decreased, there has been a growing focus on measuring outcomes relating to injury morbidity.[3 5] There is a growing body of international research investigating the long-term outcomes of patients with BTI, but within the United Kingdom (UK), there is a paucity of BTI outcomes research with little consistency in the methods employed.[6 7]

For patients recovering from BTI, it has been identified that reduced physical function, persistent altered respiratory function, and chronic pain frequently impact on all aspects of daily living.[6] Health Related Quality of Life (HRQoL) provides a strong foundation for measuring the impact of these symptoms with a holistic and comprehensive approach.[8] It is important not to isolate individual components of daily living but consider them as a single entity, and HRQoL measurement provides a vehicle to do this.[9] Furthermore, it allows the development of theory driven interventions that impact on all elements of daily living.[10] For the purposes of this study HRQOL is defined as including all aspects of self-perceived well-being that are related to, or affected by, the presence of disease or treatment.[11]

Follow-up after hospital discharge with major trauma is often limited within the UK's National Health Service (NHS), resulting in limited clinical knowledge about the recovery journey of BTI patients.[12] This also leaves clinicians without a trajectory for recovery making it very difficult to adequately educate and prepare patients for self-management in the post hospital discharge recovery period.[13] Evidence from this study will be used to facilitate the identification of patients at risk of poor outcomes after BTI, help develop health promotion materials, and to support the development of follow-up interventions within BTI management.[13]

Having the ability to predict outcomes and develop methods for risk stratification is extremely important in trauma care.[14] There is a growing body of prognostic research in this area with simple

For peer review only - http://bmjopen.bmj.com/site/about/guidelines.xhtml

scoring systems developed and validated with effective predictive abilities across different health systems.[2] Where resources for patient follow-up are limited, it is key that effective methods for identifying patients in high-risk groups for complications or poor outcomes are in place, allowing resources to be focused on these patients at the greatest risk. The aim of this study was to examine the long-term outcomes and HRQoL in patients with BTI over six-months from hospital discharge and develop models to predict long-term patient reported outcomes. Study objectives were as follows:

- Investigate changes in HRQoL over 6 months after hospital discharge with BTI.
- Examine the evolution of pain and neuropathic pain over six months after discharge from hospital with BTI.
- Develop predictive models for poor physical functioning, chronic pain, and neuropathic pain at six-months after hospital discharge with BTI.

Methods:

This prospective multicentred observational study employed longitudinal survey methods to follow-up patients recovering from BTI over a six-month period from hospital discharge. This research forms the quantitative component of a mixed methods study investigating recovery after BTI. The qualitative components will be reported separately.

Study Setting:

The study population sample was recruited from Major Trauma Centres (MTC) (Level 1) (n=5) and Trauma Units (Level 2-3) (n=7) from diverse geographical locations within urban, sub-urban and rural areas across England and Wales. Sites were set up in a stepwise fashion and in total 12 acute hospital sites recruited participants between 6 to 10 months in two separate recruitment cohorts between June 2018 and March 2020.

Eligibility Criteria:

Inclusion criteria:

- Participant is willing and able to give informed consent for participation in the study
- Male or female, aged 16 years or above
- Admitted to a Major Trauma Centre/Trauma Unit with primary BTI
- Admitted to hospital for a period of 24 hours or greater

For peer review only - http://bmjopen.bmj.com/site/about/guidelines.xhtml

Exclusion criteria:

- Unstable spinal fracture or spinal cord injury (SCI)
- Traumatic brain injury (TBI) with cognitive impairment
- Altered mental status that would prevent accurate self-assessment through questionnaires or prevent informed consent into study
- Associated unstable pelvic fracture or open abdomen requiring extended periods of treatment in hospital
- Limb injuries from same traumatic event that require surgical intervention
- Participant is a prisoner / in police custody at time of recruitment

Data Collection:

Data collection was undertaken at four timepoints: (i) Hospital discharge (including a pre-injury estimation of HRQoL); (ii) 1 month after discharge; (iii) 3 months after discharge; and (iv) 6 months after discharge. Potential participants were identified by the local clinical team or the clinical research nurses at each site. Demographic and injury related data were collated throughout the acute admission using a previously tested case report form,[1] and site staff conducted data collection at hospital discharge. Post discharge follow-up was conducted by the central research team at the host university using either postal surveys or electronic surveys hosted by SurveyMonkey. During post discharge follow-up non-responding particiapnts were contacted up to three time using a mixed multi-media approach including telephone, post, or email, to maximise participation and minimise the potential for missing data.

Ethics Considerations:

Formal informed written consent was taken from all participants after information sheets had been provided and sufficient time was allowed for discussion with family and clinical research staff. Ethical approval was granted by the 'Hampshire A' South Central Research Ethics Committee in June 2018 (ref: 18/SC/0230). Participants were informed that everyone who completed data collection at all timepoints would be placed in a prize draw to win a £50 shopping voucher to recognise the time associated with participation in this longitudinal follow-up study.

Sample Size:

This observational study had an exploratory design and pragmatic approach to sample size. Although there is no consensus on the sample requirements for logistic regression analysis, based on previous

For peer review only - http://bmjopen.bmj.com/site/about/guidelines.xhtml

studies we aimed to have 10 events for each predictor variables included in the model.[2 3] As it was likely that 10 predictor variables would be used in the modelling process for each model, the aim was to achieve 100 events (i.e., outcomes of interest) in the sample.

Outcome Measures:

HRQoL was explored firstly at a global level through the Physical Component Score (PCS) and Mental Component Score (MCS) of the Short Form-12 (SF-12),[15] and secondly, using the individual levels of the EuroQol 5-dimensions, 5-Levels (EQ5D-5L).[16 17] Pain was previously recognised as an ongoing problem throughout post hospital discharge recovery.[6] In this study, pain severity and impact were measured using the Brief Pain Inventory[18] and neuropathic pain was assessed using the PainDetect Score[19] Using existing literature on HRQoL after BTI,[6 20] 3 key outcomes were selected:

1. **Physical Function** is an individual's ability to undertake actions that involve physical activities, ranging from activities of daily living (ADLs) to more complex activities that involve a combination of skills, often within a social context.[3] For the purposes of this study, physical functioning was measured using the PCS of the SF-12 at six-months after discharge from hospital. A PCS score of ≤35 (Range: 0-100 with higher scores equalling better Physical function) was taken as defining poor physical functioning from previous literature.[15]
2. **Chronic Pain** is defined as pain that persists beyond the normal expected healing time and therefore lacks the acute warning function of physiological nociception. Pain is normally considered as chronic when it lasts or recurs for more than three to 6-months.[21] For this study, chronic pain was defined as having a Brief Pain Inventory (BPI) Pain Severity Score (PSS) of ≥3.5 (Range: 0-10 and higher scores equals increased pain severity) at 6-months after hospital discharge.[22]
3. **Neuropathic pain** is defined as pain caused by a lesion or disease of the somatosensory nervous system.[23] For the purposes of this study, participants with a potential neuropathic pain component were identified using the PainDetect Questionnaire (PDQ).[19] A PainDetect score ≥12 (Range: -1 – 38) at 6-months after hospital discharge defined our cohort of patients with a potential neuropathic pain component.[24]

Although the limitations of using binary outcome measures were acknowledged in the planning phase, this was an important component in the process of developing prognostic models which could be used as risk stratification scores in the clinical practice setting.

For peer review only

For peer review only - http://bmjopen.bmj.com/site/about/guidelines.xhtml

Selection of candidate predictor variables:

A collection of predictor variables was required which covered multiple potentially influential factors relating to the outcomes of interest. Initially, during the study development, a long list of candidate variables were selected from previous literature.[6 10 25 26] From this long-list, shortlists of variables for each outcome were compiled. Decision-making for entering variables into the modelling process was based on their clinical significance and achieving statistical significance at the 5% level in the univariate analysis phase. There was an 'a priori' plan for candidate variables to be selected in 3 phases, initially using only variables that are readily available in clinical practice, secondly, variables that are currently not readily available in clinical practice, and finally using variables collected at post discharge timepoints.

Statistical analysis:

Data analysis was undertaken using SPSS v.26. Statistical data were presented using descriptive and inferential statistical methods and reported according to the Strengthening the Reporting of Observational Studies in Epidemiology (STROBE) statement.[27] Binary and categorical data were presented as percentages and number of cases. Continuous data were presented as means with standard deviations. The statistical tests applied for binary/categorical data were Chi-square test of association, except where the expected cell counts were ≤5 when the Fisher's exact test was used. Comparisons continuous data from two independent groups was undertaken using the Student t-test. Longitudinal changes in continuous variables over the course of the post discharge follow-up period are reported at a group level. This uses the longitudinal change in the group mean from one timepoint to the next to measure change.

Missing data was addressed using the multiple imputation procedure in SPSS. Most missing data were found within the follow-up phase of data collection where units of data (i.e., surveys) were not returned. The imputation model included all predictor variables with potential for inclusion in the modelling process including candidate predictor variables from within the hospital admission and from the post discharge follow-up. A total of 10 imputed datasets were created. Longitudinal imputation of outcome variables was considered but not conducted in this study due to the high level of missingness at both 1-month and 3 months and the low number of participants who completed the follow-up data collection at all timepoints. Ultimately despite the potential limitations, a case wise approach was used.

The predictive model framework was developed in accordance with the TRIPOD (Transparent Reporting of multivariable predication model for Individual Prognosis Or Diagnosis) methodology[28]

For peer review only - http://bmjopen.bmj.com/site/about/guidelines.xhtml

and the study methods based on previously published prognostic modelling studies.[2,3,29] A multivariable logistic regression analysis was used to evaluate the association of the predictors with the outcome (dependent variable) in each imputed data set using the forward step wise method.[30] In this process, SPSS automatically produced a final model that aggregated the results across all the imputed datasets.

Calibration was evaluated using a Hosmer-Lemeshow test where a p-value >0.05 was considered to represent good calibration. In addition, graphical calibration plots were developed. A receiver operating characteristic (ROC) curve was plotted for each model. A c-statistic (area under the receiver operator curve) was calculated, that tested whether the amount of discrimination was significantly different from random (c-statistic of 0.5 indicates that discrimination is no better than the toss of a coin). Performance of the final model was assessed using sensitivity and specificity.[31] Throughout statistical significance was recognised at the 5% level (p<0.05).

Patient and Public involvement:

A patient and public reference group was formed and involved in the development phases of this study including during protocol development and application for ethical approval but have not been involved in data collection, analysis, or interpretation.

Results:

A total of 337 patients were recruited to the study. The mean age of participants was 62.0 years (±16.5) and 69% (n=231) were male. The predominant mechanism of injury was falls <2m (45%, n=153) and the mean number of rib fractures was 5.2 (±4.0). At six-months after discharge, 37% (n=77) of the sample self-reported poor physical functioning, 37% (n=77) reported chronic pain, and 22% (n=47) reported pain with a likely neuropathic pain component. **Table 1** summarises key demographic data for the total sample. Further greater detailed demographic data is presented in **Table S1** as a supplementary file to this study.

Missing Data Analysis:

A total of 337 patients were recruited to the study. Overall, the combined response rate at all timepoints was 72%. At hospital discharge 95% (n=320) completed the discharge data collection timepoint, subsequently, 62% (n=210) and 43% (n=144) completed data collection at 1-month and 3-month after discharge respectively. At 6-months, 63% (211) of the total sample completed follow-up where the study outcomes data were collected. Of the 211 participants who completed the 6-month

For peer review only

For peer review only - http://bmjopen.bmj.com/site/about/guidelines.xhtml

follow-up, n=200 (94%) completed discharge data collection, 174 (82%) completed the 1-month data collection timepoint, and 142 (67%) completed data collection at 3-months after discharge.

Overall missingness within the dataset was predominately from 'unit missing data' (i.e., completed survey not returned by participant) as individual items of missing data within a returned survey were followed up with participants at the time of data collection. Amongst candidate predictor variables 17% (n=37) were missing at 1-month follow-up, and 33% (n=69) at 3 months. Although this represents high levels of missingness within the dataset, this is not uncommon in longitudinal follow-up studies after traumatic injuries (Baker, Xyrichis, Norton, Hopkins, & Lee, 2018).

A missing data analysis was undertaken as part of the analysis process to identify any differences in the characteristics of participants who did or did not respond to follow-up at six months after hospital discharge when outcome data were collected. **Supplementary table S2** presents the results from this analysis. Although there was a statistically significant difference in participants age, incidence of cardiac disease and cancer, and MCS at the pre-injury, discharge and one-month post discharge timepoint, these were not deemed to be clinically significant differences. Furthermore, a logistic regression model was built using a stepwise forward approach using variables identified in the univariable analysis of missingness to identify predictors of responder status at six months after discharge. It was not possible to fit a model using the selected variables and no statistically significant predictors were identified. Since none were identified, it was assumed that the two groups were characteristically similar and therefore a sensitivity analysis, using weights calculated from the logistic regression model, was not undertaken. Based on Little and Rubin's taxonomy missingness was assumed to be Missing at Random (MAR) at the unit level and multiple imputation methods in SPSS were used to address item level missingness (Ayilara et al., 2019; Schlomer, Bauman, & Card, 2010).

For peer review only - http://bmjopen.bmj.com/site/about/guidelines.xhtml

Table 1: Demographics of entire cohort and a comparison of characteristics for each outcome of interest at six-months after discharge

Result: n= (%) unless otherwise indicated	Total sample (n=337)	**Poor Physical Function** (n=77)	**Good Physical Function** (n=134)	p-value	**Chronic Pain** (n=77)	**No Chronic Pain** (n=134)	p-value	**Neuropathic component likely** (n=47)	**Neuropathic component unlikely** (n=164)	p-value
Age (Mean (SD))	62.0 (16.5)	69.1 (13.9)	60.8 (16.3)	<0.001	66.7 (16.4)	62.1 (15.6)	0.05	63.4 (17.0)	63.9 (15.7)	0.84
Male (%)	231 (68.5)	49 (63.6)	97 (72.4)	0.19	54 (70.1)	91 (67.9)	0.80	33 (70.2)	112 (68.7)	0.85
Injury Severity Score (Mean (SD))	11.2 (7.3)	10.9 (7.8)	11.4 (6.8)	0.61	11.3 (7.9)	11.2 (6.8)	0.89	11.6 (8.3)	11.2 (6.9)	0.76
**Mechanism of Injury:**										
RTC	78 (23.1)	14 (18.2)	38 (28.4)		19 (24.7)	33 (24.6)		9 (19.1)	43 (26.4)	
Crush	11 (3.3)	3 (3.9)	4 (3.0)		1 (1.3)	6 (4.5)		1 (2.1)	6 (3.7)	
Ped vs. Vehicle	4 (1.2)	1 (1.3)	2 (1.5)		1 (1.3)	2 (1.5)		1 (2.1)	2 (1.2)	
Fall>2m	69 (20.5)	13 (16.9)	30 (22.4)	0.23	18 (23.4)	25 (18.7)	0.78	13 (27.7)	30 (18.4)	0.82
Fall<2m	153 (45.4)	44 (57.1)	54 (40.3)		37 (48.1)	61 (45.5)		22 (46.8)	76 (46.6)	
Assault	14 (4.2)	1 (1.3)	4 (3.0)		1 (1.3)	3 (2.2)		1 (2.1)	3 (1.8)	
CPR	4 (1.2)	1 (1.3)	0		0	1 (0.7)		0	1 (0.6)	
Rapid deceleration	4 (1.2)	0	2 (1.5)		0	2 (1.5)		0	2 (1.2)	
**Co-morbidities:**										
Respiratory co-morbidity	70 (20.8)	21 (27.3)	21 (15.7)	0.042	21 (27.3)	20 (14.9)	0.031	12 (25.5)	29 (17.8)	0.24
Cardiac co-morbidity	147 (43.6)	49 (63.6)	53 (39.6)	0.001	47 (61.0)	54 (40.3)	0.004	27 (57.4)	74 (45.4)	0.15
Neuro co-morbidity	50 (14.8)	20 (26.0)	9 (6.7)	<0.001	19 (24.7)	10 (7.5)	0.001	10 (21.3)	19 (11.7)	0.092
MSK co-morbidity	89 (26.4)	26 (33.8)	35 (26.1)	0.24	30 (39.0)	31 (23.1)	0.016	16 (34.0)	45 (27.6)	0.39
Pre-Injury regular analgesic use	61 (18.1)	23 (29.9)	15 (11.2)	0.001	30 (39.0)	8 (6.0)	<0.001	19 (40.4)	19 (11.7)	<0.001
**Injury Descriptors:**										
No. Rib Fractures (Mean (SD))	5.2 (4.0)	5.3 (3.8)	5.3 (4.0)	0.90	5.4 (3.9)	5.3 (3.9)	0.74	5.6 (4.1)	5.2 (3.8)	0.55
Operative Rib Fixation	16 (4.7)	6 (7.8)	3 (2.2)	0.05	2 (2.6)	7 (5.2)	0.36	3 (6.4)	6 (3.7)	0.42
First Rib fracture	31 (9.2)	6 (7.8)	13 (9.7)	0.64	10 (13.0)	9 (6.7)	0.13	7 (14.9)	12 (7.4)	0.11
Bilateral rib fractures	44 (13.1)	9 (11.7)	19 (14.2)	0.61	11 (14.3)	17 (12.7)	0.76	10 (21.3)	18 (11.0)	0.07
Radiological Flail	60 (17.8)	13 (16.9)	29 (21.6)	0.41	16 (20.8)	26 (19.4)	0.83	7 (14.9)	35 (21.5)	0.32
Extra-thoracic injuries	104 (30.9)	25 (32.5)	35 (26.1)	0.33	19 (24.7)	40 (29.9)	0.40	12 (25.5)	47 (28.8)	0.66
**Analgesic mode:**										

For peer review only

Intermittent analgesia only	179 (53.1)	37 (48.1)	77 (57.5)	0.19	37 (48.1)	76 (56.7)	0.20	22 (46.8)	91 (55.8)	0.28
Regional Analgesia	106 (31.5)	29 (37.7)	41 (30.6)	0.29	31 (40.3)	39 (29.1)	0.11	20 (42.6)	50 (30.7)	0.13
Patient Controlled Analgesic only	93 (27.6)	23 (29.9)	29 (21.6)	0.18	20 (26.0)	32 (23.9)	0.76	12 (25.5)	40 (24.5)	0.89
**Short term inpatient outcomes:**										
Complications:	111 (32.9)	34 (44.2)	33 (24.6)	0.003	25 (32.5)	42 (31.3)	0.89	18 (38.3)	49 (30.1)	0.29
Post Admission Ventilation:	26 (7.7)	9 (11.7)	5 (3.7)	0.025	6 (7.8)	8 (6.0)	0.62	5 (10.6)	9 (5.5)	0.22
ICU Length of Stay (Mean (SD))	7.6 (7.4)	9.42 (7.5)	6.42 (5.8)	0.16	7.6 (5.4)	7.8 (7.3)	0.93	9.4 (7.2)	7.4 (6.6)	0.49
Hospital Length of Stay (Mean (SD))	8.9 (9.5)	11.7 (10.2)	6.9 (5.7)	<0.001	10.4 (9.3)	7.7 (7.0)	0.03	9.9 (7.2)	8.3 (8.2)	0.21

Abbrev: CPR = Cardio-pulmonary Resuscitation; ICU = Intensive Care Unit; IQR = Interquartile Range; Ped = Pedestrian; RTC = Road Traffic Collision

For peer review only

For peer review only - http://bmjopen.bmj.com/site/about/guidelines.xhtml

Changes in HRQoL over six-months after discharge from hospital

Physical function was clearly affected by the injuries experienced by this cohort. Measures of HRQoL are presented in **Table 2**. As expected, the mean PCS score (SF-12) saw a 19.3% (46.5-27.2) drop from the pre-injury estimate through to the initial hospital discharge timepoint representing a substantial negative change in physical functioning. Over the 6-month follow-up there were incremental improvements in the mean PCS score (31.6, 40.1, and 41.7 at 1, 3 and 6-month respectively) but the sample failed to return to their baseline estimate of physical function (-4.8%) or population norms (-8.3%) within the 6-month follow-up period. During the same timeframe, the impact of injury on mental health was lesser than on physical function. The mean MCS score (SF-12) dropped from 49.7 to 44.4 (-5.3) from the pre-injury estimate through to hospital discharge, dropping a further 0.5% to 43.9 at 1-month after hospital discharge. Between the 1 and 6 months the mean MCS increased to 47.8 which remained 1.9% below the pre-injury estimate and 2.2% below the population norms for England.

For peer review only

For peer review only - http://bmjopen.bmj.com/site/about/guidelines.xhtml

Table 2: Cohort outcomes with comparisons between outcomes of interest at six-months after discharge

	Outcome	Total sample (n=337)	Poor physical function (n=77)	Good physical function (n=134)	p-value	Chronic Pain (n=77)	No chronic pain (n=134)	p-value	Neuropathic pain likely (n=47)	Neuropathic pain unlikely (n=164)	p-value
Pre-Injury	SF-12 PCS +	46.5 (11.0)	40.0 (11.0)	50.8 (7.7)	<0.001	41.9 (11.4)	49.6 (8.6)	<0.001	41.7 (11.5)	48.3 (9.6)	<0.001
	SF-12 MCS +	49.7 (10.7)	48.2 (11.7)	53.1 (7.6)	<0.001	49.1 (10.9)	52.7 (8.4)	0.009	48.2 (10.6)	52.3 (9.1)	0.009
Discharge (n=320)	SF-12 PCS +	27.2 (8.6)	24.3 (7.4)	28.4 (8.6)	0.001	25.1 (8.3)	27.9 (8.3)	0.02	25.1 (8.4)	27.5 (8.3)	0.09
	SF-12 MCS +	44.4 (11.8)	43.5 (11.8)	47.0 (11.5)	0.04	46.1 (11.5)	45.5 (11.9)	0.76	42.6 (11.7)	46.7 (11.6)	0.04
	BPI Pain Severity Score +	5.0 (2.1)	5.3 (2.1)	4.8 (2.0)	0.10	5.3 (2.0)	4.7 (2.0)	0.06	5.6 (2.0)	4.7 (2.0)	0.009
	BPI Pain Interference Score +	5.7 (2.3)	6.5 (2.0)	5.2 (2.4)	<0.001	5.9 (2.0)	5.5 (2.4)	0.18	6.4 (2.0)	5.4 (2.3)	0.004
	PDQ: Low Risk ++	222 (69.4)	48 (64.9)	95 (73.1)		47 (63.5)	96 (74.4)		24 (52.2)	119 (75.8)	
	PDQ: Moderate Risk ++	67 (20.9)	18 (24.3)	26 (20.0)	0.42	22 (29.7)	21 (16.3)	0.075	17 (37.0)	26 (16.6)	0.006
	PDQ: High Risk ++	31 (9.7)	8 (10.8)	9 (6.9)		5 (6.8)	12 (9.3)		5 (10.9)	12 (7.6)	
1-month (n=210)	SF-12 PCS +	31.6 (7.8)	27.0 (6.0)	33.5 (7.5)	<0.001	27.7 (6.0)	32.9 (7.8)	<0.001	27.8 (5.8)	32.1 (7.9)	<0.001
	SF-12 MCS +	43.9 (11.8)	40.8 (12.2)	46.8 (11.2)	0.001	4.07 (12.7)	46.7 (10.9)	0.001	35.6 (12.3)	47.0 (10.6)	<0.001
	BPI Pain Severity Score +	3.8 (2.2)	4.7 (2.3)	3.2 (1.9)	<0.001	5.2 (2.0)	3.0 (1.9)	<0.001	5.5 (2.2)	3.3 (1.9)	<0.001
	BPI Pain Interference Score +	4.4 (2.7)	5.6 (2.6)	3.7 (2.5)	<0.001	5.8 (2.4)	3.6 (2.5)	<0.001	5.6 (2.4)	3.8 (2.4)	<0.001
	PDQ: Low Risk ++	142 (67.6)	39 (61.9)	83 (73.5)		34 (57.6)	88 (75.2)		14 (38.9)	108 (77.1)	
	PDQ: Moderate Risk ++	45 (21.4)	14 (22.2)	22 (19.5)	0.14	13 (22.0)	23 (19.7)	0.005	9 (25.0)	27 (19.3)	<0.001
	PDQ: High Risk ++	23 (11.0)	10 (15.9)	8 (7.1)		12 (20.3)	6 (5.1)		13 (36.1)	5 (3.6)	
3-months (n=144)	SF-12 PCS +	40.1 (11.0)	31.4 (8.8)	44.4 (9.5)	<0.001	32.4 (9.1)	43.9 (10.0)	<0.001	32.9 (8.8)	42.1 (10.8)	<0.001
	SF-12 MCS +	48.3 (11.4)	43.1 (12.9)	50.9 (9.7)	<0.001	43.1 (11.8)	51.0 (10.4)	<0.001	40.0 (12.0)	50.7 (10.2)	<0.001
	BPI Pain Severity Score +	2.9 (2.6)	4.4 (2.7)	2.2 (2.2)	<0.001	5.1 (2.2)	1.8 (2.0)	<0.001	4.8 (2.2)	2.4 (2.4)	<0.001
	BPI Pain Interference Score +	2.6 (2.7)	4.4 (3.1)	1.8 (2.1)	<0.001	5.0 (2.6)	1.5 (2.0)	<0.001	5.0 (2.6)	2.0 (2.4)	<0.001
	PDQ: Low Risk ++	110 (76.4)	28 (60.9)	79 (83.2)		23 (48.9)	84 (89.4)	<0.001	9 (29.0)	98 (89.1)	
	PDQ: Moderate Risk ++	15 (10.4)	7 (15.2)	8 (8.4)	0.012	9 (19.1)	6 (6.4)		9 (29.0)	6 (5.5)	<0.001
	PDQ: High Risk ++	19 (13.2)	11 (23.9)	8 (8.4)		15 (31.9)	4 (4.3)		13 (41.9)	6 (5.5)	
6-months (n=211)	SF-12 PCS +	41.7 (11.5)	29.2 (5.1)	48.9 (7.2)	<0.001	32.9 (8.0)	46.8 (10.1)	<0.001	32.4 (8.5)	44.4 (10.9)	<0.001
	SF-12 MCS +	47.8 (11.8)	42.3 (13.2)	51.0 (9.7)	<0.001	39.4 (11.6)	52.7 (8.9)	<0.001	36.4 (11.8)	51.1 (9.6)	<0.001
	BPI Pain Severity Score +	2.7 (2.7)	4.8 (2.6)	1.5 (2.0)	<0.001	5.8 (1.8)	0.9 (1.1)	<0.001	5.4 (2.3)	1.9 (2.3)	<0.001
	BPI Pain Interference Score +	2.8 (2.9)	5.2 (2.7)	1.4 (2.0)	<0.001	5.6 (2.3)	1.2 (1.7)	<0.001	6.1 (2.6)	1.8 (2.2)	<0.001
	PDQ: Low Risk ++	164 (77.7)	44 (57.9)	119 (88.8)		40 (51.9)	123 (92.5)		0	163 (100)	
	PDQ: Moderate Risk ++	28 (13.3)	17 (22.4)	11 (8.2)	<0.001	20 (26.0)	8 (6.0)	<0.001	28 (59.6)	0	<0.001
	PDQ: High Risk ++	19 (9.0)	15 (19.7)	4 (3.0)		17 (22.1)	2 (1.5)		19 (40.4)	0	

Abbrev: BPI = Brief Pain Inventory; IQR = Interquartile Range; MCS = Mental Component Score; PCS = Physical Component Score; PDQ = PainDetect Questionnaire; SD = Standard Deviation; SF-12 = Short Form -12 Health Survey.

<u>Key:</u> + = Mean (±SD); ++ = n (%)

For peer review only

For peer review only - http://bmjopen.bmj.com/site/about/guidelines.xhtml

The impact of BTI on individual components of HRQoL were measured using the EuroQol-5d-5L questionnaire. **Table 3** presents the individual dimension/level data for each timepoint. At hospital discharge, the lowest scored (best) dimension was Anxiety and Depression with 43% of the sample reporting no problems in this dimension. At 6-months after discharge: ‘Mobility’, ‘Self-Care’, ‘Usual Activities’ and ‘Anxiety/Depression’ were the best rated dimensions, with between 42% and 73% of the sample reporting no problems in these areas. The highest scoring (worst) dimensions at hospital discharge were ‘Usual Activities’ where 42% (n=135) reported being wholly unable to complete usual activities. Between 42-43% of the sample reported moderate (3), severe (4) or extreme (5) pain at both 3-months and 6-months after discharge from hospital (42.3% (n=61) at 3-months and 43.1% (n=91) at six months). At 6-months after injury, there was a proportion of participants who reported severe or extreme problems in all dimensions, and these exceeded the pre-injury estimated levels. This demonstrates that although the 6-month follow-up period was sufficient to observe recovery in a vast proportion of this sample, there were participants who reported extreme disability beyond the follow-up.

For peer review only - http://bmjopen.bmj.com/site/about/guidelines.xhtml

395 **Table 3:** Individual dimension and level data from EuroQoL-5D-(5L)

(n= (%))	No Problems	Slight Problems	Moderate Problems	Severe Problems	Unable/extreme Problems
**Mobility:**					
Pre-injury	204 (60.5)	50 (14.8)	47(13.9)	33 (9.8)	3 (0.9)
Discharge	50 (15.6)	78 (24.4)	102 (31.9)	72 (22.5)	18 (5.6)
One-Month	65 (31.0)	76 (36.2)	38 (18.1)	26 (12.4)	5 (2.4)
Three-Months	71 (49.3)	36 (25.0)	17 (11.8)	16 (11.1)	4 (2.8)
Six-Months	110 (52.1)	41 (19.4)	32 (15.2)	23 (10.9)	5 (2.4)
**Self-Care:**					
Pre-injury	269 (79.8)	33 (9.8)	23 (6.8)	10 (3.0)	2 (0.6)
Discharge	68 (21.3)	92 (28.8)	81 (25.3)	59 (18.4)	20 (6.3)
One-Month	108 (51.4)	59 (28.1)	30 (14.3)	10 (4.8)	3 (1.4)
Three-Months	106 (73.6)	21 (14.6)	9 (6.3)	6 (4.2)	2 (1.4)
Six-Months	153 (72.5)	31 (14.7)	20 (9.5)	5 (2.4)	2 (0.9)
**Usual Activities:**					
Pre-injury	224 (66.5)	53 (15.7)	33 (9.8)	19 (5.6)	8 (2.4)
Discharge	21 (6.6)	36 (11.3)	72 (22.5)	56 (17.5)	135 (42.2)
One-Month	20 (9.5)	95 (45.2)	41 (19.5)	29 (13.8)	25 (11.9)
Three-Months	49 (34.0)	51 (35.4)	22 (15.3)	9 (6.3)	13 (9.0)
Six-Months	89 (42.2)	62 (29.4)	34 (16.1)	14 (6.6)	12 (5.7)
**Pain/Discomfort:**					
Pre-injury	157 (46.6)	94 (27.9)	61 (18.1)	18 (5.3)	7 (2.1)
Discharge	14 (4.4)	41 (12.8)	151 (47.2)	74 (23.1)	40 (12.5)
One-Month	15 (7.1)	49 (23.3)	112 (53.3)	22 (10.5)	12 (5.7)
Three-Months	37 (25.7)	46 (31.9)	47 (32.6)	10 (6.9)	4 (2.8)
Six-Months	72 (34.1)	48 (22.7)	72 (34.1)	11 (5.2)	8 (3.8)
**Anxiety/Depression:**					
Pre-injury	188 (55.8)	79 (23.4)	54 (16.0)	11 (3.3)	5 (1.5)
Discharge	138 (43.1)	85 (26.6)	64 (20.0)	20 (6.3)	13 (4.1)
One-Month	100 (47.6)	38 (18.1)	57 (27.1)	7 (3.3)	8 (3.8)
Three-Months	82 (56.9)	27 (18.8)	26 (18.1)	4 (2.8)	5 (3.5)
Six-months	119 (56.4)	42 (19.9)	33 (19.9)	8 (3.8)	9 (4.3)

396

For peer review only - http://bmjopen.bmj.com/site/about/guidelines.xhtml

Evolution of chronic pain and pain with a neuropathic component

Subjective pain evaluated through the BPI Pain Severity Score (PSS) reflects the persistent levels of pain experienced by study participants throughout the 6-month study follow-up period. Pain related outcome measures are presented in **Table 2**. Although there were consistent incremental reductions in the mean pain score over time, at 6-months after injury the mean pain severity score was 2.7 (SD 2.7); 36.5% (n=77) of participants were reporting chronic pain. The mean BPI Pain Interference Score (PIS) also demonstrated incremental decreases until the 3-month timepoint where there appears to be a plateau, followed by a marginal deterioration of the mean PIS (2.6 (SD 2.7) to 2.7 (SD 2.9)). This highlights that participants reporting pain that impacted on daily activities at three months were likely to continue to experience these interferences at 6-months after hospital discharge. Similarly, mean self-reported analgesic effectiveness decreased from 65.3 (SD 24.8) at hospital discharge to 46.5 (SD 35.8) at 3-months which then plateaued and was 49.2 (SD 33.4) at 6-months after hospital discharge. Individual components of the BPI PSS and BPI PIS can be seen in the supplementary file **Table S3 and S4.**

At hospital discharge and 1-month after, 30.6% (n= 98) and 32.4% (68%) (respectively) of patients reported moderate or high risk of having a neuropathic pain component. At 3-months and 6-months, there was a reduction in the number of participants reporting neuropathic pain (23.6% (n=34) and 22.3% (n=47) respectively) with 13.2% (n=19) and 9.0% (n=19) respectively reporting PainDetect scores in the high-risk category (representing 90% likelihood of having a neuropathic pain component).

Development of Predictive models for the three outcomes of interest

Table 4 highlights three models developed in this study, one for each outcome. Initially candidate predictors were selected from variables readily available within the inpatient clinical journey (including hospital discharge), as this would be most useful in clinical practice. Unfortunately, we were unable to isolate a model with acceptable levels of discrimination and calibration using these variables alone. In a second approach models were developed using variables from the post discharge follow-up period (Table 4). Three models with acceptable levels of calibration and discrimination were identified. All models require measures collected at hospital discharge, 1-month, and 3-months after discharge to predict the outcomes of interest.

Model 1: Poor physical function at 6-months after hospital discharge was predicted using the following: the combination of the measures of physical function both prior to injury and at 3-months

For peer review only - http://bmjopen.bmj.com/site/about/guidelines.xhtml
For peer review only

after injury, a measurement of pain levels at 1-month after discharge, and a measurement of the impact of pain on the individuals walking ability at hospital discharge. **Model 2:** It was possible to predict chronic pain at 6-months after hospital discharge using the follow variables: Pre-injury regular use of analgesic agents, measures of physical function before the injury and at 3-months after discharge, and a measurement of pain severity at 1- and 3-months after discharge. **Model 3:** neuropathic pain was predicted using the following variables: starting Pregabalin during the acute hospital admission, the Mental Component Score at 1-month and the impact of pain on mood at 3-months after discharge, and the outcome of taking the PainDetect Questionnaire at 3-months after discharge.

For peer review only

For peer review only - http://bmjopen.bmj.com/site/about/guidelines.xhtml

Table 4: Predictive models for three outcomes of interest

	Co-efficient	Std error	Odds Ratio	P-value	95% CI
**Model 1: Poor physical function at 6-months after hospital discharge**					
Pre-Injury PCS (Range: 0-100) (1 step increment increase)	-0.093	0.019	0.911	<0.001	0.878-0.945
Current Pain at 1-month after injury (Range 0-10) (1 step increment increase)	0.224	0.078	1.252	0.004	1.075-1.457
PCS at 3 months (Range: 0-100) (1 step increment increase)	-0.040	0.014	0.960	0.005	0.934-0.988
Impact of pain on walking at discharge (Range: 0-10) (1 step increment increase)	0.139	0.057	1.149	0.015	1.027-1.285
Constant	*3.745*	*1.132*	-	*0.001*	-
**Model 2: Chronic pain at 6-months after hospital discharge**					
Regular Analgesia use before injury	1.813	0.533	6.130	0.001	2.158-17.414
Pre-Injury PCS (Range: 0-100) (1 step increment increase)	-0.039	0.019	0.962	0.044	0.926-0.999
Pain score at 1 month after discharge (Range: 0-10) (1 step increment increase)	0.270	0.089	1.310	0.003	1.099-1.560
PCS at 3 months (Range: 0-100) (1 step increment increase)	-0.051	0.015	0.951	0.001	0.922-0.980
Current Pain score 3 months after discharge (Range: 0-10) (1 step increment increase)	0.219	0.084	1.245	0.009	1.057-1.467
Constant	*1.361*	*1.098*	-	*0.215*	-
**Model 3: Likely neuropathic component to pain at 6-months after hospital discharge**					
Commenced on Pregabalin during Hospital admission	2.340	1.156	10.377	0.043	1.076-100.113
MCS at 1 month after discharge (Range: 0-100) (1 step increment increase)	-0.058	0.026	0.944	0.026	0.897-0.993
Pain Detect score at 3 months (Range: -1-38) (1 step increment increase)	0.174	0.043	1.190	<0.001	1.094-1.295
Impact of Pain on Mood at 3 months after discharge (Range 0-10) (1 step increment increase)	0.192	0.095	1.212	0.043	1.212-1.006
Constant	*-1.421*	*1.188*	-	*0.232*	-

Insert Figure 1 and Figure 2 here please.

For peer review only

For peer review only - http://bmjopen.bmj.com/site/about/guidelines.xhtml

The ROC diagrams for each model are presented in **Figure 1**. **Figure 2** presents the graphitised calibration plots whereby plot points present risk of outcome in tenths of patients with similar predicted probabilities. The line highlights the relationship between the observed frequency and the predicted probability of developing a poor outcome. **Model 1** for predicting poor physical functioning at 6 months after discharge had a c-statistic (area under the receiver operator curve) of 0.846 (Std error: 0.028; p<0.001; 95%CI 0.790-0.901) with a Hosmer and Lemeshow Chi-square value of 5.570 (p=0.695) with 8 degrees of freedom (df), demonstrating acceptable goodness-of-fit. This resulted in a model with a sensitivity of 79.0% and a specificity of 81.2%. **Model 2** for predicting chronic pain at 6-months after discharge had a c-statistic of 0.878 (Std error: 0.023; p<0.001; 95%CI 0.833-0.923) with a Hosmer and Lemeshow Chi-Square value of 6.778 (p=0.561) with 8 degrees of freedom (df), also demonstrating acceptable goodness-of-fit. This resulted in a model with a sensitivity of 63.6% and a specificity of 86.5%. Finally, **Model 3** for predicting pain with a neuropathic component had a c-statistic of 0.910 (Std error: 0.027; <0.001; 95%CI 0.857-0.964) with a Hosmer and Lemeshow Chi-square value of 2.221 (p=0.912) with 8 degrees of freedom (df), demonstrating acceptable goodness-of-fit. This resulted in a model with a sensitivity of 61.3% and a specificity of 92.7%. The overall accuracy of models 1-3 were 80.6%, 78.1% and 85.8% respectively. The calibration plots for each model **(Fig. 2)** demonstrated acceptable levels of calibration as many of the plots were close to the 45-degree line.

Discussion:

This study investigated changes in self-reported HRQoL and pain outcomes over a 6-month period from hospital discharge safter BTI. The study has provided new insights into the recovery of this patient group within the UK. This study has identified the high levels of pain and poor physical function experienced after BTI in the UK and measured the presence of neuropathic pain components within this population. Although it was not possible to construct a predictive model using only clinically available baseline and discharge level variables, the process of predictive prognostic modelling has identified factors that predict outcomes. This suggests that it is possible to develop a risk stratification score, but this involves using variables which are not routinely available in clinical practice currently. It is acknowledged there is limited clinical utility in these current models as the data required to achieve accurate predictions of outcomes is not routinely available in clinical practice. Further research is needed to understand how clinically useful prediction models can be developed for this population. There is potential, with refinement, to make the current prediction models more clinically useful, and there are ways that this score could have clinical utility in

For peer review only - http://bmjopen.bmj.com/site/about/guidelines.xhtml

identifying patients at risk of poor functional outcomes or chronic/neuropathic pain, subject to external validation in a future study. In respect to the models presented in this study, it is important to remember that good model performance in a development sample does not necessarily mean good predictive abilities in a new external validation dataset.

The limitations of using generic Patient Reported Outcome Measures (PROMs) to measure HRQoL have been reported previously.[7 32] Although validated within the major trauma population, it remains unclear how specific and comprehensive generic outcome measures (e.g., SF-12 and EQ5D-5L) are in groups like the BTI population. Although a trauma PROM has previously been developed,[33] there is a need for injury specific measures, particularly where unique outcomes have been identified.[7 34] Physical functioning was measured at a global level in this study using the SF-12 PCS and using the individual levels of the EuroQoL-5d-5L questionnaire. Both measures highlighted the extent of the altered physical function experienced by this population, which had not returned to their perceived baseline level of function at 6-months. In previous non-UK research, sequela related to BTI has been measured up to 2-years after injury highlighting the long-term impact of BTI.[35] Due to funding, it was not possible to extend the follow-up in the current study, but the 6-month timepoint was clinically ideal for the prognostic modelling process, particularly as Marasco et al. highlighted that patients reporting poor physical function at 6-months after injury reported poor physical function at 1 year and greater odds of reporting poor physical function at 2-years after injury.[35]

One key factor that influences HRQoL after BTI is pain, and the efficacy of pain management.[6] Measures of pain severity within this study highlighted 43.1% (n=91) of participants reported pain that was moderate, severe, or extreme at 6-months after hospital discharge with a mean Pain Severity Score of 2.7 (SD 2.7). For these participants, the mean analgesic effectiveness was 49.2 (SD 33.4) demonstrating how a chronic pain state existed where analgesic agents were not effective in managing pain. The variables in model 2 demonstrate the complexity in chronic pain management particularly in identifying those at risk through the predictive modelling process. Within this model it was not possible to predict the likelihood of whether a participant would experience chronic pain at 6-months after discharge using clinically available variables only. The final model included variables from pre-injury estimates through to 3-months after discharge which highlighted the need to alter our current model for patient follow-up to enable measurements at 1 and 3 months after discharge. Unsurprisingly, participants using regular analgesic agents prior to their injury had an odds ratio greater than six highlighting the need to identify patients with pre-existing chronic pain states during hospital admission as these patients are most likely to have ongoing injury related pain requirements at six months after hospital discharge.

For peer review only

For peer review only - http://bmjopen.bmj.com/site/about/guidelines.xhtml

There were greater odds of reporting neuropathic pain at 6 months if the participant had been initiated on a neuropathic pain agent (e.g. pregabalin) during the acute hospital admission. This suggests that clinical teams and specialist pain teams are accurately identifying patients at risk of neuropathic pain and commencing treatment. This unfortunately also highlights that these patients are still progressing to develop symptoms of neuropathic pain at six-months after discharge which suggests that greater input from specialist pain services after hospital discharge may be required to manage this pain trajectory. The model for neuropathic pain highlights an association with the MCS of the SF-12 at one month and the effect of pain on mood at 3-months after hospital discharge. The relationship between neuropathic pain and mental health has previously been identified in the non-trauma literature.[36] Although there was a less substantial drop in SF-12 MCS compared to the PCS and this returned to the pre-injury baseline within the 6-month follow-up period, this shows the importance of measuring mental wellbeing as both a part of HRQoL and as an important predictor of neuropathic pain.

This study has developed a model for each of the three outcomes of interest: physical function, chronic pain, and neuropathic pain at 6-months after hospital discharge with BTI. The next phase of the modelling process will be to conduct a further study to validate the predictive ability of the models within an external sample of participants with BTI. This study will further explore the follow-up needs of patients with BTI and look at effective ways of collecting these data needed to predict 6-month outcomes. It will also be of interest to investigate these outcomes beyond the 6-months follow-up period used in this study. In the meantime, these predictive variables have clinical utility for clinicians reviewing patients during the follow-up period in primary and secondary care settings and these variables may be useful in highlighting patients at risk who need referral to specialist services (e.g., physiotherapy or specialist pain services).

Strengths and limitations of this study:

Although we recruited a moderate sized sample using the exploratory approach to this study, we did not achieve the aim of recruiting 100 events relating to our three outcomes of interest. Care was taken to ensure that that the number of variables inputted into each model were justified by the number participants reporting the outcomes of interest. There are reports within the literature that the arbitrary rule of 10 cases per variable does not necessarily negatively impact on the model fit.[37] During subsequent validation of the models, care will be taken to assess for overfitting of the models but from the model diagnostics presented here, there is no suggestion of overfitting within the current sample.[31] It is important to remember that the final models are to some degree variable selection method dependent. In an ideal world, it would have been beneficial to use more than one

For peer review only - http://bmjopen.bmj.com/site/about/guidelines.xhtml

method of selection but in the case of this study, it was not possible to undertake backward selection because of concerns of overfitting the model.

Overall, the combined response rate of 72% at all timepoints is comparable to equivalent studies undertaken previously in this area of trauma research.[6] Despite this, the response rate was a limitation as the key outcomes were measured at the 6-month timepoint resulting in only 63% (n=211) of the overall recruited sample being included in the modelling process. For further studies investigating outcomes within a longitudinal follow-up process, it will be important to use these data to consider potential sample sizes required to adequately power these studies.

Although multiple imputation was used in the management of missingness for independent variables, it is acknowledged that the case-wise deletion approach used in the management of missingness in the outcome data is a limitation in this study. In the presence of MAR data, this approach results in large proportions of data being discarded, which results in the introduction of potential bias, and reduced precision and power. For this reason, and despite there being no clinically significant differences between the characteristics of responders and non-responders, the findings of this study should be interpreted cautiously. Despite all efforts to maximise response rates at all timepoints, future longitudinal studies in the BTI population will need to consider other ways of accessing patients to facilitate complete follow-up data collection in a geographically diverse population.

As subjective outcome measures have been used throughout this study, it is important to acknowledge the risk of bias which is inherent within these measures, particularly in relation to participants selecting the extremes of a rating scale.[38] Care has been taken within the study to control this bias through the instructions given to patients prior to completing each questionnaire and investigation of outliers within the sample during data analysis.[39] In future research, it would be interesting to compare the predictive ability of both subjective outcomes (i.e. SF-12 PCS for physical function) with an objective measure (grip strength testing as an estimate of physical functional ability for example) and compare the difference in the predictive functions of these outcomes.[40]

Finally, although most of the study was undertaken before the Covid-19 pandemic began in the UK, there will be seen and unseen impacts of the Covid-19 pandemic on this study.[41] The final three sites closed to recruitment 2 to 4 weeks early causing them to not meet recruitment targets. Furthermore, follow-up continued throughout the first wave of the pandemic in the UK. Although we were not able to collect data relating Covid-19 infection amongst our sample, we were not informed that any participants had been infected with the virus. Despite this, there is potential bias

For peer review only - http://bmjopen.bmj.com/site/about/guidelines.xhtml

603 relating to lockdown, social isolation that remains unmeasured within our sample which may have impacted on the self-reported outcome measures reported in this study.[42]

Conclusions:

It remains vital that the interdisciplinary team do not underestimate the impact of the long-term outcomes associated with BTI, particularly relating to physical functioning, chronic pain, and neuropathic pain. Although predictive models were successfully developed, this study has demonstrated the complexity and challenge of developing models which are clinically useful as many of the variables included in these final models are not routinely available in practice. Despite this, the study has shown how predictive models can be developed for outcomes and highlight how PROMS can be used to develop prognostic models particularly in longitudinal processes where objective clinical data is not routinely collected. Ultimately, these models add to our understanding of which factors influence the development of sequela after hospital discharge with BTI and after external validation, will have clinical utility in the risk stratification of high-risk patient groups.

For peer review only - http://bmjopen.bmj.com/site/about/guidelines.xhtml

Reference:

1. Baker EJ, Lee GA. A Retrospective Observational Study Examining the Effect of Thoracic Epidural and Patient Controlled Analgesia on Short-term Outcomes in Blunt Thoracic Trauma Injuries. *Medicine (Baltimore)* 2016;95(2):e2374. doi: 10.1097/MD.0000000000002374
2. Battle C, Hutchings H, Lovett S, et al. Predicting outcomes after blunt chest wall trauma: development and external validation of a new prognostic model. *Crit Care* 2014;18(3):R98. doi: 10.1186/cc13873 [published Online First: 2014/05/14]
3. de Graaf MW, Reininga IHF, Heineman E, et al. The development and internal validation of a model to predict functional recovery after trauma. *PLoS One* 2019;14(3):e0213510. doi: 10.1371/journal.pone.0213510 [published Online First: 2019/03/14]
4. Cole E, Lecky F, West A, et al. The Impact of a Pan-regional Inclusive Trauma System on Quality of Care. *Ann Surg* 2016;264(1):188-94. doi: 10.1097/SLA.0000000000001393
5. Martino C, Russo E, Santonastaso DP, et al. Long-term outcomes in major trauma patients and correlations with the acute phase. *World J Emerg Surg* 2020;15:6. doi: 10.1186/s13017-020-0289-3 [published Online First: 2020/01/13]
6. Baker E, Xyrichis A, Norton C, et al. The long-term outcomes and health-related quality of life of patients following blunt thoracic injury: a narrative literature review. *Scand J Trauma Resusc Emerg Med* 2018;26(1):67. doi: 10.1186/s13049-018-0535-9 [published Online First: 2018/08/17]
7. Craxford S, Deacon C, Myint Y, et al. Assessing outcome measures used after rib fracture: A COSMIN systematic review. *Injury* 2019;50(11):1816-25. doi: 10.1016/j.injury.2019.07.002 [published Online First: 2019/07/04]
8. Wilson IB, Cleary PD. Linking clinical variables with health-related quality of life. A conceptual model of patient outcomes. *JAMA* 1995;273(1):59-65.
9. Ojelabi AO, Graham Y, Haighton C, et al. A systematic review of the application of Wilson and Cleary health-related quality of life model in chronic diseases. *Health Qual Life Outcomes* 2017;15(1):241. doi: 10.1186/s12955-017-0818-2 [published Online First: 2017/12/11]
10. Baker E, Woolley A, Xyrichis A, et al. How does the implementation of a patient pathway-based intervention in the acute care of blunt thoracic injury impact on patient outcomes? A systematic review of the literature. *Injury* 2020 doi: 10.1016/j.injury.2020.06.002 [published Online First: 2020/06/04]
11. Karimi M, Brazier J. Health, Health-Related Quality of Life, and Quality of Life: What is the Difference? *Pharmacoeconomics* 2016;34(7):645-9. doi: 10.1007/s40273-016-0389-9
12. Vernon D, Brown JE, Griffiths E, et al. Reducing readmission rates through a discharge follow-up service. *Future Healthc J* 2019;6(2):114-17. doi: 10.7861/futurehosp.6-2-114
13. Braaf S, Ameratunga S, Nunn A, et al. Patient-identified information and communication needs in the context of major trauma. *BMC Health Serv Res* 2018;18(1):163. doi: 10.1186/s12913-018-2971-7 [published Online First: 2018/03/07]
14. Keene DJ, Vadher K, Willett K, et al. Predicting patient-reported and objectively measured functional outcome 6 months after ankle fracture in people aged 60 years or over in the UK: prognostic model development and internal validation. *BMJ Open* 2019;9(7):e029813. doi: 10.1136/bmjopen-2019-029813 [published Online First: 2019/07/23]
15. Ware J, Kosinski M, Keller SD. A 12-Item Short-Form Health Survey: construction of scales and preliminary tests of reliability and validity. *Med Care* 1996;34(3):220-33.
16. Devlin NJ, Parkin D, Browne J. Patient-reported outcome measures in the NHS: new methods for analysing and reporting EQ-5D data. *Health Econ* 2010;19(8):886-905. doi: 10.1002/hec.1608
17. Parkin D, Rice N, Devlin N. Statistical analysis of EQ-5D profiles: does the use of value sets bias inference? *Med Decis Making* 2010;30(5):556-65. doi: 10.1177/0272989X09357473 [published Online First: 2010/03/12]

For peer review only

For peer review only - http://bmjopen.bmj.com/site/about/guidelines.xhtml

18. Kumar SP. Utilization of brief pain inventory as an assessment tool for pain in patients with cancer: a focused review. *Indian J Palliat Care* 2011;17(2):108-15. doi: 10.4103/0973-1075.84531
19. Freynhagen R, Baron R, Gockel U, et al. painDETECT: a new screening questionnaire to identify neuropathic components in patients with back pain. *Curr Med Res Opin* 2006;22(10):1911-20. doi: 10.1185/030079906X132488
20. Battle CE, Hutchings H, James K, et al. The risk factors for the development of complications during the recovery phase following blunt chest wall trauma: a retrospective study. *Injury* 2013;44(9):1171-6. doi: 10.1016/j.injury.2012.05.019 [published Online First: 2012/06/12]
21. Treede RD, Rief W, Barke A, et al. A classification of chronic pain for ICD-11. *Pain* 2015;156(6):1003-7. doi: 10.1097/j.pain.0000000000000160
22. Boonstra AM, Schiphorst Preuper HR, Balk GA, et al. Cut-off points for mild, moderate, and severe pain on the visual analogue scale for pain in patients with chronic musculoskeletal pain. *Pain* 2014;155(12):2545-50. doi: 10.1016/j.pain.2014.09.014 [published Online First: 2014/09/17]
23. Murnion BP. Neuropathic pain: current definition and review of drug treatment. *Aust Prescr* 2018;41(3):60-63. doi: 10.18773/austprescr.2018.022 [published Online First: 2018/06/01]
24. Mathieson S, Lin C. painDETECT questionnaire. *J Physiother* 2013;59(3):211. doi: 10.1016/S1836-9553(13)70189-9
25. Unsworth A, Curtis K, Asha SE. Treatments for blunt chest trauma and their impact on patient outcomes and health service delivery. *Scand J Trauma Resusc Emerg Med* 2015;23:17. doi: 10.1186/s13049-015-0091-5 [published Online First: 2015/02/08]
26. Kourouche S, Buckley T, Munroe B, et al. Development of a blunt chest injury care bundle: An integrative review. *Injury* 2018;49(6):1008-23. doi: 10.1016/j.injury.2018.03.037 [published Online First: 2018/04/07]
27. von Elm E, Altman DG, Egger M, et al. The Strengthening the Reporting of Observational Studies in Epidemiology (STROBE) Statement: guidelines for reporting observational studies. *Int J Surg* 2014;12(12):1495-9. doi: 10.1016/j.ijsu.2014.07.013 [published Online First: 2014/07/18]
28. Collins GS, Reitsma JB, Altman DG, et al. Transparent reporting of a multivariable prediction model for individual prognosis or diagnosis (TRIPOD): the TRIPOD statement. *BMJ* 2015;350:g7594. doi: 10.1136/bmj.g7594 [published Online First: 2015/01/07]
29. Pareek N, Kordis P, Beckley-Hoelscher N, et al. A practical risk score for early prediction of neurological outcome after out-of-hospital cardiac arrest: MIRACLE2. *Eur Heart J* 2020 doi: 10.1093/eurheartj/ehaa570 [published Online First: 2020/07/30]
30. Shipe ME, Deppen SA, Farjah F, et al. Developing prediction models for clinical use using logistic regression: an overview. *J Thorac Dis* 2019;11(Suppl 4):S574-S84. doi: 10.21037/jtd.2019.01.25
31. Alba AC, Agoritsas T, Walsh M, et al. Discrimination and Calibration of Clinical Prediction Models: Users' Guides to the Medical Literature. *JAMA* 2017;318(14):1377-84. doi: 10.1001/jama.2017.12126
32. Hoffman K, Cole E, Playford ED, et al. Health outcome after major trauma: what are we measuring? *PLoS One* 2014;9(7):e103082. doi: 10.1371/journal.pone.0103082 [published Online First: 2014/07/22]
33. Attenberger C, Amsler F, Gross T. Clinical evaluation of the Trauma Outcome Profile (TOP) in the longer-term follow-up of polytrauma patients. *Injury* 2012;43(9):1566-74. doi: 10.1016/j.injury.2011.01.002 [published Online First: 2011/01/20]
34. Rosenberg GM, Stave C, Spain DA, et al. Patient-reported outcomes in trauma: a scoping study of published research. *Trauma Surg Acute Care Open* 2018;3(1):e000202. doi: 10.1136/tsaco-2018-000202 [published Online First: 2018/09/04]

For peer review only - http://bmjopen.bmj.com/site/about/guidelines.xhtml

35. Marasco S, Lee G, Summerhayes R, et al. Quality of life after major trauma with multiple rib fractures. *Injury* 2015;46(1):61-5. doi: 10.1016/j.injury.2014.06.014 [published Online First: 2014/06/21]
36. Torta R, Ieraci V, Zizzi F. A Review of the Emotional Aspects of Neuropathic Pain: From Comorbidity to Co-Pathogenesis. *Pain Ther* 2017;6(Suppl 1):11-17. doi: 10.1007/s40122-017-0088-z [published Online First: 2017/11/24]
37. Ogundimu EO, Altman DG, Collins GS. Adequate sample size for developing prediction models is not simply related to events per variable. *J Clin Epidemiol* 2016;76:175-82. doi: 10.1016/j.jclinepi.2016.02.031 [published Online First: 2016/03/08]
38. Dowling NM, Bolt DM, Deng S, et al. Measurement and control of bias in patient reported outcomes using multidimensional item response theory. *BMC Med Res Methodol* 2016;16:63. doi: 10.1186/s12874-016-0161-z [published Online First: 2016/05/26]
39. Tempelaar D, Rienties B, Nguyen Q. Subjective data, objective data and the role of bias in predictive modelling: Lessons from a dispositional learning analytics application. *PLoS One* 2020;15(6):e0233977. doi: 10.1371/journal.pone.0233977 [published Online First: 2020/06/12]
40. Koskinen S, Hokkinen EM, Wilson L, et al. Comparison of subjective and objective assessments of outcome after traumatic brain injury using the International Classification of Functioning, Disability and Health (ICF). *Disabil Rehabil* 2011;33(25-26):2464-78. doi: 10.3109/09638288.2011.574776 [published Online First: 2011/05/03]
41. Baker E, Lee G. Reflecting on the challenges of sustaining emergency care research during the Covid-19 pandemic. *Int Emerg Nurs* 2020;51:100894. doi: 10.1016/j.ienj.2020.100894 [published Online First: 2020/07/13]
42. Baker E, Clark LL. Biopsychopharmacosocial approach to assess impact of social distancing and isolation on mental health in older adults. *Br J Community Nurs* 2020;25(5):231-38. doi: 10.12968/bjcn.2020.25.5.231

Figure Legends/Caption:

Figure 1: Receiver Operator Curve (ROC) for predictive models for three outcomes of interest

Figure 2: Calibration plot for model using expected and observed probabilities of developing poor outcomes

For peer review only - http://bmjopen.bmj.com/site/about/guidelines.xhtml

Declarations:

Author Contribution:

EB, GL, AX, CN, PH conceived and designed the study and developed the study protocol. AB, CB, EC, CD, JF, RJ, LP, AR, IS, AT, MT, & LY were local primary investigators and coordinated and contributed significantly to the recruitment and data collection processes. Data analysis was planned with EB, GL, TM & CB. Data analysis was undertaken by EB and reviewed by TM. EB developed the first draft of the manuscript and EC, CB, AX, CN, PH, & GL made subsequent revisions. All authors commented on and approved the manuscript prior to submission for publication. EB takes overall responsibility for the manuscript.

Acknowledgements:

The research team would like to acknowledge the study participants who gave up their time to participate in this interview study and the clinical research staff at each site who contributed to the participants recruitment and initial data collection for the Rib Injury Outcomes Study.

Conflict of Interest:

Nothing to declare.

Funding Statement:

Edward Baker is funded by a National Institute for Health Research (NIHR), Clinical Doctoral Research Fellowship for this research project (ICA-CDRF-2016-02-006). The publication presents independent research funded by the National Institute for Health Research (NIHR).

For peer review only - http://bmjopen.bmj.com/site/about/guidelines.xhtml

For peer review only

778 The views expressed are those of the authors and not necessarily those of the NHS, the
779 NIHR or the Department of Health and Social Care.

780 **Data Statement:**

781 The authors confirm that the data supporting the findings of this study are available within
782 the article and its supplementary materials.

783 **Ethical Approvals:**

784 Ethical approval was granted by the 'Hampshire A' South Central Research Ethics Committee
785 in June 2018 (ref: 18/SC/0230).

For peer review only

For peer review only - http://bmjopen.bmj.com/site/about/guidelines.xhtml

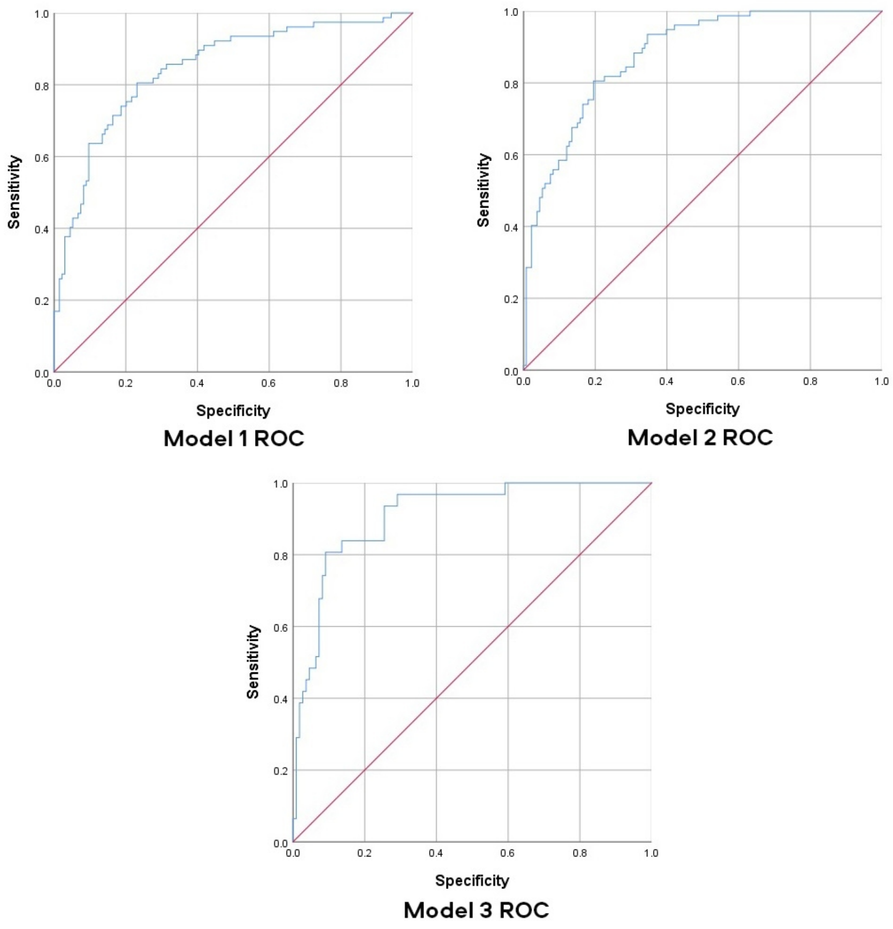

Figure 1: Receiver Operator Curve (ROC) for predictive models for three outcomes of interest

187x187mm (144 x 144 DPI)

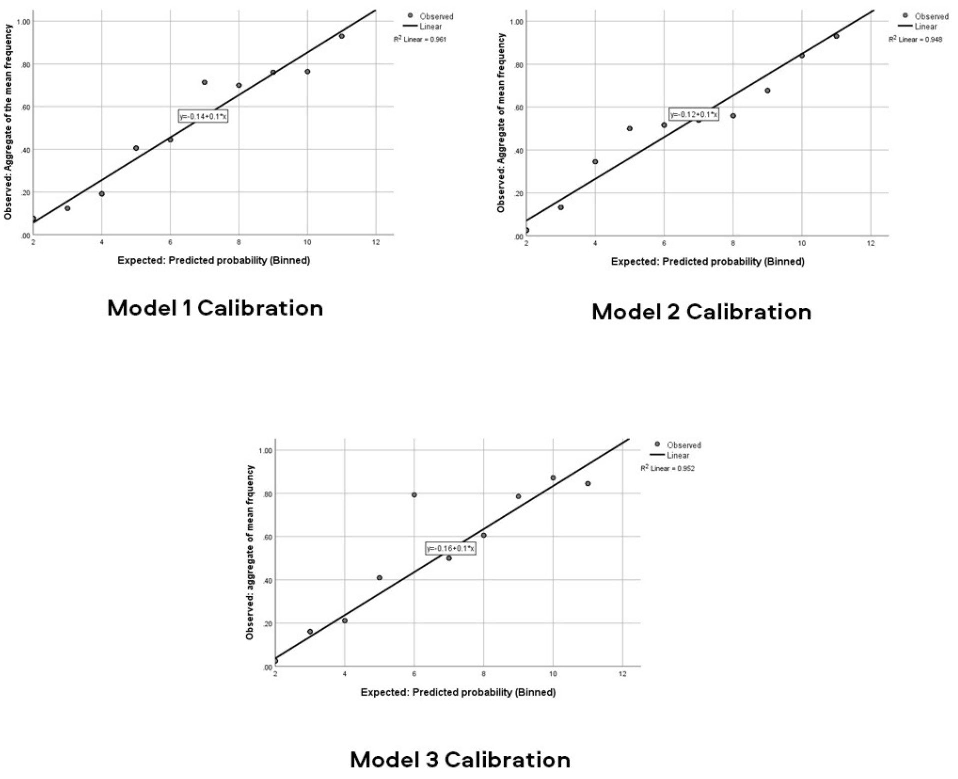

Figure 2: Calibration plots for models using expected and observed probabilities of developing poor outcomes of interest

187x187mm (144 x 144 DPI)

Supplementary file: Table S1: Extended Characteristics of the Sample/Candidate predictor variables

Result: n= (%) unless otherwise indicated	**Total sample** (n=337)	**6/12 PCS≤35** (n=77)	**6/12 PCS>35** (n=134)	p-value	**6/12 Pain Severity Score ≥3.5** (n=77)	**6/12 Pain Severity Score <3.5** (n=134)	p-value	**6/12 PDQ score ≥12** (n=47)	**6/12 PDQ score <12** (n=163)	p-value
Age (Mean (±SD))	62.0 (16.5)	69.1 (13.9)	60.8 (16.3)	<0.001*	66.7 (16.4)	62.1 (15.6)	0.05	63.4 (17.0)	63.9 (15.7)	0.80
Male	231 (68.5)	49 (63.6)	97 (72.4)	0.20	54 (70.1)	91 (67.9)	0.80	33 (70.2)	112 (68.7)	0.90
Injury Severity Score (Mean (±SD))	11.2 (7.3)	10.9 (7.8)	11.4 (6.8)	0.60	11.3 (7.9)	11.2 (6.8)	1.0	11.6 (8.3)	11.2 (6.9)	0.80
Battle/StUMBle Score (Mean (±SD))	22.2 (12.0)	23.8 (11.0)	21.8 (11.3)	0.20	23.9 (11.4)	21.8 (11.0)	0.20	24.2 (12.0)	22.1 (10.9)	0.30
**Mechanism of Injury:**										
RTC	78 (23.1)	14 (18.2)	38 (28.4)		19 (24.7)	33 (24.6)		9 (19.1)	43 (26.4)	
Crush	11 (3.3)	3 (3.9)	4 (3.0)		1 (1.3)	6 (4.5)		1 (2.1)	6 (3.7)	
Ped vs. Vehicle	4 (1.2)	1 (1.3)	2 (1.5)		1 (1.3)	2 (1.5)		1 (2.1)	2 (1.2)	
Fall>2m	69 (20.5)	13 (16.9)	30 (22.4)	0.20	18 (23.4)	25 (18.7)	0.80	13 (27.7)	30 (18.4)	0.80
Fall<2m	153 (45.4)	44 (57.1)	54 (40.3)		37 (48.1)	61 (45.5)		22 (46.8)	76 (46.6)	
Assault	14 (4.2)	1 (1.3)	4 (3.0)		1 (1.3)	3 (2.2)		1 (2.1)	3 (1.8)	
CPR	4 (1.2)	1 (1.3)	0		0	1 (0.7)		0	1 (0.6)	
Rapid deceleration	4 (1.2)	0	2 (1.5)		0	2 (1.5)		0	2 (1.2)	
**Pre-morbid state:**										
Respiratory	70 (20.8)	21 (27.3)	21 (15.7)	0.04*	21 (27.3)	20 (14.9)	0.03*	12 (25.5)	29 (17.8)	0.20
Cardiac	147 (43.6)	49 (63.6)	53 (39.6)	0.001*	47 (61.0)	54 (40.3)	0.004*	27 (57.4)	74 (45.4)	0.20
Neuro	50 (14.8)	20 (26.0)	9 (6.7)	<0.001*	19 (24.7)	10 (7.5)	0.001*	10 (21.3)	19 (11.7)	0.09
MSK	89 (26.4)	26 (33.8)	35 (26.1)	0.20	30 (39.0)	31 (23.1)	0.02*	16 (34.0)	45 (27.6)	0.40
Cancer Current	9 (2.7)	4 (5.2)	3 (2.2)	0.08	4 (5.2)	3 (2.2)	0.009*	3 (6.4)	4 (2.5)	0.40
Cancer Historical	26 (7.7)	12 (15.6)	10 (7.5)		14 (18.2)	8 (6.0)		4 (8.5)	18 (11.0)	
DM2	33 (9.8)	9 (11.7)	11 (8.2)	0.40	5 (6.5)	15 (11.2)	0.30	5 (10.6)	15 (9.2)	0.80
No of Comorbidities (Mean (±SD))	2.3 (2.2)	2.3 (0.3)	1.8 (0.2)	<0.001*	3.1 (2.2)	1.9 (2.0)	<0.001*	2.9 (2.5)	2.2 (2.1)	0.07
Chronic Pain	27 (8.0)	11 (14.3)	8 (6.0)	0.042*	13 (16.9)	6 (4.5)	0.003*	7 (14.9)	12 (7.4)	0.10
Regular Analgesic Use	61 (18.1)	23 (29.9)	15 (11.2)	0.001*	30 (39.0)	8 (6.0)	<0.001*	19 (40.4)	19 (11.7)	<0.001*

For peer review only

Smoking Status:										
Current smoker	73 (21.7)	11 (14.3)	21 (15.7)	0.90	10 (13.0)	22 (16.4)	0.07	11 (23.4)	21 (12.9)	0.05
Ex-Smoker	90 (26.7)	25 (32.5)	37 (27.6)		27 (35.1)	34 (25.4)		18 (38.3)	43 (26.4)	
Never smoked	122 (36.2)	30 (39.0)	52 (67.5)		23 (29.9)	59 (44.0)		14 (29.8)	68 (41.7)	
Smoking Status unknown	50 (14.8)	11 (14.3)	23 29.9)		17 (22.1)	17 (12.7)		4 (8.5)	30 (18.4)	
Pack years (Mean (±SD))	22.1 (18.4)	27.6 (14.2)	20.3 (18.8)	0.1	19.9 (14.3)	24.1 (19.2)	0.4	21.2 (14.8)	23.3 (18.9)	0.7
**Indicators of Injury Severity:**										
No. Rib Fractures (Mean (±SD))	5.2 (4.0)	5.3 (3.8)	5.3 (4.0)	0.90	5.4 (3.9)	5.3 (3.9)	0.70	5.6 (4.1)	5.2 (3.8)	0.60
Rib fracture Categories:										
0	19 (5.6)	5 (6.5)	8 (6.0)	0.30	6 (7.8)	7 (5.2)	0.80	1 (2.1)	12 (7.4)	0.70
1-4	160 (47.5)	39 (50.6)	58 (43.3)		33 (42.9)	63 (47.0)		25 (53.2)	71 (43.6)	
5-9	114 (33.8)	22 (28.6)	52 (38.8)		46 (59.7)	28 (20.9)		14 (29.8)	60 (36.8)	
10-15	29 (8.6)	9 (11.7)	9 (6.7)		7 (9.1)	11 (8.2)		5 (10.6)	13 (8.0)	
15+	15 (4.5)	2 (2.6)	7 (5.2)		3 (2.9)	6 (4.5)		2 (4.3)	7 (4.3)	
Other Indicators of Severity:										
Operative Rib Fixation	16 (4.7)	6 (7.8)	3 (2.2)	0.05*	2 (2.6)	7 (5.2)	0.40	3 (6.4)	6 (3.7)	0.40
First Rib fracture	31 (9.2)	6 (7.8)	13 (9.7)	0.60	10 (13.0)	9 (6.7)	0.10	7 (14.9)	12 (7.4)	0.10
Bilateral rib fractures	44 (13.1)	9 (11.7)	19 (14.2)	0.60	11 (14.3)	17 (12.7)	0.80	10 (21.3)	18 (11.0)	0.07
Scapular fracture	27 (8.0)	9 (11.7)	11 (8.2)	0.40	9 (11.7)	11 (8.2)	0.40	4 (8.5)	16 (9.8)	0.90
Clavicle fracture	29 (8.6)	8 (10.4)	14 (10.4)	0.50	8 (10.4)	14 (10.4)	0.50	4 (8.5)	18 (11.0)	0.08
Sternum Fracture	26 (7.7)	4 (5.2)	13 (9.7)	0.30	5 (6.5)	12 (9.0)	0.50	2 (4.3)	15 (9.2)	0.30
Manubrium Fracture	8 (2.4)	1 (1.3)	4 (3.0)	0.40	2 (2.6)	3 (2.2)	0.90	1 (2.1)	4 (2.5)	0.90
Thoracic Process #	31 (9.2)	6 (7.8)	12 (9.0)	0.80	11 (14.3)	7 (5.2)	0.02*	5 (10.6)	13 (8.0)	0.60
No. of Thoracic Process # (Mean (±SD))	3.3 (2.8)	2.2 (1.3)	2.8 (1.4)	0.40	2.8 (1.5)	2.3 (1.2)	0.50	2.5 (1.3)	2.7 (1.5)	0.80
Lung Contusion	76 (22.6)	14 (18.2)	32 (23.9)	0.30	9 (11.7)	36 (26.9)	0.009*	8 (17.0)	37 (22.7)	0.40
Pneumothorax	107 (31.8)	20 (26.0)	50 (37.3)	0.20	20 (26.0)	50 (37.3)	0.20	15 (31.9)	55 (33.7)	0.90
Haemothorax	80 (23.7)	15 (19.5)	38 (28.4)	0.20	14 (18.2)	38 (28.4)	0.90	5 (10.6)	47 (28.8)	0.01*
Massive Haemothorax	4 (1.2)	0	1 (0.7)	0.50	1 (1.3)	0	0.20	0	1 (0.6)	0.60
Haemopneumothorax	45 (13.4)	7 (9.1)	23 (17.2)	0.10	21 (27.3)	8 (6.0)	0.30	4 (8.5)	25 (15.3)	0.20
Radiological Flail	60 (17.8)	13 (16.9)	29 (21.6)	0.40	16 (20.8)	26 (19.4)	0.80	7 (14.9)	35 (21.5)	0.30
Extra-thoracic injuries	104 (30.9)	25 (32.5)	35 (26.1)	0.30	19 (24.7)	40 (29.9)	0.40	12 (25.5)	47 (28.8)	0.70

**Initial Vital Signs:**										
Respiratory Rate (Mean (±SD))	19.6 (±4.6)	20.6 (4.8)	18.9 (4.1)	0.01*	20.1 (5.1)	19.2 (4.0)	0.20	20.2 (4.5)	19.3 (4.4)	0.30
Oxygen Saturation (%) (Mean (±SD))	95.9 (4.7)	95.4 (4.4)	96.4 (3.6	0.10	95.6 (3.1)	96.3 (4.3)	0.20	95.8 (3.1)	96.1 (4.1)	0.70
Heart Rate (Mean (±SD))	81.5 (19.3)	81.8 (4.1)	79.4 (17.3)	0.40	82.5 (22.1)	79.0 (17.3)	0.20	83.9 (17.2)	79.3 (19.6)	0.10
Systolic Blood Pressure (Mean (±SD))	142.5 (23.0)	144.8 (23.9)	141.7 (22.3)	0.40	147.9 (25.8)	140.2 (20.7)	0.03*	148.7 (25.3)	141.3 (22.0)	0.09
Temperature (Mean (±SD))	36.6 (4.2)	37.4 (7.5)	36.5 (0.7)	0.30	37.4 (7.7)	36.5 (0.7)	0.20	36.4 (0.5)	37.0 (5.3)	0.20
**Chest Drain Management:**										
Intercostal Drain	67 (19.9)	14 (18.2)	33 (24.6)	0.30	14 (18.2)	32 (23.9)	0.30	9 (19.1)	37 (22.7)	0.60
Duration of Intercostal Drain (days) (Mean (±SD))	4.9 (3.2)	4.6 (2.6)	5.0 (3.8)	0.70	6.5 (4.7)	4.4 (2.9)	0.20	4.7 (1.4)	5.0 (3.8)	0.70
**Admission Level of Care (1-3):**										
Level 1	252 (74.8)	59 (76.6)	106 (79.1)		60 (77.9)	104 (77.6)		35 (74.5)	129 (79.1)	
Level 2	65 (19.3)	13 (16.9)	26 (19.4)	0.10	13 (16.9)	26 (19.4)	0.50	9 (19.1)	30 (18.4)	0.40
Level 3	18 (5.3)	5 (6.5)	2 (1.5)		4 (5.2)	3 (2.2)		3 (6.4)	4 (2.5)	
**Initial Emergency Department analgesic management:**										
None	28 (8.3)	6 (7.8)	14 (10.4)		6 (7.8)	14 (10.4)		2 (4.3)	18 (11.0)	
Opioid	207 (61.4)	47 (61.0)	79 (59.0)	0.80	42 (54.5)	83 (61.9)	0.30	27 (57.4)	98 (60.1)	0.20
Non-opioid	102 (30.3)	24 (31.2)	41 (30.6)		29 (37.7)	36 (26.9)		18 (38.3)	47 (28.8)	
**Inpatient Analgesic management:**										
Intermittent analgesia only	179 (53.1)	37 (48.1)	77 (57.5)	0.20	37 (48.1)	76 (56.7)	0.20	22 (46.8)	91 (55.8)	0.30
Regional Analgesia	106 (31.5)	29 (37.7)	41 (30.6)	0.30	31 (40.3)	39 (29.1)	0.10	20 (42.6)	50 (30.7)	0.10
Thoracic Epidural	50 (14.8)	13 (16.9)	23 (17.2)	0.90	14 (18.2)	22 (16.4)	0.80	8 (17.0)	28 (17.2)	1.0
Time to commence TE (hrs) (Mean (±SD))	27.7 (53.0)	23.2 (26.5)	37.2 (76.9)	0.50	53.5 (92.6)	18.3 (26.0)	0.10	15.1 (14.9)	36.9 (69.8)	0.20
Paravertebral block	16 (4.7)	5 (6.5)	5 (3.7)	0.40	8 (10.4)	2 (1.5)	0.004*	4 (8.5)	6 (3.7)	0.20
Time to commence PVB (hrs) (Mean (±SD))	20.4 (17.3)	11.8 (8.8)	29.3 (27.7)	0.40	12.5 (8.6)	-	-	-	-	-
Intercostal Nerve Block	4 (1.2)	3 (3.9)	0	0.02	1 (1.3)	2 (1.5)	0.90	1 (2.1)	2 (1.2)	0.50
Time to commence Intercostal Nerve Block (hrs) (Mean (±SD))	65.3 (57.6)	39.0 (28.9)	-	-	-	-	-	-	-	-
Serratus Plane Block	22 (6.5)	4 (5.2)	14 (10.4)	0.5	4 (5.2)	10 (7.5)	0.50	2 (4.3)	12 (7.4)	0.70

For peer review only

Time to commence SPB (hrs) (Mean (±SD))	17.6 (19.5)	34.8 (36.9)	13.6 (8.5)	0.10	17.6 (6.4)	20.4 (25.5)	0.80	14.3 (2.5)	20.5 (23.2)	0.40
Erector Spinae Block	20 (5.9)	6 (7.8)	4 (3.0)	0.10	7 (9.1)	3 (2.2)	0.03*	5 (10.6)	5 (3.1)	0.03*
Time to commence Erector Spinae Block (hrs) (Mean (±SD))	30.4 (19.8)	25.8 (29.5)	29.5 (27.3)	1.0	27.0 (22.5)	25.8 (-)	1.0	31.0 (32.0)	24.4 (24.7)	0.60
Combined Region Analgesia with Patient Controlled Analgesic	43 (12.8)	11 (14.3)	14 (10.4)	0.40	10 (13.0)	15 (11.2)	0.70	6 (12.8)	19 (11.7)	0.80
Patient Controlled Analgesic only	93 (27.6)	23 (29.9)	29 (21.6)	0.20	20 (26.0)	32 (23.9)	0.80	12 (25.5)	40 (24.5)	0.90
Time to commence PCA (hrs) (Mean (±SD))	16.2 (23.9)	12.4 (10.9)	12.7 (12.4)	0.90	12.8 (12.2)	12.5 (11.6)	0.90	14.1 (16.3)	12.1 (10.0)	0.70
**Other Analgesics:**										
Gabapentin	37 (11.0)	11 (14.3)	11 (8.2)	0.20	11 (14.3)	11 (8.2)	0.20	9 (19.1)	13 (8.0)	0.03*
Pregabalin	15 (4.5)	5 (6.5)	4 (3.0)	0.20	7 (9.1)	2 (1.5)	0.009*	6 (12.8)	3 (1.8)	0.001*
Amitriptyline	16 (4.7)	7 (9.1)	3 (2.2)	0.02*	7 (9.1)	3 (2.2)	0.03*	5 (10.6)	5 (3.1)	0.03*
Transdermal Lignocaine	113 (33.5)	28 (36.4)	43 (32.1)	0.50	27 (35.1)	44 (32.8)	0.80	12 (25.5)	59 (36.2)	0.20
**Discharge Analgesia:**										
Opioid	220 (65.3)	51 (66.2)	95 (70.9)	0.48	55 (71.4)	90 (67.2)	0.60	33 (70.2)	112 (68.7)	0.90
Neuropathic Pain Agent	41 (12.2)	14 (18.2)	11 (8.2)	0.03*	12 (15.6)	13 (9.7)	0.20	10 (21.3)	15 (9.2)	0.02*
NSAIDS	88 (26.1)	13 (16.9)	37 (27.6)	0.08	19 (24.7)	31 (23.1)	0.80	14 (29.8)	36 (22.1)	0.30
Transdermal Lignocaine	63 (18.7)	15 (19.5)	24 (17.9)	0.80	16 (20.8)	23 (17.2)	0.50	8 (17.0)	31 (19.0)	0.80
No discharge analgesics prescribed	34 (10.1)	7 (9.1)	9 (6.7)	0.50	6 (7.8)	10 (7.5)	0.90	5 (10.6)	11 (6.7)	0.40
Number of discharge analgesic agents (Mean (±SD))	2.1 (1.3)	2.1 (1.3)	2.1 (1.2)	0.80	2.2 (1.3)	2.1 (1.2)	0.70	2.3 (1.4)	2.1 (1.2)	0.30
**Complications:**	111 (32.9)	34 (44.2)	33 (24.6)	0.003*	25 (32.5)	42 (31.3)	0.90	18 (38.3)	49 (30.1)	0.30
Type 1 Respiratory Failure	18 (5.4)	2 (2.6)	4 (3.0)	0.90	2 (2.6)	4 (3.0)	0.90	2 (4.3)	4 (2.5)	0.60
Type 2 Respiratory Failure	10 (3.0)	5 (6.5)	1 (0.7)	0.02*	1 (1.3)	5 (3.7)	0.30	4 (8.5)	2 (1.2)	0.02*
Pulmonary Embolism	4 (1.2)	0	1 (0.7)	0.50	0	1 (0.7)	0.50	0	1 (0.6)	1.0
Pneumonia	42 (12.5)	13 (16.9)	12 (9.0)	0.09	11 (14.3)	14 (10.4)	0.40	8 (17.0)	17 (10.4)	0.20
Ventilator Associated Pneumonia	5 (1.5)	3 (3.9)	1 (0.7)	0.10	2 (2.6)	2 (1.5)	0.60	2 (4.3)	2 (1.2)	0.20
ARDS	2 (0.6)	1 (1.3)	0	0.20	0	1 (0.7)	0.50	0	1 (0.6)	1.0

For peer review only

LRTI	58 (17.2)	16 (20.8)	19 (14.2)	0.20	11 (14.3)	24 (17.9)	0.50	7 (14.9)	28 (17.2)	0.70
Treated with Abx	110 (32.6)	29 (37.7)	38 (28.4)	0.20	22 (28.6)	45 (33.6)	0.40	15 (31.9)	52 (31.9)	1.0
**Post Admission Ventilation:**	26 (7.7)	9 (11.7)	5 (3.7)	0.03*	6 (7.8)	8 (6.0)	0.60	5 (10.6)	9 (5.5)	0.20
CPAP	4 (1.2)	2 (2.6)	0	0.06	0	2 (1.5)	0.50	0	2 (1.2)	1.0
BiPAP	1 (0.3)	1 (1.3)	0	0.20	0	1 (0.7)	1.0	0	1 (0.6)	1.0
Invasive Ventilation	17 (5.0)	7 (9.1)	2 (1.5)	0.01*	3 (3.9)	6 (4.5)	1.0	2 (4.3)	7 (4.3)	1.0
High Flow nasal Oxygen	19 (5.6)	6 (7.8)	5 (3.7)	0.20	5 (6.5)	6 (4.5)	0.50	4 (8.5)	7 (4.3)	0.30
Moved to Critical Care	29 (8.6)	10 (13.0)	7 (5.2)	0.05*	6 (7.8)	11 (8.2)	0.90	5 (10.6)	12 (7.3)	0.50
ICU Length of Stay (Mean (±SD))	7.6 (7.4)	9.42 (7.5)	6.42 (5.8)	0.20	7.6 (5.4)	7.8 (7.3)	0.90	9.4 (7.2)	7.4 (6.6)	0.50
Hospital Length of Stay (Mean (±SD))	8.9 (9.5)	11.7 (10.2)	6.9 (5.7)	<0.001*	10.4 (9.3)	7.7 (7.0)	0.03*	9.9 (7.2)	8.3 (8.2)	0.2
**Discharge outcome:**										
Home	306 (90.8)	68 (88.3)	128 (95.5)	0.01*	70 (90.9)	125 (93.3)	0.5	43 (91.5)	152 (93.3)	0.3
Rehabilitation Bed	9 (2.7)	6 (7.8)	0		3 (3.9)	3 (2.2)		3 (6.4)	3 (1.8)	
Repatriation to local hospital	15 (4.5)	3 (3.9)	5 (3.7)		3 (3.9)	5 (3.7)		1 (2.1)	7 (4.3)	
Long term care	4 (1.2)	0	1 (0.7)		1 (1.3)	0		0	1 (0.6)	

For peer review only

Supplementary Table S2: Comparison of characteristics of responders and non-responders

Significance denoted by *<br>Result= n=(%) unless otherwise indicated	Responded at 6 months (n=211)	Non-respondent at 6 months (n=126)	P-value
Age (Mean (SD))	63.8 (16.0)	59.0 (17.0)	0.01*
Male (%)	146 (69.5)	85 (66.9)	0.62
Injury Severity Score (Mean (SD))	11.3 (7.2)	11.1 (7.6)	0.86
Battle/StUMBle Score (Mean (SD))	22.5 (11.2)	21.5 (13.3)	0.48
**Pre-morbid state:**			
Respiratory	42 (20.0)	28 (22.0)	0.65
Cardiac	102 (48.6)	45 (35.4)	0.02*
Neuro	28 (13.3)	22 (17.3)	0.32
MSK	60 (28.6)	29 (22.8)	0.25
Cancer Current	7 (3.3)	2 (1.6)	0.03*
Cancer Historical	22 (10.5)	4 (3.1)	
DM2	20 (9.5)	13 (10.2)	0.81
No of Comorbidities (Mean (SD))	2.3 (2.2)	2.3 (2.2)	0.86
Chronic Pain	19 (9.0)	8 (6.3)	0.37
Regular Analgesic Use	38 (18.1)	23 (18.1)	1.0
**Indicators or Injury severity:**			
No. Rib Fractures (Mean (SD))	5.3 (3.9)	5.0 (4.2)	0.56
Operative Rib Fixation	9 (4.3)	7 (5.5)	0.61
First Rib fracture	19 (9.0)	12 (9.4)	0.90
Bilateral rib fractures	28 (13.3)	16 (12.6)	0.85
**Admission Level of Care (1-3):**			
Level 1	164 (78.1)	88 (69.3)	0.07
Level 2	39 (18.6)	26 (20.5)	
Level 3	7 (3.3)	11 (8.7)	
**Complications:**	66 (31.4)	45 (35.4)	0.39
Type 1 Respiratory Failure	6 (2.9)	12 (9.4)	0.008
Type 2 Respiratory Failure	6 (2.9)	4 (3.1)	0.86
Pulmonary Embolism	1 (0.5)	3 (2.4)	0.12
Pneumonia	25 (11.9)	17 (13.4)	0.65
Ventilator Associated Pneumonia	4 (1.9)	1 (0.8)	0.42
ARDS	1 (0.5)	1 (0.8)	0.71
LRTI	34 (16.2)	24 (18.9)	0.55
Treated with Abx	66 (31.4)	44 (34.6)	0.48
Post Admission Ventilation:	14 (6.7)	12 (9.4)	0.33
ICU Length of Stay (Mean (SD))	7.7 (6.7)	7.4 (8.2)	0.84
Hospital Length of Stay (Mean (SD))	8.6 (8.0)	9.5 (11.7)	0.48
**Discharge outcome:**			
Home	195 (92.9)	111 (87.4)	0.47
Rehabilitation Bed	6 (2.9)	3 (2.4)	
Repatriation to local hospital	8 (3.8)	7 (5.5)	
Long term care	1 (0.5)	3 (2.4)	
**Health Related Quality of Life:**			
Pre-injury SF-12 PCS (Mean (SD))	46.8 (10.4)	45.9 (12.0)	0.44
Pre-injury SF-12 MCS (Mean (SD))	51.2 (9.5)	47.1 (12.1)	0.001*
Discharge SF-12 PCS (Mean (SD))	26.9 (4.4)	27.7 (9.0)	0.43
Discharge SF-12 MCS (Mean (SD))	45.8 (11.6)	41.9 (11.9)	0.004*
1-month SF-12 PCS (Mean (SD))	31.2 (7.67)	33.8 (8.27)	0.09
1-month SF-12 MCS (Mean (SD))	44.8 (11.6)	39.5 (11.4)	0.02*
3-month SF-12 PCS (Mean (SD))	40.2 (11.1)	35.8 (4.03)	0.19
3-month SF-12 MCS (Mean (SD))	48.3 (11.5)	45.6 (9.51)	0.68

For peer review only

Supplementary File: Table S3: Extended Outcomes of interest measured at 6-months after discharge (The total sample)

Time-point	PROM	Min	Max	Mean (±SD)	Dif. from Pop. Norms	Pop Norm
Pre-Injury	SF-12 Physical Component Score	13.1	64.9	46.5 (11.0)	-3.5	50.0 (SD 1.0)
	SF-12 Mental Component Score	13.5	67.2	49.7 (10.7)	-0.3	50.0 (SD 1.0)
	EQ Index Score	-0.3	1.0	0.7 (0.3)	-0.2	0.9 (SE 0.004)
	EQ VAS Score	10.0	100.0	74.9 (19.7)	-7.9	82.8 (SE 0.4)
Discharge	SF-12 Physical Component Score	10.9	55.5	27.2 (8.6)	-22.8	50.0 (SD 1.0)
	SF-12 Mental Component Score	15.9	68.9	44.4 (11.8)	-5.6	50.0 (SD 1.0)
	EQ Index Score	-0.5	1.0	0.3 (0.3)	-0.6	0.9 (SE 0.004)
	EQ VAS Score	0	99.0	47.4 (23.8)	35.4	82.8 (SE 0.4)
	BPI Pain Severity Score	0	10.0	5.0 (2.1)	-	-
	BPI Pain Interference Score	0	10.0	5.7 (2.3)	-	-
	BPI Analgesic Effectiveness	0	100.0	65.3 (24.8)	-	-
	PainDetect Score	-1	29	10.3 (6.0)	-	-
	Prop. reporting Thoracic Pain (%)	-	-	283 (88.4)	-	-
	Mean No. affected body regions (21)	0	16.0	2.5 (2.1)	-	-
1-month	SF-12 Physical Component Score	14.8	55.7	31.6 (7.8)	-18.4	50.0 (SD 1.0)
	SF-12 Mental Component Score	18.4	66.3	43.9 (11.8)	-6.1	50.0 (SD 1.0)
	EQ Index Score	-0.5	1.0	0.5 (0.3)	-0.4	0.9 (SE 0.004)
	EQ VAS Score	5.0	100.0	60.0 (22.7)	-22.8	82.8 (SE 0.4)
	BPI Pain Severity Score	0	10.0	3.8 (2.2)	-	-
	BPI Pain Interference Score	0	10.0	4.4 (2.7)	-	-
	BPI Analgesic Effectiveness	0	100.0	48.4 (30.0)	-	-
	PainDetect Score	0	35.0	8.2 (7.5)	-	-
	Prop. reporting Thoracic Pain (%)	-	-	162 (77.1)	-	-
	Mean No. affected body regions (21)	0	12	3.0 (2.3)	-	-
3-months	SF-12 Physical Component Score	13.3	63.5	40.1 (11.0)	-9.9	50.0 (SD 1.0)
	SF-12 Mental Component Score	19.1	66.8	48.3 (11.4)	-1.7	50.0 (SD 1.0)
	EQ Index Score	-0.5	1.0	0.7 (0.3)	-0.2	0.9 (SE 0.004)
	EQ VAS Score	0	100.0	68.9 (24.4)	-13.9	82.8 (SE 0.4)
	BPI Pain Severity Score	0	9.8	2.9 (2.6)	-	-
	BPI Pain Interference Score	0	9.9	2.6 (2.7)	-	-
	BPI Analgesic Effectiveness	0	100.0	46.5 (35.8)	-	-
	PainDetect Score	0	32.0	8.2 (7.5)	-	-
	Prop. reporting Thoracic Pain (%)	-	-	79 (54.9)	-	-
	Mean No. affected body regions (21)	0	20.0	2.6 (2.7)	-	-
6-months	SF-12 Physical Component Score	12.3	62.1	41.7 (11.5)	-8.3	50.0 (SD 1.0)
	SF-12 Mental Component Score	19.1	65.8	47.8 (11.8)	-2.2	50.0 (SD 1.0)
	EQ Index Score	-0.3	1.0	0.7 (0.3)	-0.2	0.9 (SE 0.004)
	EQ VAS Score	0	100.0	69.2 (26.5)	-13.6	82.8 (SE 0.4)
	BPI Pain Severity Score	0	10.0	2.7 (2.7)	-	-
	BPI Pain Interference Score	0	10.0	2.8 (2.9)	-	-
	BPI Analgesic Effectiveness	0	100.0	49.2 (33.4)	-	-
	PainDetect Score	-1	33	7.5 (7.4)	-	-
	Prop. reporting Thoracic Pain (%)	-	-	99 (46.9)	-	--
	Mean No. affected body regions (21)	0	21	2.6 (3.2)	-	

For peer review only

Supplemental File: Table S4: Extended Outcomes of Interest Data/Candidate predictor variables at six-months after discharge

Mean (±SD)	Outcome Data:	PCS <35 (n=76)	PCS>35 (n=135)	p-value	PSS ≥3.5 (n=77)	PSS <3.5 (n=134)	p-value	PDQ >12 (n=47)	PDQ <12 (n=164)	p-value
Pre-Injury	PCS	40.0 (11.0)	50.8 (7.7)	<0.001*	41.9 (11.4)	49.6 (8.6)	<0.001*	41.7 (11.5)	48.3 (9.6)	<0.001*
	MCS	48.2 (11.7)	53.1 (7.6)	<0.001*	49.1 (10.9)	52.7 (8.4)	0.009*	48.2 (10.6)	52.3 (9.1)	0.009*
	Prop. PCS<20 (n=3) (%)	3 (100)	0	0.022*	3 (100)	0	0.021*	1 (2.2)	2 (1.2)	0.63
	Prop. PCS <30 (n= 22) (%)	17 (77.3)	5 (22.7)	<0.001*	17 (22.4)	5 (3.8)	<0.001*	11 (23.9)	11 (6.7)	0.001*
	Prop. PCS <35 (n=37) (%)	30 (81.1)	7 (18.9)	<0.001*	23 (30.3)	14 (10.5)	<0.001*	16 (34.8)	21 (12.9)	0.001*
Discharge	PCS	24.3 (7.4)	28.4 (8.6)	0.001*	25.1 (8.3)	27.9 (8.3)	0.02*	25.1 (8.4)	27.5 (8.3)	0.09
	MCS	43.5 (11.8)	47.0 (11.5)	0.04*	46.1 (11.5)	45.5 (11.9)	0.76	42.6 (11.7)	46.7 (11.6)	0.04*
	Prop. PCS<20 (n=54) (%)	28 (37.3))	26 (20.0)	0.007*	27 (36.5)	27 (20.8)	0.014*	19 (40.4)	35 (22.3)	0.013*
	Prop. PCS <30 (n=150) (%)	64 (85.3))	86 (66.2)	0.003*	59 (79.7)	90 (69.2)	0.10	39 (83.0)	110 (70.1)	0.08
	Prop. PCS <35 (n=154) (%)	65 (56.7)	89 (68.5)	0.004*	61 (82.4)	93 (71.5)	0.082	36 (76.6)	118 (75.2)	0.84
	BPI Worst Pain	7.4 (2.0)	7.1 (2.2)	0.30	7.3 (2.0)	7.1 (2.3)	0.40	7.5 (1.8)	7.1 (2.2)	0.20
	BPI Least Pain	3.5 (2.7)	3.2 (2.6)	0.40	3.7 (2.7)	3.0 (2.6)	0.06	3.9 (2.6)	3.0 (2.6)	0.04*
	BPI Average Pain	5.4 (2.1)	5.1 (2.0)	0.30	5.4 (2.0)	5.1 (2.1)	0.40	6.7 (2.0)	5.1 (2.1)	0.08
	BPI Current Pain	4.7 (2.8)	3.8 (2.6)	0.01*	4.7 (2.8)	3.8 (2.5)	0.02*	5.3 (2.7)	3.8 (2.6)	0.001*
	BPI Pain Severity Score	5.3 (2.1)	4.8 (2.0)	0.10	5.3 (2.0)	4.7 (2.0)	0.06	5.6 (2.0)	4.7 (2.0)	0.009*
	Prop. PSS >3.5 (n=157) (%)	60 (81.1)	97 (74.6)	0.30	59 (80.8)	97 (74.6)	0.30	40 (87.0)	116 (73.9)	0.07
	Analgesic Effectiveness	57.8 (27.4)	68.4 (22.8)	0.003*	63.3 (26.2)	65.8 (23.8)	0.50	64.3 (23.3)	65.1 (25.1)	0.90
	General Activities	7.8 (2.6)	6.6 (3.0)	0.006*	7.3 (2.7)	6.9 (3.0)	0.30	7.4 (2.8)	6.9 (3.0)	0.30
	Mood	5.5 (3.2)	4.0 (3.2)	0.002*	4.6 (3.0)	4.4 (3.5)	0.70	5.3 (3.2)	4.2 (3.3)	0.05
	Walking	6.9 (3.0)	4.7 (3.1)	<0.001*	6.4 (3.2)	5.0 (3.2)	0.004*	6.8 (3.0)	5.1 (3.2)	0.001*
	Normal Work	8.6 (2.3)	7.4 (3.3)	0.005*	8.4 (2.5)	7.5 (3.3)	0.04*	8.3 (2.4)	7.7 (3.2)	0.20
	Relations	3.6 (3.7)	2.5 (2.9)	0.02*	3.1 (3.3)	2.7 (3.2)	0.50	3.9 (3.5)	2.5 (3.1)	0.02*
	Sleep	6.0 (3.3)	2.3 (3.3)	0.20	5.6 (3.5)	5.5 (3.2)	0.70	6.4 (3.0)	5.3 (3.4)	0.03*
	Enjoyment	7.0 (3.1)	3.0 (3.5)	0.04*	6.2 (3.4)	6.4 (3.4)	0.70	3.9 (3.0)	6.2 (3.5)	0.20
	BPI Pain Interference Score	6.5 (2.0)	5.2 (2.4)	<0.001*	5.9 (2.0)	5.5 (2.4)	0.18	6.4 (2.0)	5.4 (2.3)	0.004*
	PDQ: Low Risk (n=143) (%)	48 (64.9)	95 (73.1)	0.42	47 (63.5)	96 (74.4)	0.08	24 (52.2)	119 (75.8)	0.006*
	PDQ: Moderate Risk (n=44) (%)	18 (24.3)	26 (20.0)		22 (29.7)	21 (16.3)		17 (37.0)	26 (16.6)	
	PDQ: High Risk (n=17) (%)	8 (10.8)	9 (6.9)		5 (6.8)	12 (9.3)		5 (10.9)	12 (7.6)	
1-month	PCS	27.0 (6.0)	33.5 (7.5)	<0.001*	27.7 (6.0)	32.9 (7.8)	<0.001*	27.8 (5.8)	32.1 (7.9)	<0.001*
	MCS	40.8 (12.2)	46.8 (11.2)	0.001*	4.07 (12.7)	46.7 (10.9)	0.001*	35.6 (12.3)	47.0 (10.6)	<0.001*

For peer review only

	Prop. PCS<20 (n=10) (%)	7 (10.9)	3 (2.7)	0.022*	6 (10.0)	4 (3.4)	0.073	3 (8.1)	7 (5.0)	0.47
	Prop. PCS <30 (n=95) (%)	50 (78.1)	45 (39.8)	<0.001*	42 (70.0)	53 (45.3)	0.002*	26 (70.3)	69 (49.3)	0.023*
	Prop. PCS <35 (n=130) (%)	60 (93.8)	70 (61.9)	<0.001*	52 (86.7)	78 (66.7)	0.004*	33 (89.2)	97 (69.3)	0.015*
	BPI Worst Pain	5.7 (2.5)	4.5 (2.3)	0.002*	6.1 (2.2)	4.4 (2.3)	<0.001*	6.6 (2.3)	4.5 (2.3)	<0.001*
	BPI Least Pain	3.6 (2.4)	2.5 (2.2)	0.003*	4.4 (2.3)	2.2 (2.0)	<0.001*	4.5 (2.3)	2.5 (2.1)	<0.001*
	BPI Average Pain	4.8 (2.3)	3.5 (2.0)	<0.001*	5.3 (2.0)	3.2 (2.0)	<0.001*	5.7 (2.2)	3.5 (2.0)	<0.001*
	BPI Current Pain	4.5 (2.6)	2.5 (2.2)	<0.001*	5.0 (2.4)	2.4 (2.1)	<0.001*	5.4 (2.6)	2.7 (2.2)	<0.001*
	BPI Pain Severity Score	4.7 (2.3)	3.2 (1.9)	<0.001*	5.2 (2.0)	3.0 (1.9)	<0.001*	5.5 (2.2)	3.3 (1.9)	<0.001*
	Prop. PSS >3.5 (n=101) (%)	45 (71.4)	56 (49.6)	0.005*	49 (83.1)	52 (44.4)	<0.001*	31 (86.1)	70 (50.0)	<0.001*
	Analgesic Effectiveness	50.0 (24.8)	48.2 (32.7)	0.70	49.7 (23.6)	48.4 (33.1)	0.8	50.0 (27.2)	48.6 (30.8)	0.80
	General Activities	6.1 (2.9)	3.9 (2.6)	<0.001*	6.2 (2.8)	3.9 (2.7)	<0.001*	6.7 (2.8)	4.2 (2.7)	<0.001*
	Mood	4.5 (3.2)	3.1 (2.9)	0.006*	5.0 (3.0)	2.9 (2.9)	<0.001*	5.8 (3.2)	3.0 (2.8)	<0.001*
	Walking	5.8 (3.1)	3.1 (3.0)	<0.001*	5.9 (3.0)	3.1 (3.1)	<0.001*	6.1 (3.2)	3.5 (3.1)	<0.001*
	Normal Work	6.8 (3.1)	4.4 (3.1)	<0.001*	6.8 (2.9)	4.5 (3.2)	<0.001*	7.4 (2.7)	4.7 (3.2)	<0.001*
	Relations	4.0 (3.5)	2.4 (3.0)	0.001*	4.2 (3.3)	2.3 (3.1)	<0.001*	5.3 (3.1)	2.4 (3.0)	<0.001*
	Sleep	5.9 (3.2)	4.8 (3.1)	0.04*	6.7 (2.8)	4.5 (3.1)	<0.001*	7.6 (2.5)	4.6 (3.0)	<0.001*
	Enjoyment	6.2 (3.3)	4.1 (3.1)	<0.001*	6.2 (3.1)	4.2 (3.3	<0.001*	7.0 (3.0)	4.3 (3.2)	<0.001*
	BPI Pain Interference Score	5.6 (2.6)	3.7 (2.5)	<0.001*	5.8 (2.4)	3.6 (2.5)	<0.001*	6.6 (2.4)	3.8 (2.4)	<0.001*
	PDQ: Low Risk (n=122) (%)	39 (61.9)	83 (73.5)		34 (57.6)	88 (75.2)		14 (38.9)	108 (77.1)	
	PDQ: Moderate Risk (n=36) (%)	14 (22.2)	22 (19.5)	0.10	13 (22.0)	23 (19.7)	0.005*	9 (25.0)	27 (19.3)	<0.001*
	PDQ: High Risk (n=18) (%)	10 (15.9)	8 (7.1)		12 (20.3)	6 (5.1)		13 (36.1)	5 (3.6)	
3-months	PCS	31.4 (8.8)	44.4 (9.5)	<0.001*	32.4 (9.1)	43.9 (10.0)	<0.001*	32.9 (8.8)	42.1 (10.8)	<0.001*
	MCS	43.1 (12.9)	50.9 (9.7)	<0.001*	43.1 (11.8)	51.0 (10.4)	<0.001*	40.0 (12.0)	50.7 (10.2)	<0.001*
	Prop. PCS<20 (n=4) (%)	3 (6.4)	1 (1.1)	0.07	3 (6.4)	1 (1.1)	0.071	1 (3.2)	3 (2.7)	0.88
	Prop. PCS <30 (n=32) (%)	24 (51.1)	8 (8.4)	<0.001*	23 (48.9)	9 (9.5)	<0.001*	14 (45.2)	18 (16.2)	0.001*
	Prop. PCS <35 (n=56) (%)	35 (74.5)	21 (22.1)	<0.001*	34 (74.5)	22 (23.2)	<0.001*	21 (67.7)	35 (31.5)	<0.001*
	BPI Worst Pain	5.6 (2.8)	2.9 (2.5)	<0.001*	6.2 (2.2)	2.6 (2.4)	<0.001*	5.9 (2.2)	3.2 (2.8)	<0.001*
	BPI Least Pain	3.4 (2.9)	1.7 (2.3)	0.001*	4.4 (2.7)	1.2 (1.9)	<0.001*	4.0 (2.7)	1.8 (2.4)	<0.001*
	BPI Average Pain	4.6 (2.8)	2.3 (2.3)	<0.001*	5.3 (2.4)	2.0 (2.0)	<0.001*	5.0 (2.4)	2.5 (2.5)	<0.001*
	BPI Current Pain	4.1 (3.2)	1.8 (2.3)	<0.001*	4.7 (2.7)	1.5 (2.2)	<0.001*	4.4 (2.8)	2.0 (2.7)	<0.001*
	BPI Pain Severity Score	4.4 (2.7)	2.2 (2.2)	<0.001*	5.1 (2.2)	1.8 (2.0)	<0.001*	4.8 (2.2)	2.4 (2.4)	<0.001*
	Prop. PSS >3.5 (n=53) (%)	27 (58.7)	26 (27.4)	<0.001*	35 (74.5)	18 (19.1)	<0.001*	23 (74.2)	30 (27.3)	<0.001*
	Analgesic Effectiveness	40.0 (32.1)	50.1 (37.6)	0.10	48.5 (29.6)	44.5 (40.0)	0.60	48.7 (31.3)	45.3 (37.5)	0.70
	General Activities	4.8 (3.5)	2.1 (2.6)	<0.001*	5.7 (3.0)	1.6 (2.2)	<0.001*	5.6 (3.0)	2.2 (2.8)	<0.001*

For peer review only

	Mood	4.0 (3.5)	1.5 (2.3)	<0.001*	4.6 (3.1)	1.2 (2.2)	<0.001*	5.1 (3.1)	1.6 (2.5)	<0.001*
	Walking	4.9 (3.8)	1.4 (2.4)	<0.001*	5.0 (3.4)	1.4 (2.6)	<0.001*	4.4 (3.3)	2.1 (3.2)	0.001*
	Normal Work	5.4 (3.7)	2.0 (2.7)	<0.001*	5.7 (3.2)	1.8 (2.7)	<0.001*	5.6 (3.2)	2.5 (3.2)	<0.001*
	Relations	2.5 (3.0)	1.1 (2.1)	0.001*	3.2 (3.0)	0.8 (1.7)	<0.001*	3.8 (3.2)	1.0 (1.8)	<0.001*
	Sleep	4.2 (3.4)	2.2 (2.7)	<0.001*	4.9 (3.1)	1.9 (2.5)	<0.001*	5.1 (3.2)	2.3 (2.7)	<0.001*
	Enjoyment	5.0 (3.7)	2.0 (2.6)	<0.001*	5.7 (3.4)	1.7 (2.4)	<0.001*	5.3 (3.6)	2.4 (3.0)	<0.001*
	BPI Pain Interference Score	4.4 (3.1)	1.8 (2.1)	<0.001*	5.0 (2.6)	1.5 (2.0)	<0.001*	5.0 (2.6)	2.0 (2.4)	<0.001*
	PDQ: Low Risk (n=107) (%)	28 (60.9)	79 (83.2)		23 (48.9)	84 (89.4)		9 (29.0)	98 (89.1)	
	PDQ: Moderate Risk (n=15) (%)	7 (15.2)	8 (8.4)	0.012*	9 (19.1)	6 (6.4)	<0.001*	9 (29.0)	6 (5.5)	<0.001*
	PDQ: High Risk (n=19) (%)	11 (23.9)	8 (8.4)		15 (31.9)	4 (4.3)		13 (41.9)	6 (5.5)	
6-months	PCS	29.2 (5.1)	48.9 (7.2)	<0.001*	32.9 (8.0)	46.8 (10.1)	<0.001*	32.4 (8.5)	44.4 (10.9)	<0.001*
	MCS	42.3 (13.2)	51.0 (9.7)	<0.001*	39.4 (11.6)	52.7 (8.9)	<0.001*	36.4 (11.8)	51.1 (9.6)	<0.001*
	Prop. PCS<20 (n=4) (%)	-	-	-	3 (4.0)	1 (0.7)	0.1	3 (6.4)	1 (0.6)	0.01*
	Prop. PCS <30 (n=42) (%)	-	-	-	30 (39.0)	12 (9.0)	<0.001*	20 (42.6)	22 (13.4)	<0.001*
	Prop. PCS <35 (n=76) (%)	-	-	-	51 (66.2)	25 (18.8)	<0.001*	32 (68.1)	44 (26.8)	<0.001*
	BPI Worst Pain	5.8 (4.5)	2.1 (2.4)	<0.001*	6.5 (1.8)	1.6 (2.0)	<0.001*	6.3 (2.2)	2.6 (2.7)	<0.001*
	BPI Least Pain	4.1 (3.0)	1.2 (2.0)	<0.001*	5.2 (2.4)	0.6 (0.9)	<0.001*	4.9 (2.8)	1.5 (2.2)	<0.001*
	BPI Average Pain	5.0 (2.6)	1.6 (2.0)	<0.001*	5.9 (1.8)	1.1 (1.3)	<0.001*	5.4 (2.4)	2.1 (2.4)	<0.001*
	BPI Current Pain	4.5 (3.0)	1.2 (2.0)	<0.001*	5.6 (2.2)	0.5 (0.9)	<0.001*	5.2 (2.7)	1.6 (2.4)	<0.001*
	BPI Pain Severity Score	4.8 (2.6)	1.5 (2.0)	<0.001*	5.8 (1.8)	0.9 (1.1)	<0.001*	5.4 (2.3)	1.9 (2.3)	<0.001*
	Prop. PSS >3.5 (n=77) (%)	51 (67.1)	26 (19.3)	<0.001*	-	-	-	37 (78.7)	40 (24.5)	<0.001*
	Analgesic Effectiveness	45.6 (28.6)	53.0 (37.6)	0.22	46.6 (26.5)	52.7 (41.0)	0.32	49.1 (24.7)	49.2 (37.8)	1.0
	General Activities	5.7 (3.0)	1.5 (2.2)	<0.001*	6.2 (2.2)	1.2 (2.1)	<0.001*	6.3 (2.7)	2.1 (2.7)	<0.001*
	Mood	4.5 (3.3)	1.3 (2.1)	<0.001*	5.0 (3.0)	1.0 (1.9)	<0.001*	6.1 (2.9)	1.4 (2.1)	<0.001*
	Walking	6.0 (3.5)	1.3 (2.2)	<0.001*	6.0 (3.1)	1.3 (2.4)	<0.001*	6.2 (3.4)	2.1 (2.9)	<0.001*
	Normal Work	6.0 (3.2)	1.6 (2.5)	<0.001*	6.4 (2.8)	1.3 (2.3)	<0.001*	6.5 (3.0)	2.3 (3.0)	<0.001*
	Relations	3.8 (3.4)	0.8 (1.8)	<0.001*	4.26 (3.2)	0.5 (1.4)	<0.001*	5.0 (3.1)	1.0 (2.0)	<0.001*
	Sleep	5.0 (3.3)	1.9 (2.6)	<0.001*	5.6 (2.8)	1.6 (2.4)	<0.001*	6.5 (2.6)	2.05 (2.6)	<0.001*
	Enjoyment	5.5 (3.2)	1.5 (2.4)	<0.001*	6.0 (2.7)	1.2 (2.2)	<0.001*	6.4 (3.2)	2.0 (2.6)	<0.001*
	BPI Pain Interference Score	5.2 (2.7)	1.4 (2.0)	<0.001*	5.6 (2.3)	1.2 (1.7)	<0.001*	6.1 (2.6)	1.8 (2.2)	<0.001*
	PDQ: Low Risk (n=) (%)	44 (57.9)	119 (88.8)		40 (51.9)	123 (92.5)		0	163 (100)	
	PDQ: Moderate Risk (n=) (%)	17 (22.4)	11 (8.2)	<0.001*	20 (26.0)	8 (6.0)	<0.001*	28 (59.6)	0	<0.001*
	PDQ: High Risk (n=) (%)	15 (19.7)	4 (3.0)		17 (22.1)	2 (1.5)		19 (40.4)	0	

For peer review only

For peer review only

STROBE Statement—checklist of items that should be included in reports of observational studies

	Item No.	Recommendation	Page No.
**Title and abstract**	1	(*a*) Indicate the study's design with a commonly used term in the title or the abstract	1
		(*b*) Provide in the abstract an informative and balanced summary of what was done and what was found	1
**Introduction**			
Background/rationale	2	Explain the scientific background and rationale for the investigation being reported	2
Objectives	3	State specific objectives, including any prespecified hypotheses	3
**Methods**			
Study design	4	Present key elements of study design early in the paper	3
Setting	5	Describe the setting, locations, and relevant dates, including periods of recruitment, exposure, follow-up, and data collection	3
Participants	6	(*a*) *Cohort study*—Give the eligibility criteria, and the sources and methods of selection of participants. Describe methods of follow-up<br>*Case-control study*—Give the eligibility criteria, and the sources and methods of case ascertainment and control selection. Give the rationale for the choice of cases and controls<br>*Cross-sectional study*—Give the eligibility criteria, and the sources and methods of selection of participants	3 &4
		(*b*) *Cohort study*—For matched studies, give matching criteria and number of exposed and unexposed<br>*Case-control study*—For matched studies, give matching criteria and the number of controls per case	N/A
Variables	7	Clearly define all outcomes, exposures, predictors, potential confounders, and effect modifiers. Give diagnostic criteria, if applicable	5
Data sources/ measurement	8*	For each variable of interest, give sources of data and details of methods of assessment (measurement). Describe comparability of assessment methods if there is more than one group	5
Bias	9	Describe any efforts to address potential sources of bias	4-6
Study size	10	Explain how the study size was arrived at	4

Continued on next page

For peer review only - http://bmjopen.bmj.com/site/about/guidelines.xhtml

For peer review only

Quantitative variables	11	Explain how quantitative variables were handled in the analyses. If applicable, describe which groupings were chosen and why	6
Statistical methods	12	(*a*) Describe all statistical methods, including those used to control for confounding	6
		(*b*) Describe any methods used to examine subgroups and interactions	6
		(*c*) Explain how missing data were addressed	6
		(*d*) *Cohort study*—If applicable, explain how loss to follow-up was addressed<br>*Case-control study*—If applicable, explain how matching of cases and controls was addressed<br>*Cross-sectional study*—If applicable, describe analytical methods taking account of sampling strategy	6
		(*e*) Describe any sensitivity analyses	-
**Results**			
Participants	13*	(a) Report numbers of individuals at each stage of study—eg numbers potentially eligible, examined for eligibility, confirmed eligible, included in the study, completing follow-up, and analysed	7
		(b) Give reasons for non-participation at each stage	7
		(c) Consider use of a flow diagram	-
Descriptive data	14*	(a) Give characteristics of study participants (eg demographic, clinical, social) and information on exposures and potential confounders	7
		(b) Indicate number of participants with missing data for each variable of interest	7
		(c) *Cohort study*—Summarise follow-up time (eg, average and total amount)	7
Outcome data	15*	*Cohort study*—Report numbers of outcome events or summary measures over time	9
		Case-control study—Report numbers in each exposure category, or summary measures of exposure	-
		Cross-sectional study—Report numbers of outcome events or summary measures	-
Main results	16	(*a*) Give unadjusted estimates and, if applicable, confounder-adjusted estimates and their precision (eg, 95% confidence interval). Make clear which confounders were adjusted for and why they were included	8-10
		(*b*) Report category boundaries when continuous variables were categorized	8-10
		(*c*) If relevant, consider translating estimates of relative risk into absolute risk for a meaningful time period	N/A

Continued on next page

For peer review only

Other analyses	17	Report other analyses done—eg analyses of subgroups and interactions, and sensitivity analyses	8-10
**Discussion**			
Key results	18	Summarise key results with reference to study objectives	-
Limitations	19	Discuss limitations of the study, taking into account sources of potential bias or imprecision. Discuss both direction and magnitude of any potential bias	10-12
Interpretation	20	Give a cautious overall interpretation of results considering objectives, limitations, multiplicity of analyses, results from similar studies, and other relevant evidence	10-12
Generalisability	21	Discuss the generalisability (external validity) of the study results	10-12
**Other information**			
Funding	22	Give the source of funding and the role of the funders for the present study and, if applicable, for the original study on which the present article is based	Declarations section

*Give information separately for cases and controls in case-control studies and, if applicable, for exposed and unexposed groups in cohort and cross-sectional studies.

Note: An Explanation and Elaboration article discusses each checklist item and gives methodological background and published examples of transparent reporting. The STROBE checklist is best used in conjunction with this article (freely available on the Web sites of PLoS Medicine at http://www.plosmedicine.org/, Annals of Internal Medicine at http://www.annals.org/, and Epidemiology at http://www.epidem.com/). Information on the STROBE Initiative is available at www.strobe-statement.org.

For peer review only - http://bmjopen.bmj.com/site/about/guidelines.xhtml
